# Complete response to BRICS in Locally advanced pancreatic cancer (pMMR, CPS 30): a case report

**DOI:** 10.3389/fimmu.2026.1743752

**Published:** 2026-01-21

**Authors:** Songlong Yang, Liangxiu Shao, Yating Wu, Yonghai Peng, Weiqiang Fan

**Affiliations:** 1 Department of Spleen-Stomach-Hepatobiliary, Quanzhou Hospital of Traditional Chinese Medicine, Quanzhou, Fujian, China; 2 Department of Pathology, 900th Hospital of PLA Joint Logistic Support Force, Fuzhou, Fujian, China; 3 Department of Oncology, The CangShan District of the 900th Hospital, Fuzhou, Fujian, China

**Keywords:** complete remission, immunotherapy, locally advanced, pancreatic cancer, PD-L1, PMMR, SBRT, toripalimab

## Abstract

**Background:**

Locally advanced pancreatic cancer (LAPC) has a dismal prognosis, marked by an exceedingly low 5-year survival rate. While immune checkpoint inhibitors (ICIs) have demonstrated efficacy across various solid tumors, their application in the treatment of pancreatic ductal adenocarcinoma—particularly in mismatch repair–proficient (pMMR) cases—is restricted by the immunosuppressive nature of the tumor microenvironment (TME). Thus, more effective treatment strategies for pMMR LAPC are urgently needed.

**Case presentation:**

We present a 65-year-old female diagnosed with LAPC (cT4N1M0, Stage III), confirmed pathologically as pancreatic ductal adenocarcinoma with pMMR status and a high programmed death-ligand 1 (PD-L1) combined positive score (CPS) of 30. Initial tumor markers were significantly elevated (CA19-9: 62,228.8 U/L; CEA: 100 ng/mL). The patient received a novel multimodal treatment referred to as the BRICS Regimen, an acronym derived from Bifidobacterium supplementation, Radiotherapy (hypofractionated), Immunotherapy (PD-1 inhibitors), Chemotherapy (low-dose), and Stereotactic approach. This protocol can be modified to match the individual disease characteristics. In this case, the treatment comprised SBRT 24 Gy/3 fractions → q21d toripalimab 240 mg + nab-paclitaxel 200→100 mg + anlotinib 12 mg d1–14, with continuous Bifidobacterium triple viable tablets. Imaging following treatment indicated a complete response (CR), and tumor markers remained normal for 5 months post-therapy. The treatment was well tolerated, with no severe adverse events reported.

**Conclusion:**

This report suggests that a combined modality approach—integrating SBRT, chemotherapy, antiangiogenic therapy, ICI, and probiotics—may achieve CR in patients presenting with pMMR LAPC and high PD-L1 expression, even without surgery. These results challenge the prevailing assumption that pMMR status invariably predicts resistance to immunotherapy. These findings suggest that, in pMMR pancreatic cancers with high PD-L1 CPS, multimodal treatment strategies may remodel the tumor microenvironment and overcome immune resistance, highlighting a promising therapeutic direction.

## 1 Introduction

Pancreatic cancer ranks among the most lethal malignancies affecting the digestive system, with a growing incidence and a grim prognosis, as evidenced by an overall 5-year survival rate below 10% (1). More than half of patients are diagnosed with distant metastasis, while 30-35% present with locally advanced pancreatic cancer (LAPC) (2). The standard treatment for LAPC primarily consists of chemotherapy options such as FOLFIRINOX or gemcitabine combined with nab-paclitaxel, occasionally supplemented by radiotherapy. However, the median overall survival remains dismal under one year, underscoring the urgent need for more effective therapeutic strategies (3).

Although immune checkpoint inhibitors (ICIs) for PD-1/PD-L1 have improved the management of advanced solid tumors (4), pancreatic cancer largely remains resistant to ICI monotherapy. This resistance is primarily due to its distinctive biological characteristics, such as a low tumor mutational burden (TMB), a dense desmoplastic stroma, and a highly immunosuppressive TME characterized by regulatory T cells (Tregs), myeloid-derived suppressor cells (MDSCs), and tumor-associated macrophages (TAMs). Together, these factors contribute to the classification of pancreatic cancer as an immunologically “cold” tumor (5). Currently, ICIs are only recommended for the small fraction (1-2%) of pancreatic cancer patients who present with high microsatellite instability (MSI-H) or mismatch repair deficiency (dMMR) (6). Consequently, devising effective immunotherapeutic strategies for the majority of pMMR pancreatic cancers remains a significant challenge.

PD-L1 expression serves as another predictive biomarker for ICIs, with reports indicating that 71% of pancreatic cancers (7). Nevertheless, its predictive value in pMMR pancreatic cancer is unclear. Here, we report an exceptional case of a patient with pMMR LAPC whose tumor exhibited a remarkably high PD-L1 Combined Positive Score (CPS) of 30. A novel multimodal regimen known as the BRICS Regimen, an acronym derived from Bifidobacterium supplementation, Radiotherapy (hypofractionated), Immunotherapy (PD-1 inhibitors), Chemotherapy (low-dose), and Stereotactic approach, which can be adjusted to match the individual disease characteristics. Notably, this regimen achieved both radiographic and serologic complete responses, with sustained remission following treatment cessation. The BRICS regimen was specifically designed to enhance antitumor immunity, particularly in patients who show resistance to immunotherapy. Our previous research in PD-L1-negative, EGFR/ALK wild-type metastatic NSCLC demonstrated good efficacy and a safety profile, suggesting that this approach can effectively alleviate intrinsic immunotherapy resistance (8). This case highlights the clinical significance of the rare pMMR/PD-L1-high subtype and elucidates the mechanisms by which multimodal combination therapy achieves synergistic antitumor effects.

## 2 Case presentation

**Figure 1:**
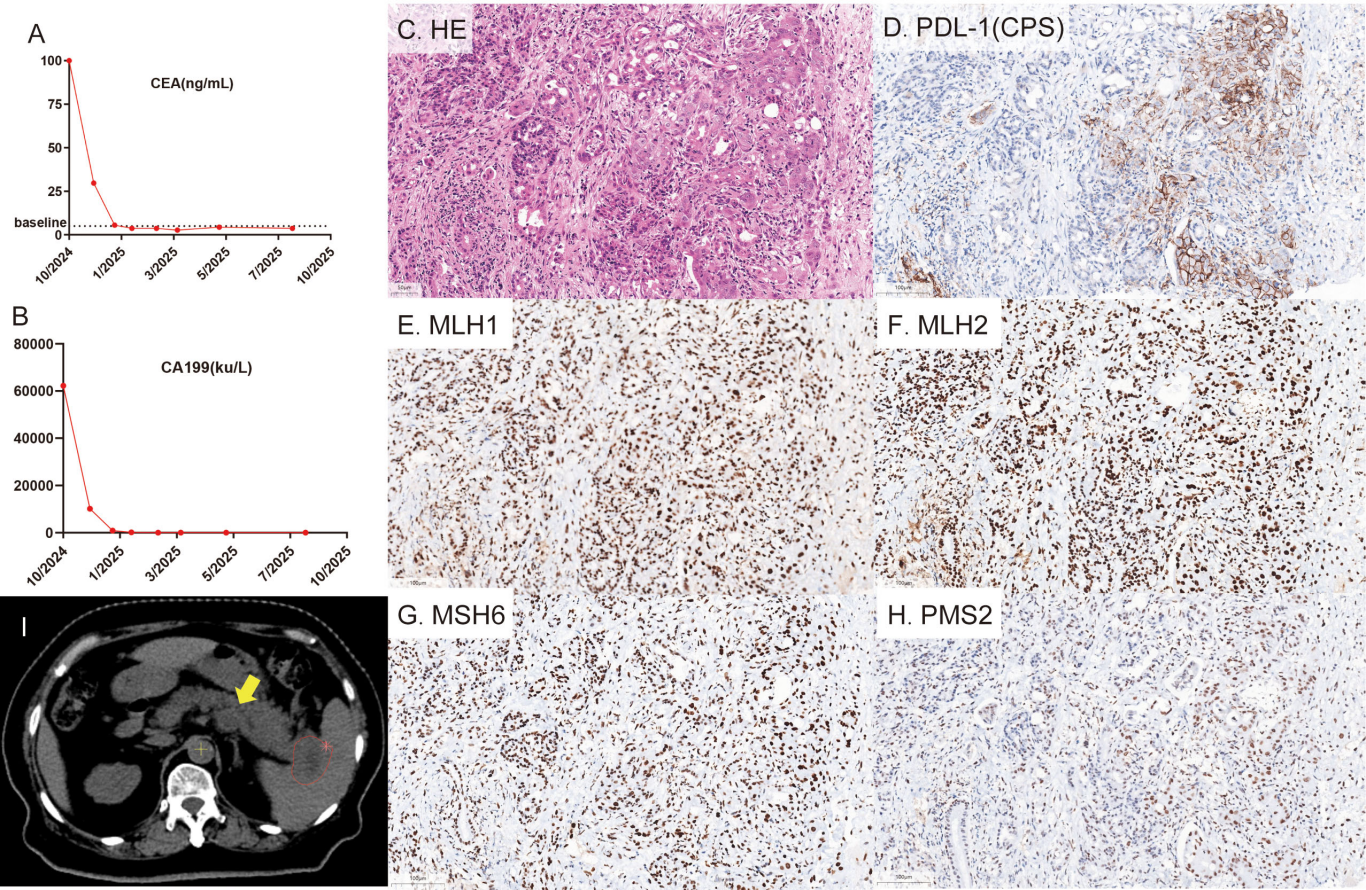
**(A, B)** Temporal changes in serum tumor marker levels, generated using GraphPad Prism software (version 10). **(C–H)** Histopathological and immunohistochemical (IHC) characterization of the pancreatic lesion. **(C)** Hematoxylin and eosin (H&E) staining reveals the tumor morphology. **(D)** Immunohistochemical staining for PD-L1 expression, showing a combined positive score (CPS) of 30. **(E-H)** Representative IHC staining for DNA mismatch repair (MMR) proteins demonstrates intact nuclear expression of MLH1 **(E)**, MSH2 **(F)**, MSH6 **(G)**, and PMS2 **(H)**, consistent with proficient MMR (pMMR) status. All photomicrographs were acquired at 20× original magnification; scale bars = 100 μm (indicated in the lower-left corner of each panel). **(I)**. Delineation of the gross tumor volume (GTV). The red contour outlines the primary GTV, and yellow arrows indicate non-irradiated metastatic lesions.

**Figure 2:**
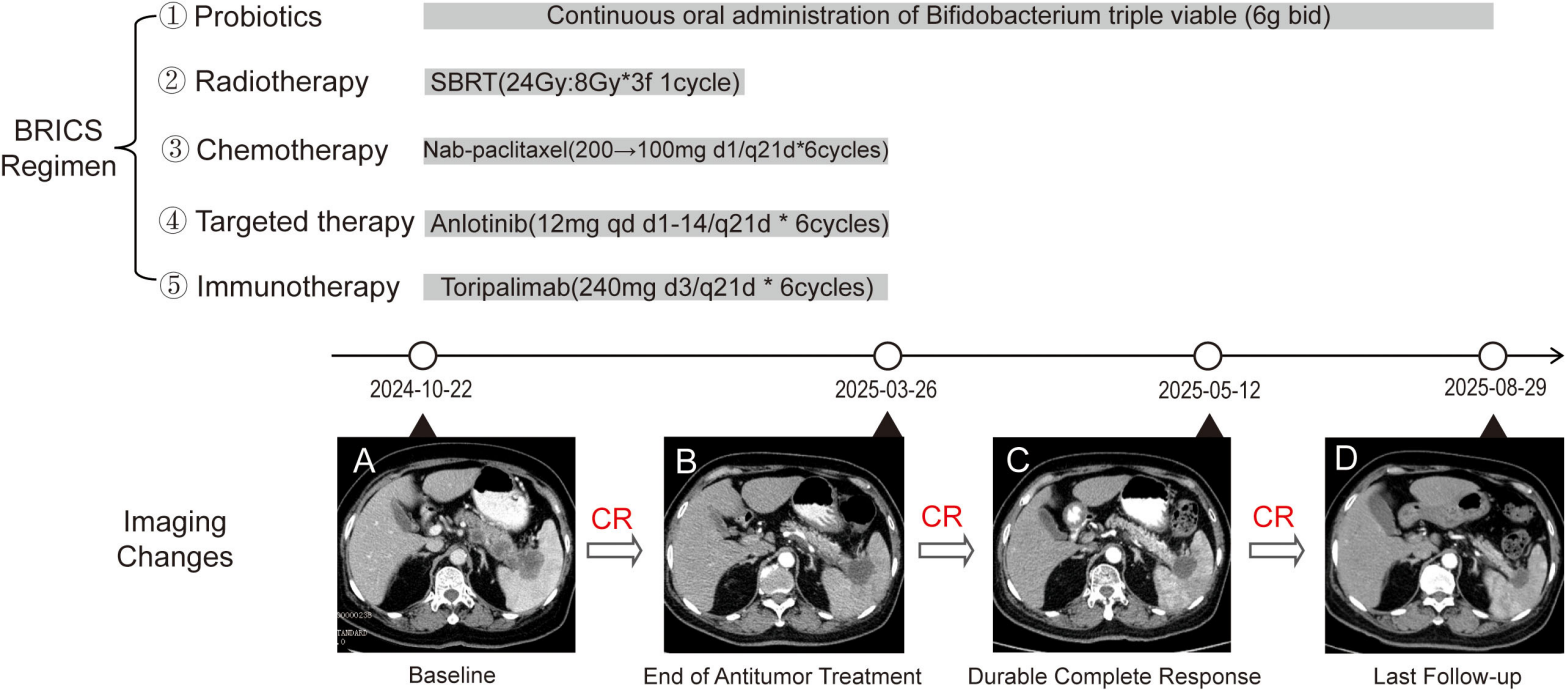
Timeline of the therapeutic intervention and image changes.

In October 2024, a 65-year-old woman visited our hospital due to a one-month history of persistent epigastric pain radiating to her back. Her family and medical history were unremarkable. Physical examination detected mild tenderness in the epigastric region, with an Eastern Cooperative Oncology Group (ECOG) performance status of 2. The pain intensity was rated as 4 on a 0–10 numerical rating scale (NRS). Laboratory tests indicated significantly elevated tumor markers: carcinoembryonic antigen (CEA) at 100 ng/mL and carbohydrate antigen 19-9 (CA19-9) at 62,228.8 ku/L(**Figures 1A, B**). A contrast-enhanced abdominal CT scan revealed multiple pancreatic mass lesions, suggestive of pancreatic malignancy, with infiltration into the splenic artery, vein, and spleen, as well as retroperitoneal lymph node metastases (**Figure 2A**, **Supplementary Figure S1**), staging the disease as cT4N1M0, Stage III. A biopsy ultimately confirmed the diagnosis of pancreatic ductal adenocarcinoma.

Immunohistochemistry (IHC) (**Supplementary Table S1**, **Supplementary Figure S2**) indicated proficient mismatch repair (pMMR) status, characterized by the intact expression of MLH1, MSH2, MSH6, and PMS2 proteins. Notably, PD-L1 testing (22C3 antibody) revealed a high Combined Positive Score (CPS) of 30(**Figures 1C-H**), implying a potentially immunoresponsive tumor microenvironment despite the pMMR status—a rare molecular phenotype in pancreatic cancer.

Considering the locally advanced disease and the patient’s refusal of surgery and standard chemotherapy, the choice of personalized regimen was selected based on both the encouraging precedent of our previous case of advanced pancreatic neuroendocrine carcinoma, which achieved over 7-year CR (9) and good biomarker profile (pMMR with high PD-L1 CPS). Therefore, a multimodal therapeutic strategy was devised to initiate and potentiate anti-tumor immunity. The treatment began with stereotactic body radiotherapy (SBRT) targeting the primary pancreatic lesion, delivering a total of 24 Gy in three fractions to promote immunogenic cell death. As a safety precaution, the radiotherapy target volume was limited to the pancreatic tail and splenic hilum, with the elective omission of some lesions located in the pancreatic body (**Figure 1I**, **Supplementary Figure S3**). Five days after completing SBRT, systemic therapy was initiated, consisting of six 21-day cycles that included Toripalimab (240 mg, Q3W), nab-paclitaxel (200 mg on day 1, reduced to 100 mg starting from cycle 3 due to grade 2 neutropenia), and Anlotinib (12 mg, days 1-14). Additionally, oral *Bifidobacterium* triple viable tablets (*Bifidobacterium longum, Lactobacillus bulgaricus, Streptococcus thermophilus*) were administered throughout the treatment to modulate the gut microbiome (**Table 1**).

**Table 1:** Timeline of diagnosis and treatment.

Timeline	Regimen/ Occurrence	Detail	Duration	Cycles	CEA (ng/mL)	CA199 (ku/L)
2024-10	Diagnosis	LAPC(T4N1M0, Stage III) Ki-67 80%, CPS: 30, pMMR	-	-	100	62228.8
2024-10-26	SBRT	Site: pancreatic lesion; Dose: 24 Gy/3 fractions(8 Gy per fraction)	3day	1	–	–
2024-10-26	Probiotics	Bifidobacterium triple viable 6g bid	Continuous	Continuous	–	–
2024-11-01	Combination Therapies	Nab-paclitaxel 200mg d1+Toripalimab 240 mg d3+Anlotinib 12mg d1-14/q21d	14day	1/6	–	–
2024-11-27	Combination Therapies	Nab-paclitaxel 200mg d1+Toripalimab 240 mg d3+Anlotinib 12mg d1-14/q21d	14day	2/6	29.7	10192.5
2024-12-26	Combination Therapies	Nab-paclitaxel 100mg d1+Toripalimab 240 mg d3+Anlotinib 12mg d1-14/q21d	14day	3/6	5.56	924.3
2025-01-16	Combination Therapies	Nab-paclitaxel 100mg d1+Toripalimab 240 mg d3+Anlotinib 12mg d1-14/q21d	14day	4/6	3.71	164.3
2025-02-20	Combination Therapies	Nab-paclitaxel 100mg d1+Toripalimab 240 mg d3+Anlotinib 12mg d1-14/q21d	14day	5/6	3.69	51.3
2025-03-27	Combination Therapies	Nab-paclitaxel 100mg d1+Toripalimab 240 mg d3+Anlotinib 12mg d1-14/q21d	14day	6/6	2.67	31.4
2025-05-12	Follow-up	–	–	–	4.35	30.7
2025-08-18	Follow-up	–	–	–	3.74	23.7

The treatment was well-tolerated, with manageable grade 2 neutropenia observed. After two cycles, CA19–9 and CEA levels significantly decreased to 924.3 ku/L and 5.56 ng/mL, respectively, accompanied by notable tumor shrinkage on imaging. The pain score was reduced from a baseline of 4 to 0(indicating a shift from moderate pain to no pain). Upon completing six cycles in March 2025, tumor markers normalized (CA19-9: 31.4 ku/L, CEA: 2.67 ng/mL), and a follow-up CT scan confirmed a complete response (RECIST 1.1) (**Figures 2B, C**, **Supplementary Figure S4**). As of August 18, 2025, the patient remains in complete remission, with an ECOG score of 0 and stable serial tumor markers and imaging (**Figures 1A, B**, **2D**, **Supplementary Figure S5**).

## 3 Discussion

This case provides an important insight into the treatment of pMMR pancreatic cancer, demonstrating that pMMR status can coexist with a high PD-L1 combined positive score. This biomarker profile may inform the rational selection of synergistic, multimodal treatment strategies, as supported by the favorable clinical outcome observed in this patient.

Conventional understanding suggests that pMMR/MSS tumors generally exhibit a poor response to immune checkpoint inhibitors (ICIs) due to their low tumor mutational burden (TMB) and limited neoantigen generation (10). However, the case presented here—a patient with locally advanced pancreatic cancer characterized by pMMR who achieved a complete response (CR)—challenges the prevailing notion that MMR status is the definitive predictor of ICI efficacy in pancreatic cancer. A key finding in this case was a high Combined Positive Score (CPS) of 30. The CPS indicates PD-L1 presence on immune and tumor cells; a high score signifies a pre-existing but suppressed antitumor T-cell response within the tumor microenvironment. This suggests that PD-L1 expression may act as an independent and potentially dominant predictive biomarker for ICI efficacy in pancreatic cancer, regardless of MMR status. The mechanisms driving elevated PD-L1 expression in pMMR tumors are not fully understood but may involve specific oncogenic mutations, such as KRAS and TP53, or inflammatory signaling pathways like IFN-γ pathway activation (11, 12). This case strongly advocates for the routine evaluation of PD-L1 expression in all pancreatic cancer patients, as those with high expression levels may still benefit from immunotherapy, even within the pMMR subset.

As precision oncology continues to transform cancer treatment paradigms, identifying mismatch repair deficiency (dMMR) or microsatellite instability-high (MSI-H) has become crucial for predicting therapeutic responses to ICIs across various solid tumors. However, depending solely on MSI-H/d-MMR status to guide immunotherapy decisions—especially in pancreatic cancer and other guideline-recommended tumor types—may pose significant limitations, as illustrated in **Table 2** (7, 13–25), pancreatic cancer has a notably low incidence of MSI-H/d-MMR (<5%), while exhibiting a wide range of PD-L1 combined positive score (CPS ≥1) positivity rates, from 13.5% to 71%. This discrepancy indicates that a considerable number of patients with PD-L1-positive tumors may be excluded from the potential benefits of ICIs if selection is solely based on the MSI-H/d-MMR status.

**Table 2:** Prevalence of MSI-H/d-MMR and PD-L1 (CPS) in tumors types where MMR/MSI testing is guideline-recommended.

Tumor type	Incidence of MSI-H/d-MMR (%)	Positivity Rate of CPS (CPS ≥1) (%)
Colorectal Cancer	6–19	87–95
	Ref. (13–17)	Ref. (19, 20)
Endometrial Cancer	17–33	60
	Ref. (14, 15, 17)	Ref. (21)
Gastric Cancer	8–22	49.4–72.7
	Ref. (14–17)	Ref. (22–25)
Ovarian Cancer	2–12	34.3
	Ref. (15, 17, 18)	Ref. (21)
Pancreatic Cancer	< 5	13.5–71
	Ref. (15–17)	Ref. (7, 20)
Biliary Tract Cancer	< 5	NA
	Ref. (15, 16)	

A similar trend is observed in other cancers. Colorectal cancer has a moderate incidence of MSI-H/d-MMR (6–19%) but features an exceptionally high CPS positivity rate (87–95%). Furthermore, endometrial and gastric cancers exhibit significant CPS expression, with rates of 60% and 49.4–72.7%, respectively, despite differing rates of MSI-H/d-MMR prevalence. In ovarian cancer, although MSI-H/dMMR is infrequent (2–12%), PD-L1 expression is found in more than one-third of tumors, underscoring the broader relevance of immune checkpoint pathways. This relevance is further illustrated by the phase III KEYNOTE-062 trial, which demonstrated that pembrolizumab provided a clinically meaningful overall survival improvement compared to chemotherapy in patients with advanced gastric cancer and PD-L1 CPS ≥10. Notably, this benefit persisted even after excluding MSI-H tumors (26).

Collectively, these findings show that tumors classified as MSS/p-MMR yet PD-L1-positive may be sensitive to immunotherapy. Therefore, employing a one-dimensional biomarker strategy risks missing patients who could benefit from ICIs. Consequently, integrating multiplexed biomarker approaches, such as PD-L1 CPS alongside MSI-H/d-MMR testing, can enhance patient stratification and maximize therapeutic efficacy, particularly in tumors characterized by low MSI-H/d-MMR frequencies but significant immune activation.

The success of this case arises not from a single therapeutic agent, but from the synergistic interaction of five distinct modalities. We posit that this integrated approach effectively surmounted the significant immunosuppressive barriers typical of pancreatic cancer. The regimen commenced with SBRT (8Gy*3f), which was utilized not solely for local tumor debulking, but also to leverage its “*in situ* vaccine” effect. This specific fractionation promotes immunogenic cell death (ICD), inducing the release of damage-associated molecular patterns (DAMPs) and tumor-associated antigens (TAAs) (27, 28), while also upregulating tumor MHC-I expression, promoting dendritic cell (DC) maturation, and enhancing intratumoral T-cell infiltration (29). Unlike conventional radiotherapy, which is often limited by the emergence of radioresistance and limitations imposed by surrounding healthy tissues, this approach creates a strong immunopermissive microenvironment for subsequent PD-1 blockade (30, 31).

Nab-paclitaxel played a dual role by not only exerting direct cytotoxic effects and facilitating antigen release but also through immunomodulation. It reduces the number of immunosuppressive MDSCs and Tregs, thereby enhancing the efficacy of PD-1 inhibition (32). Furthermore, anlotinib, a multi-targeted antiangiogenic TKI, normalized tumor vasculature to alleviate hypoxia and remodel the tumor immune microenvironment (TME). This facilitated effector T-cell infiltration and potentially suppressed VEGFR-positive immunosuppressive cells like Tregs and TAMs, thereby removing a critical barrier to PD-1 inhibitor efficacy (33). Finally, sustained supplementation with Bifidobacterium may have strengthened systemic immune responses, corroborating findings suggesting that specific gut microbiota enhance the efficacy of ICIs (34). However, its precise effects need to be further studied. Collectively, this multi-modal approach sequentially primed the immune response (SBRT), amplified antigenicity (chemotherapy), restored the TME (antiangiogenics), and enhanced the T-cell activity (PD-1 inhibitor), creating a coherent chain of antitumor immune reactions.

Despite the immunosuppressive feature of the TME and the low tumor mutational burden characteristic of pancreatic ductal adenocarcinoma (PDAC), which inhibits the effectiveness of ICIs as monotherapy, it has been shown that immunotherapy is effective in a subset of non-dMMR/MSI-H patients. In locally advanced PDAC, neoadjuvant or conversion therapy incorporating ICIs combined with chemotherapy or radiotherapy has facilitated successful R0 resection and even pathological complete response (pCR) in some cases (35, 36). For instance, near-complete pathological responses have been observed following radiotherapy combined with anti-PD-1 therapy, suggesting that radiation may remodel the tumor immune landscape and synergize with ICIs (35). In metastatic conditions, personalized neoantigen nanovaccines administered together with ICIs have been reported to induce robust tumor-specific T-cell responses, reduce tumor markers and resolve metastatic lesions (37). Moreover, combination chemoimmunotherapy has yielded satisfactory outcomes, such as radiological CR and sustained long-term survival beyond three years after treatment discontinuation in individual cases (38). Collectively, these findings underscore that combinatorial immunotherapy strategies can yield significant clinical responses in non-dMMR/MSI-H pancreatic cancer, thereby reinforcing the importance of biomarker-driven patient selection and the integration of multimodal treatment.

This case represents the first documented patient with LAPC of the pMMR/high CPS molecular subtype achieving a CR through non-surgical therapy. This outcome challenges the common belief that pMMR tumors are unresponsive to ICIs and underscores the potential predictive value of high PD-L1 expression (CPS) within this group.

Furthermore, the multimodal BRICS regimen—integrating SBRT with low-dose chemotherapy, anti-angiogenic agents, ICIs, and microbiome modulation—proposes a potentially testable framework for treating pMMR pancreatic cancer. Notably, we acknowledge that as a single-case report, these findings should be interpreted with caution and cannot be broadly generalized. Further prospective clinical trials are essential to determine whether the pMMR/high CPS subtype identifies a responsive subgroup and to validate the safety and efficacy of this combination strategy in a larger LAPC population.

The patient reported significant satisfaction with the remarkable therapeutic efficacy and the well-tolerated nature of the treatment, which was free from significant adverse events. She has given her full consent for the sharing of her clinical data to aid future research and clinical advancements.

## 4 Conclusion

This case report presents, for the first time, a patient with LAPC of the pMMR/high CPS subtype who achieved a complete response (CR) through non-surgical management. We demonstrate that a treatment-naïve patient with pMMR/high CPS LAPC achieved a sustained CR following treatment with a novel regimen comprising SBRT, nab-paclitaxel, anlotinib, toripalimab, and triple Bifidobacterium tablets. This finding suggests that pMMR pancreatic cancer should not be automatically categorized as an “immune-cold” tumor unfit for immunotherapy in the context of precision medicine. Instead, a comprehensive molecular subtyping, including PD-L1 evaluation, along with synergistic multimodal strategies tailored to the tumor microenvironment, may open new avenues for therapeutic options in this particularly challenging disease.

## Data Availability

The original contributions presented in the study are included in the article/**Supplementary Material**. Further inquiries can be directed to the corresponding authors.

## Supplementary material

Supplementary Figure 1 Baseline contrast-enhanced CT of the tumor lesion.

Supplementary Figure 2 Pathological images of routine histology and immunohistochemical markers.

Supplementary Figure 3 GTV delineation. (The GTV is delineated by the red line).

Supplementary Figure 4 Contrast-enhanced CT image of the tumor lesion (March 26, 2025).

Supplementary Figure 5 Contrast-enhanced CT image of the tumor lesion (August 19, 2025).

Supplementary Table 1 Immunohistochemistry Markers.
