# Supplementary material for: Complete response to BRICS in Locally advanced pancreatic cancer (pMMR, CPS 30): a case report
Source: Front Immunol. 2026 Jan 21;17:1743752. doi: 10.3389/fimmu.2026.1743752 (PMC12867830; doi:10.3389/fimmu.2026.1743752)
Supplement: Supplementary Figure 1 — Baseline contrast-enhanced CT of the tumor lesion. [file DataSheet1.pdf]

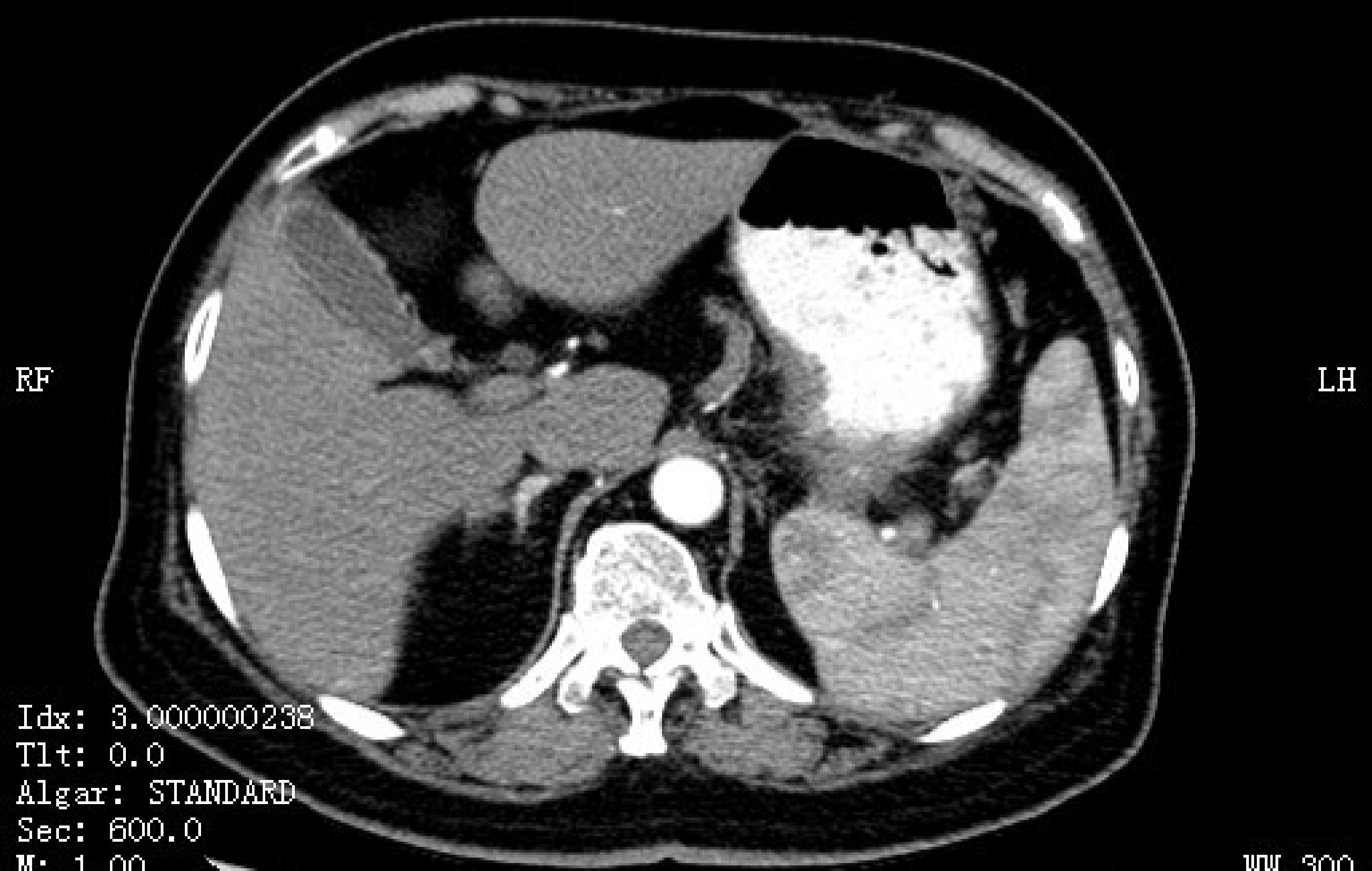
RF
LH
Idx: 3.000000238
Tlt: 0.0
Algar: STANDARD
Sec: 600.0

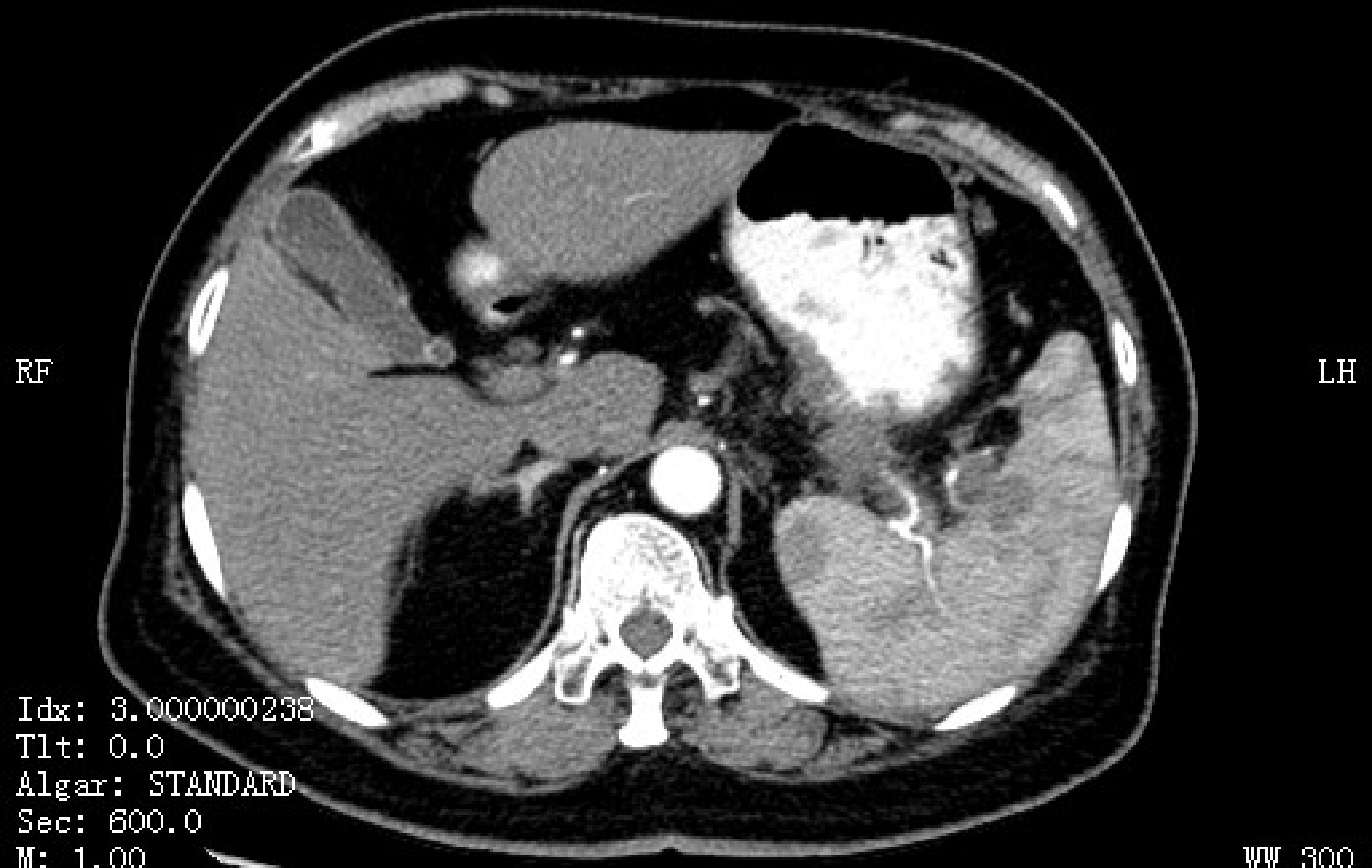
RF
LH
Idx: 3.000000238
Tlt: 0.0
Algar: STANDARD
Sec: 600.0
M: 1.00

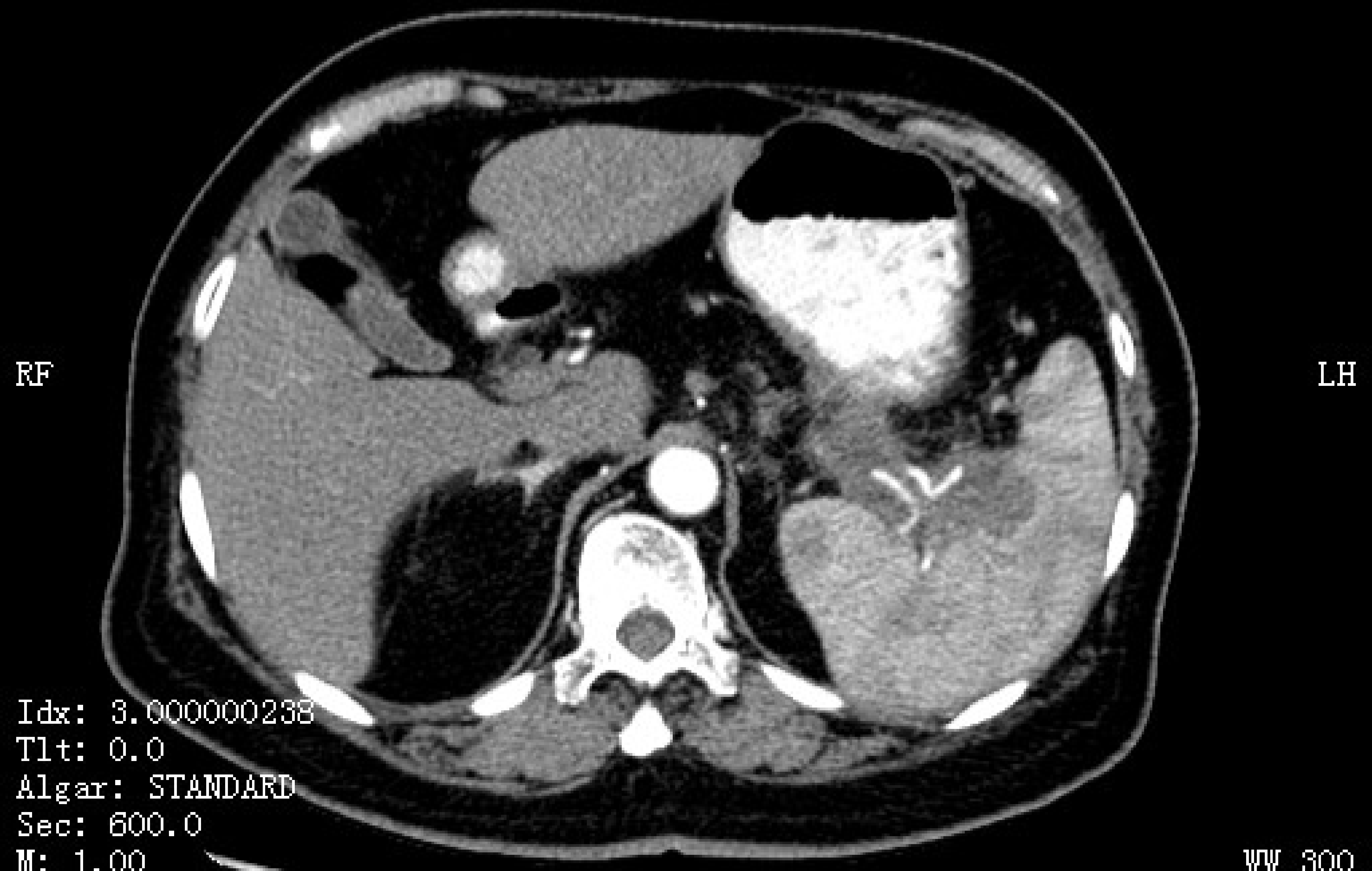
RF
LH
Idx: 3.000000238
Tlt: 0.0
Algar: STANDARD
Sec: 600.0
M: 1.00
WW 300

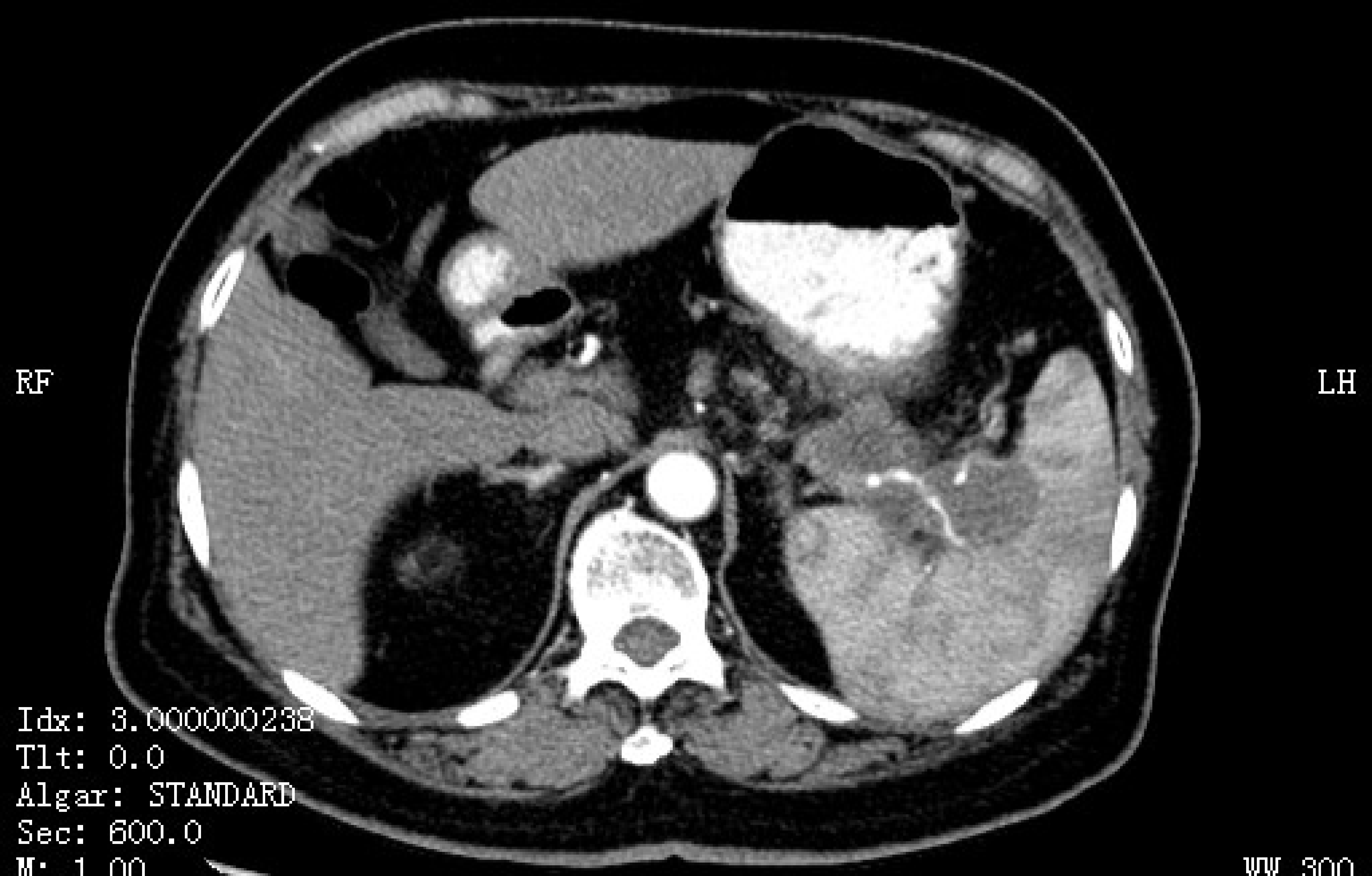
RF
LH
Idx: 3.000000238
Tlt: 0.0
Algar: STANDARD
Sec: 600.0

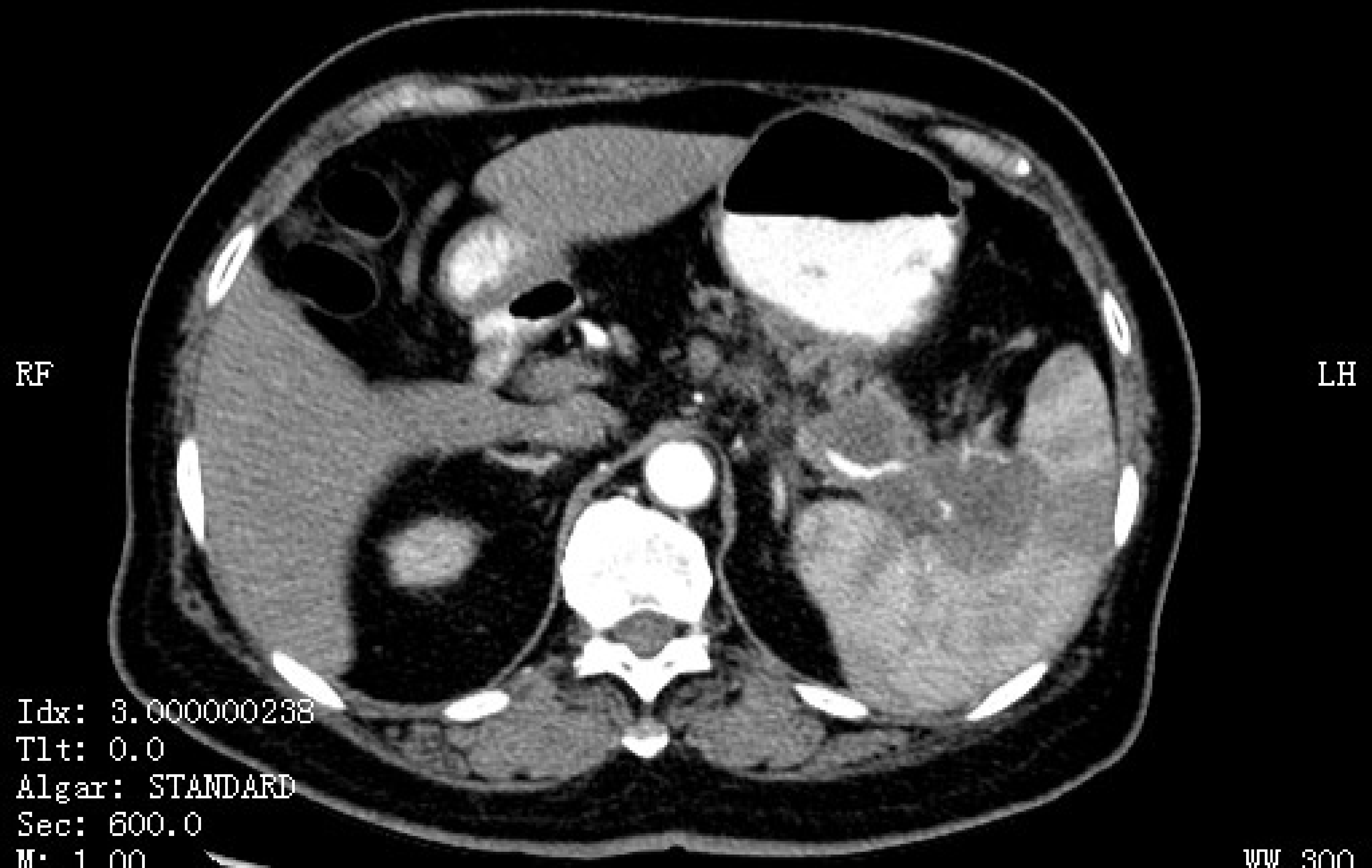
RF
LH
Idx: 3.000000238
Tlt: 0.0
Algar: STANDARD
Sec: 600.0

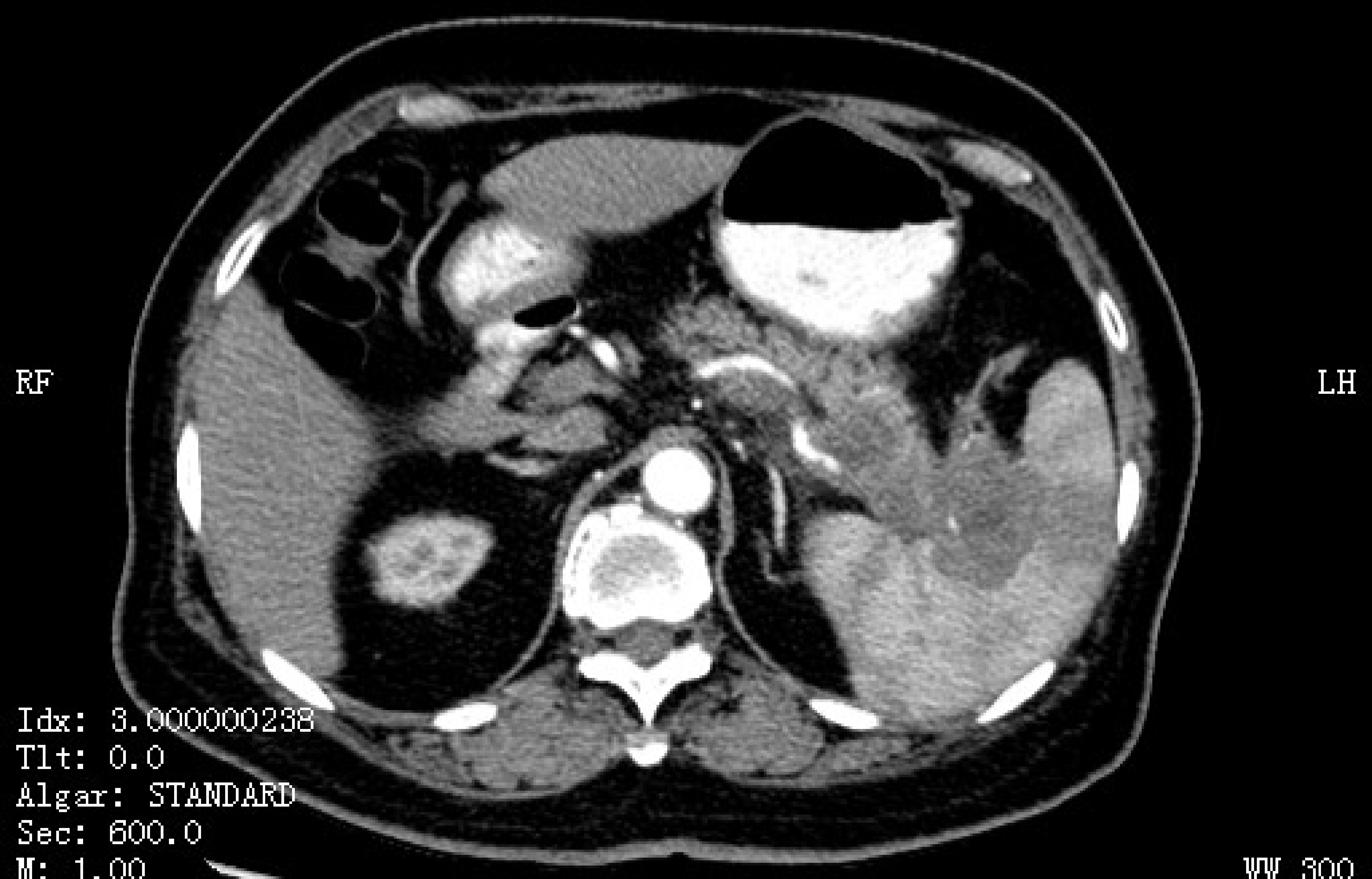
RF
LH
Idx: 3.000000238
Tlt: 0.0
Algar: STANDARD
Sec: 600.0
M: 1.00
WW 300

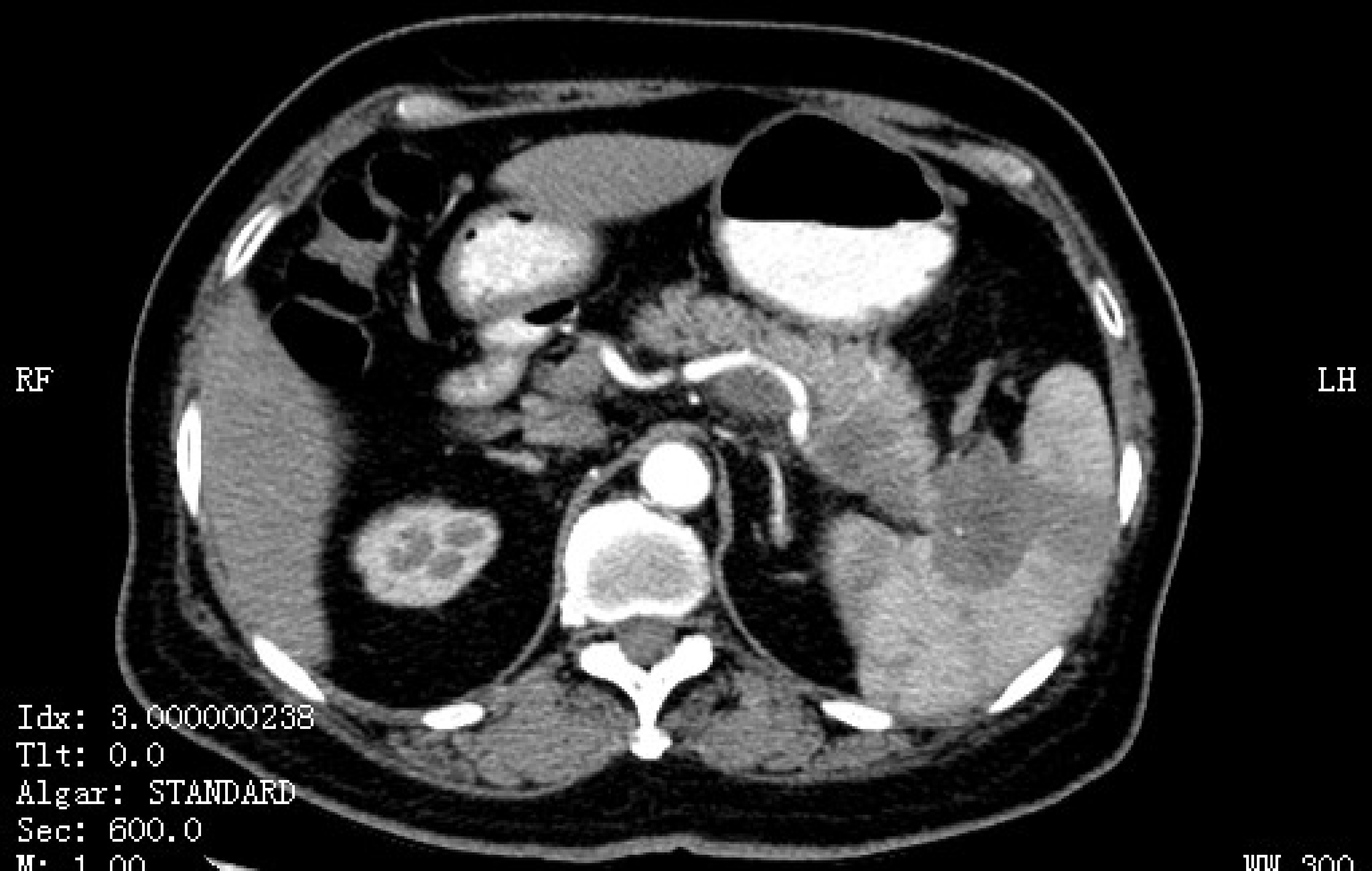
RF
LH
Idx: 3.000000238
Tlt: 0.0
Algar: STANDARD
Sec: 600.0

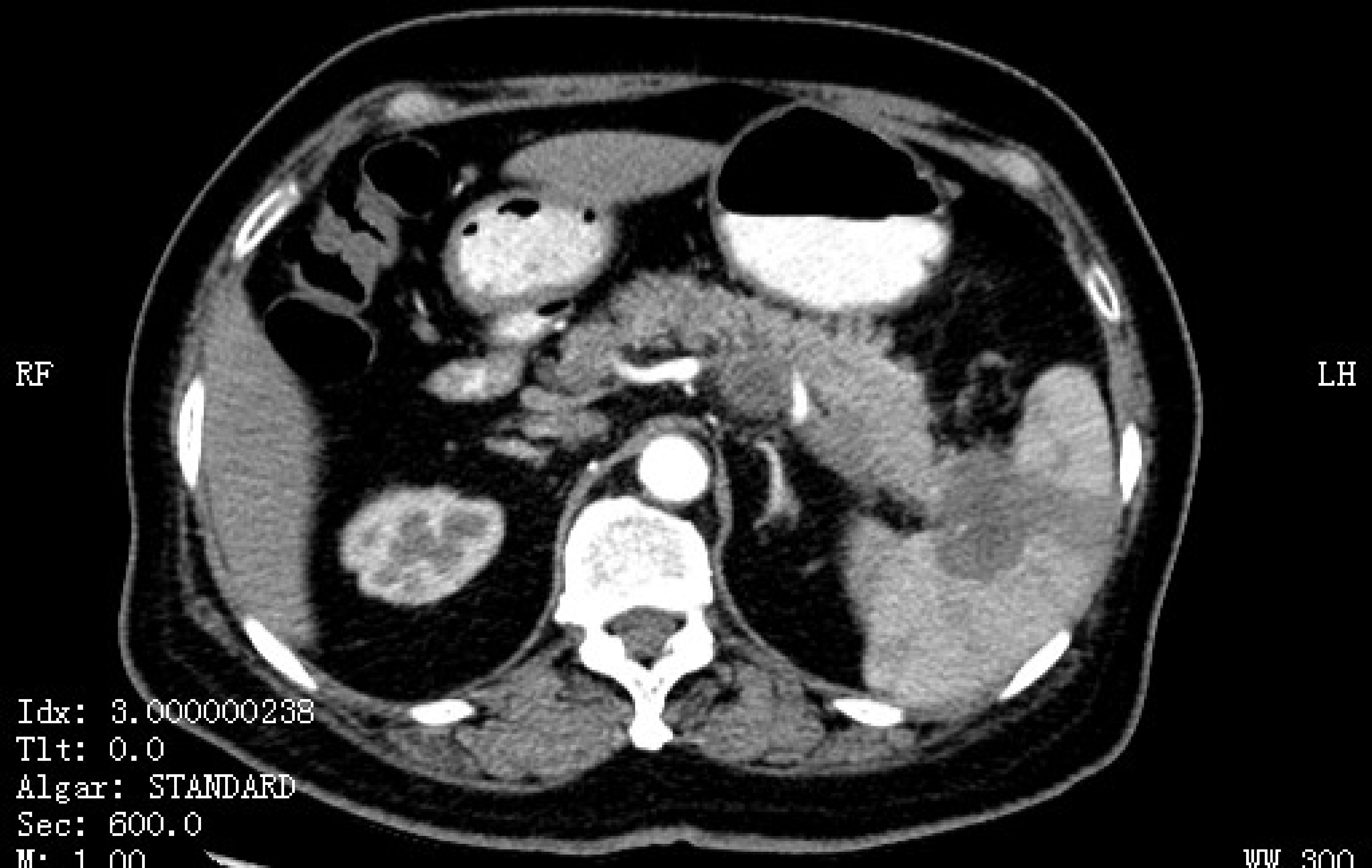
RF
LH
Idx: 3.000000238
Tlt: 0.0
Algar: STANDARD
Sec: 600.0
M: 1.00
WW 300

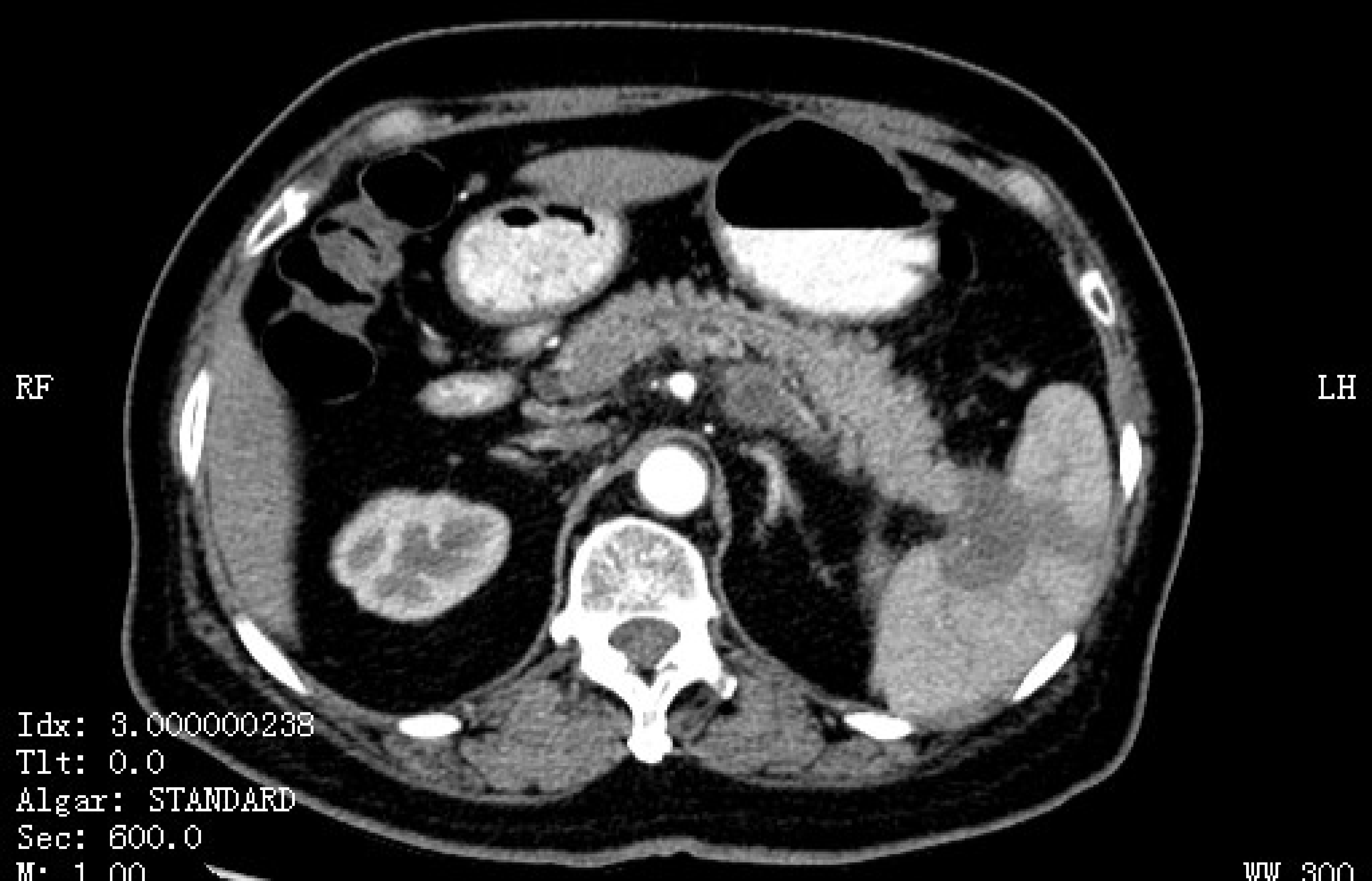
RF
LH
Idx: 3.000000238
Tlt: 0.0
Algar: STANDARD
Sec: 600.0
M: 1.00
WW 300

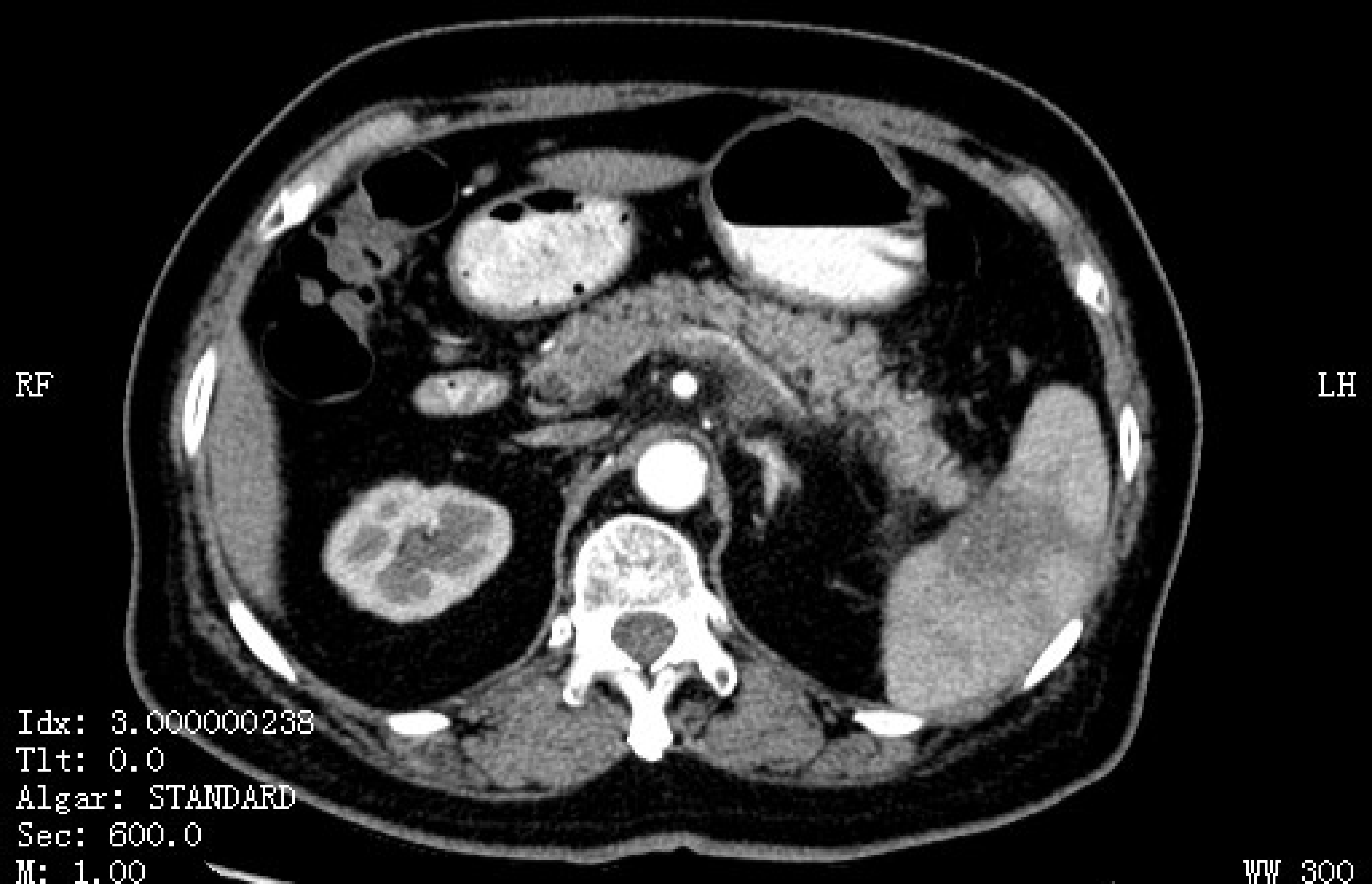
RF
LH
Idx: 3.000000238
Tlt: 0.0
Algar: STANDARD
Sec: 600.0
M: 1.00
WW 300

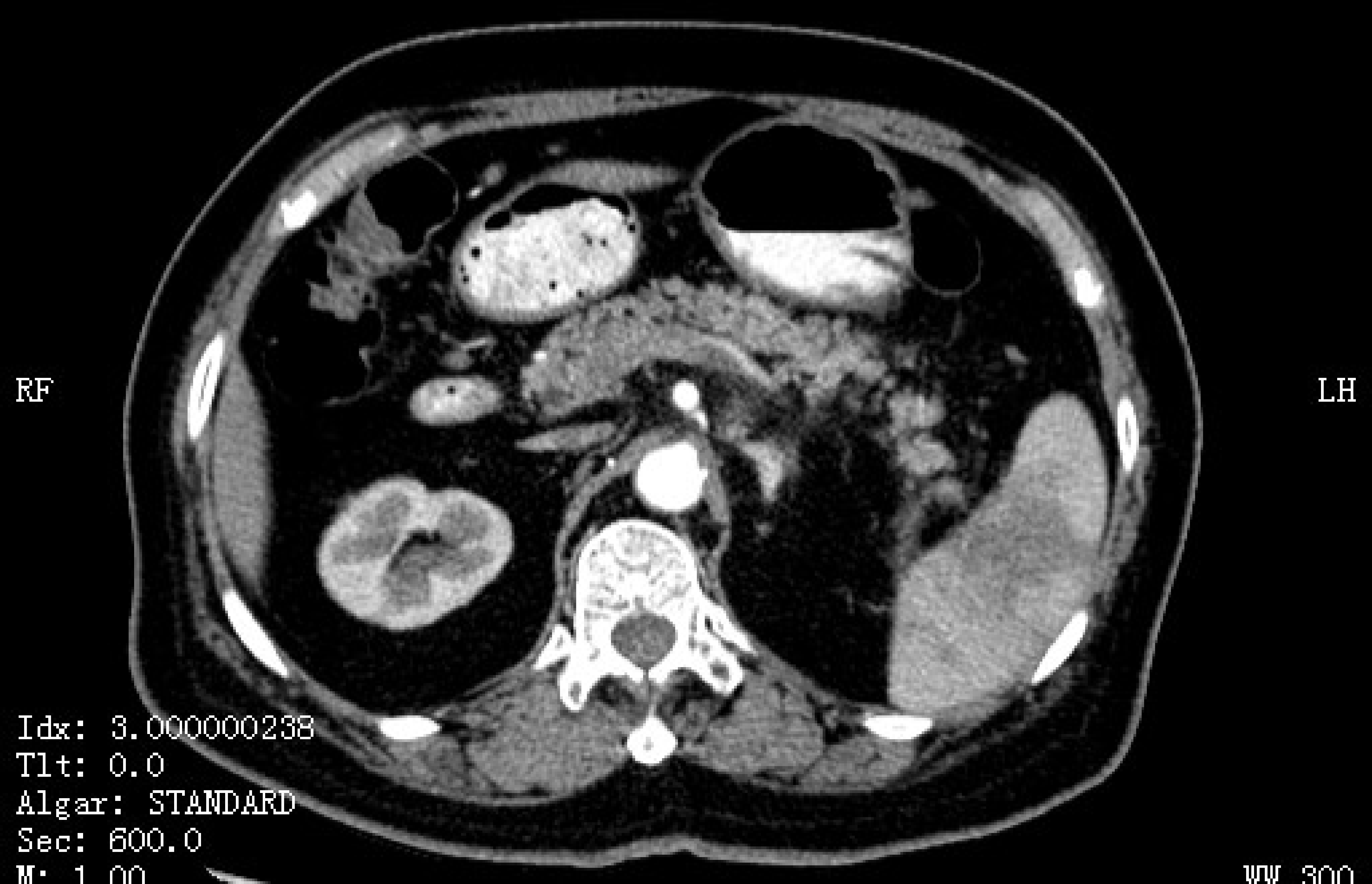
RF
LH
Idx: 3.000000238
Tlt: 0.0
Algar: STANDARD
Sec: 600.0
M: 1.00
WW 300

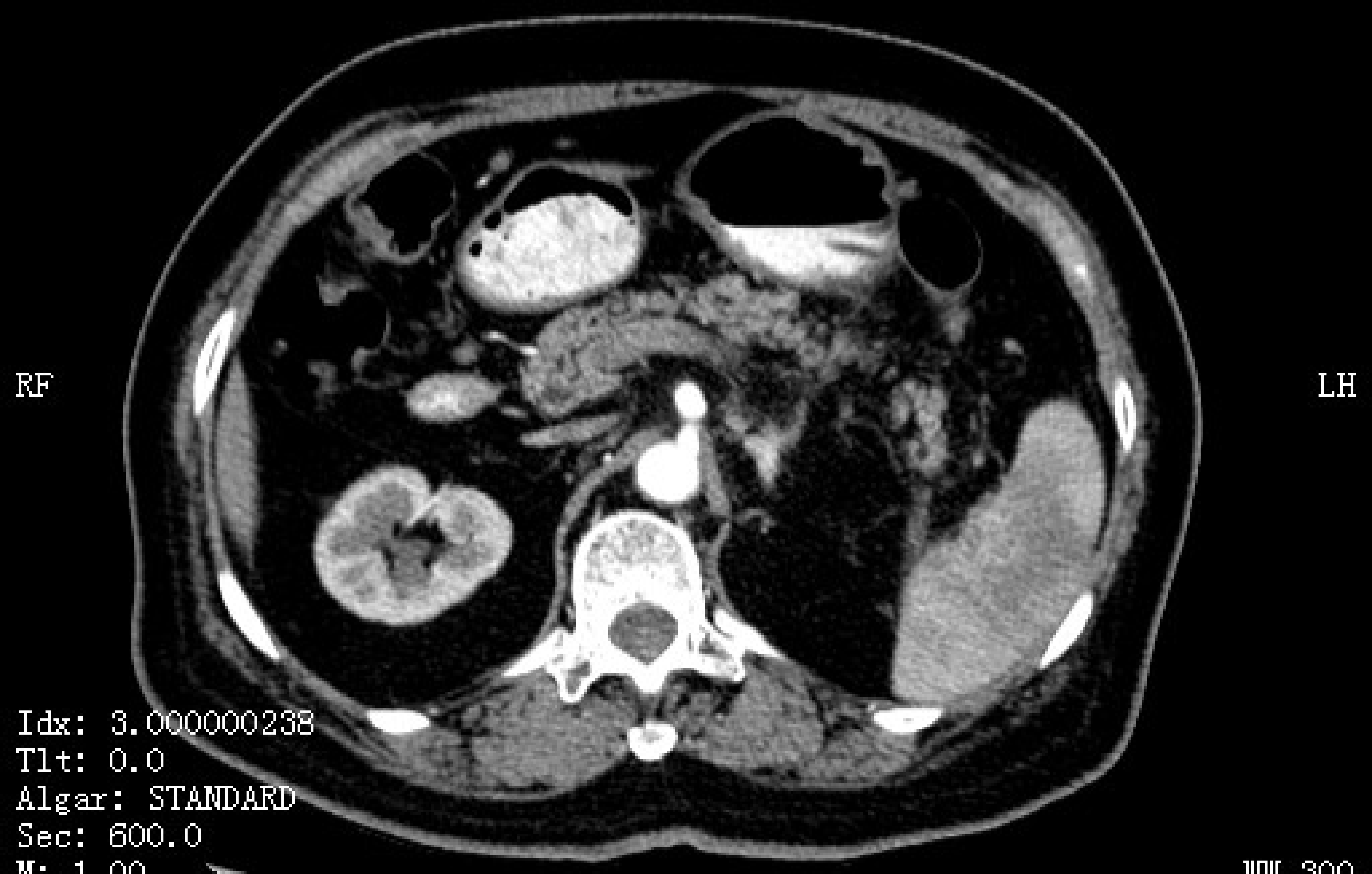
RF
LH
Idx: 3.000000238
Tlt: 0.0
Algar: STANDARD
Sec: 600.0

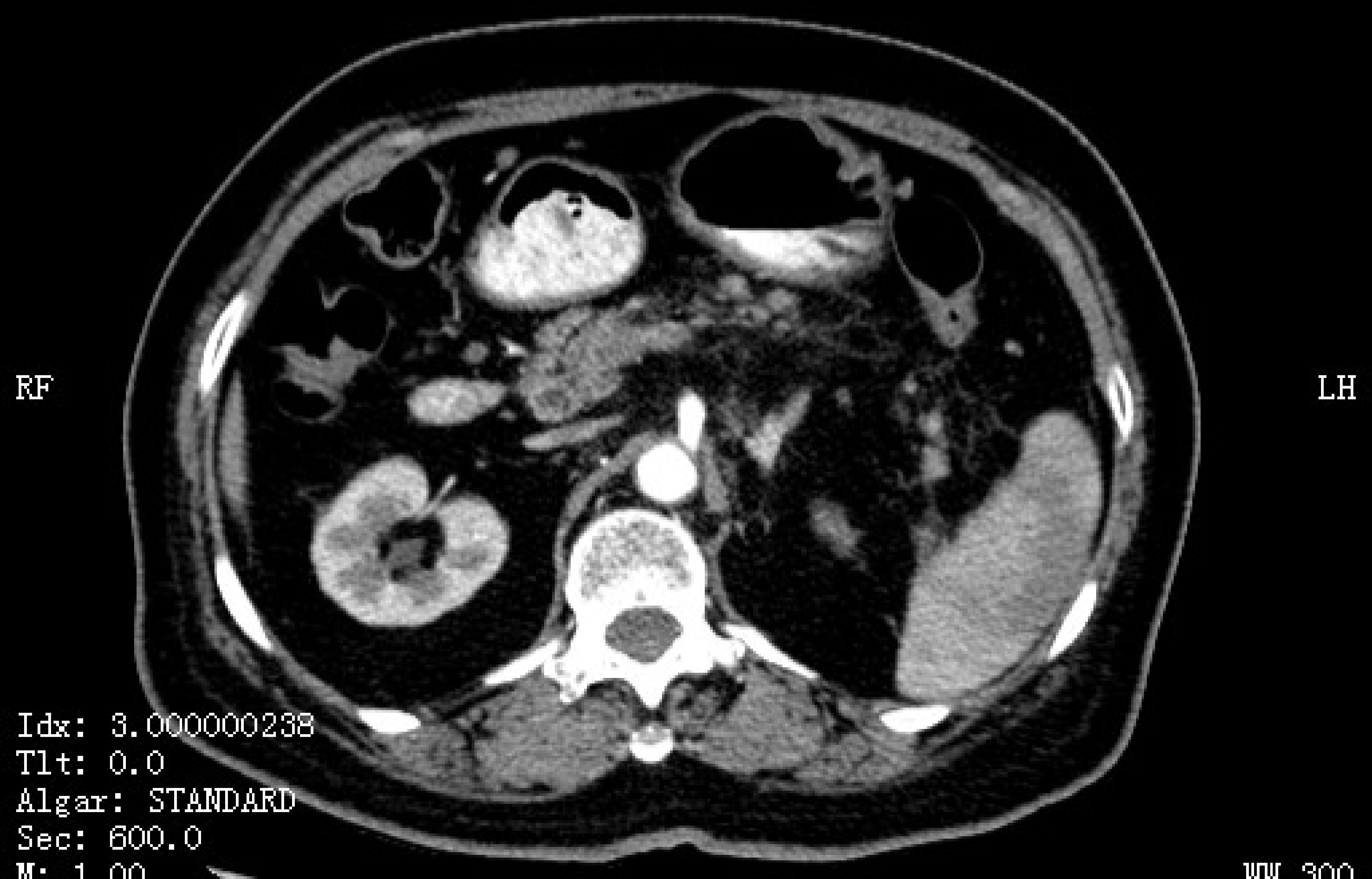
RF
LH
Idx: 3.000000238
Tlt: 0.0
Algar: STANDARD
Sec: 600.0

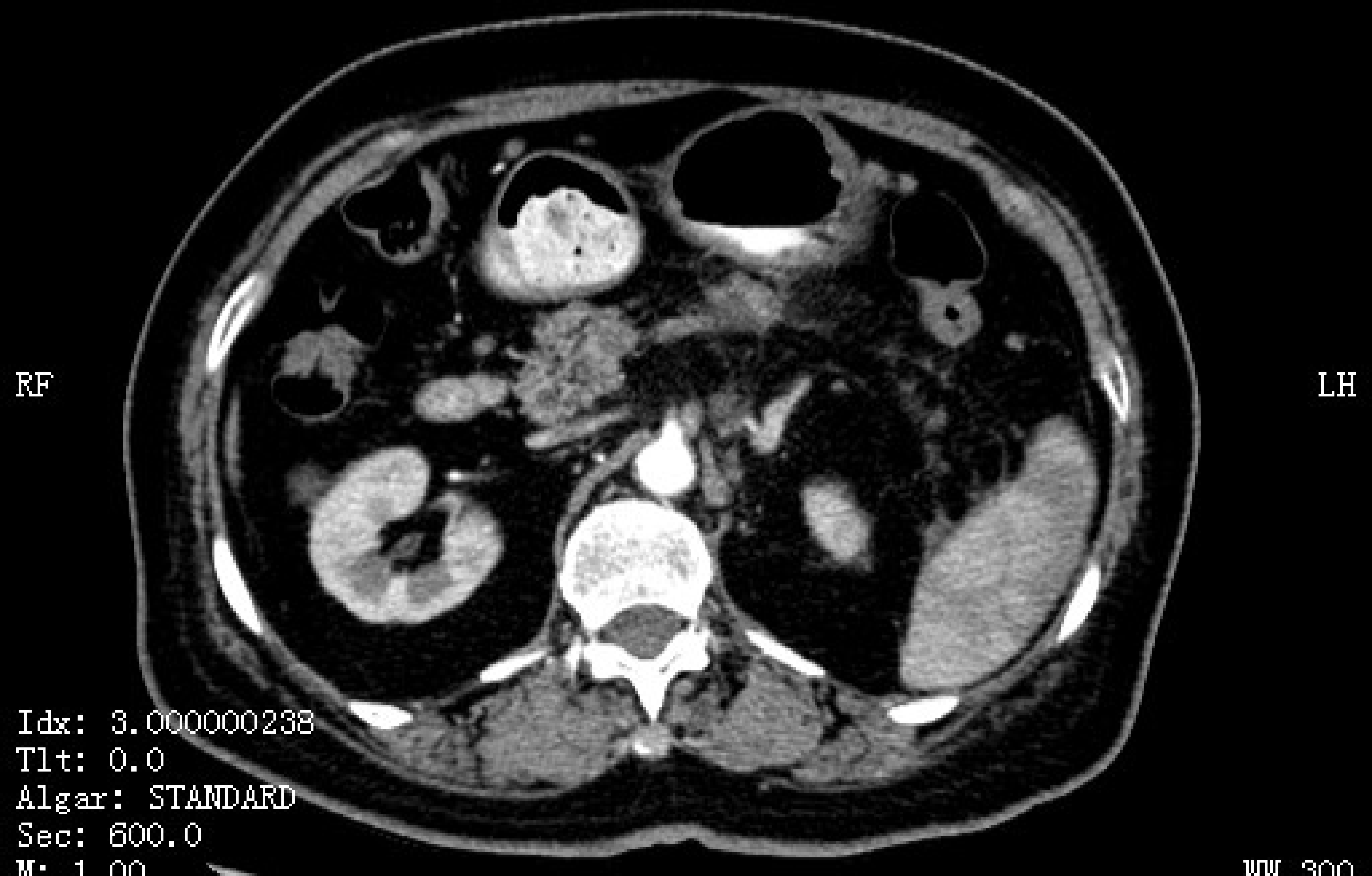
RF
LH
Idx: 3.000000238
Tlt: 0.0
Algar: STANDARD
Sec: 600.0

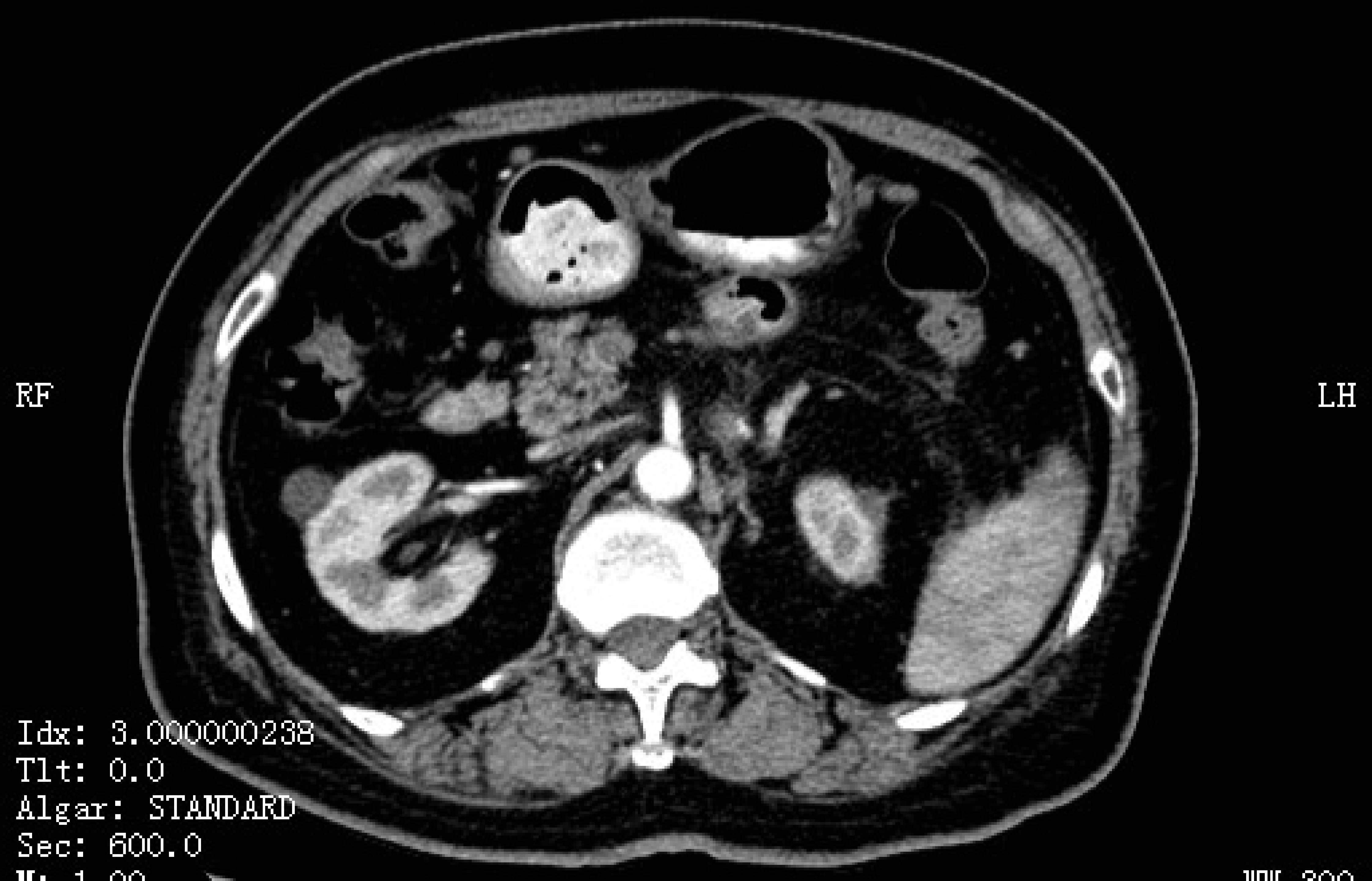
RF
LH
Idx: 3.000000238
Tlt: 0.0
Algar: STANDARD
Sec: 600.0

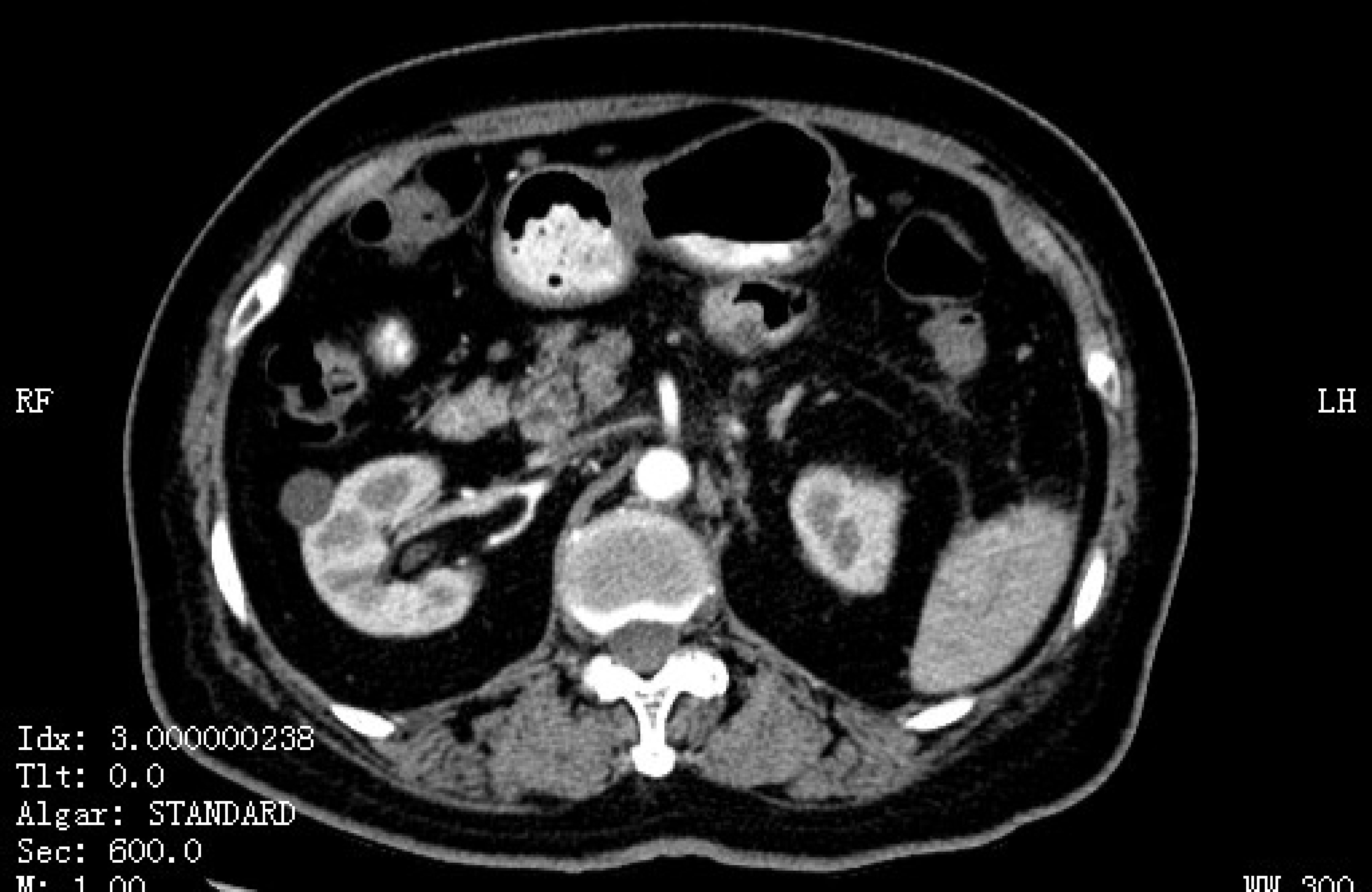
RF
LH
Idx: 3.000000238
Tlt: 0.0
Algar: STANDARD
Sec: 600.0

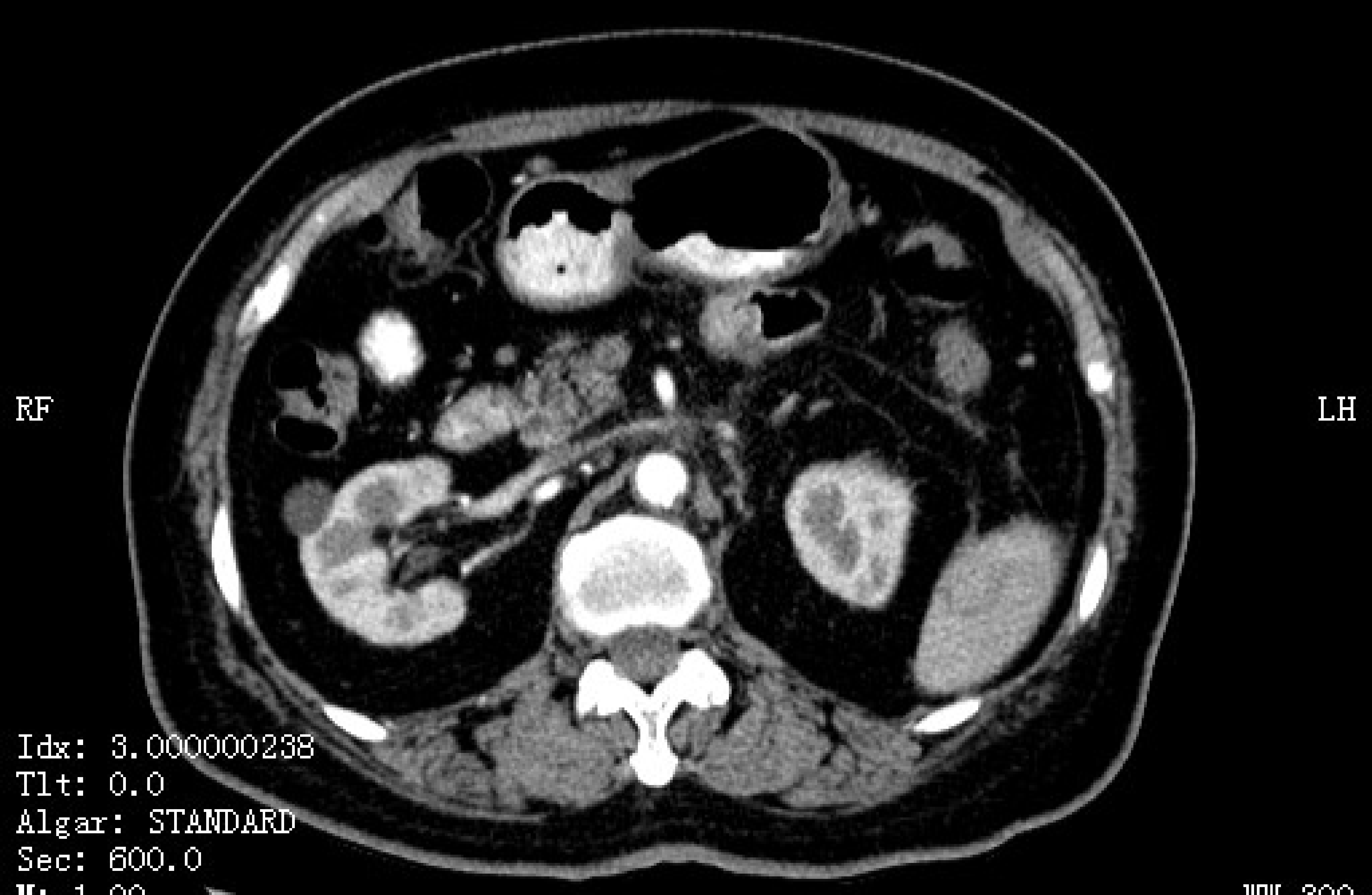
RF
LH
Idx: 3.000000238
Tlt: 0.0
Algar: STANDARD
Sec: 600.0

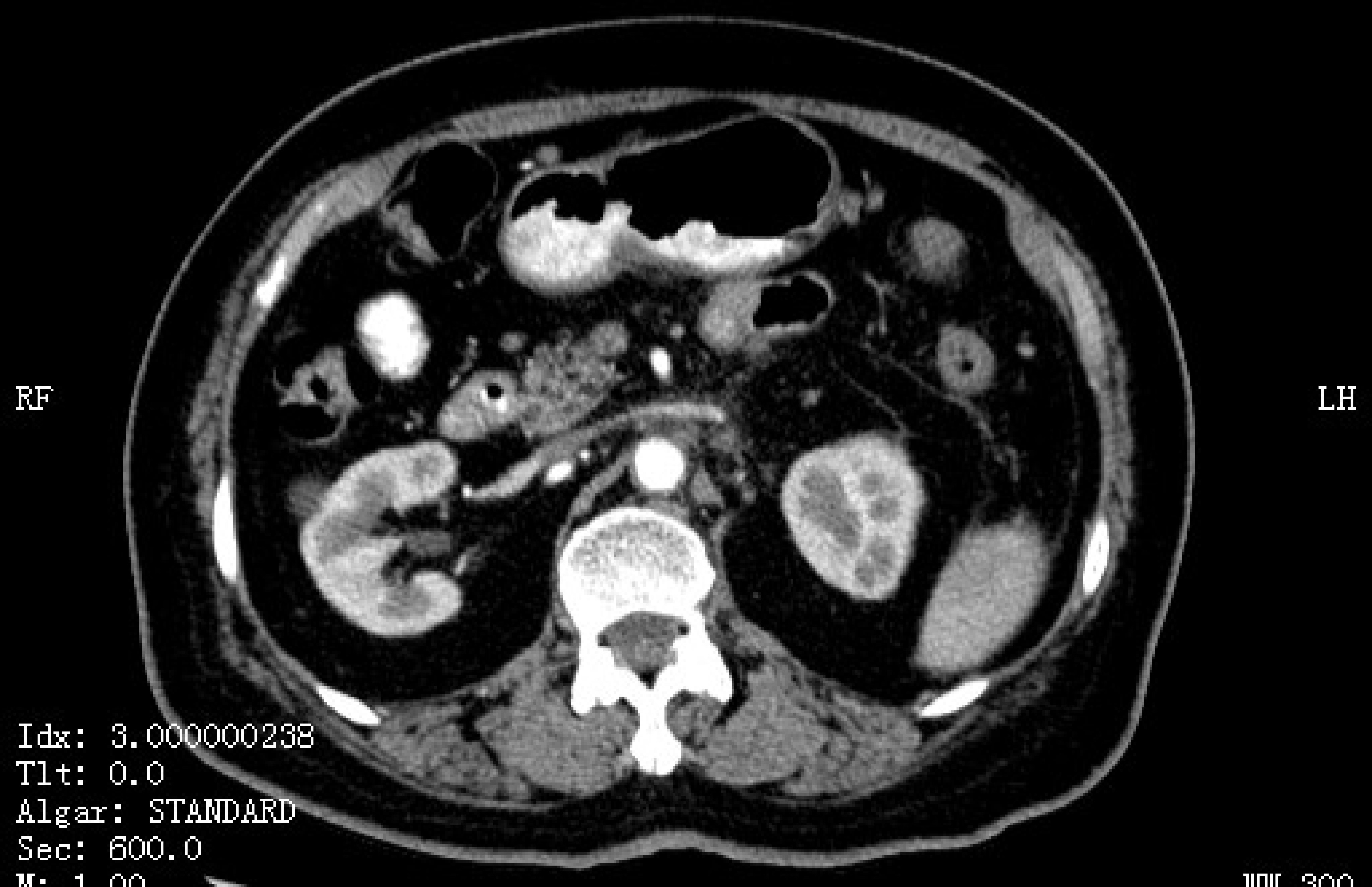
RF
LH
Idx: 3.000000238
Tlt: 0.0
Algar: STANDARD
Sec: 600.0

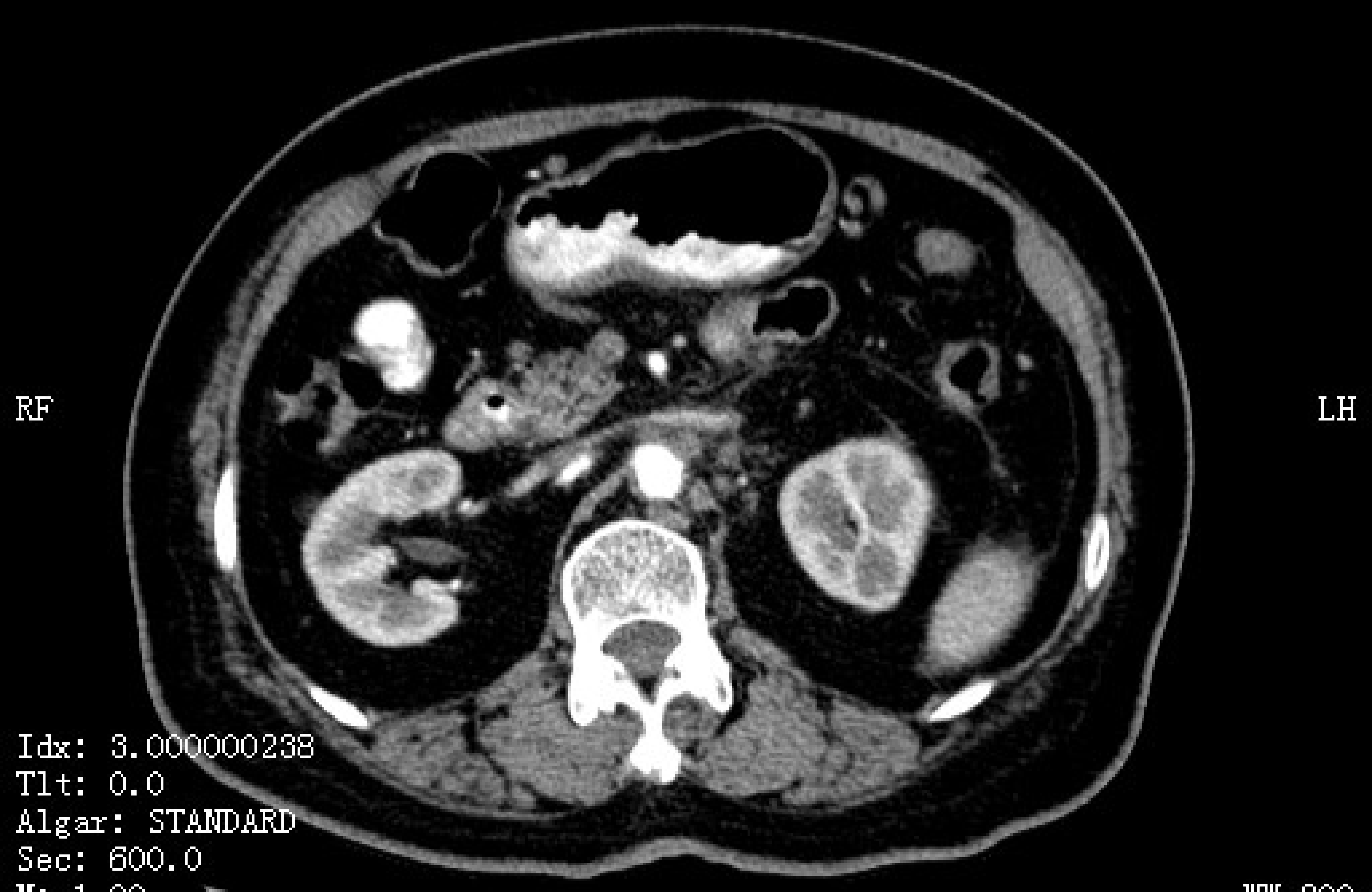
RF
LH
Idx: 3.000000238
Tlt: 0.0
Algar: STANDARD
Sec: 600.0

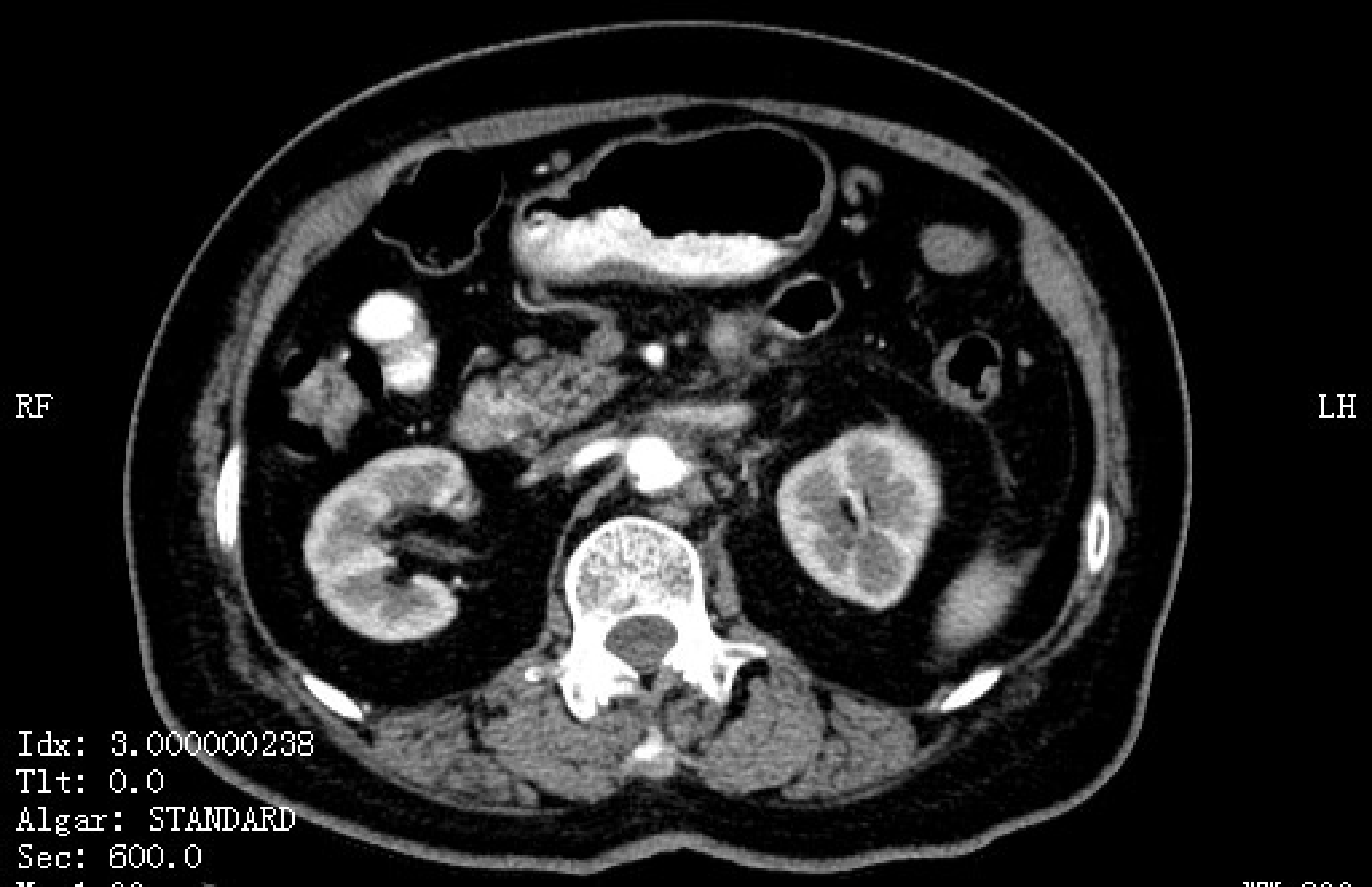
RF
LH
Idx: 3.000000238
Tlt: 0.0
Algar: STANDARD
Sec: 600.0

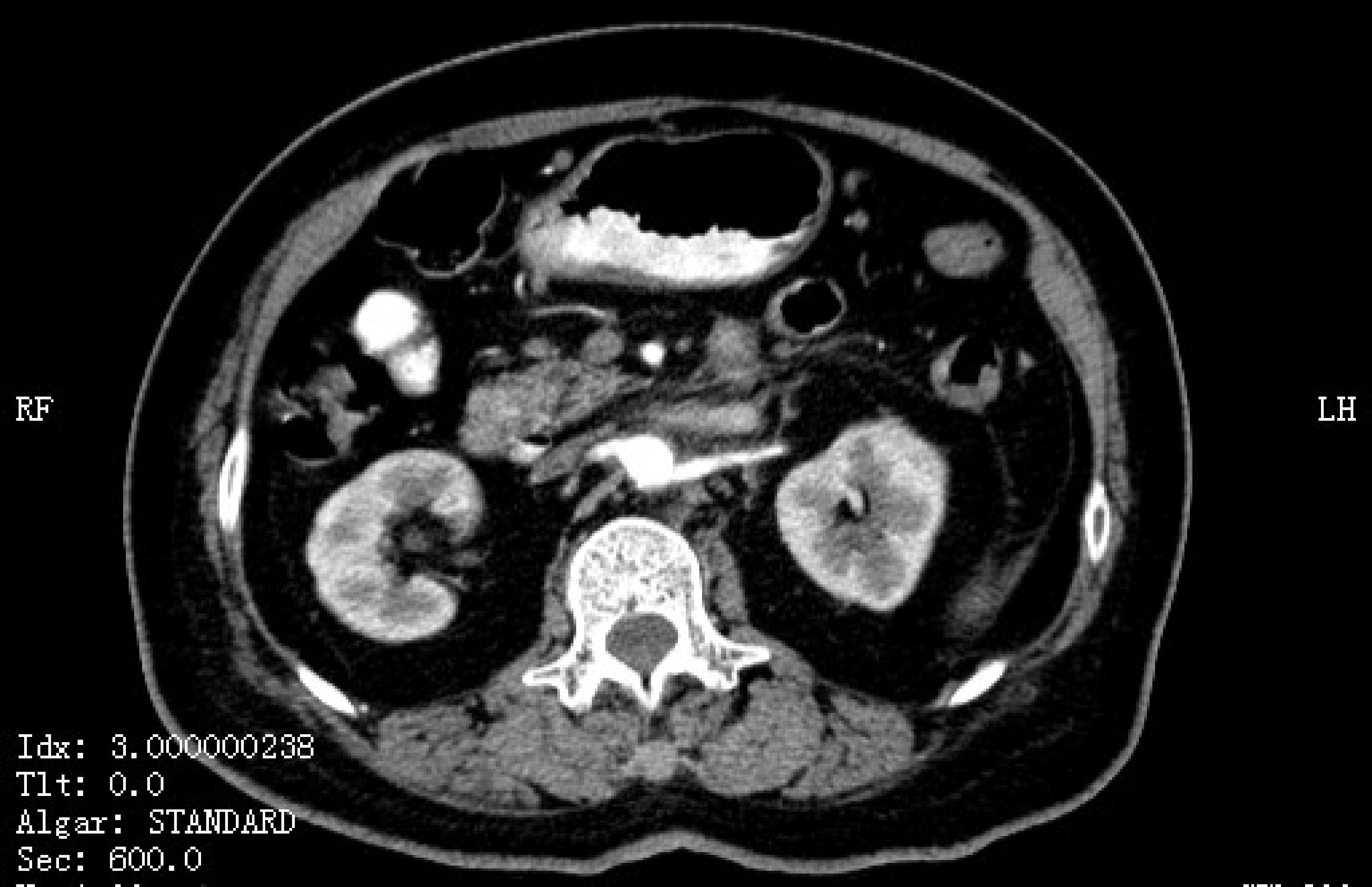
RF
LH
Idx: 3.000000238
Tlt: 0.0
Algar: STANDARD
Sec: 600.0

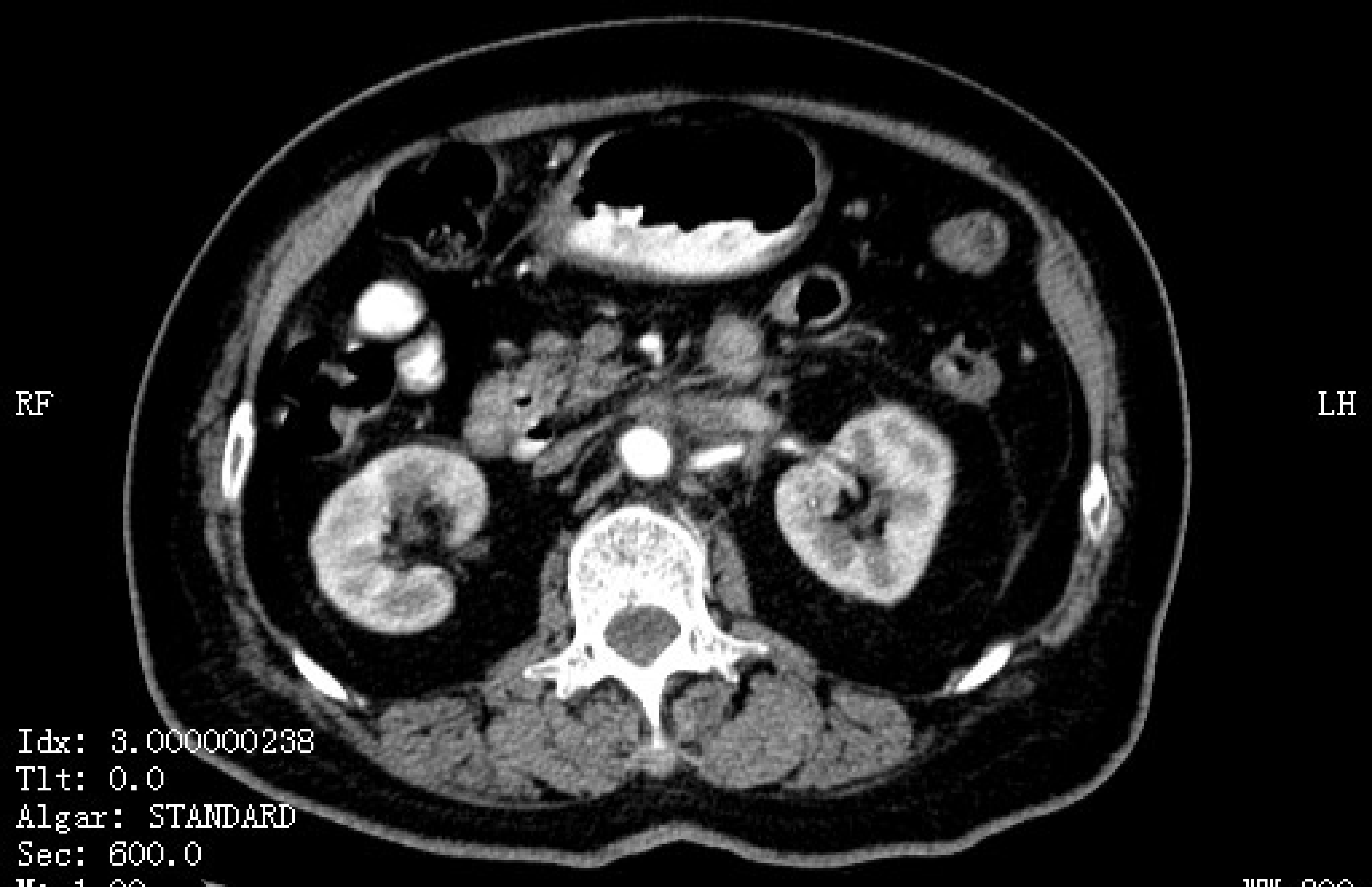
RF
LH
Idx: 3.000000238
Tlt: 0.0
Algar: STANDARD
Sec: 600.0

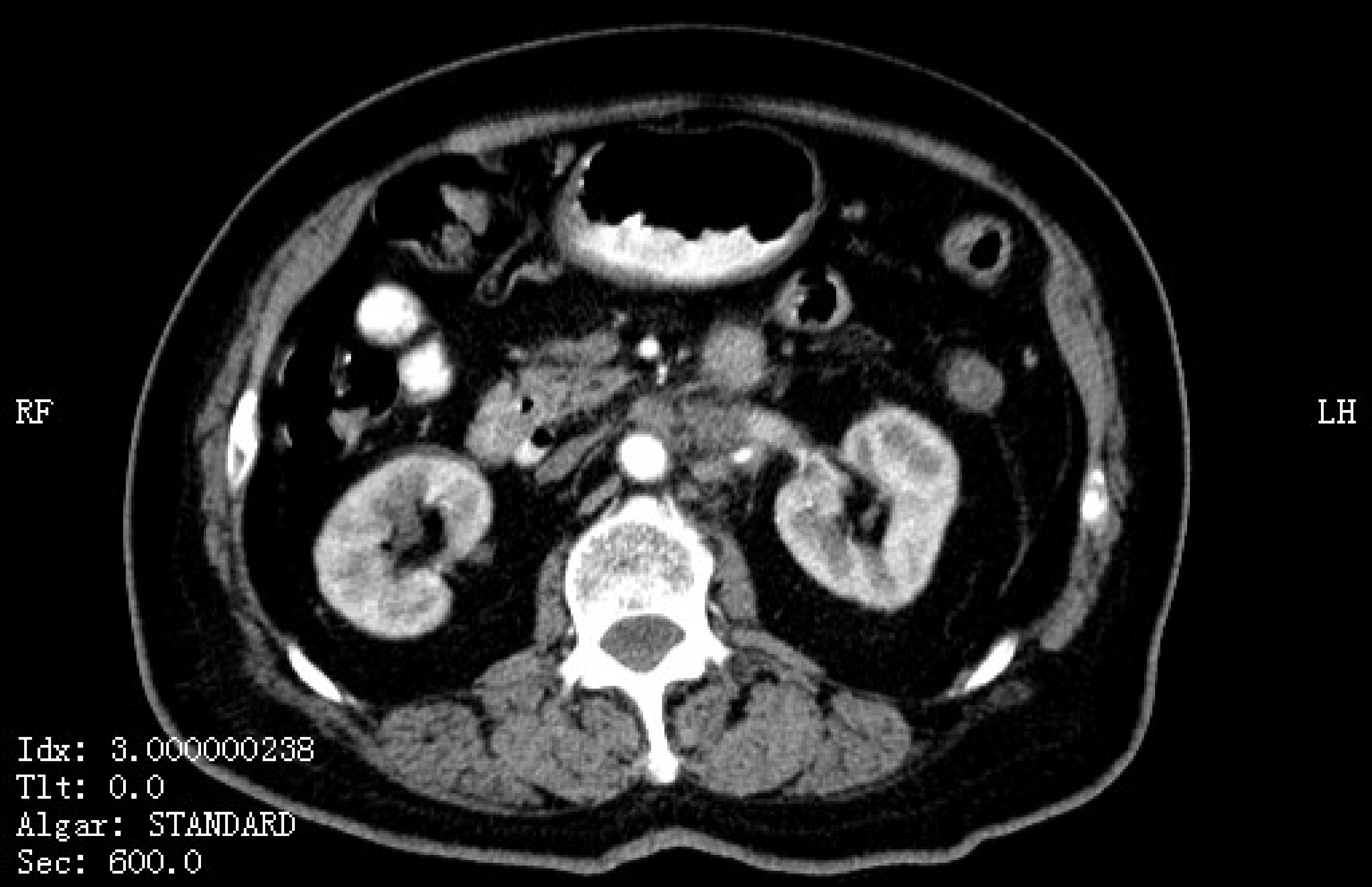
RF
LH
Idx: 3.000000238
Tlt: 0.0
Algar: STANDARD
Sec: 600.0

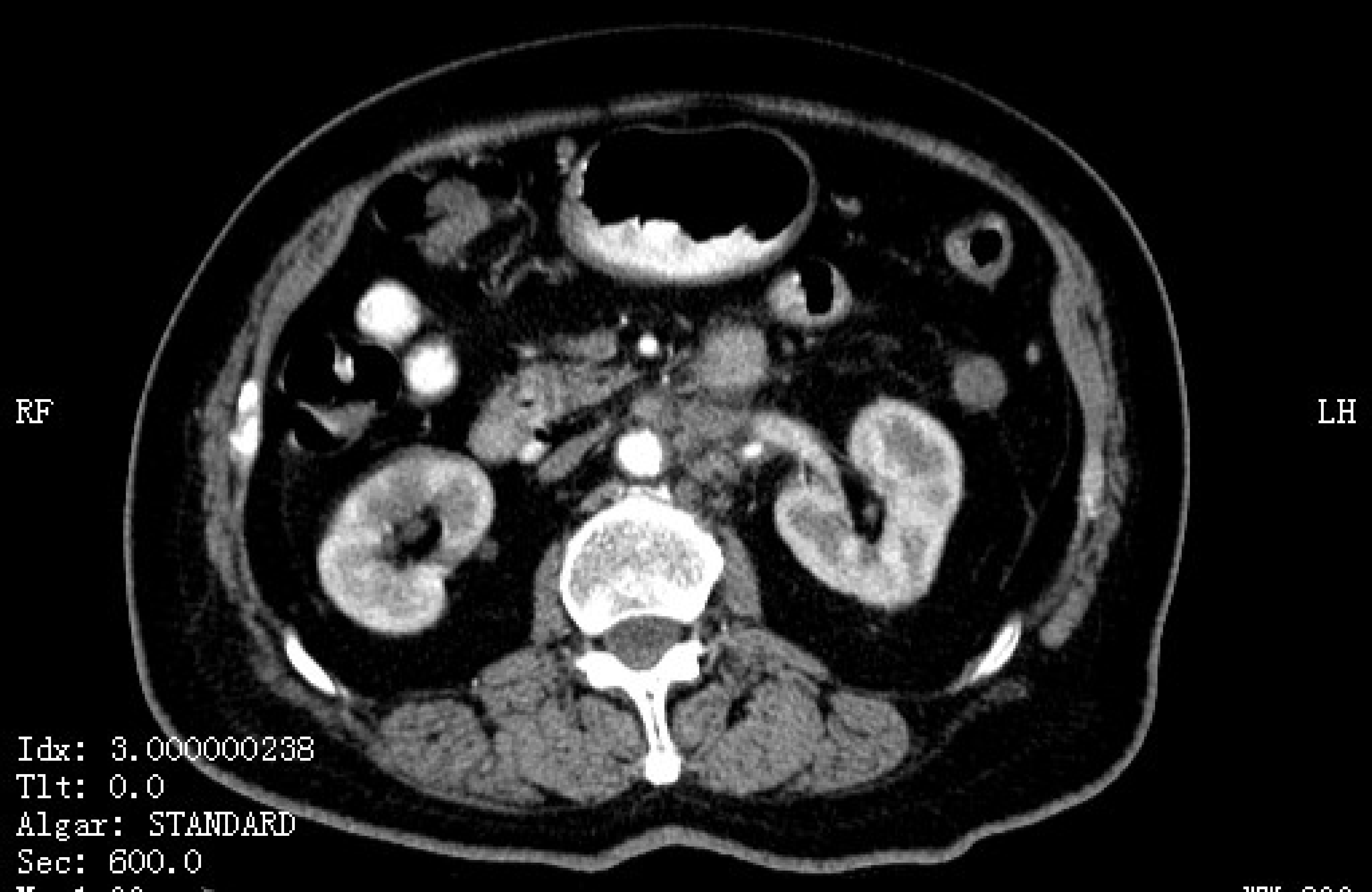
RF
LH
Idx: 3.000000238
Tlt: 0.0
Algar: STANDARD
Sec: 600.0
