# Supplementary material for: Complete response to BRICS in Locally advanced pancreatic cancer (pMMR, CPS 30): a case report
Source: Front Immunol. 2026 Jan 21;17:1743752. doi: 10.3389/fimmu.2026.1743752 (PMC12867830; doi:10.3389/fimmu.2026.1743752)
Supplement: Supplementary Figure 2 — Pathological images of routine histology and immunohistochemical markers. [file DataSheet2.pdf]

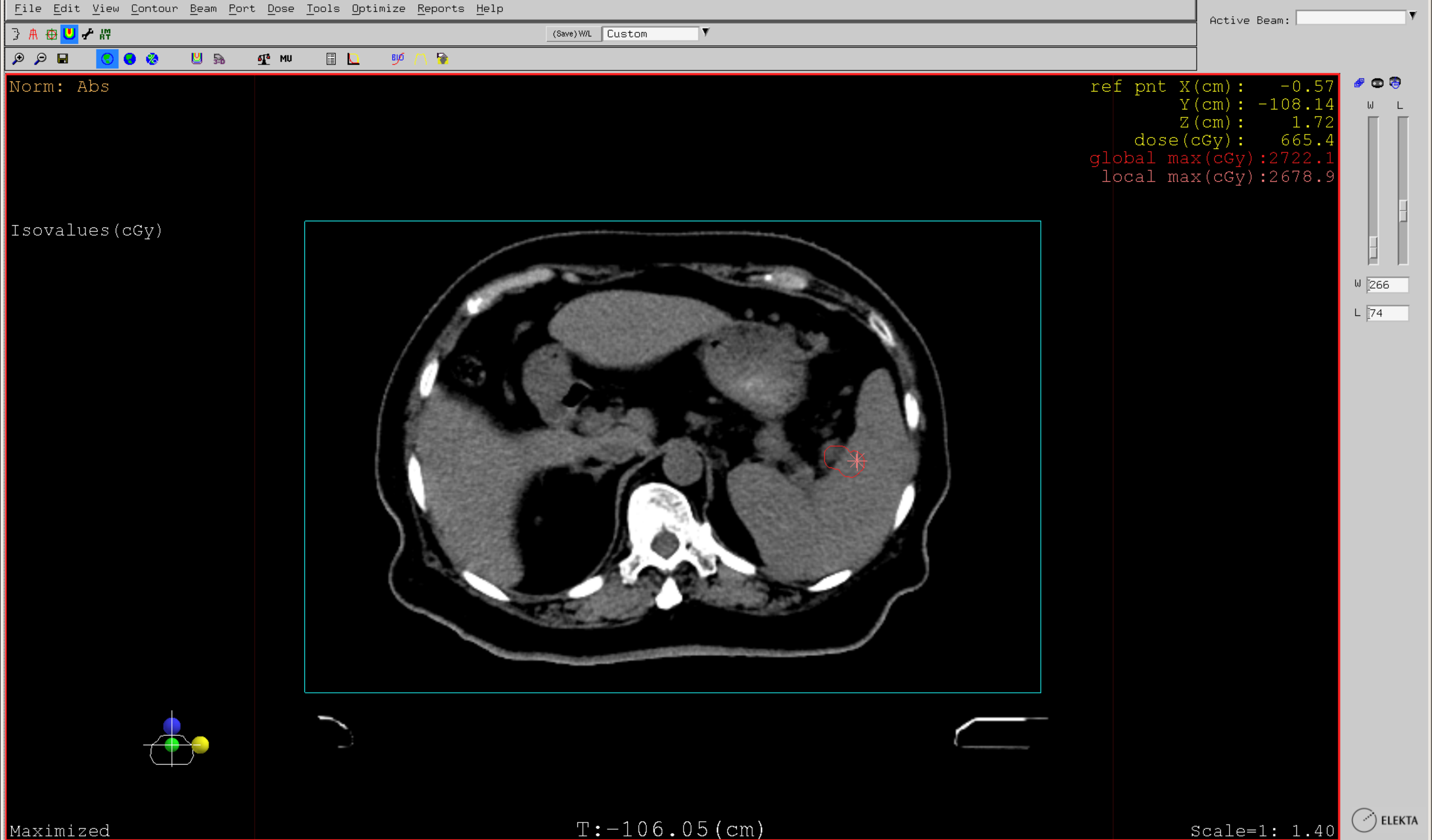
File Edit View Contour Beam Port Dose Tools Optimize Reports Help
Active Beam:
(Save) W/L
Custom
MU
Norm: Abs
ref pnt X(cm): -0.57
Y(cm): -108.14
Z(cm): 1.72
dose(cGy): 665.4
global max(cGy):2722.1
local max(cGy):2678.9
Isovalues(cGy)
W
L
W 266
L 74
Maximized
T:-106.05(cm)
Scale=1: 1.40
ELEKTA

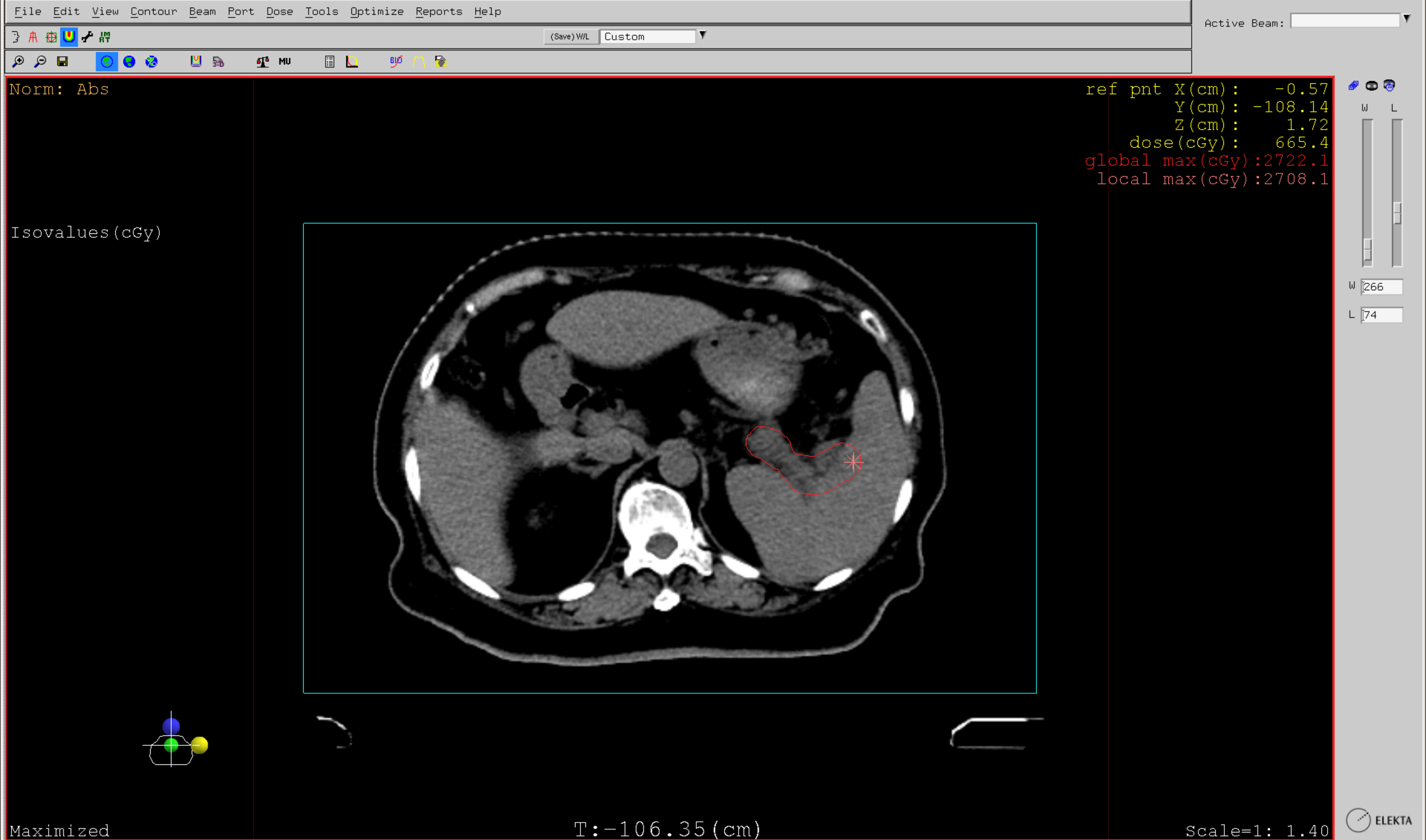
File Edit View Contour Beam Port Dose Tools Optimize Reports Help
(Save) W/L
Custom
Active Beam:
MU
Norm: Abs
ref pnt X(cm): -0.57
Y(cm): -108.14
Z(cm): 1.72
dose(cGy): 665.4
global max(cGy):2722.1
local max(cGy):2708.1
Isovalues(cGy)
W
L
W 266
L 74
Maximized
T:-106.35(cm)
Scale=1: 1.40
ELEKTA

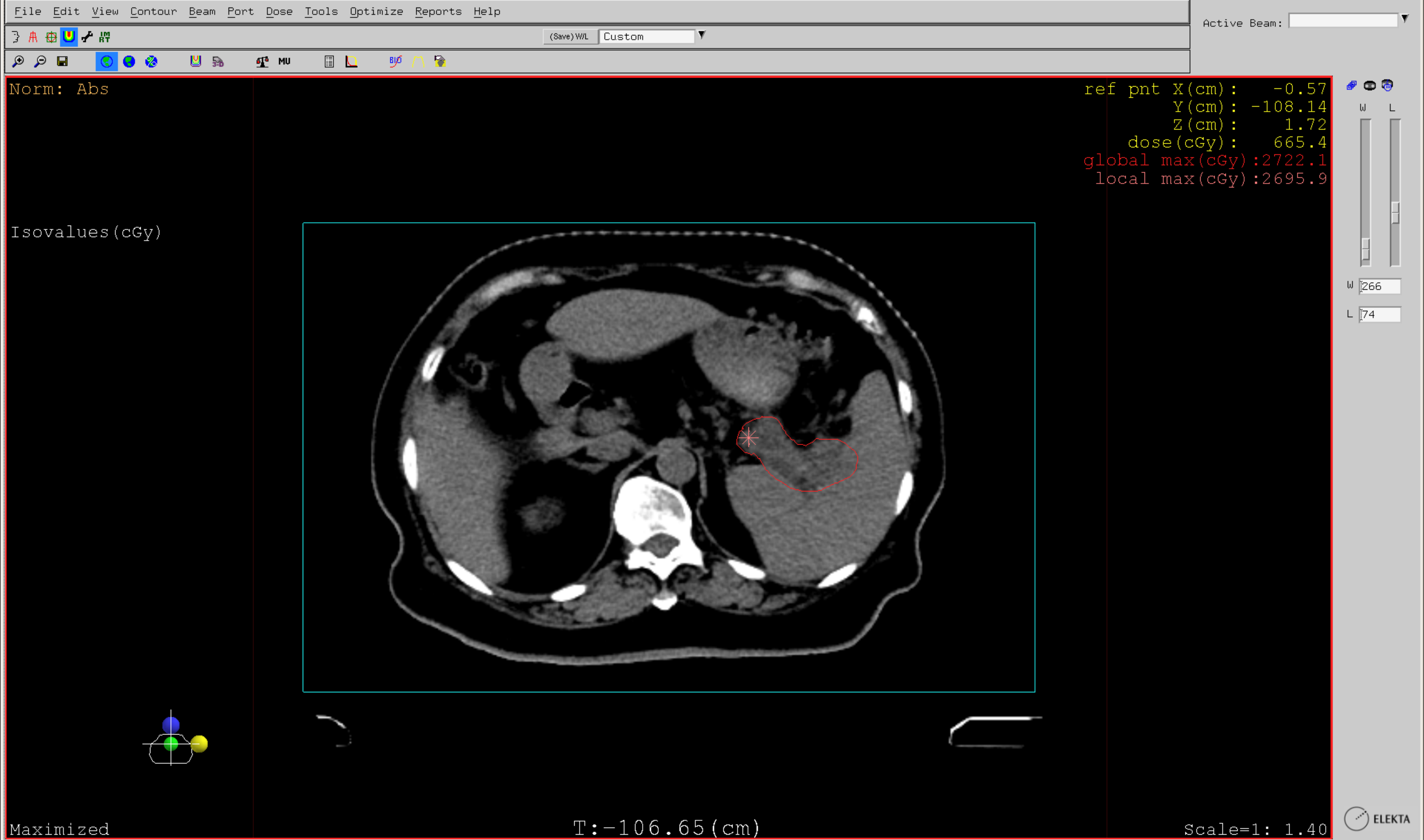
File Edit View Contour Beam Port Dose Tools Optimize Reports Help
Active Beam:
(Save) W/L
Custom
MU
Norm: Abs
ref pnt X(cm): -0.57
Y(cm): -108.14
Z(cm): 1.72
dose(cGy): 665.4
global max(cGy):2722.1
local max(cGy):2695.9
Isovalues(cGy)
W
L
W 266
L 74
Maximized
T:-106.65(cm)
Scale=1: 1.40
ELEKTA

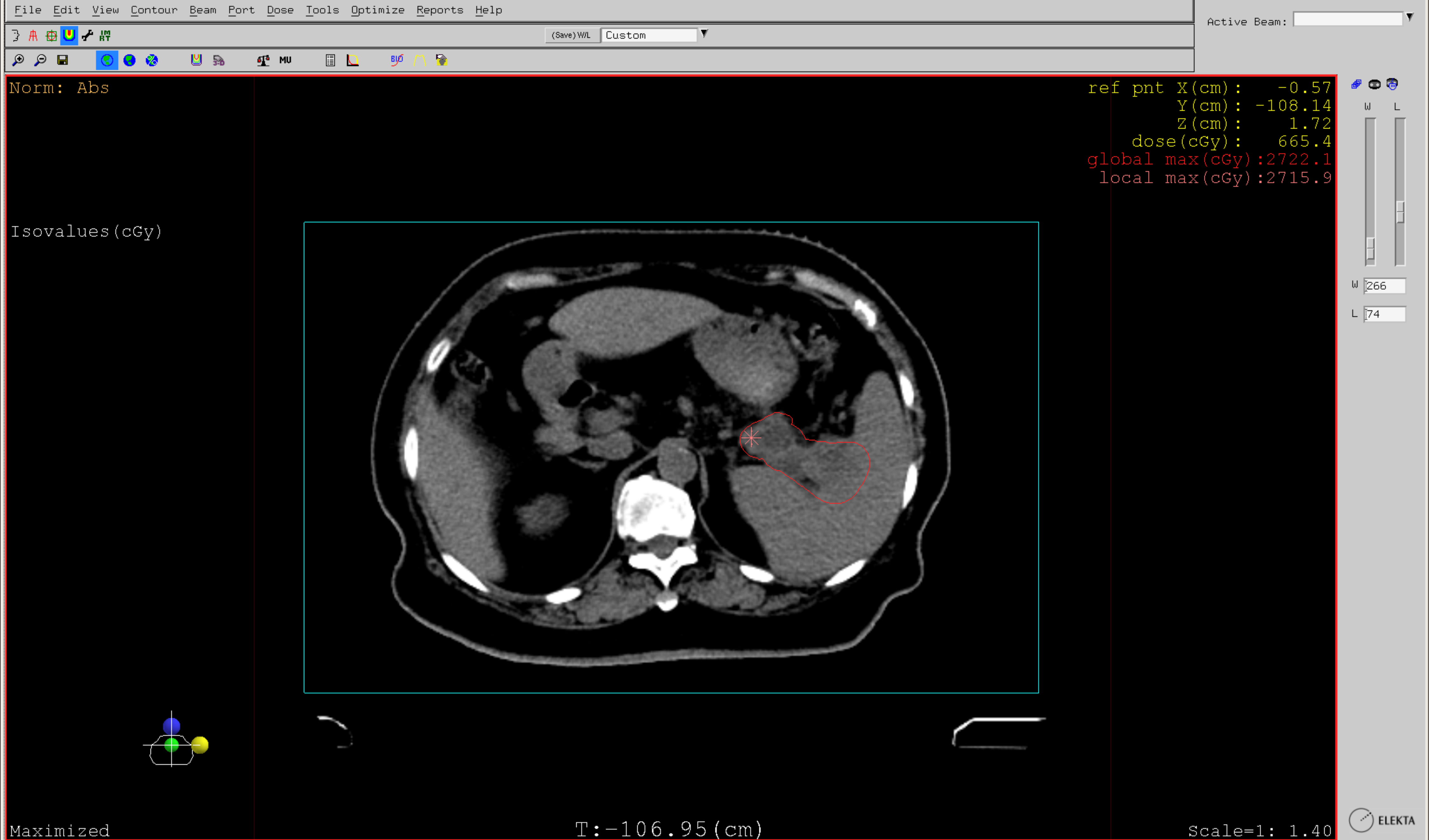
File Edit View Contour Beam Port Dose Tools Optimize Reports Help
Active Beam:
(Save) W/L
Custom
MU
Norm: Abs
ref pnt X(cm): -0.57
Y(cm): -108.14
Z(cm): 1.72
dose(cGy): 665.4
global max(cGy):2722.1
local max(cGy):2715.9
Isovalues(cGy)
W
L
W 266
L 74
Maximized
T:-106.95(cm)
Scale=1: 1.40
ELEKTA

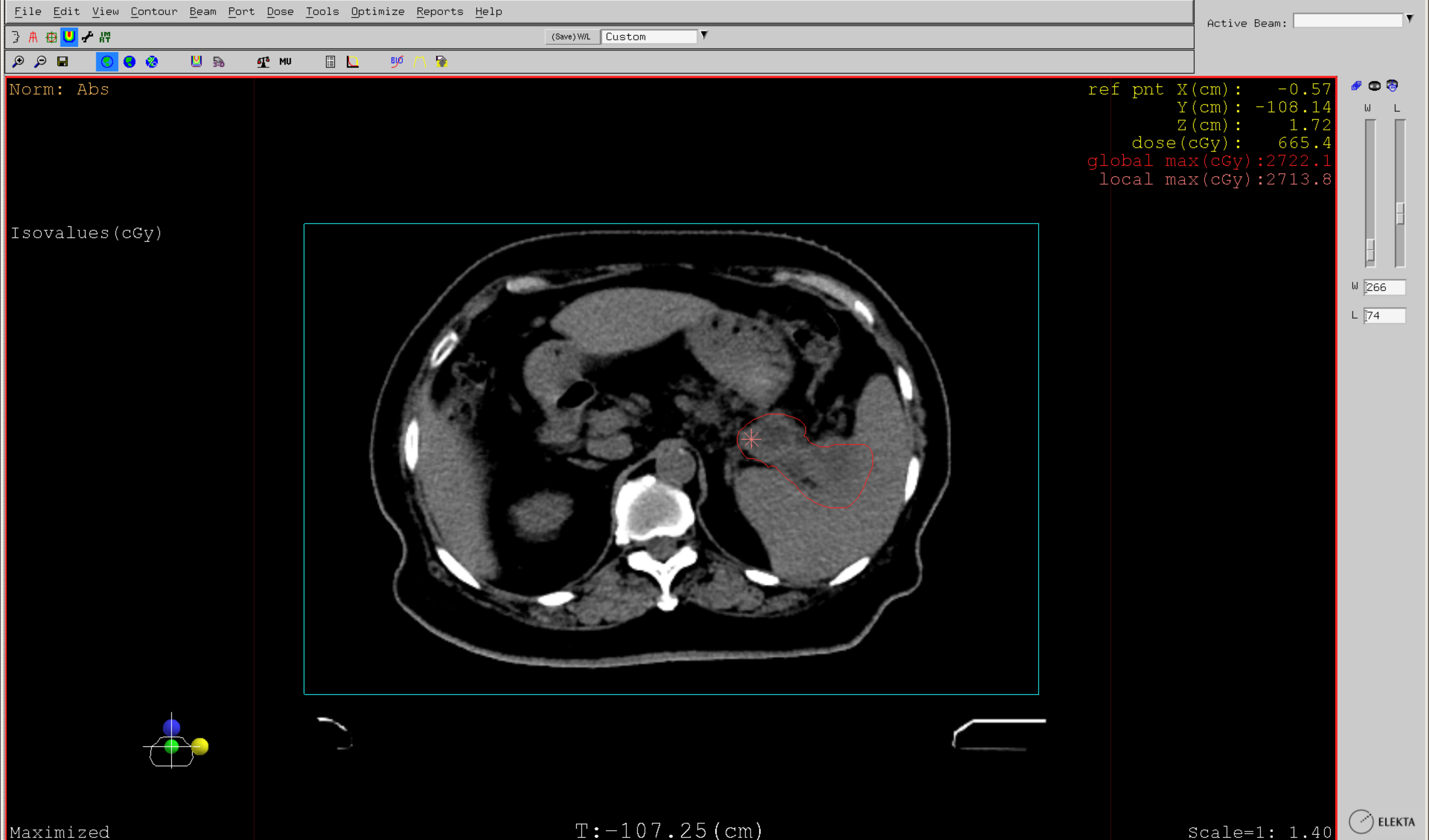
File Edit View Contour Beam Port Dose Tools Optimize Reports Help
(Save) W/L
Custom
Active Beam:
MU
Norm: Abs
ref pnt X(cm): -0.57
Y(cm): -108.14
Z(cm): 1.72
dose(cGy): 665.4
global max(cGy):2722.1
local max(cGy):2713.8
Isovalues(cGy)
W
L
W 266
L 74
Maximized
T:-107.25(cm)
Scale=1: 1.40
ELEKTA

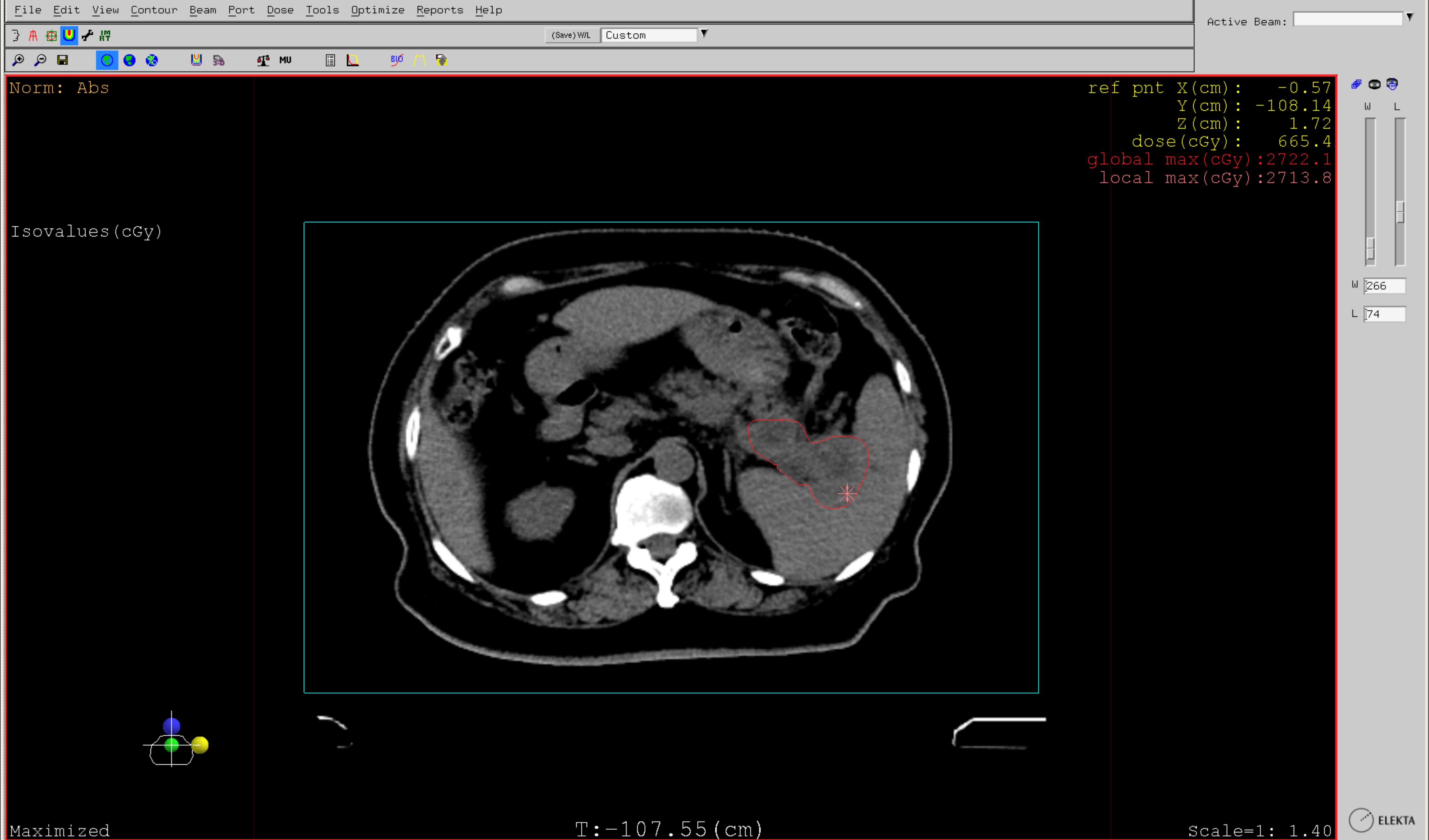
File Edit View Contour Beam Port Dose Tools Optimize Reports Help
Active Beam:
(Save) W/L
Custom
MU
Norm: Abs
ref pnt X(cm): -0.57
Y(cm): -108.14
Z(cm): 1.72
dose(cGy): 665.4
global max(cGy):2722.1
local max(cGy):2713.8
Isovalues(cGy)
W
L
W 266
L 74
Maximized
T:-107.55(cm)
Scale=1: 1.40
ELEKTA

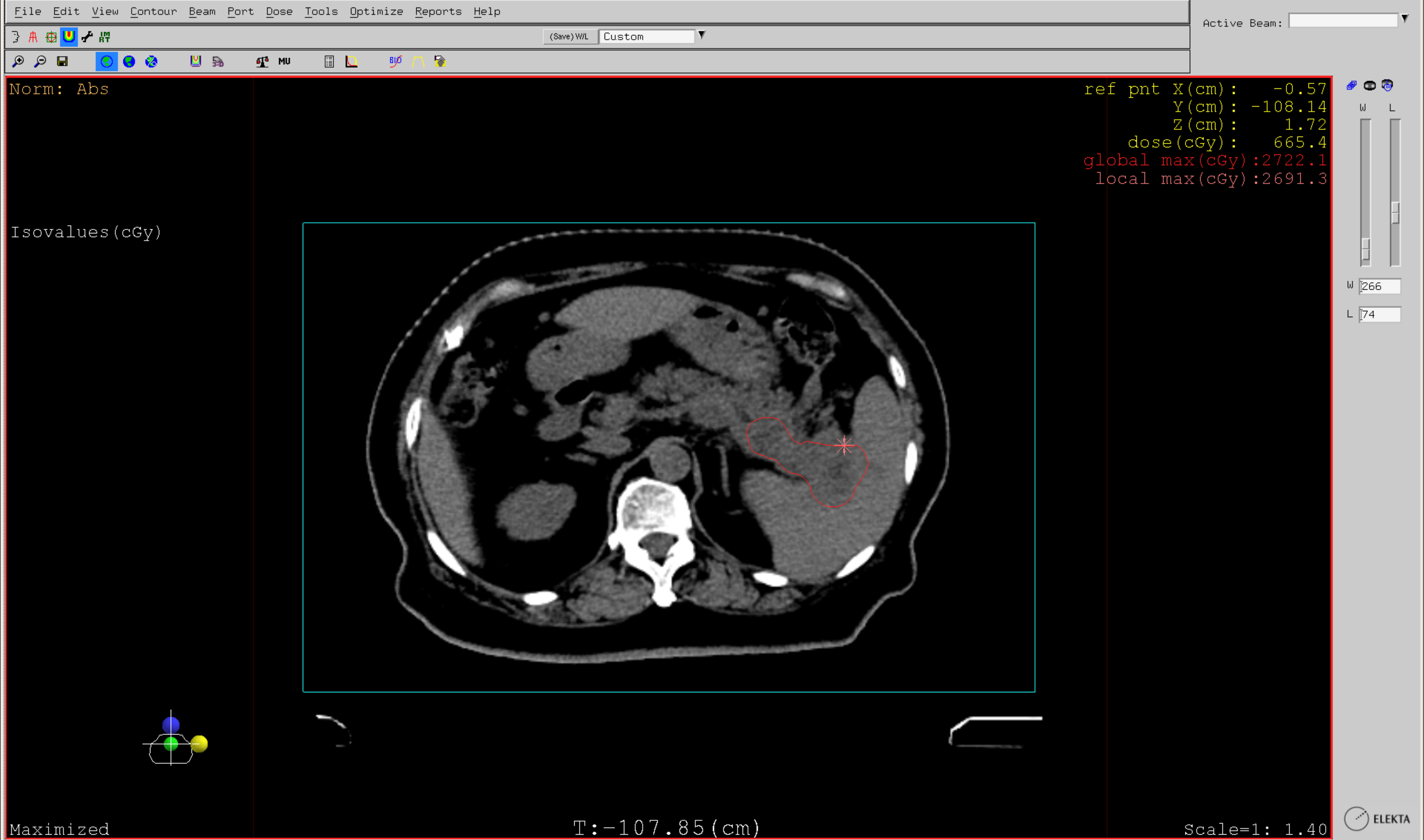
File Edit View Contour Beam Port Dose Tools Optimize Reports Help
Active Beam:
(Save) W/L
Custom
MU
Norm: Abs
ref pnt X(cm): -0.57
Y(cm): -108.14
Z(cm): 1.72
dose(cGy): 665.4
global max(cGy):2722.1
local max(cGy):2691.3
W
L
W 266
L 74
Isovalues(cGy)
Maximized
T:-107.85(cm)
Scale=1: 1.40
ELEKTA

File Edit View Contour Beam Port Dose Tools Optimize Reports Help

Active Beam:

(Save) W/L Custom

MU

Norm: Abs

ref pnt X(cm): -0.57
Y(cm): -108.14
Z(cm): 1.72
dose(cGy): 665.4
global max(cGy):2722.1
local max(cGy):2681.5

W L

W 266

L 74

Isovalues(cGy)

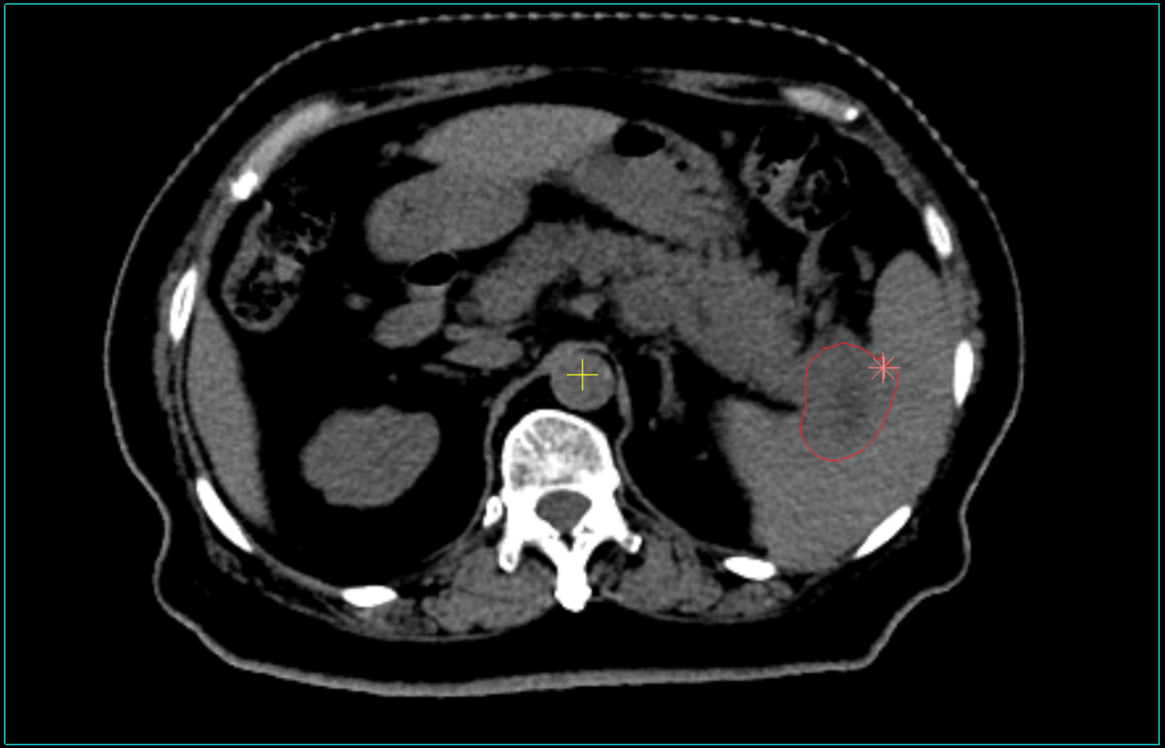

Maximized

T:-108.15(cm)

Scale=1: 1.40

ELEKTA

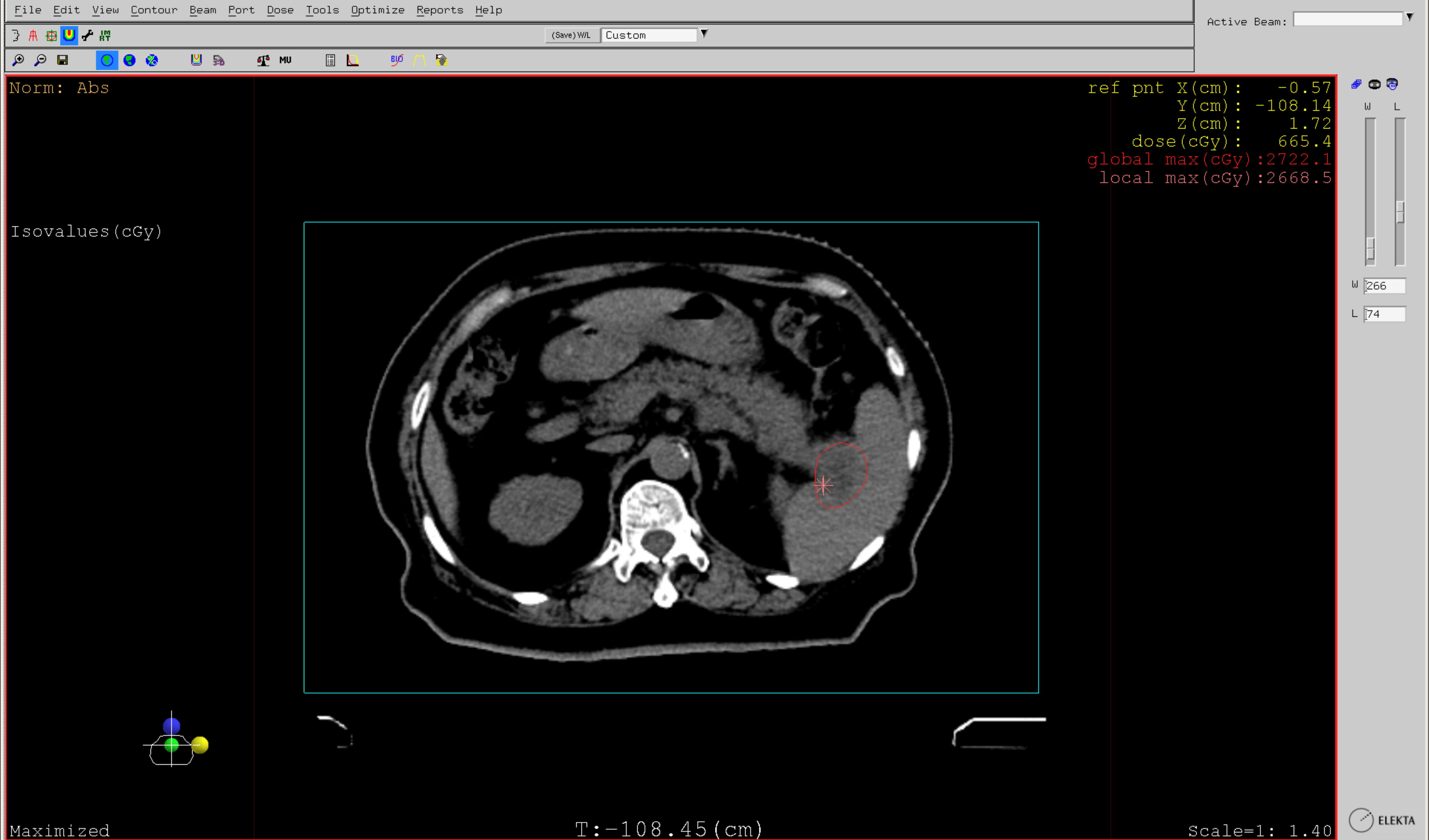
File Edit View Contour Beam Port Dose Tools Optimize Reports Help
Active Beam:
(Save) W/L
Custom
MU
Norm: Abs
ref pnt X(cm): -0.57
Y(cm): -108.14
Z(cm): 1.72
dose(cGy): 665.4
global max(cGy):2722.1
local max(cGy):2668.5
Isovalues(cGy)
W
L
W 266
L 74
Maximized
T:-108.45(cm)
Scale=1: 1.40
ELEKTA

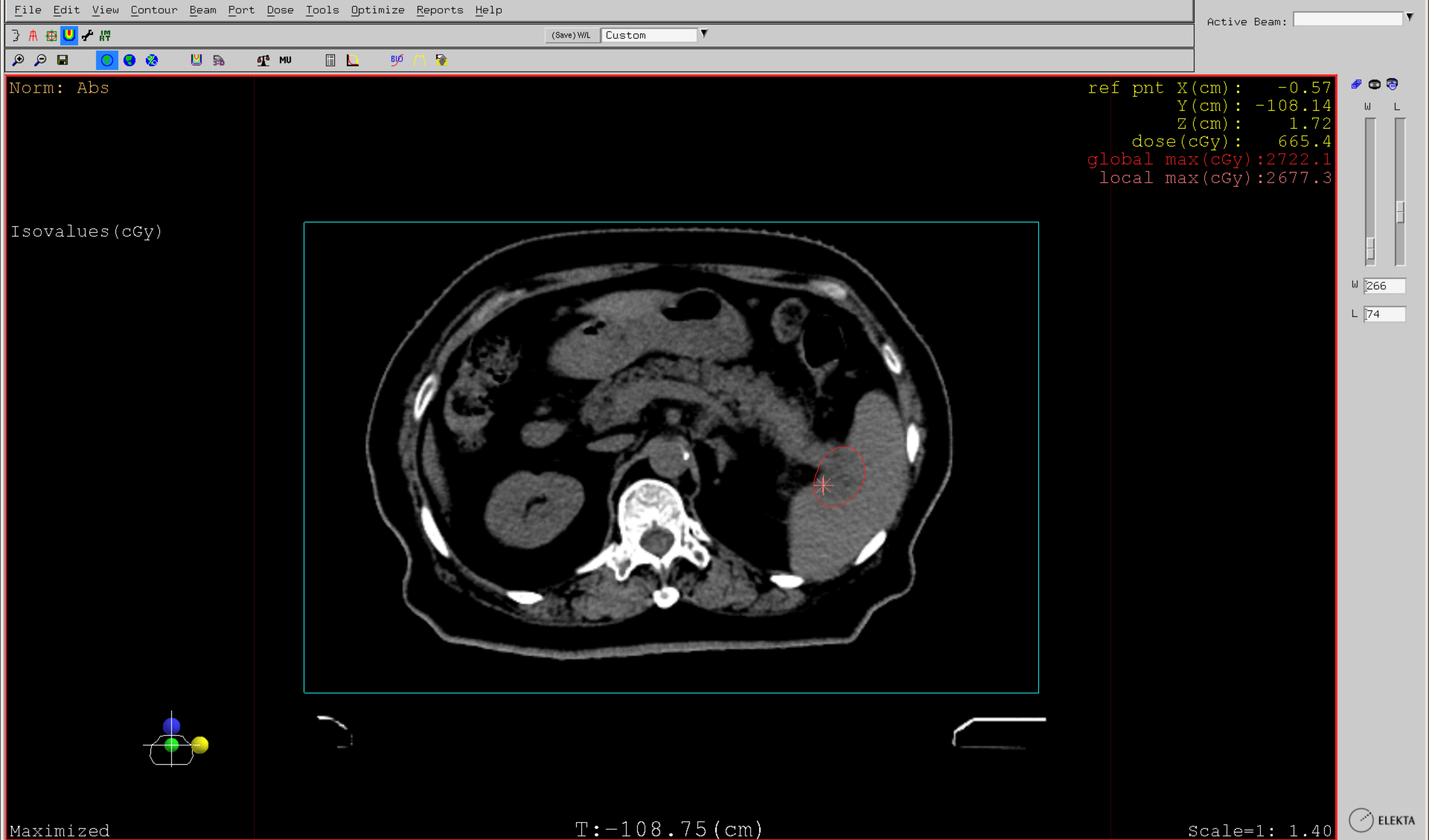

File Edit View Contour Beam Port Dose Tools Optimize Reports Help
Active Beam:
(Save) W/L
Custom
MU
Norm: Abs
ref pnt X(cm): -0.57
Y(cm): -108.14
Z(cm): 1.72
dose(cGy): 665.4
global max(cGy):2722.1
local max(cGy):2677.3
Isovalues(cGy)
W
L
W 266
L 74
Maximized
T:-108.75(cm)
Scale=1: 1.40
ELEKTA

File Edit View Contour Beam Port Dose Tools Optimize Reports Help

Active Beam:

(Save) W/L Custom

MU

Norm: Abs

ref pnt X(cm): -0.57
Y(cm): -108.14
Z(cm): 1.72
dose(cGy): 665.4
global max(cGy):2722.1
local max(cGy):2648.1

Isovalues(cGy)

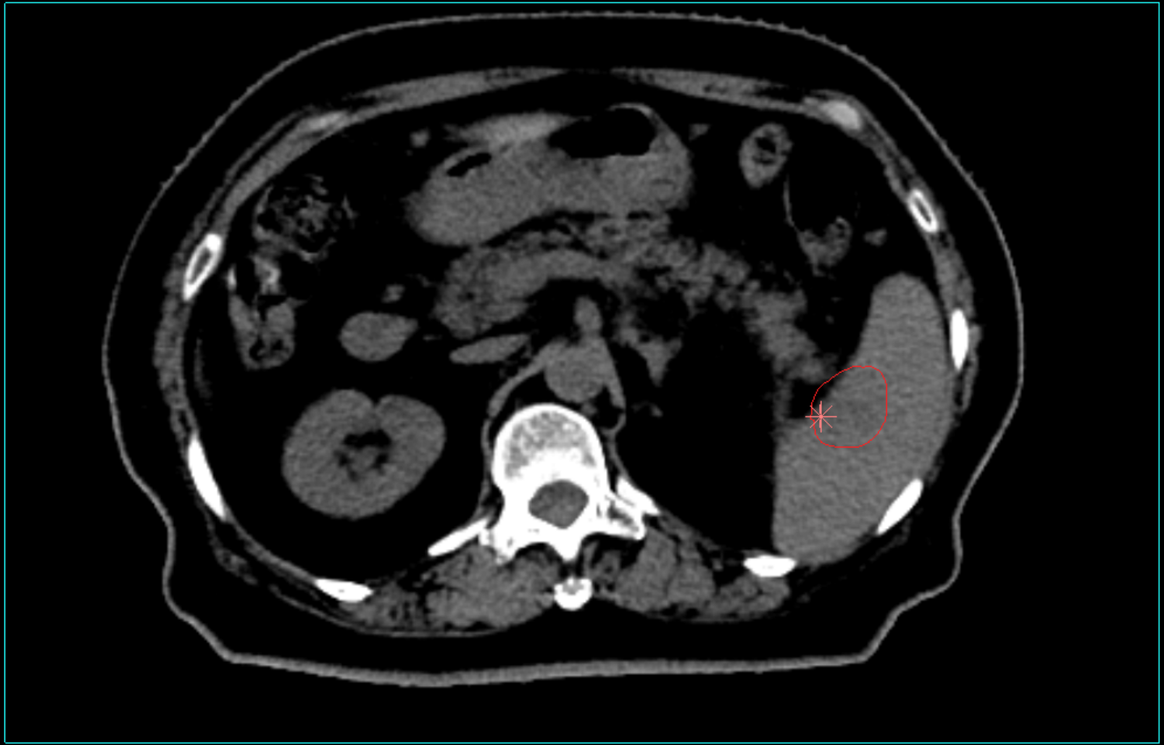

W L

W 266

L 74

Maximized

T:-109.05(cm)

Scale=1: 1.40

ELEKTA
