# Supplementary material for: Complete response to BRICS in Locally advanced pancreatic cancer (pMMR, CPS 30): a case report
Source: Front Immunol. 2026 Jan 21;17:1743752. doi: 10.3389/fimmu.2026.1743752 (PMC12867830; doi:10.3389/fimmu.2026.1743752)
Supplement: Supplementary Figure 3 — GTV delineation. (The GTV is delineated by the red line). [file DataSheet3.pdf]

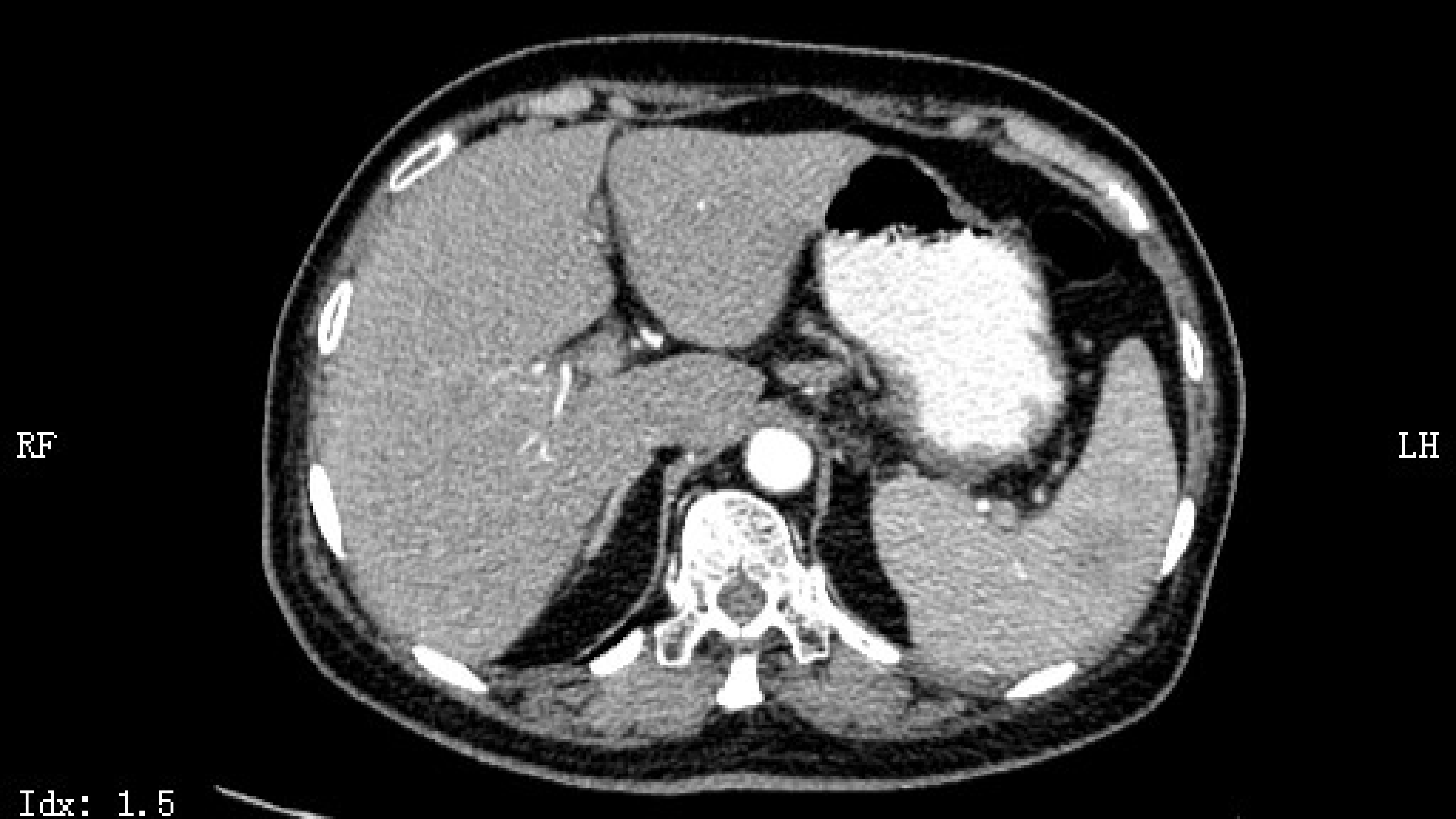
RF
LH
Idx: 1.5

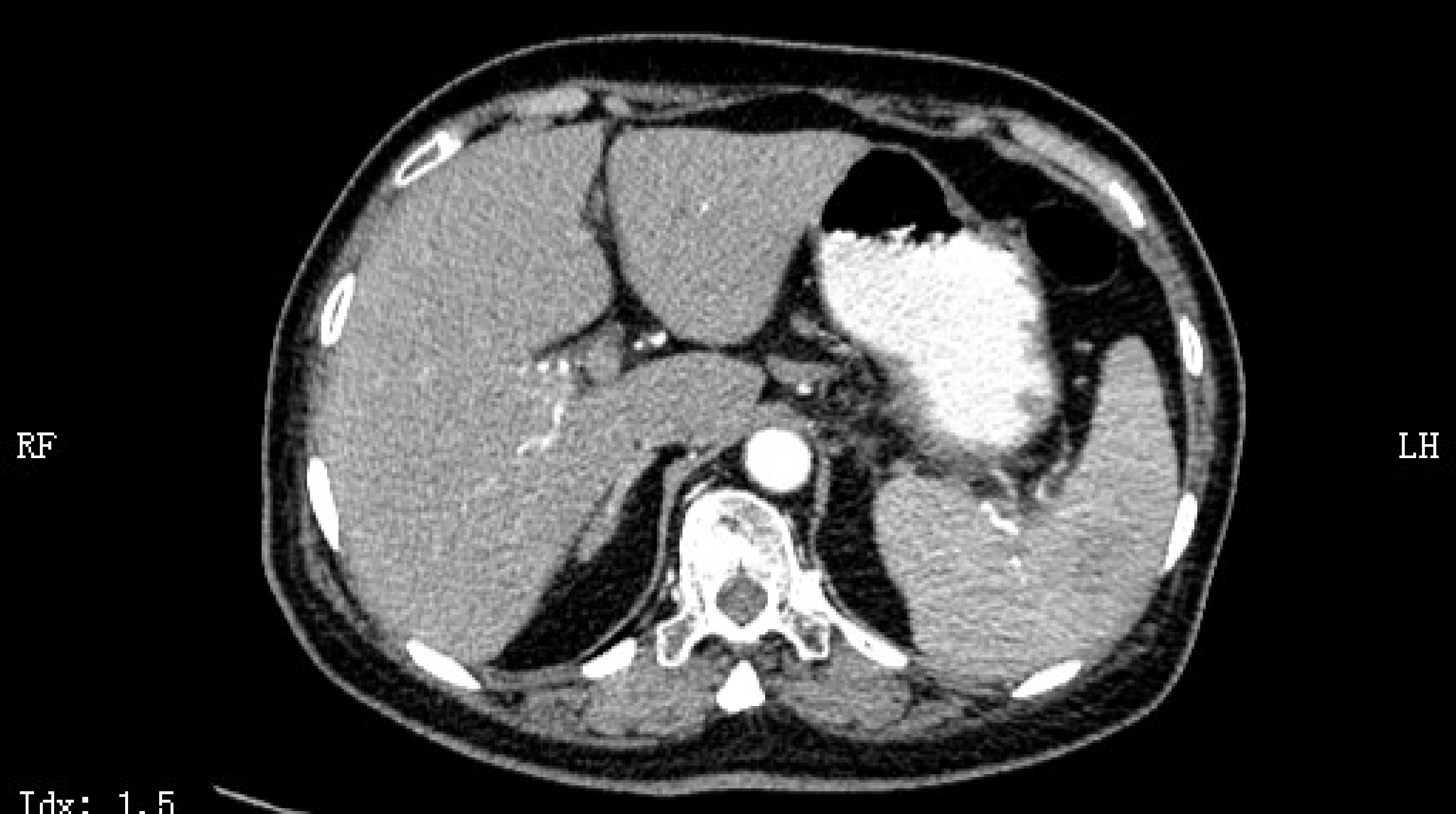
RF
LH
Idx: 1.5

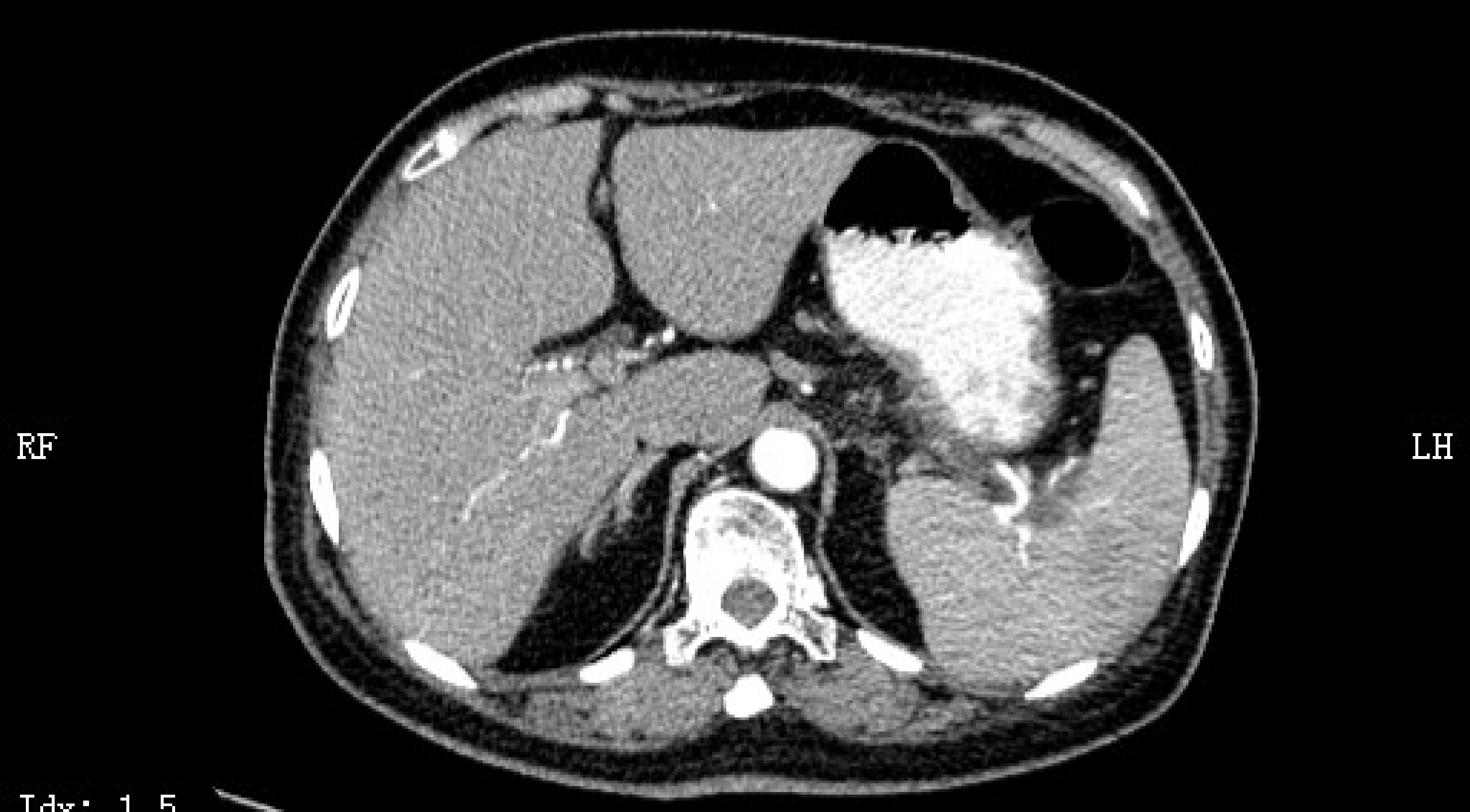
RF
LH
Idx: 1.5

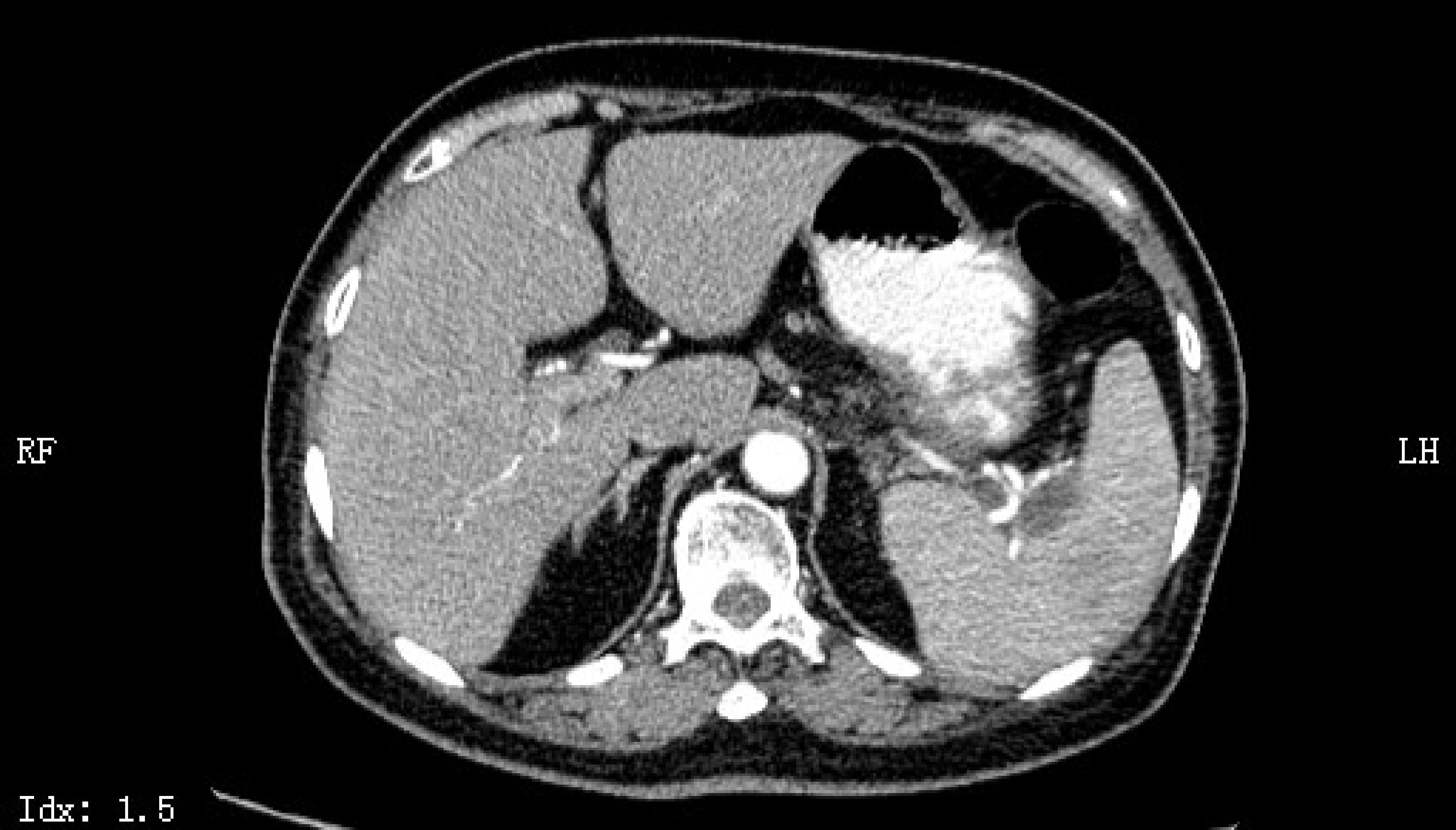
RF
LH
Idx: 1.5

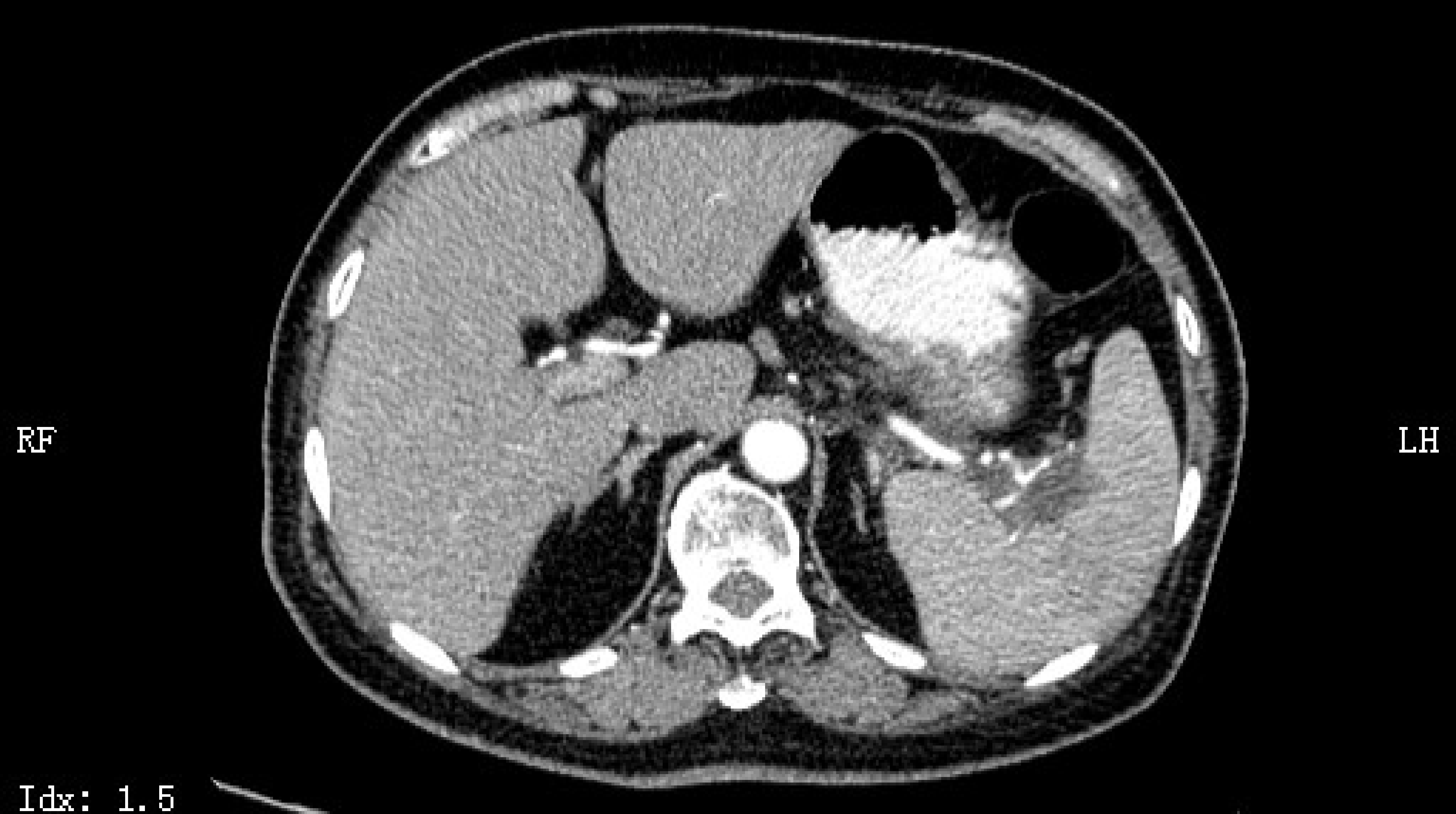

RF
LH
Idx: 1.5

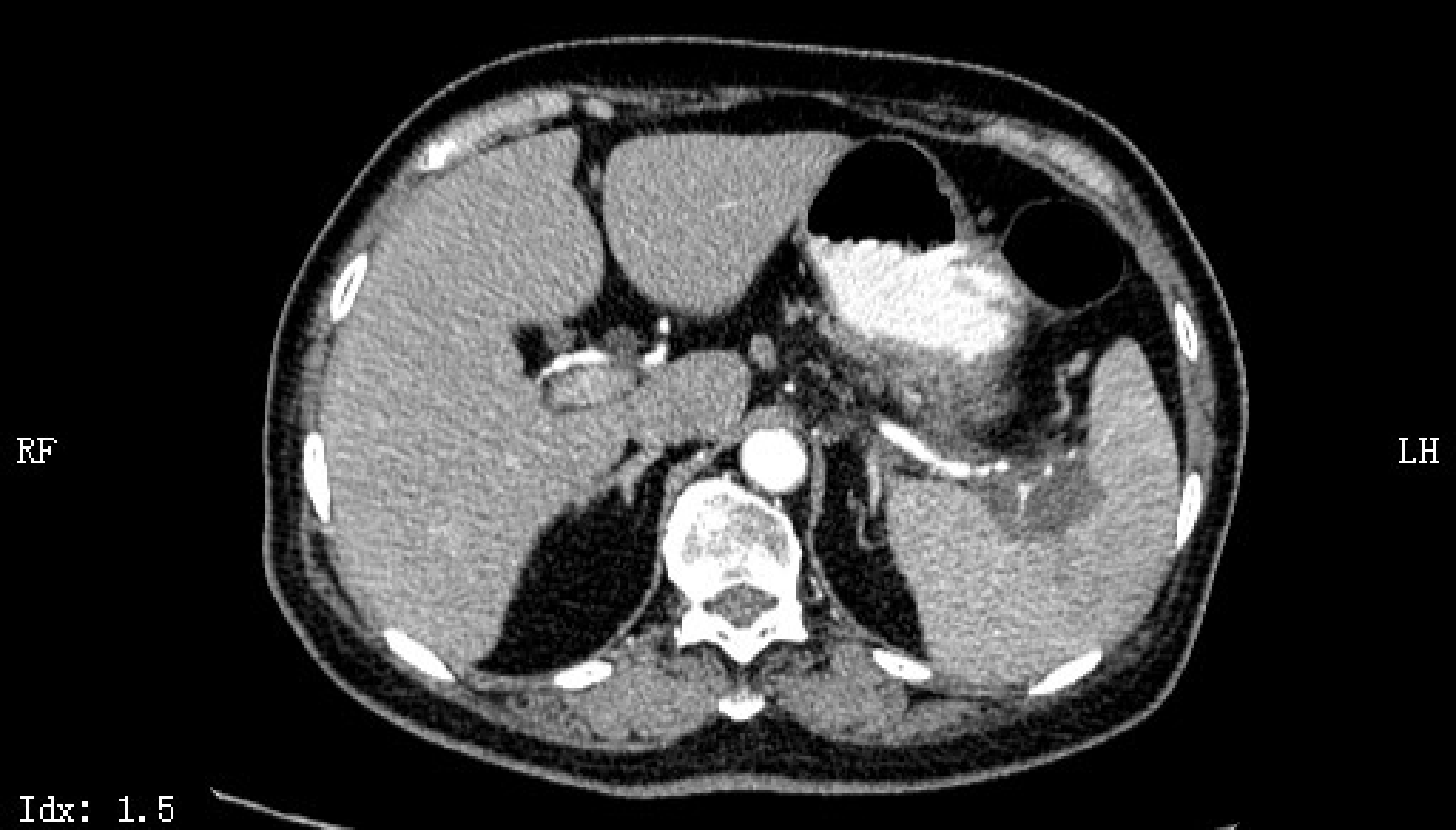
RF
LH
Idx: 1.5

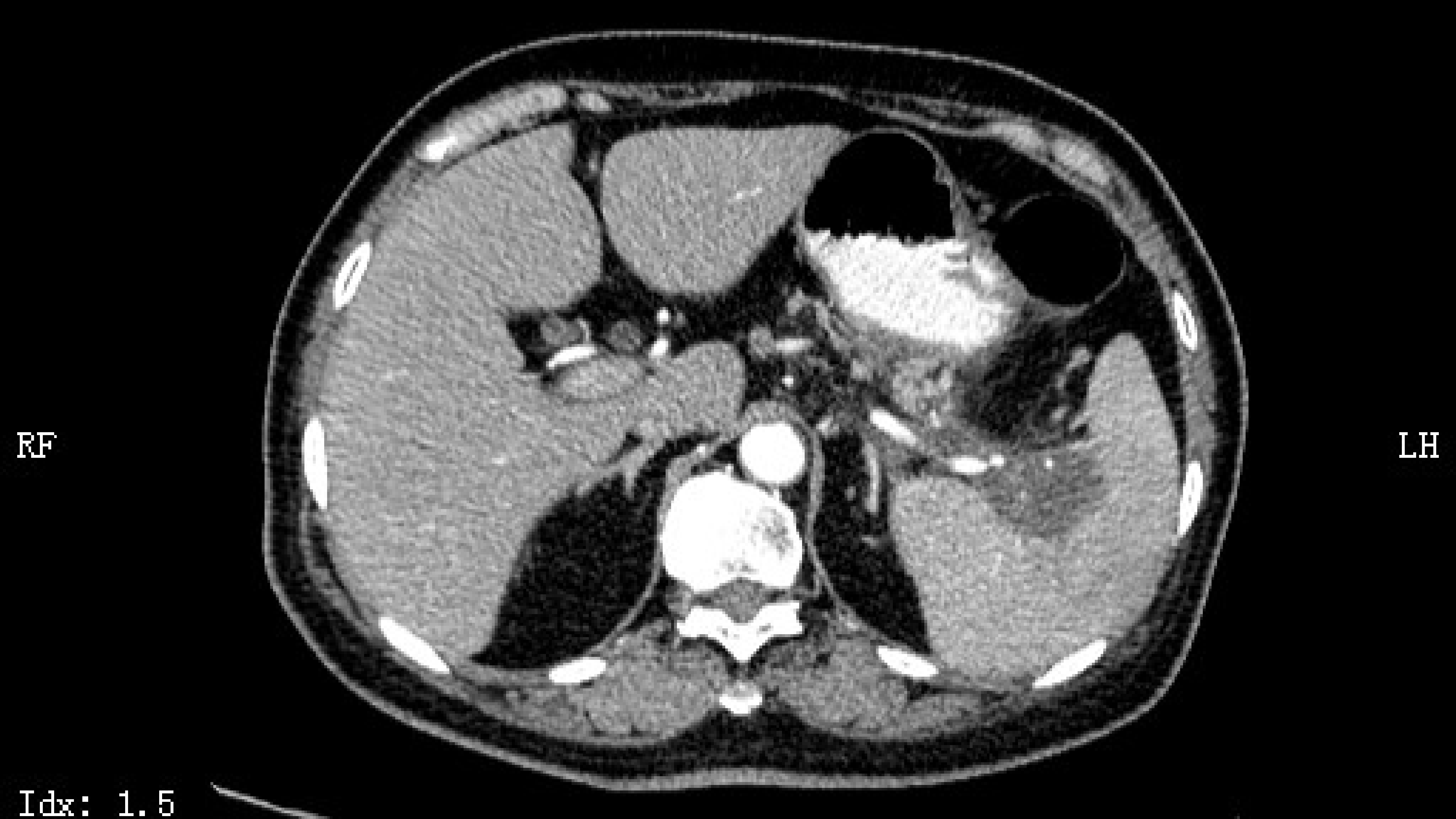
RF
LH
Idx: 1.5

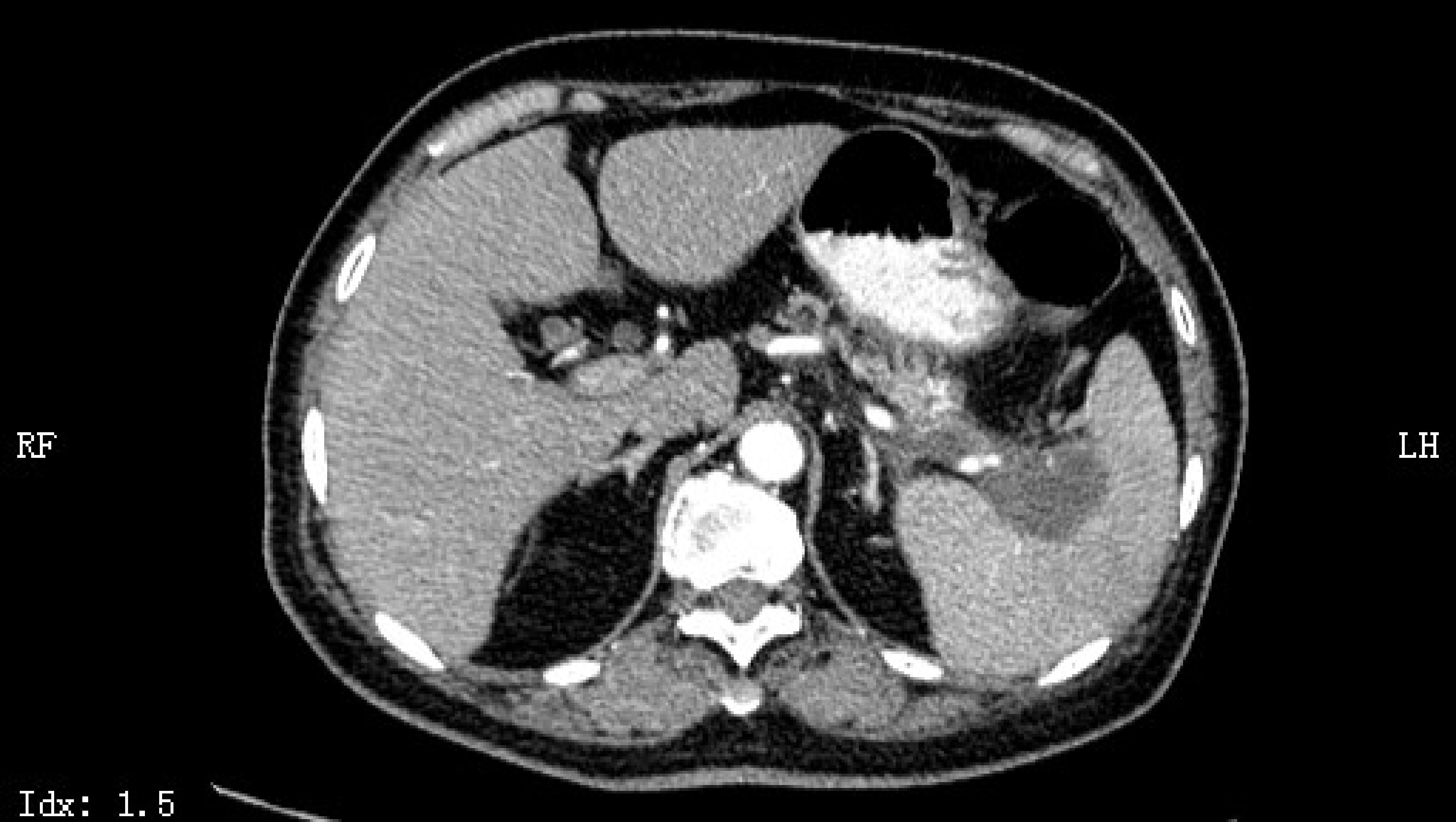
RF
LH
Idx: 1.5

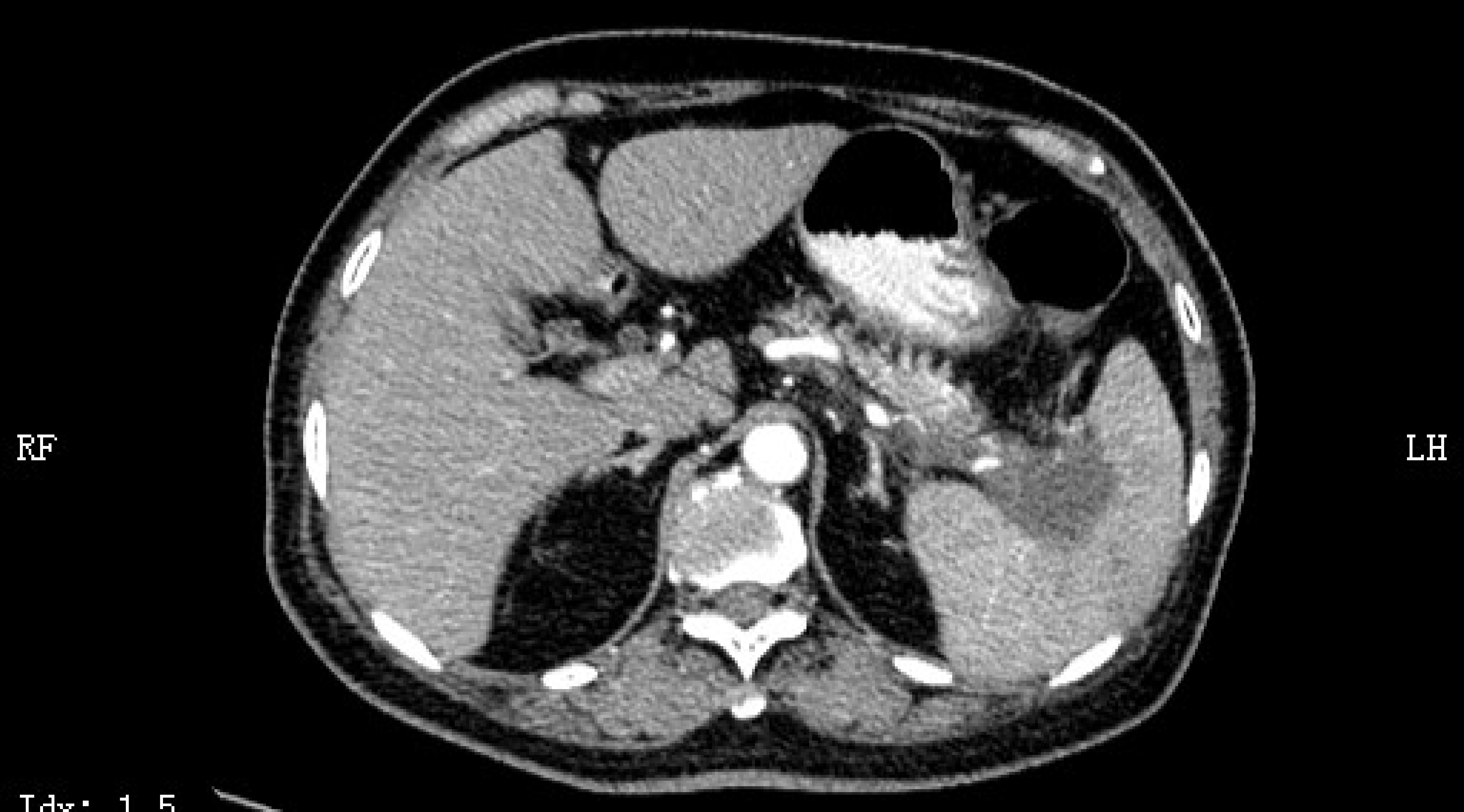
RF
LH
Idx: 1.5

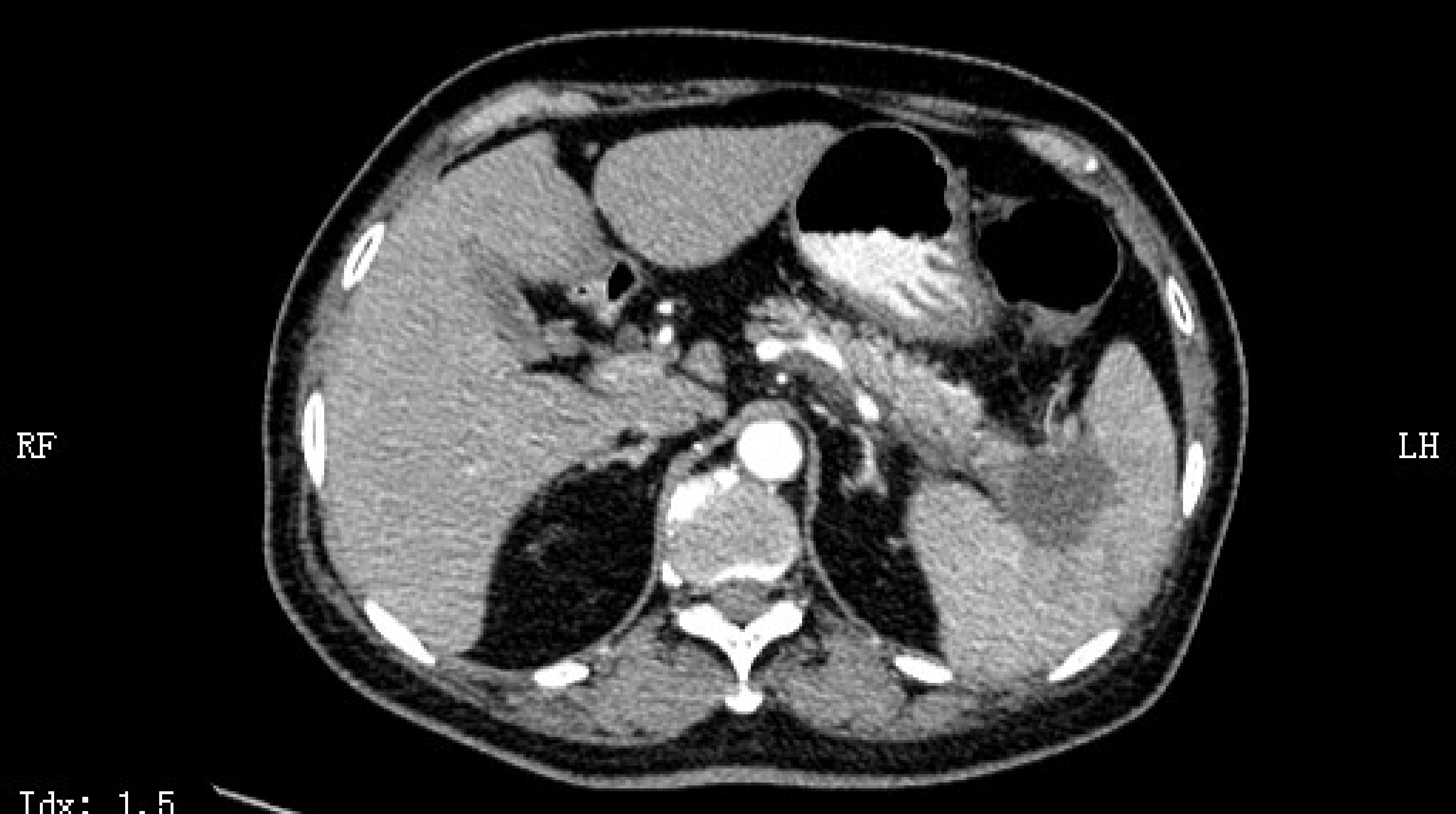
RF
LH
Idx: 1.5

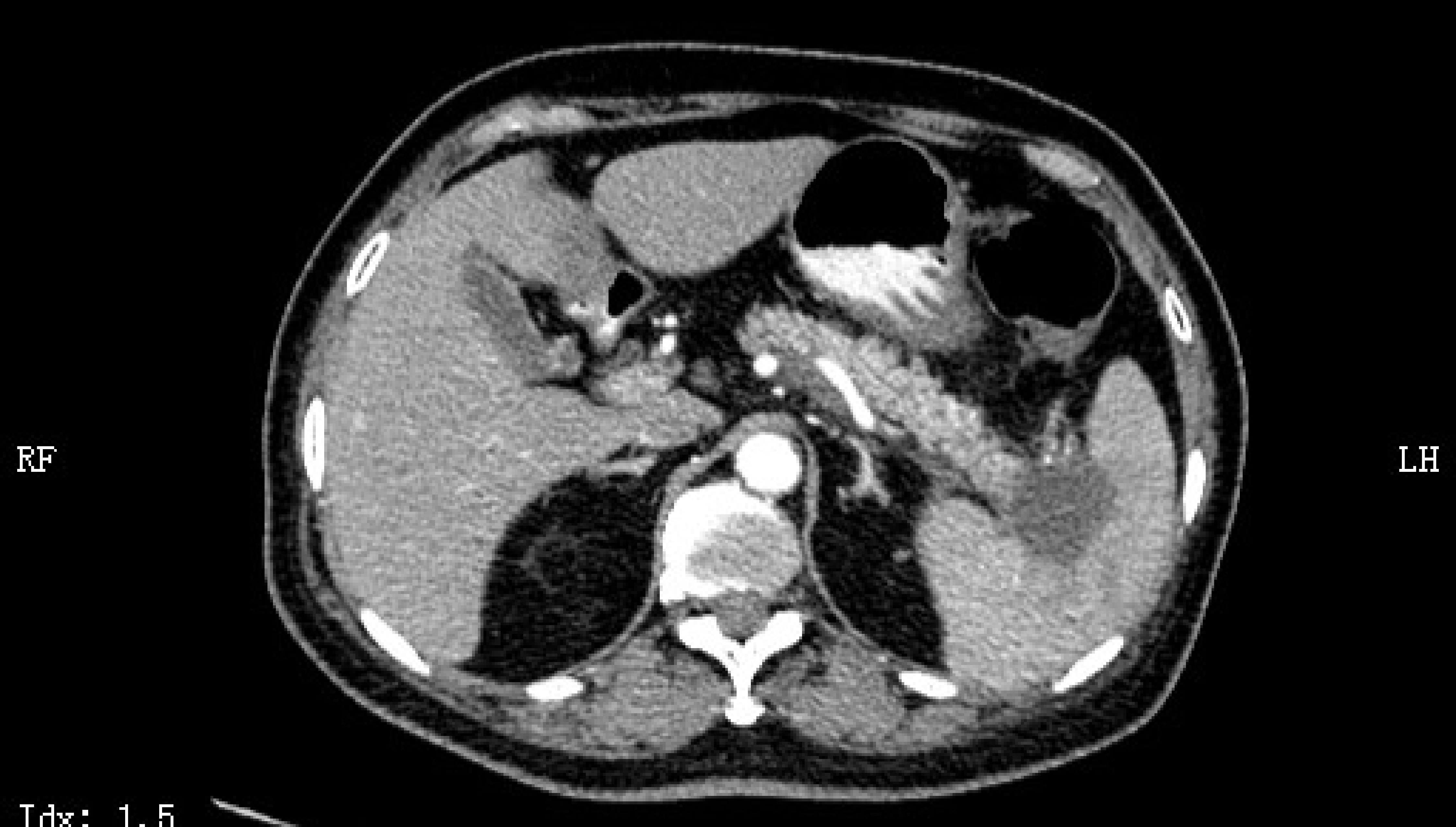
RF
LH
Idx: 1.5

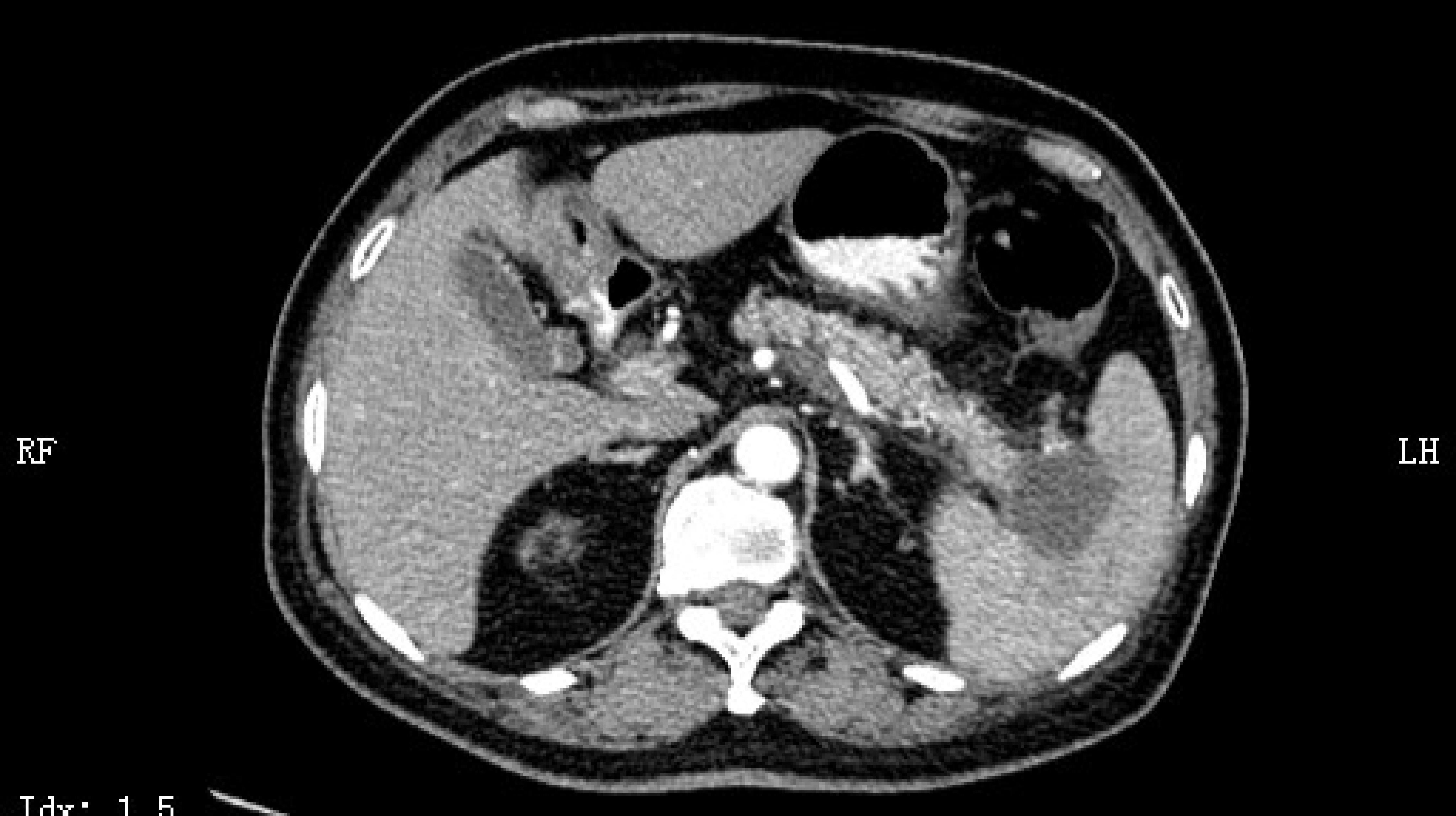
RF
LH

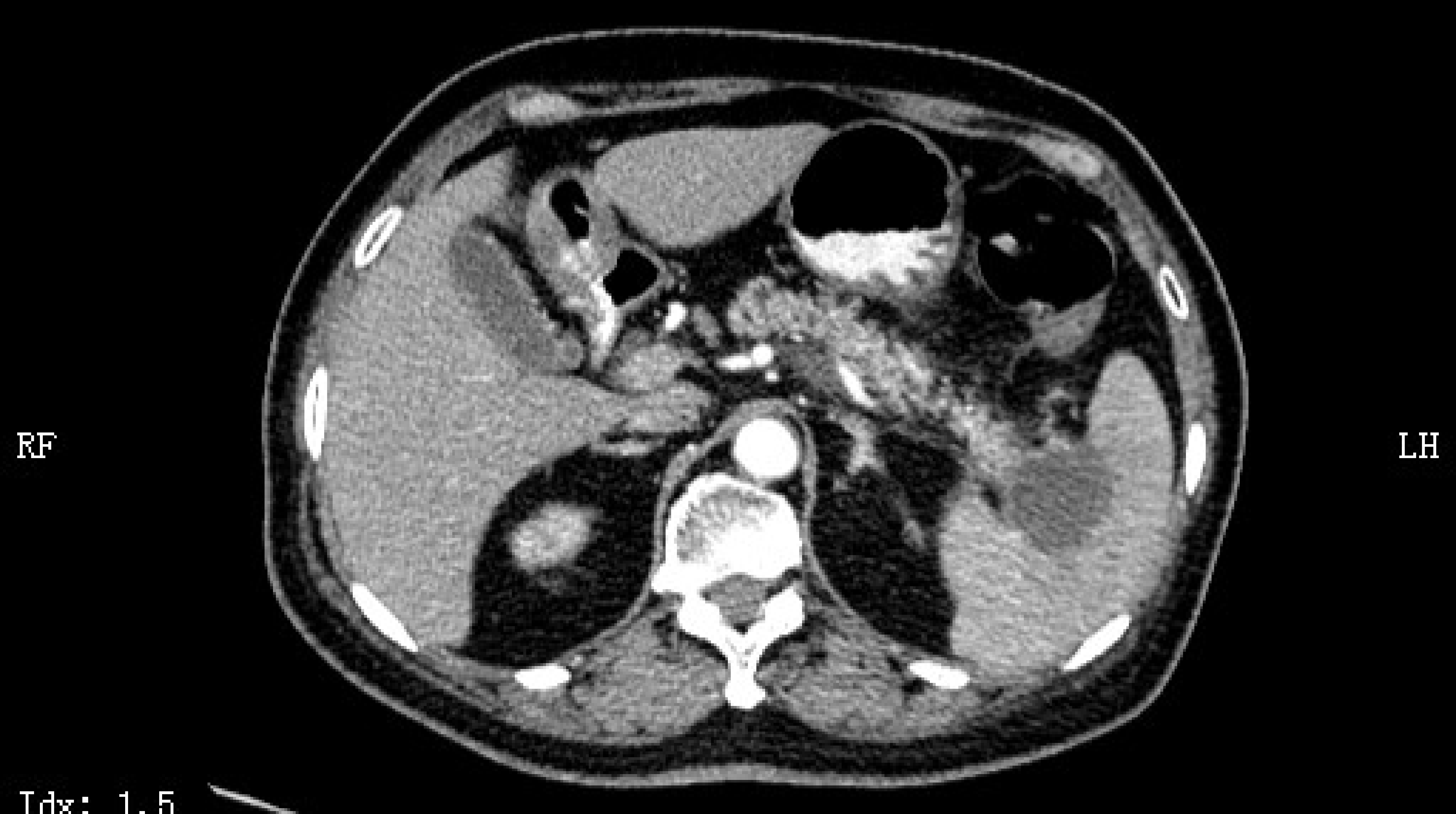
RF
LH
Idx: 1.5

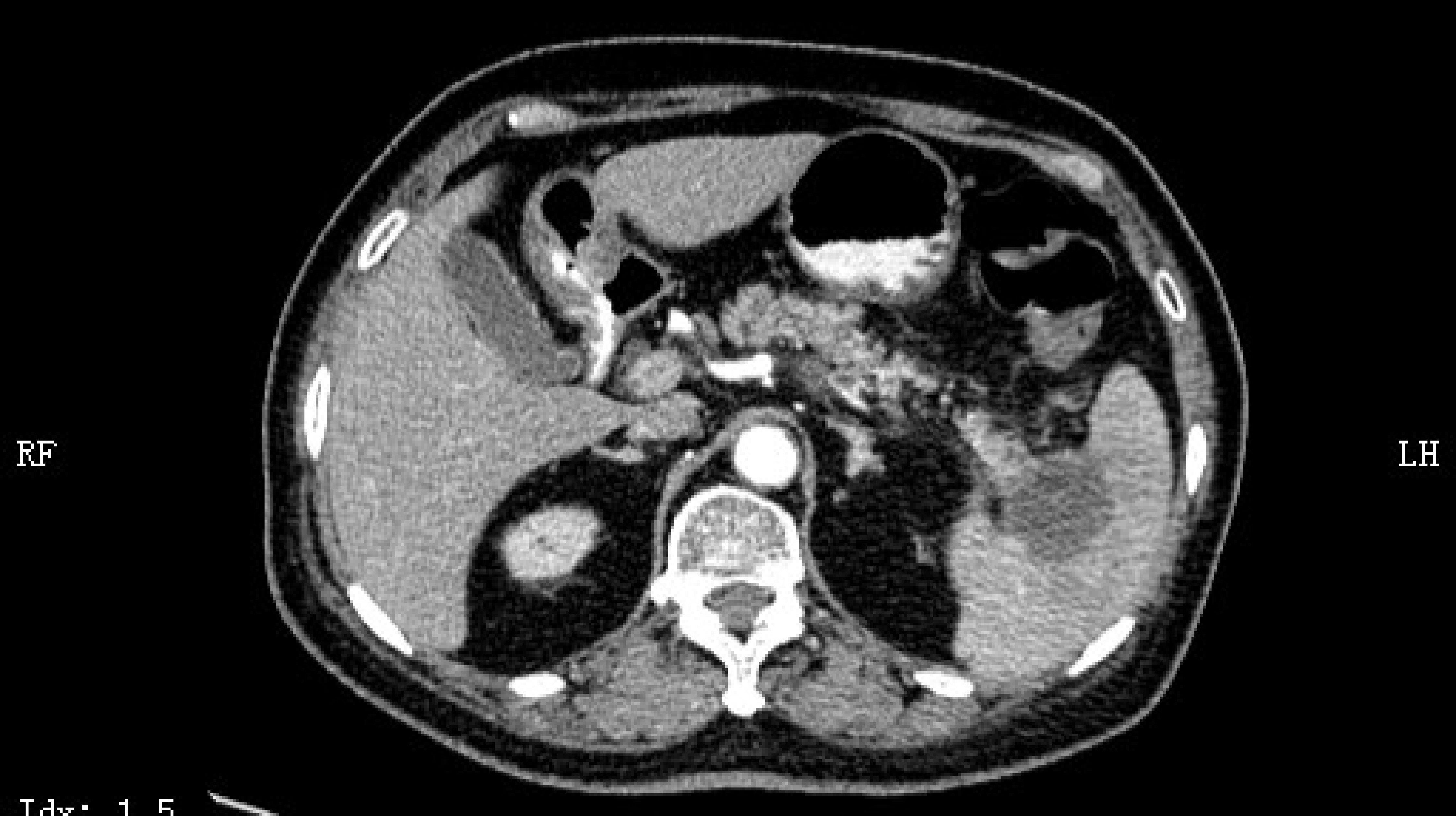
RF
LH
Idx: 1.5

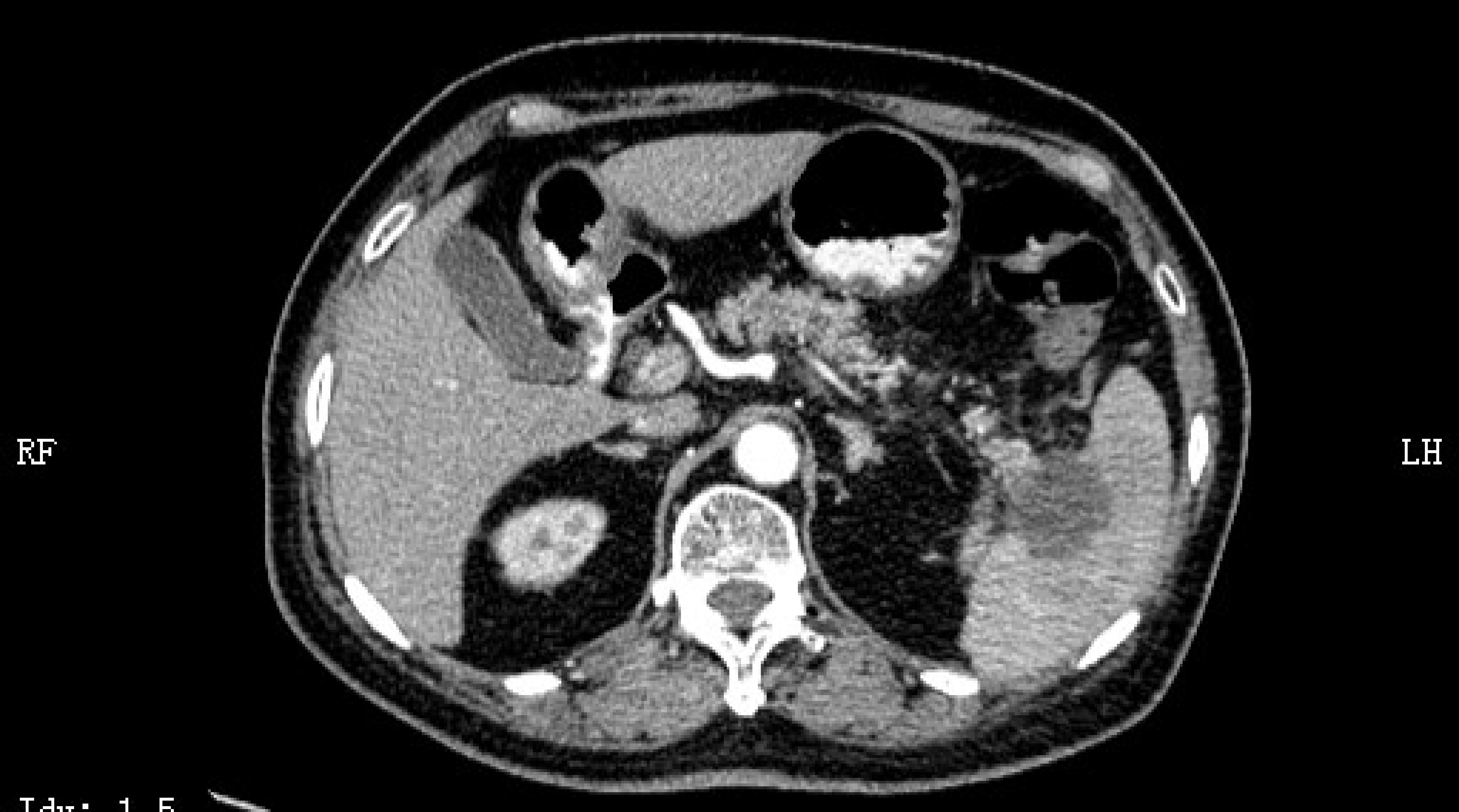
RF
LH

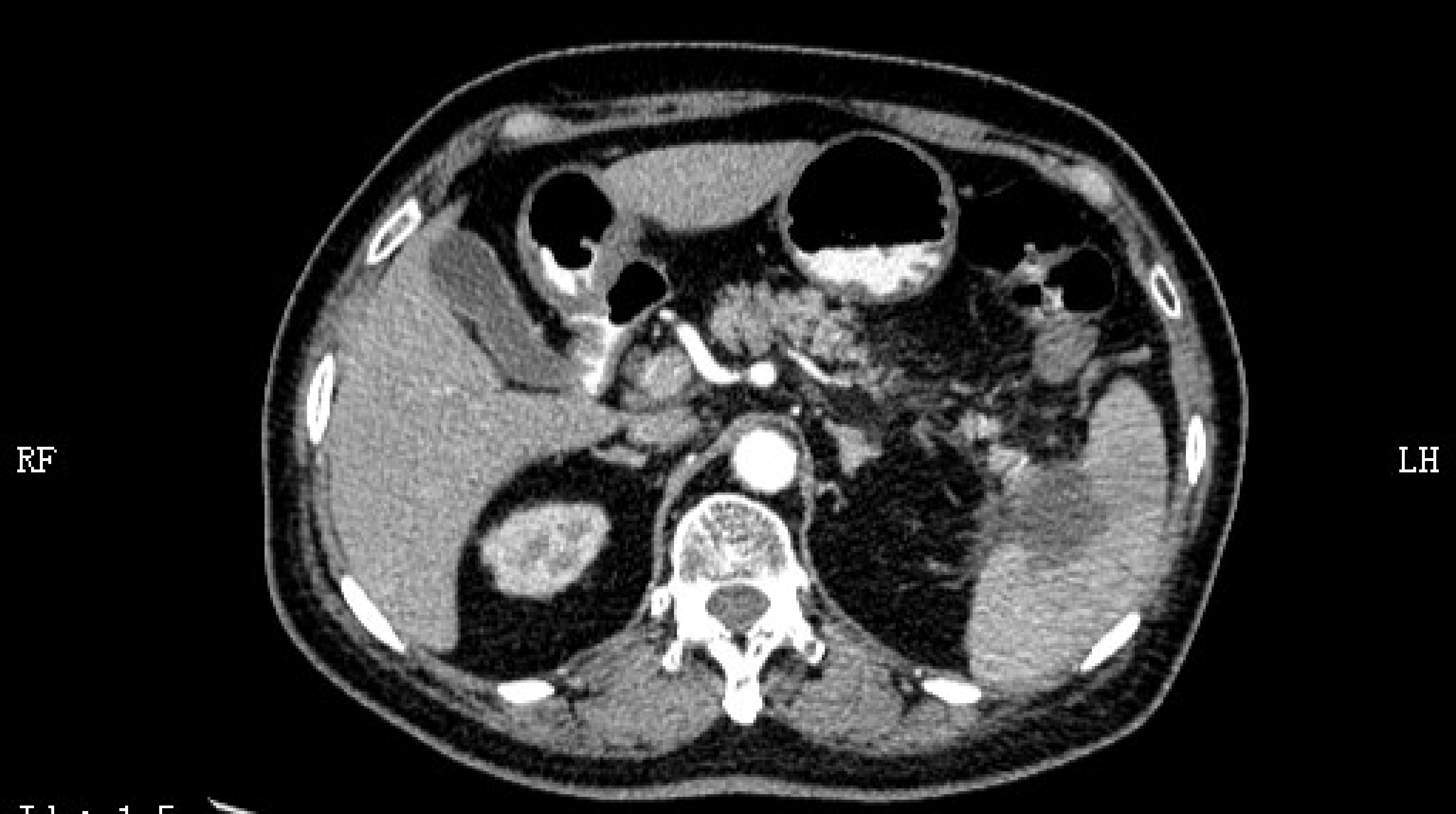
RF
LH

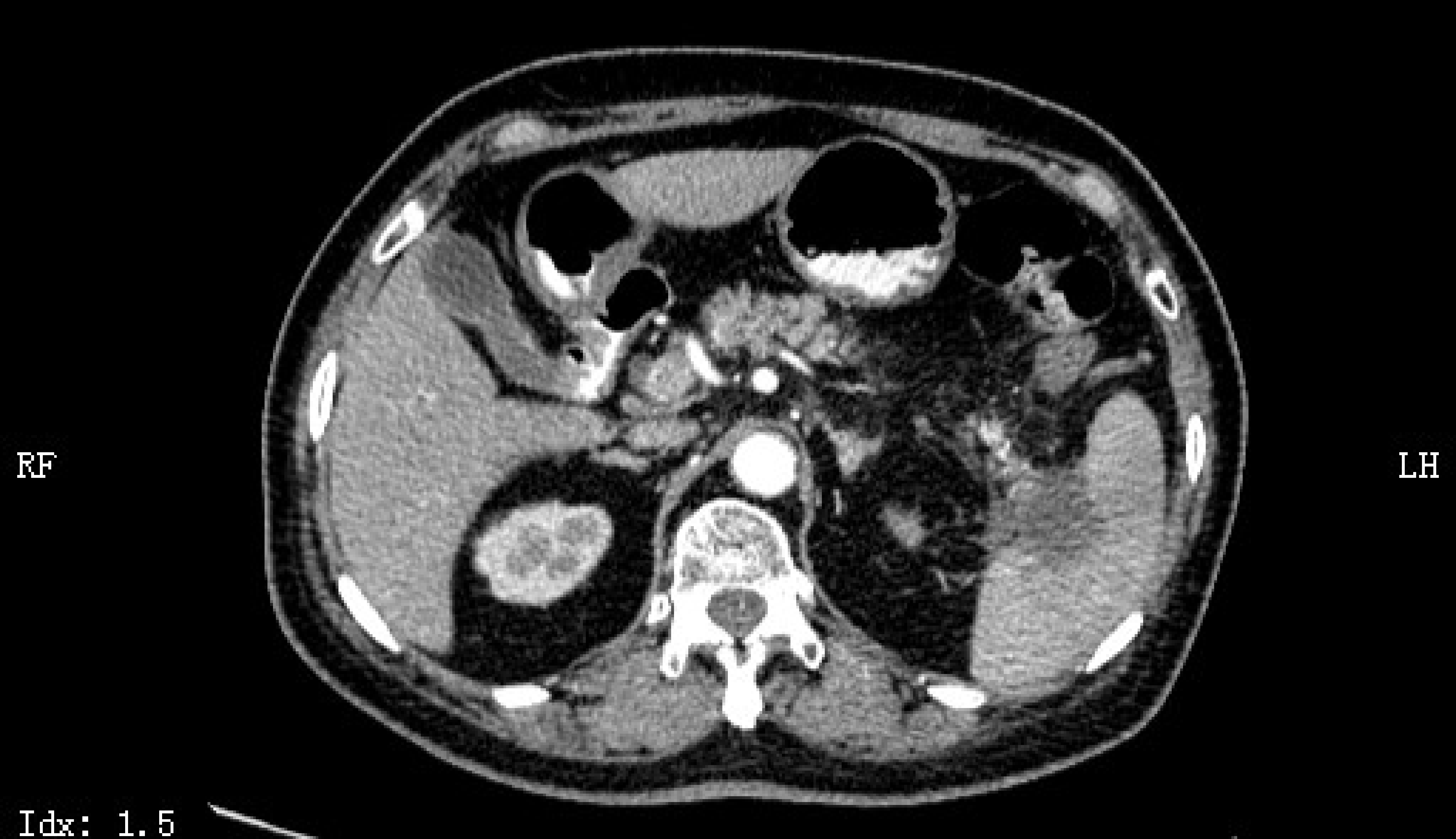
RF
LH
Idx: 1.5

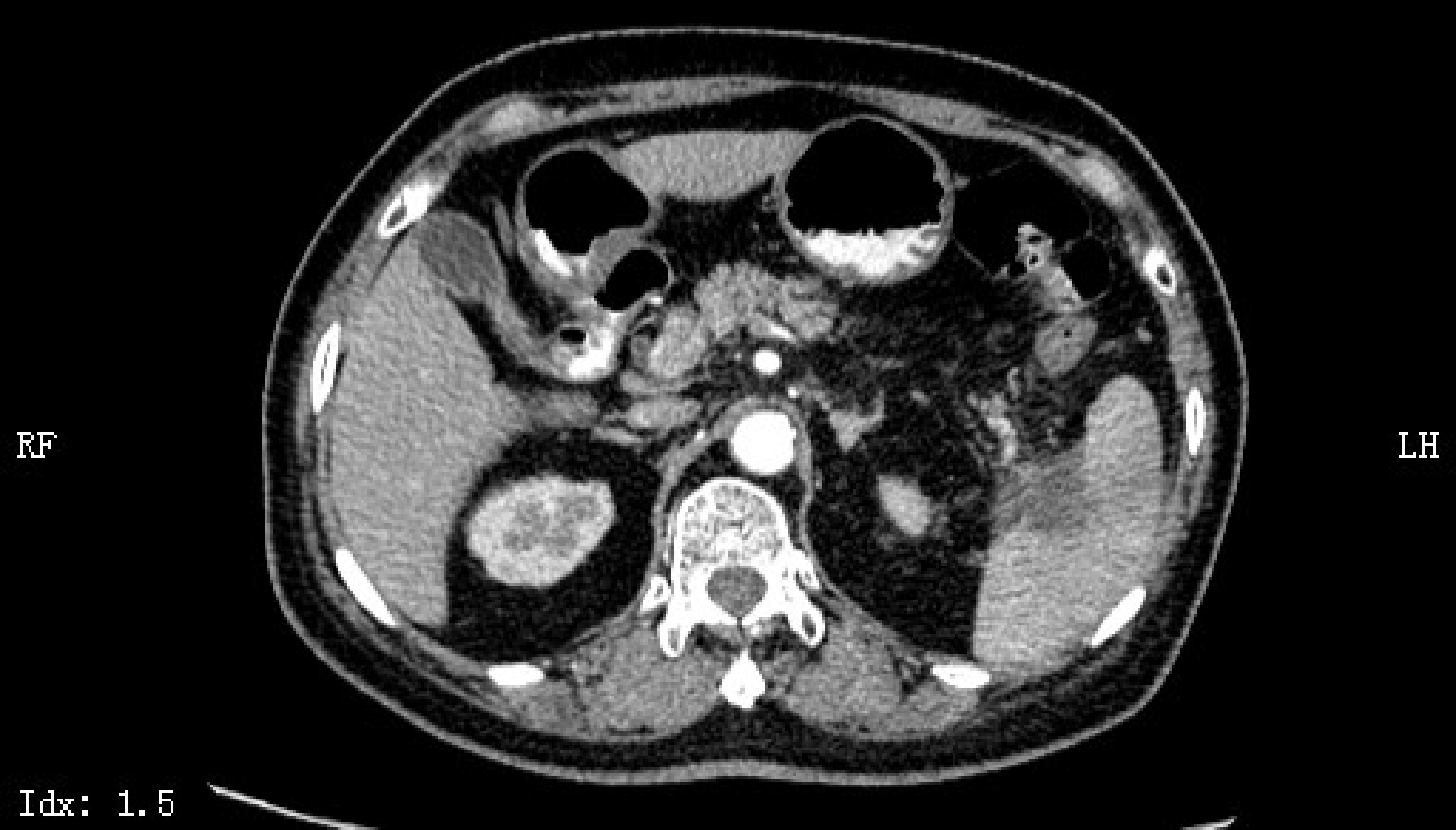
RF
LH
Idx: 1.5

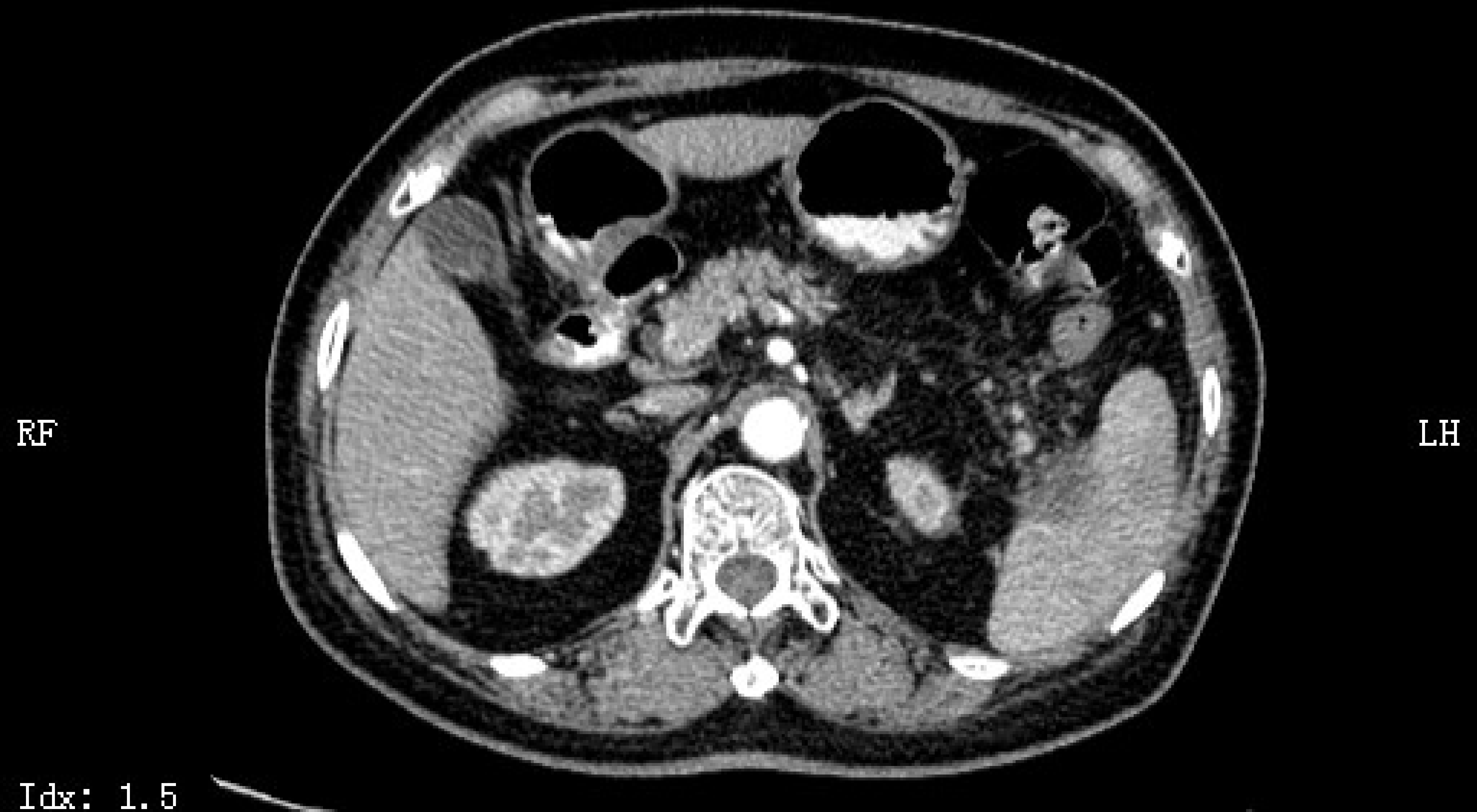
RF
LH
Idx: 1.5

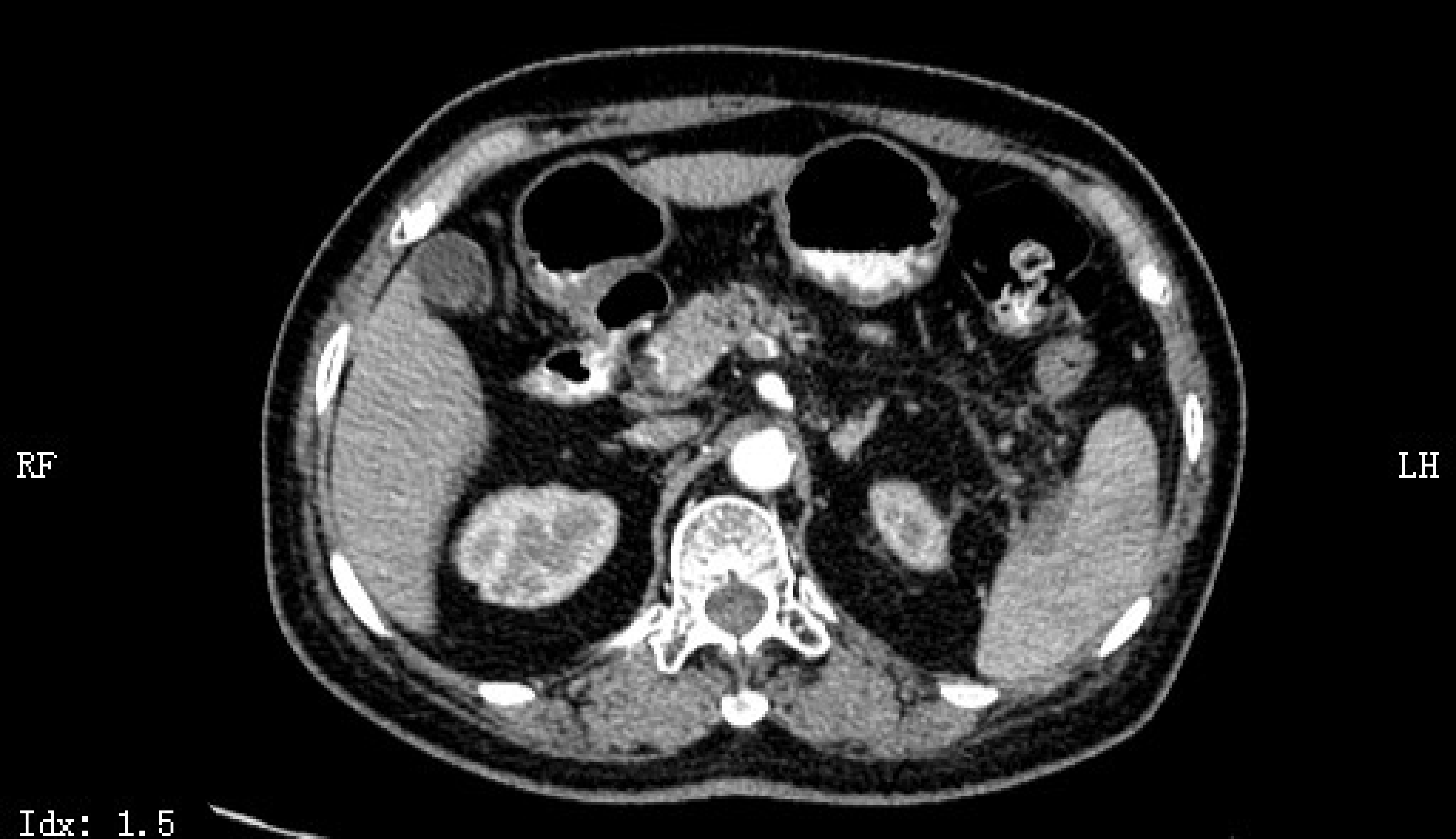

RF
LH
Idx: 1.5

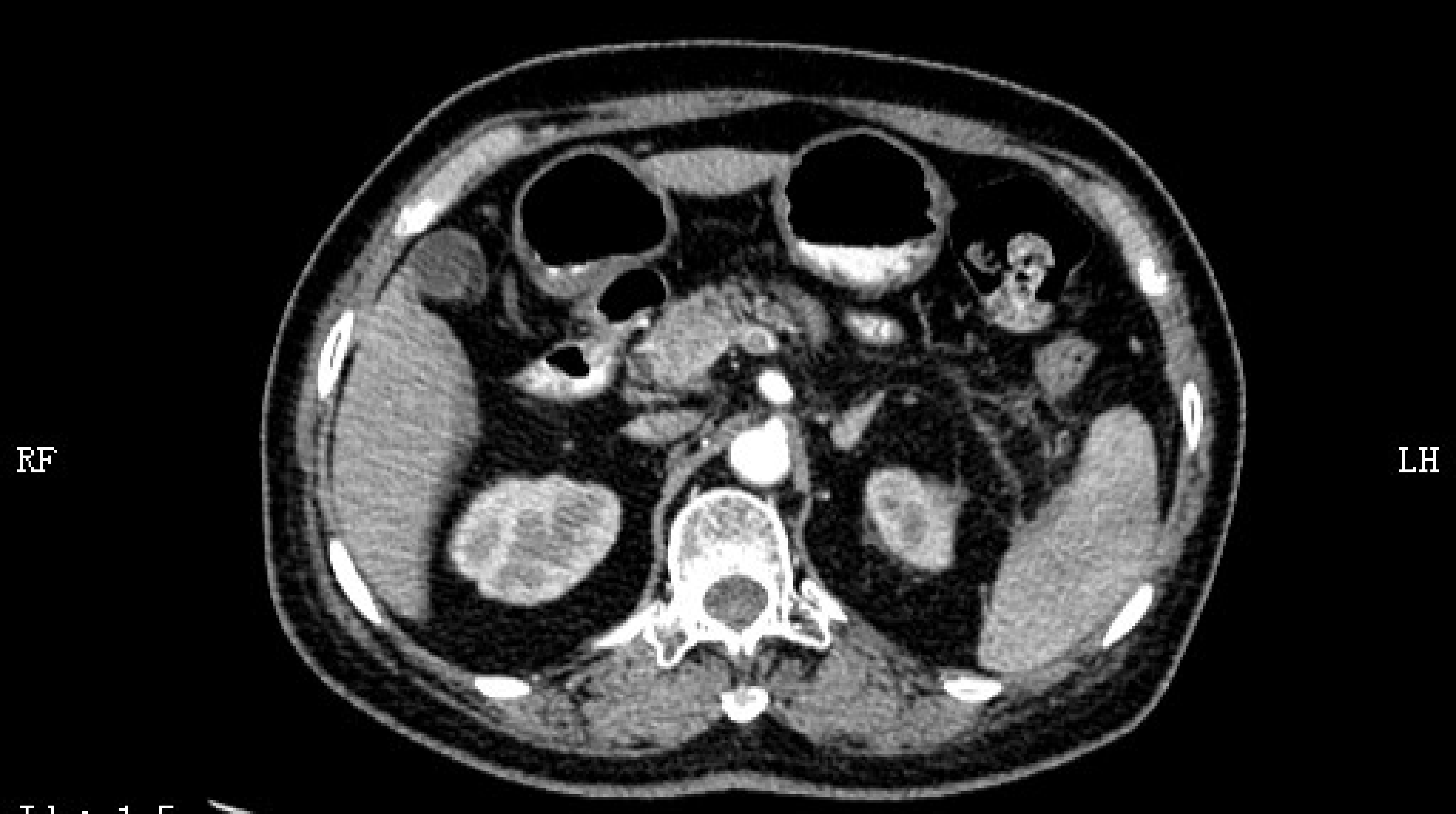
RF
LH

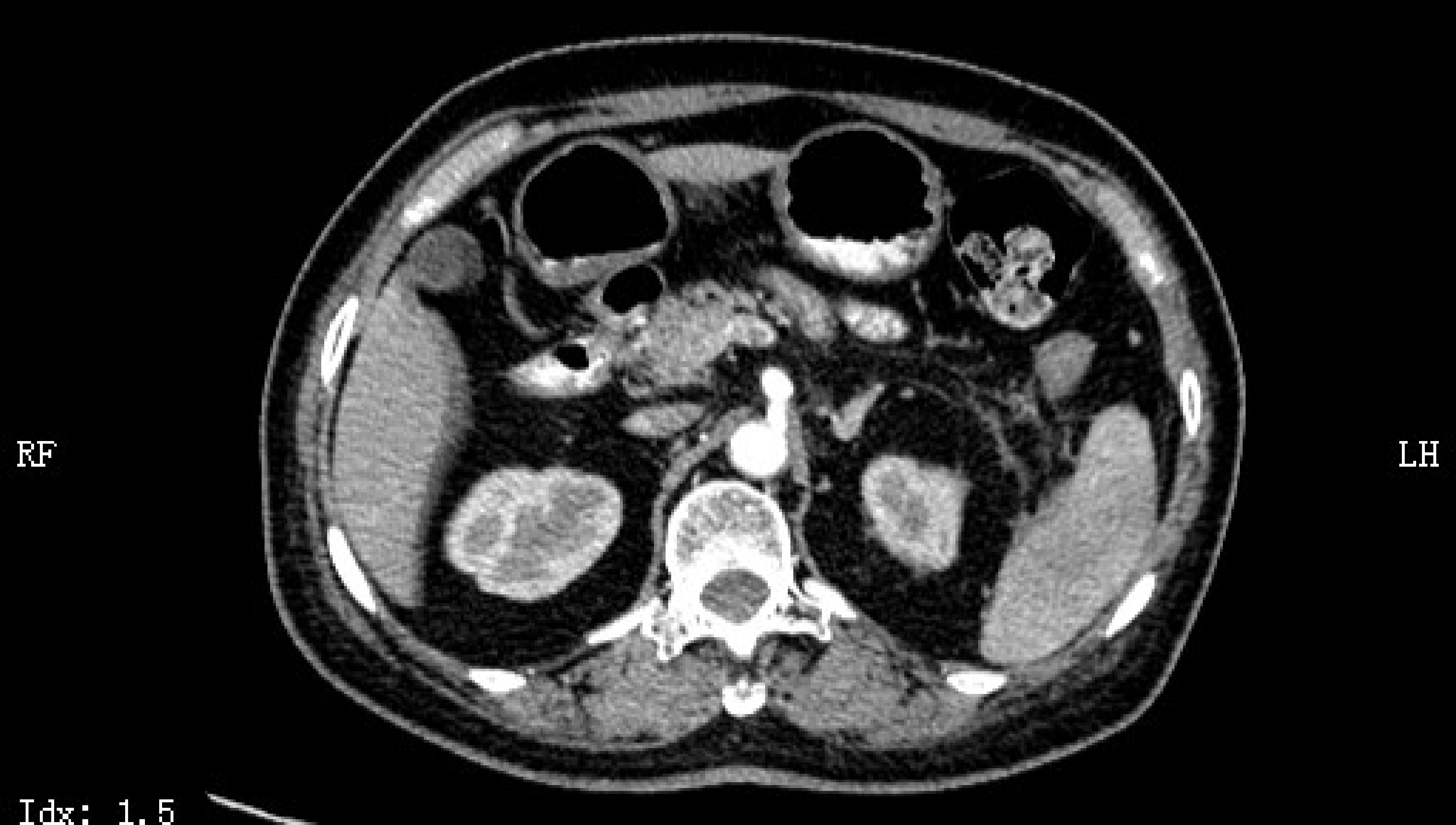
RF
LH
Idx: 1.5

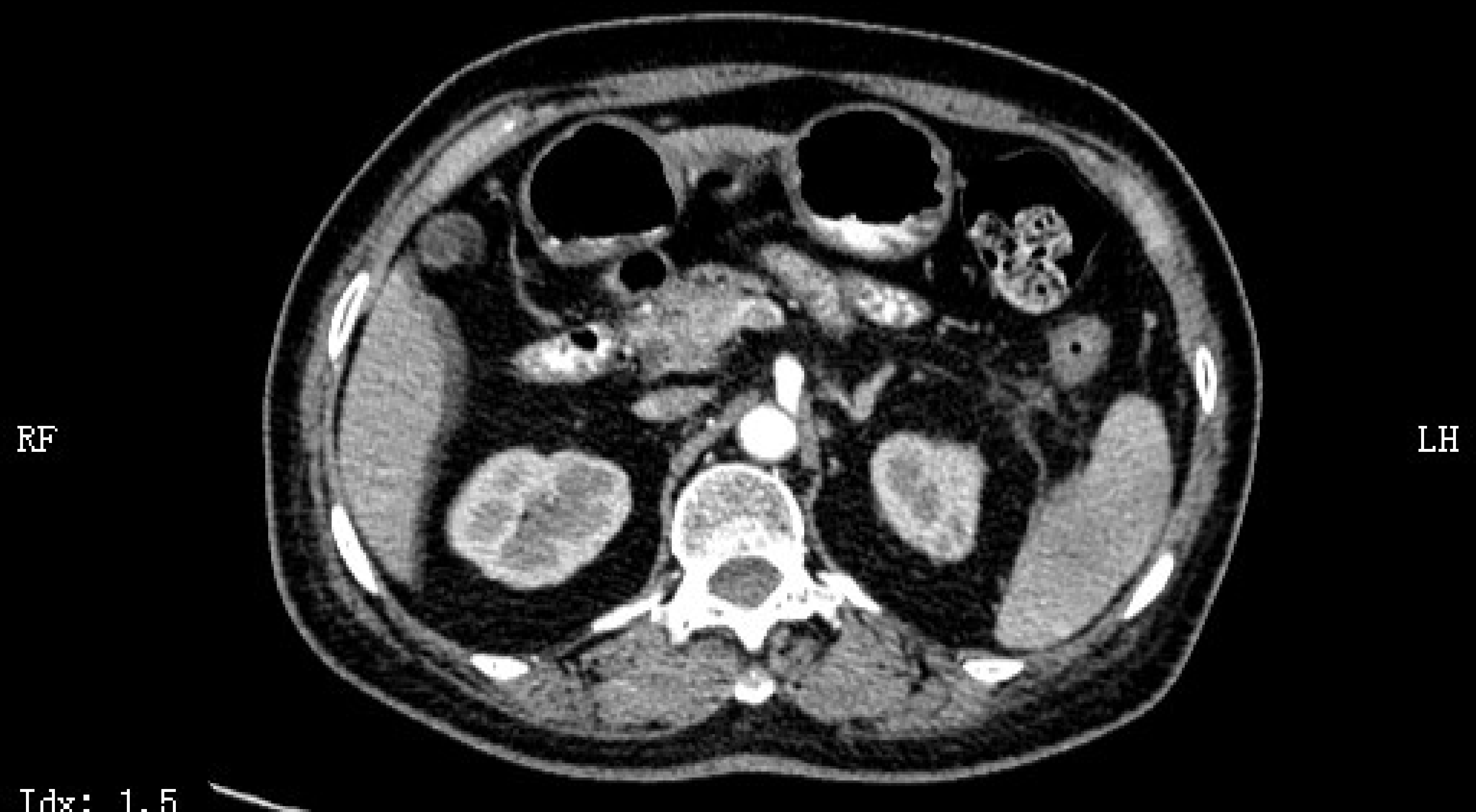
RF
LH
Idx: 1.5

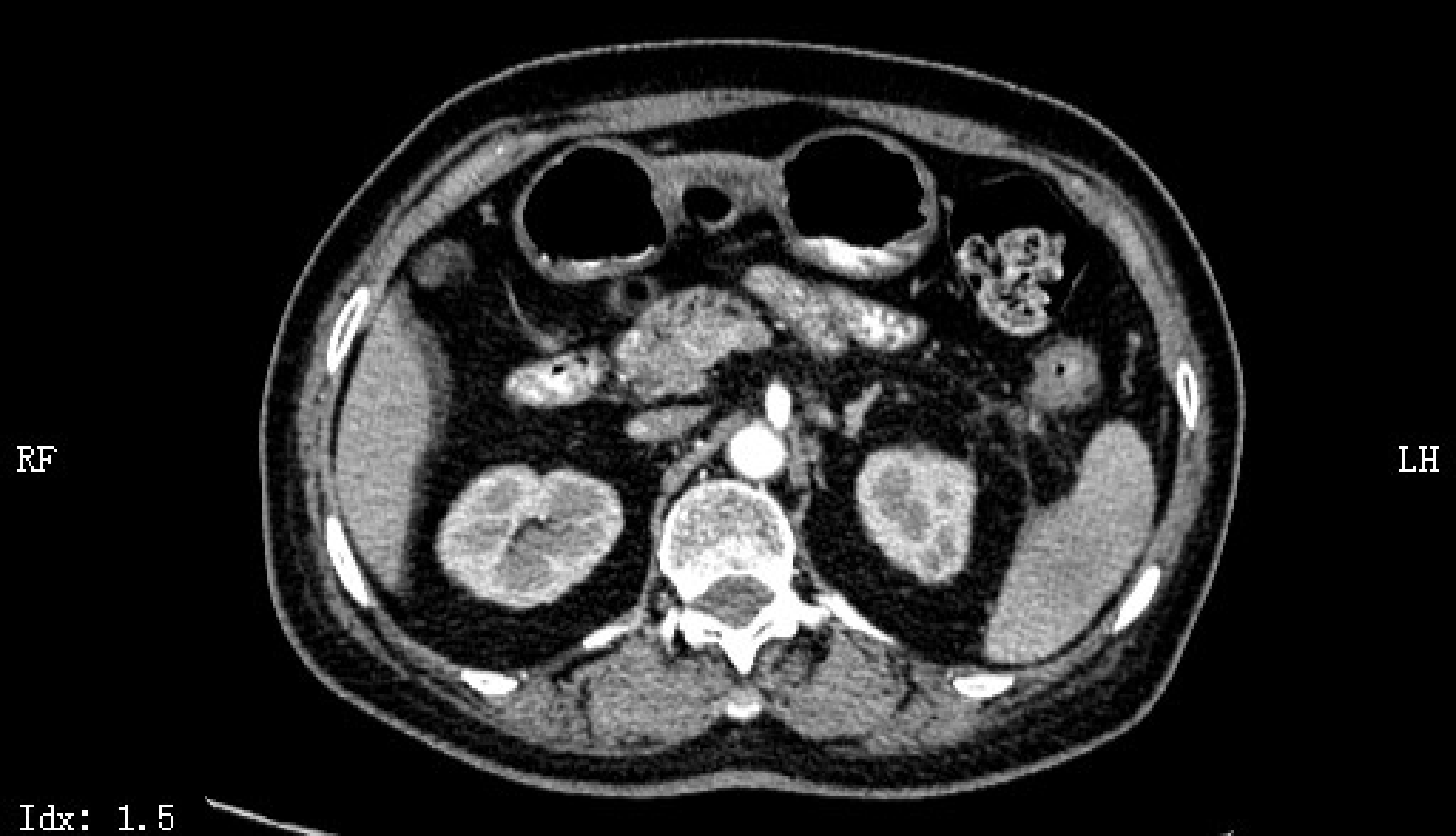

RF
LH
Idx: 1.5

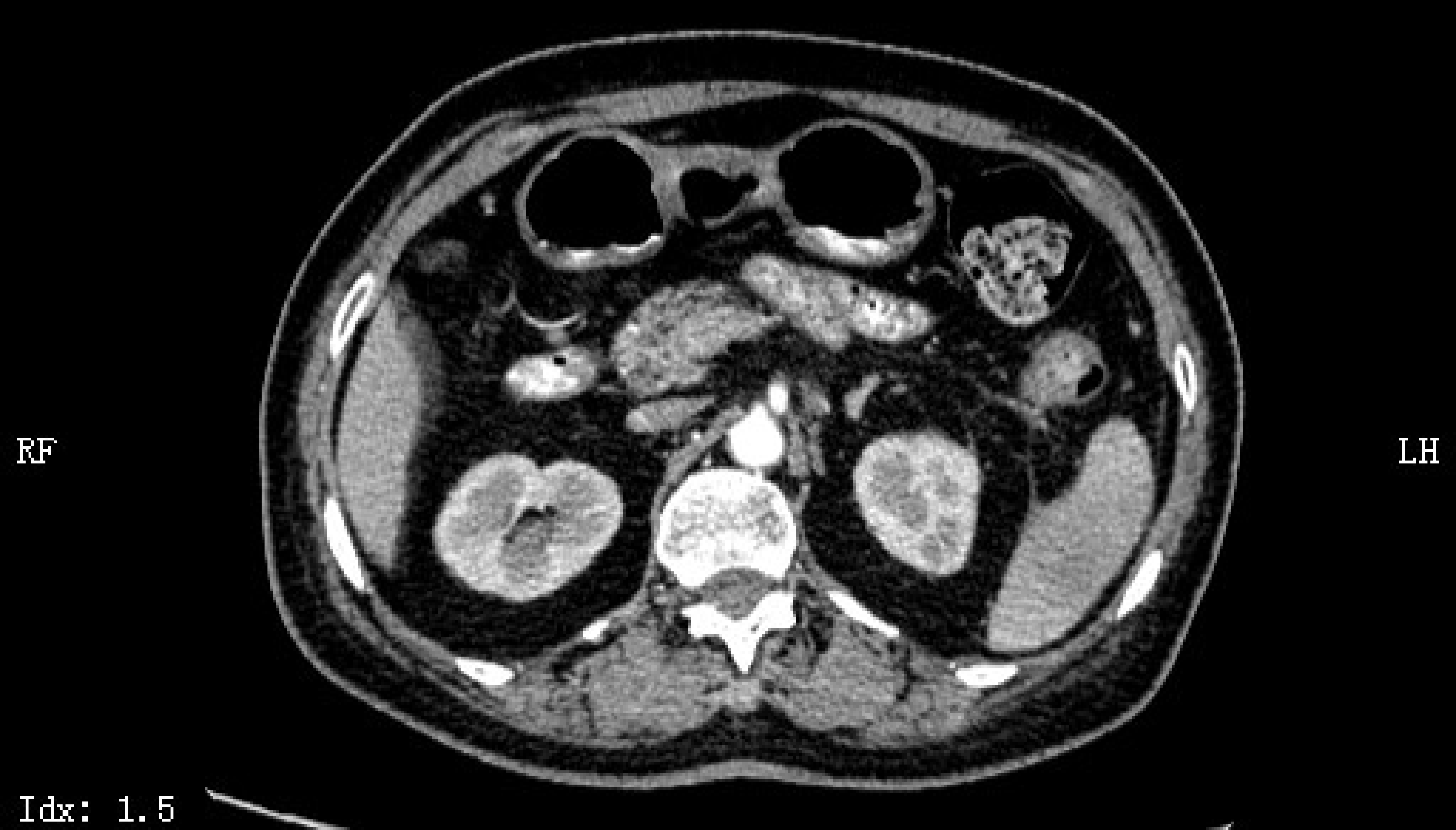
RF
LH
Idx: 1.5
