# Supplementary material for: Complete response to BRICS in Locally advanced pancreatic cancer (pMMR, CPS 30): a case report
Source: Front Immunol. 2026 Jan 21;17:1743752. doi: 10.3389/fimmu.2026.1743752 (PMC12867830; doi:10.3389/fimmu.2026.1743752)
Supplement: Supplementary Figure 4 — Contrast-enhanced CT image of the tumor lesion (March 26, 2025). [file DataSheet4.pdf]

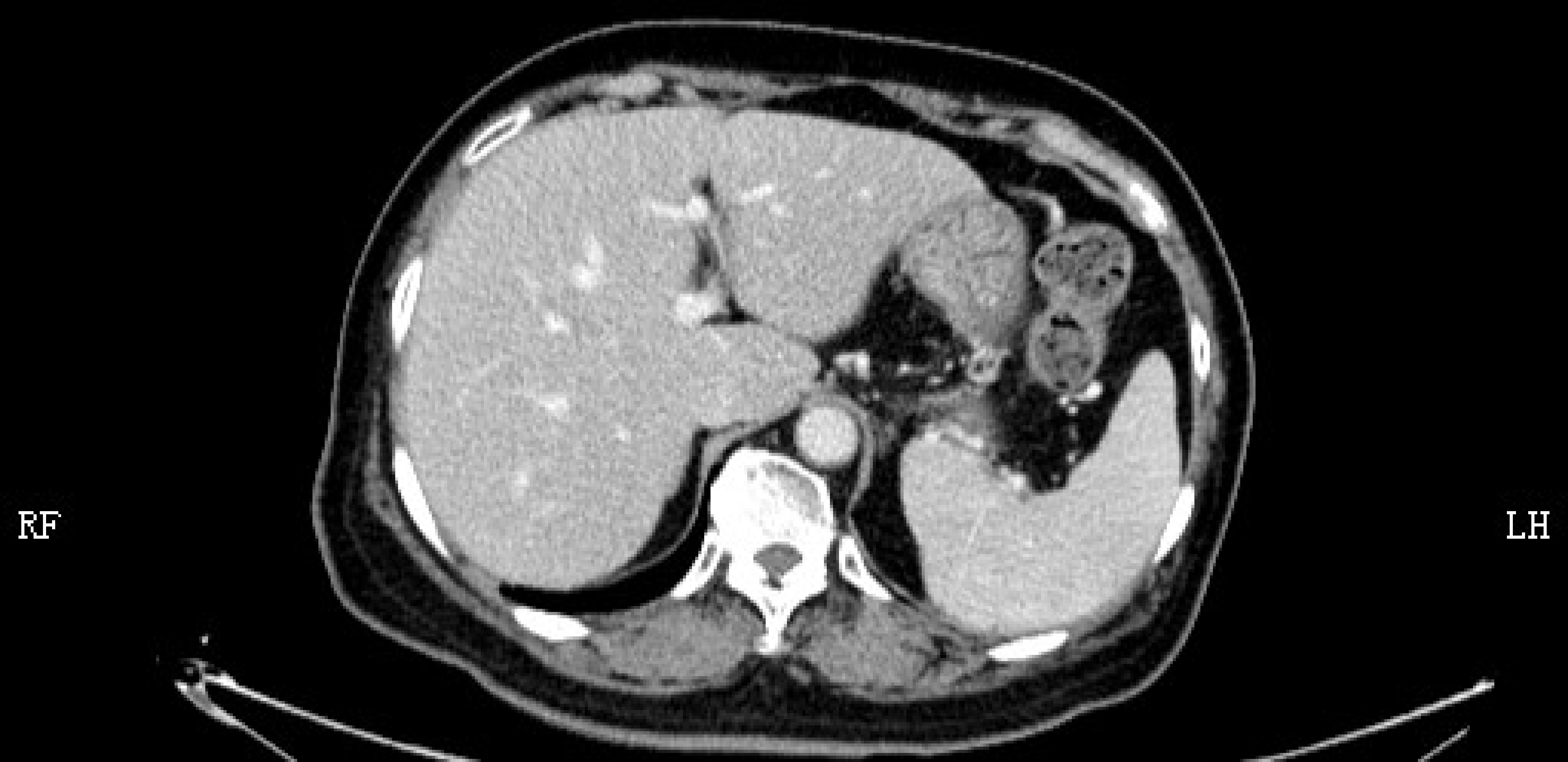
RF
LH

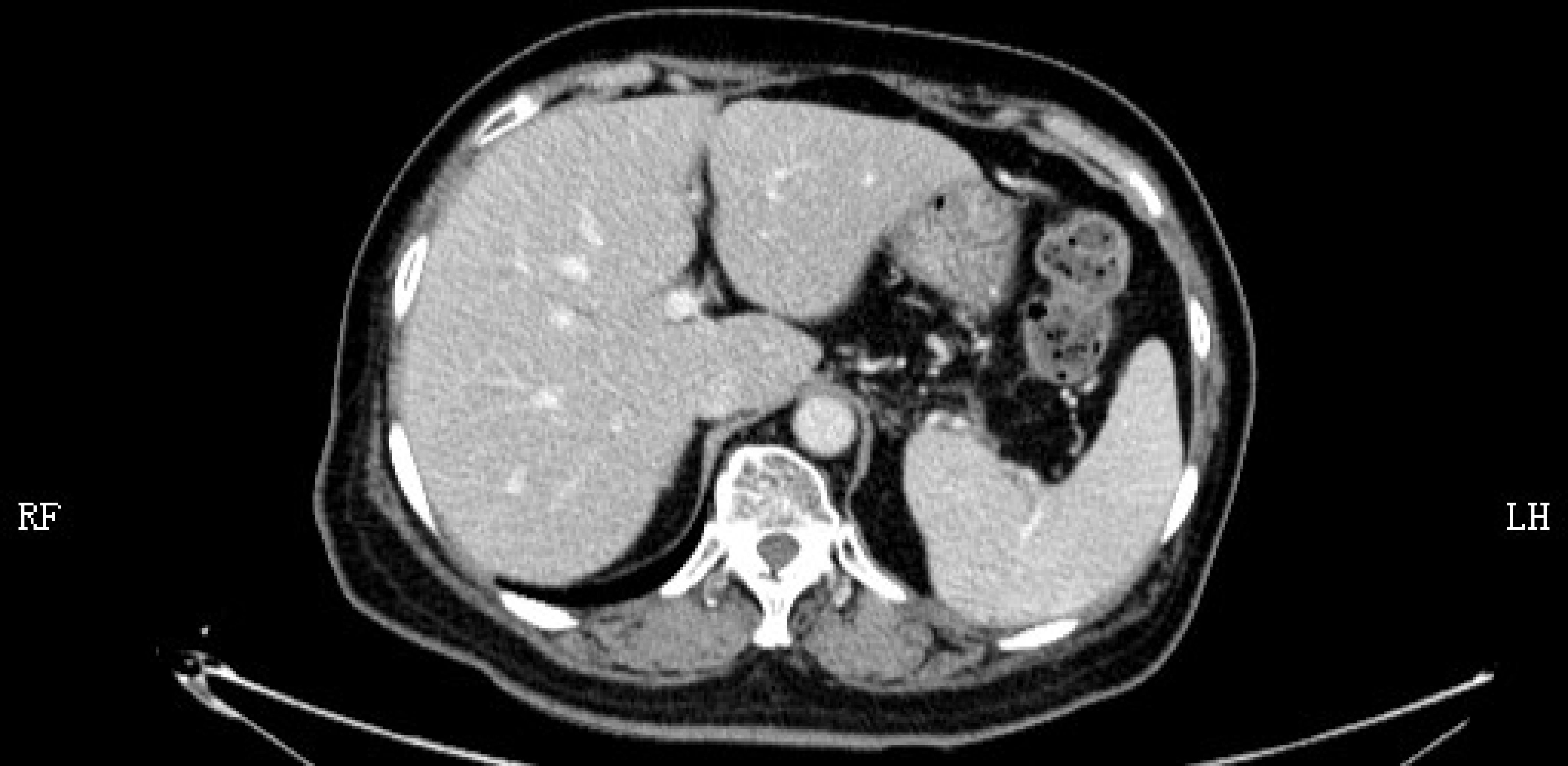
RF
LH

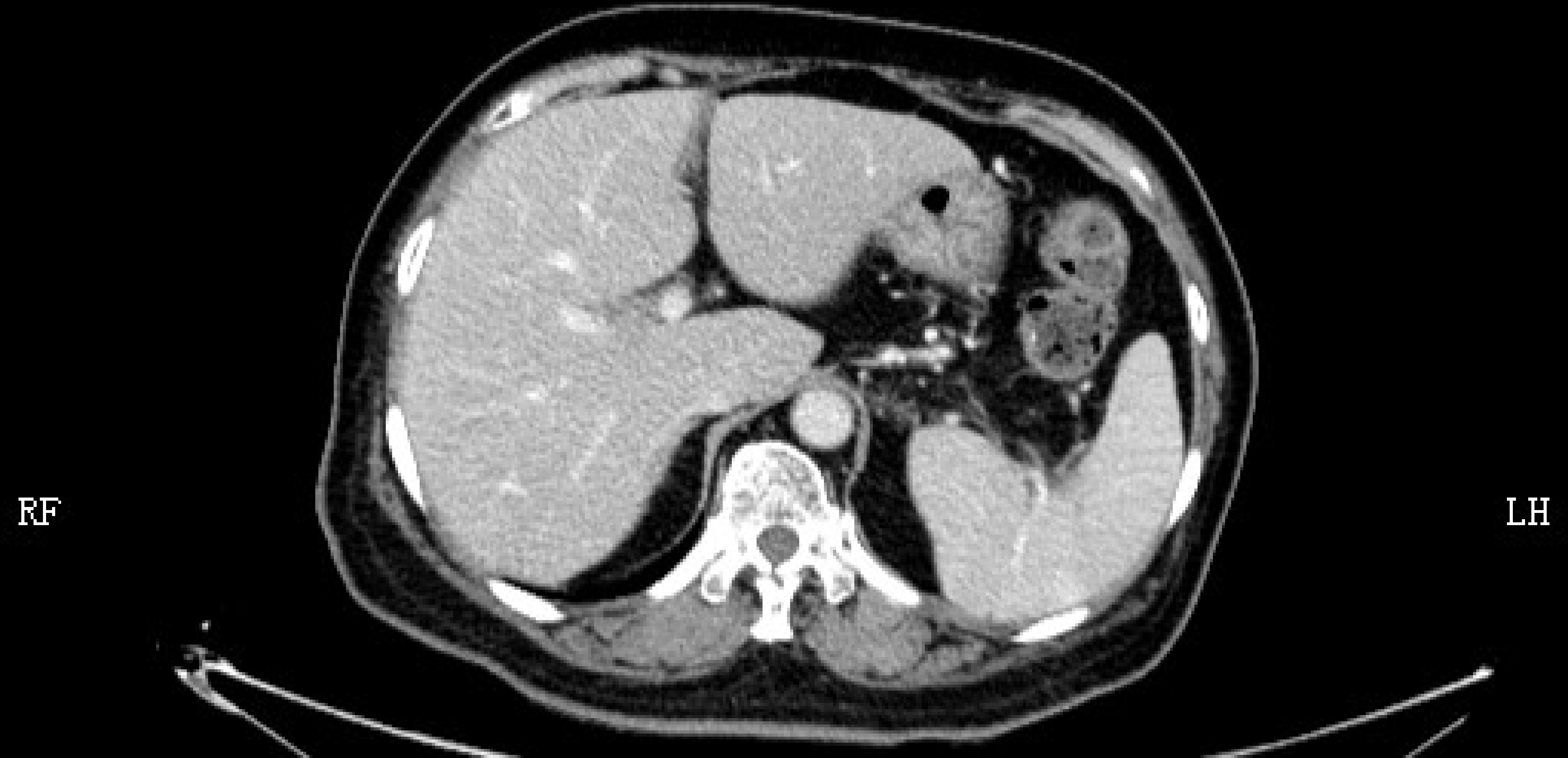
RF
LH

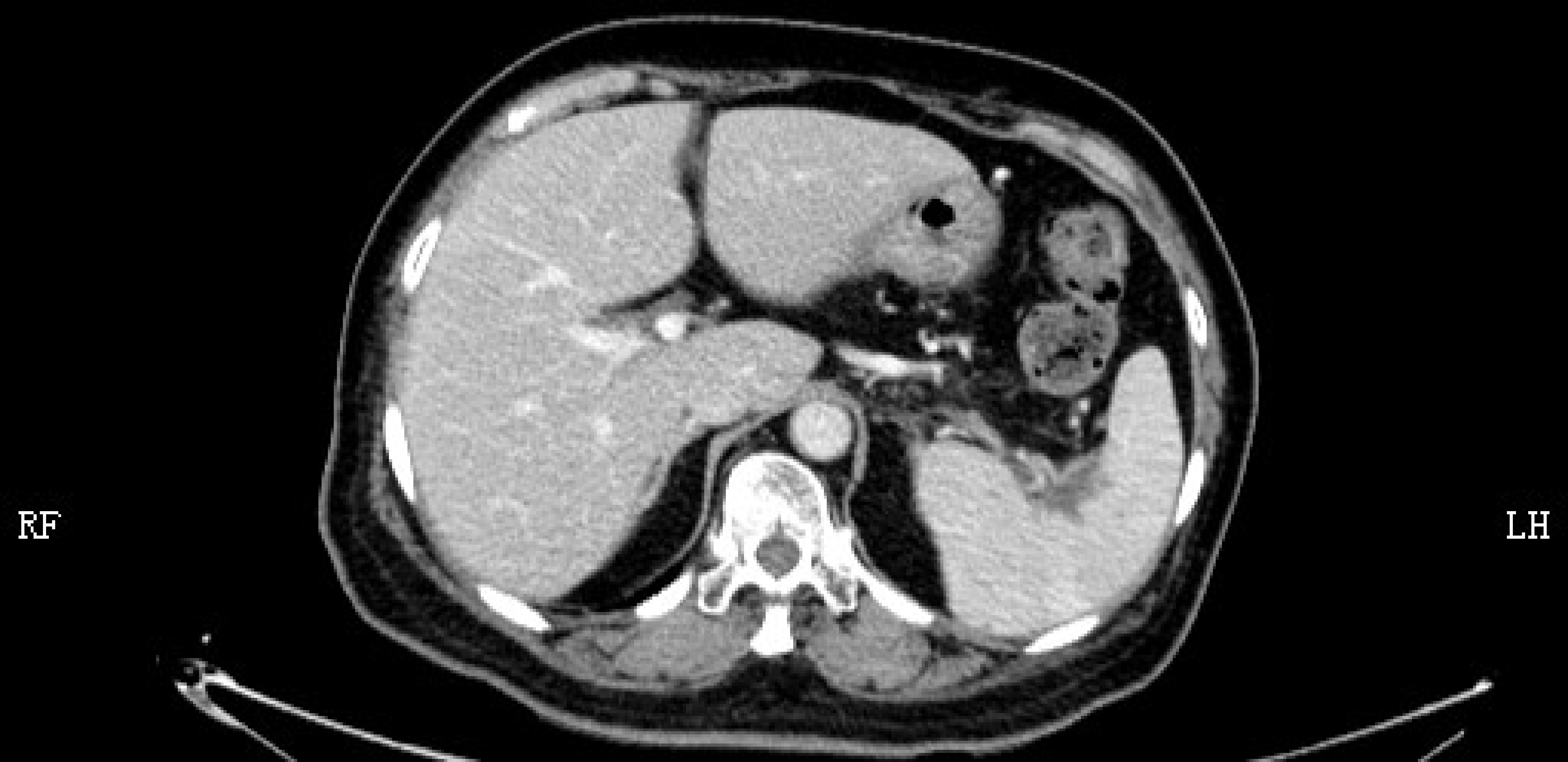
RF
LH

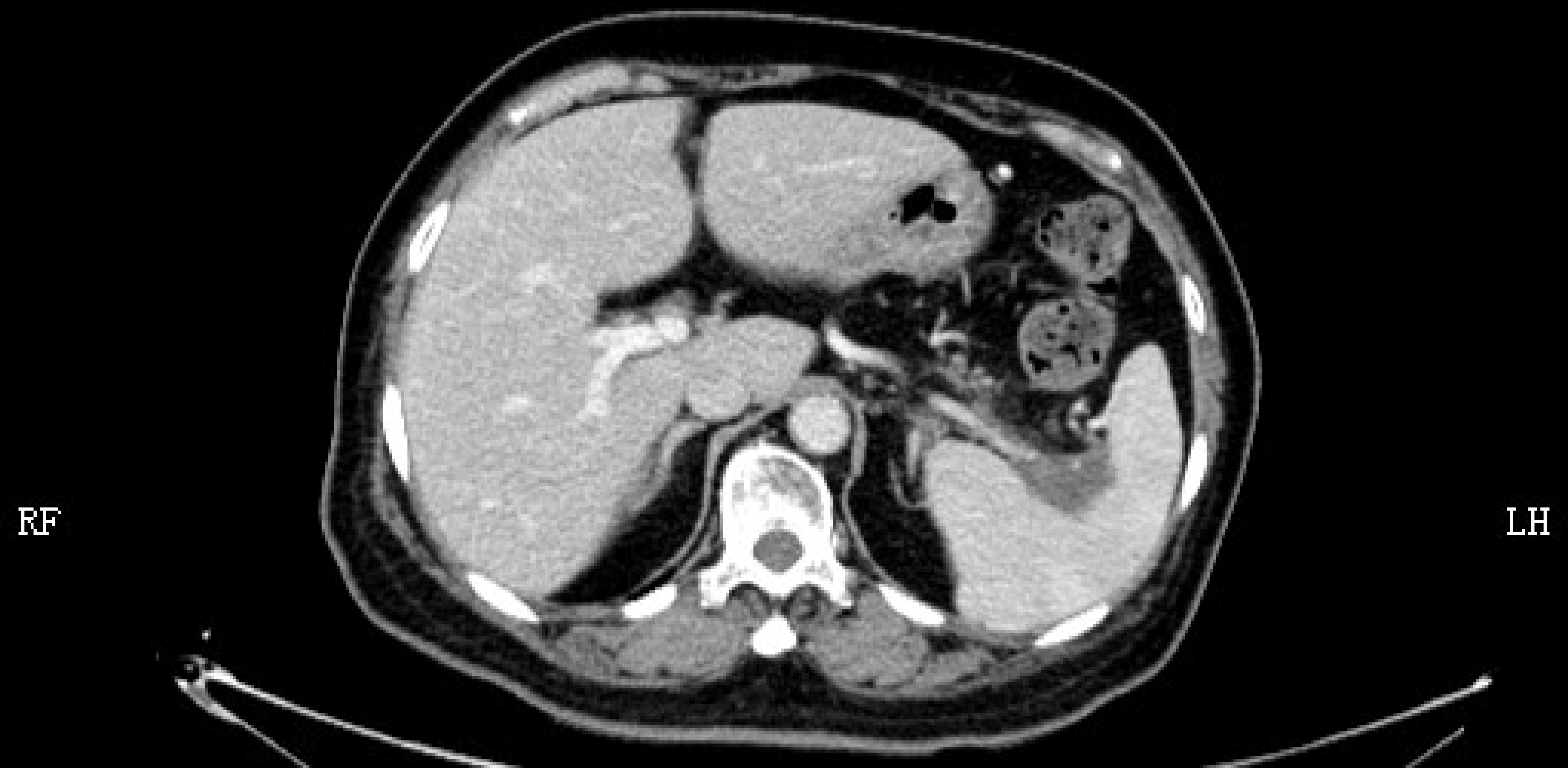
RF
LH

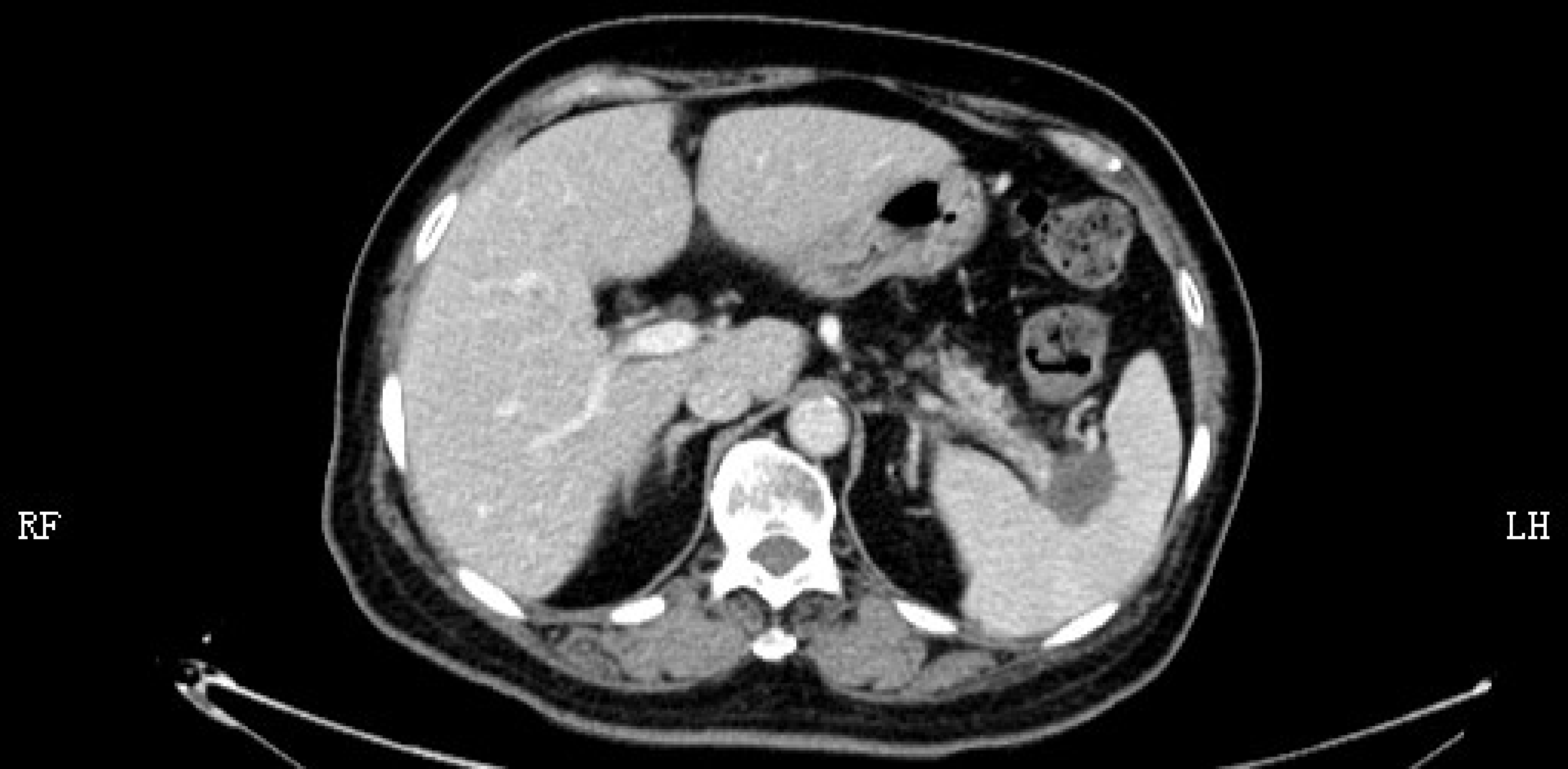
RF
LH

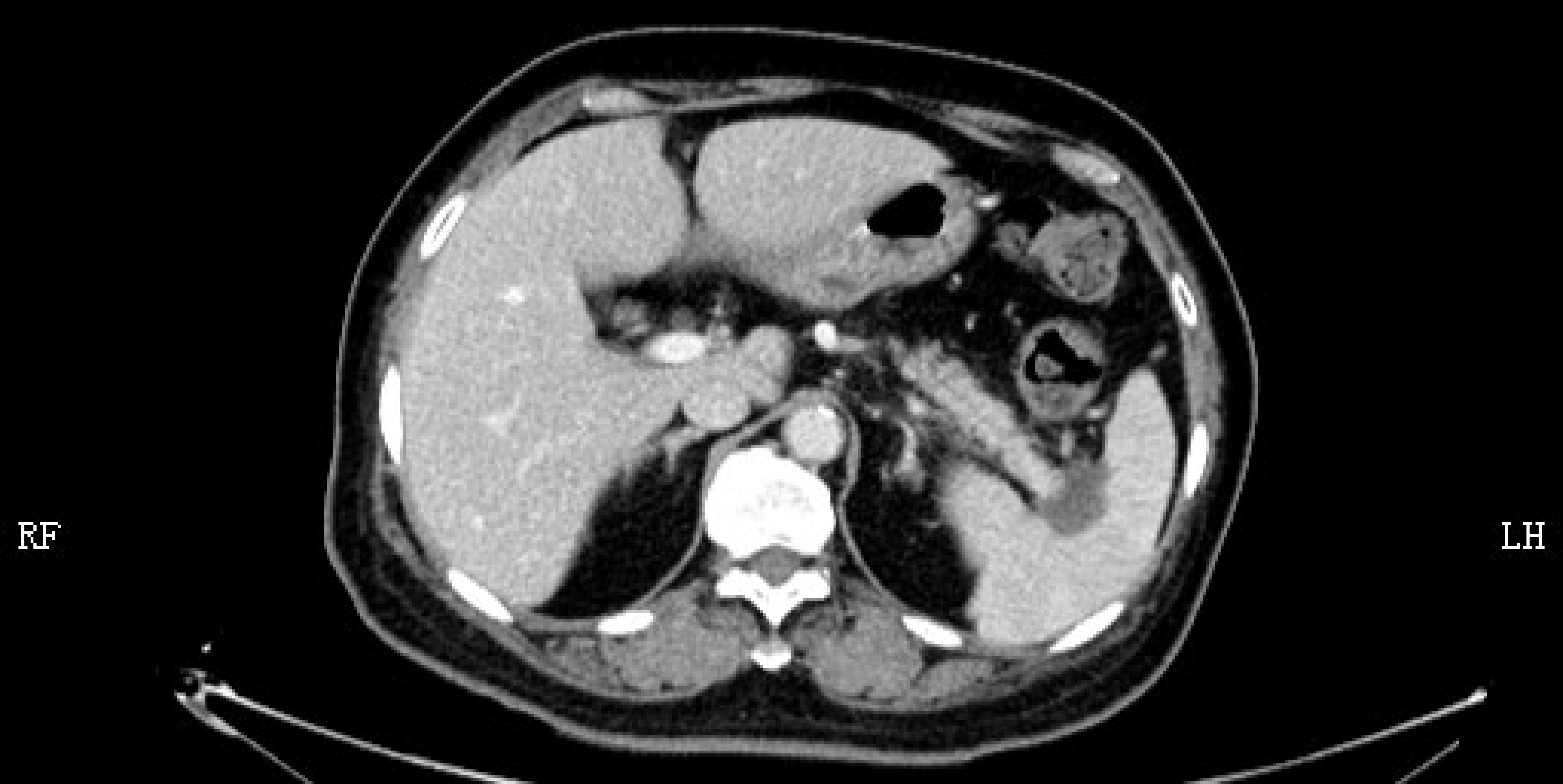
RF
LH

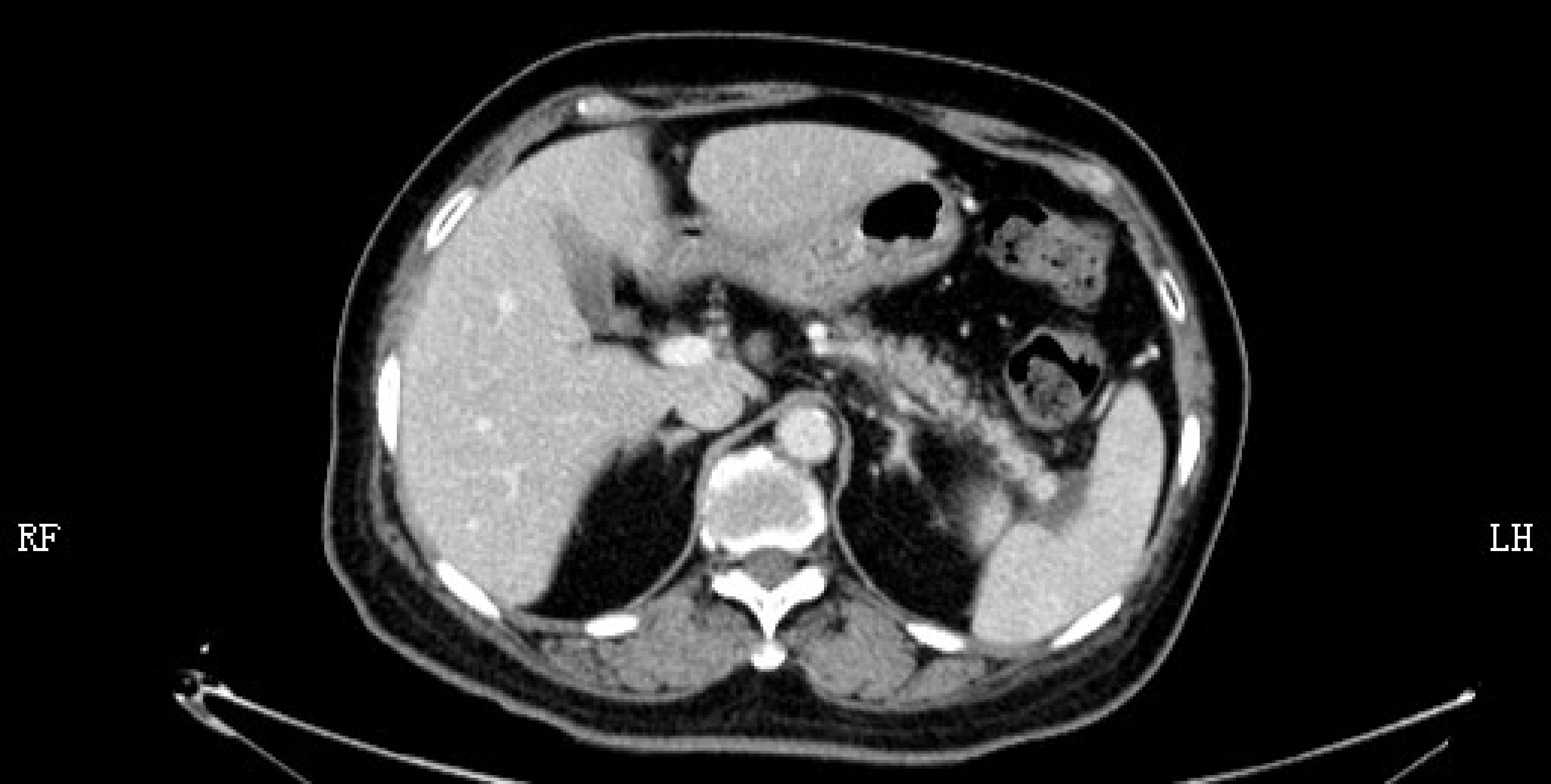
RF
LH

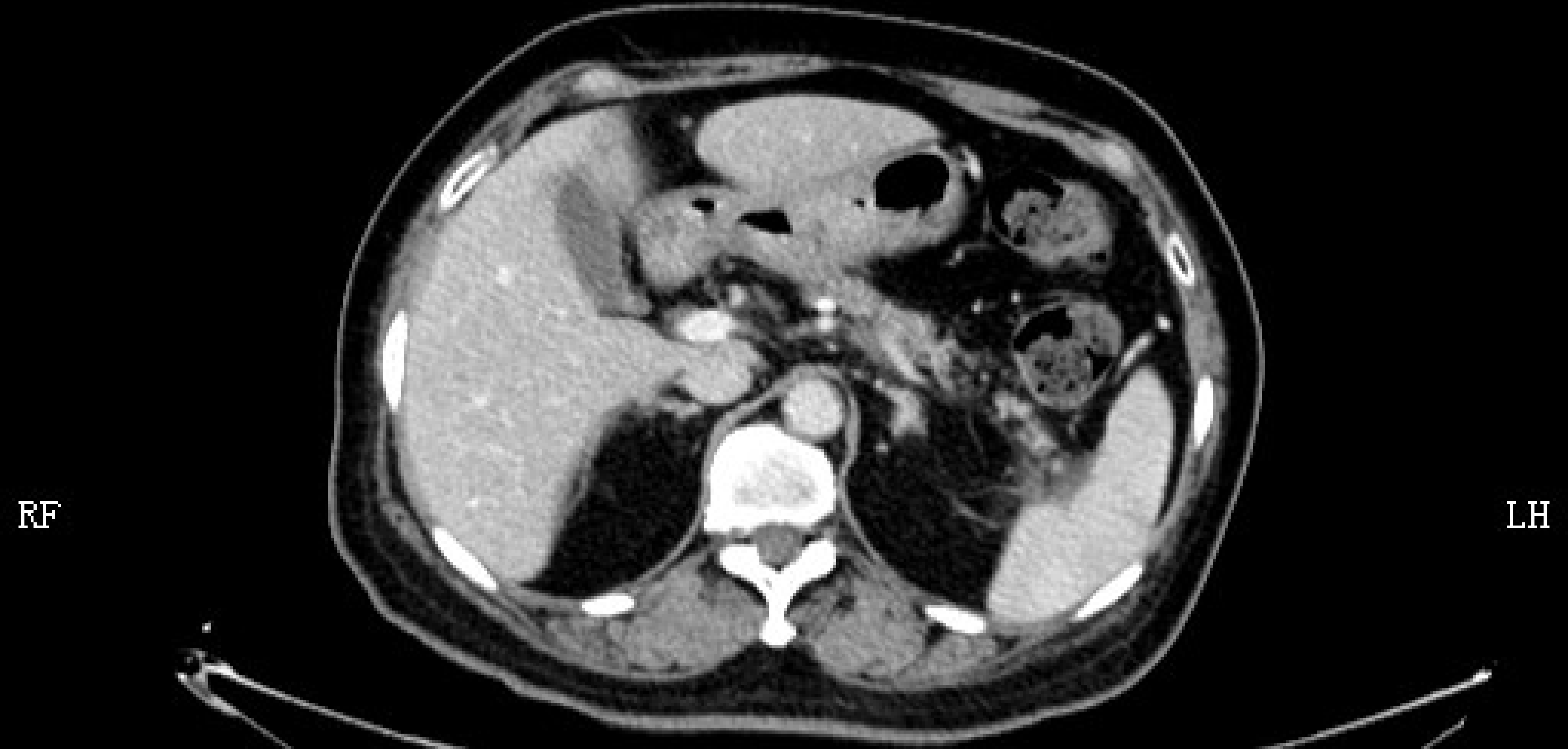
RF
LH

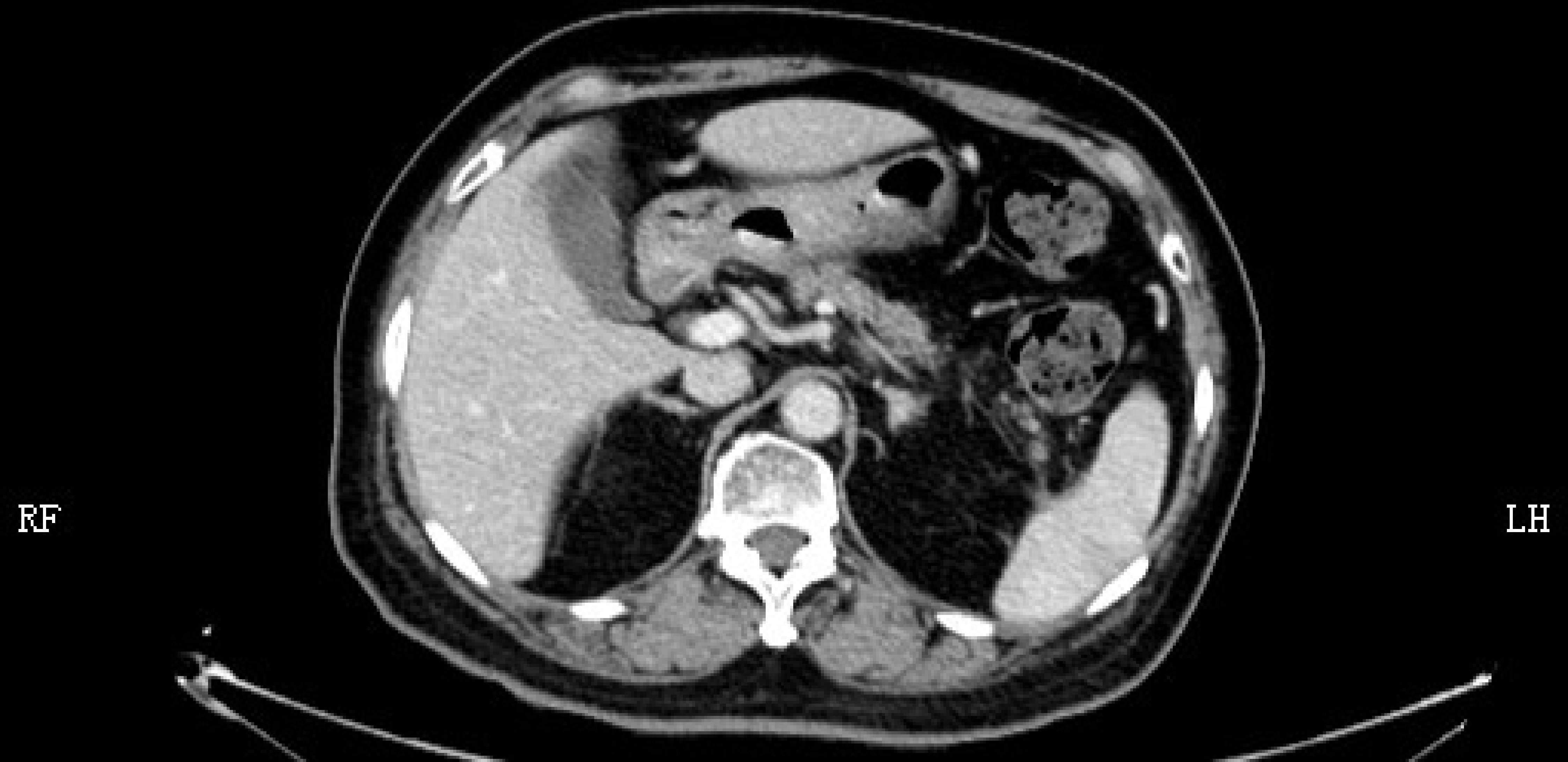

RF
LH

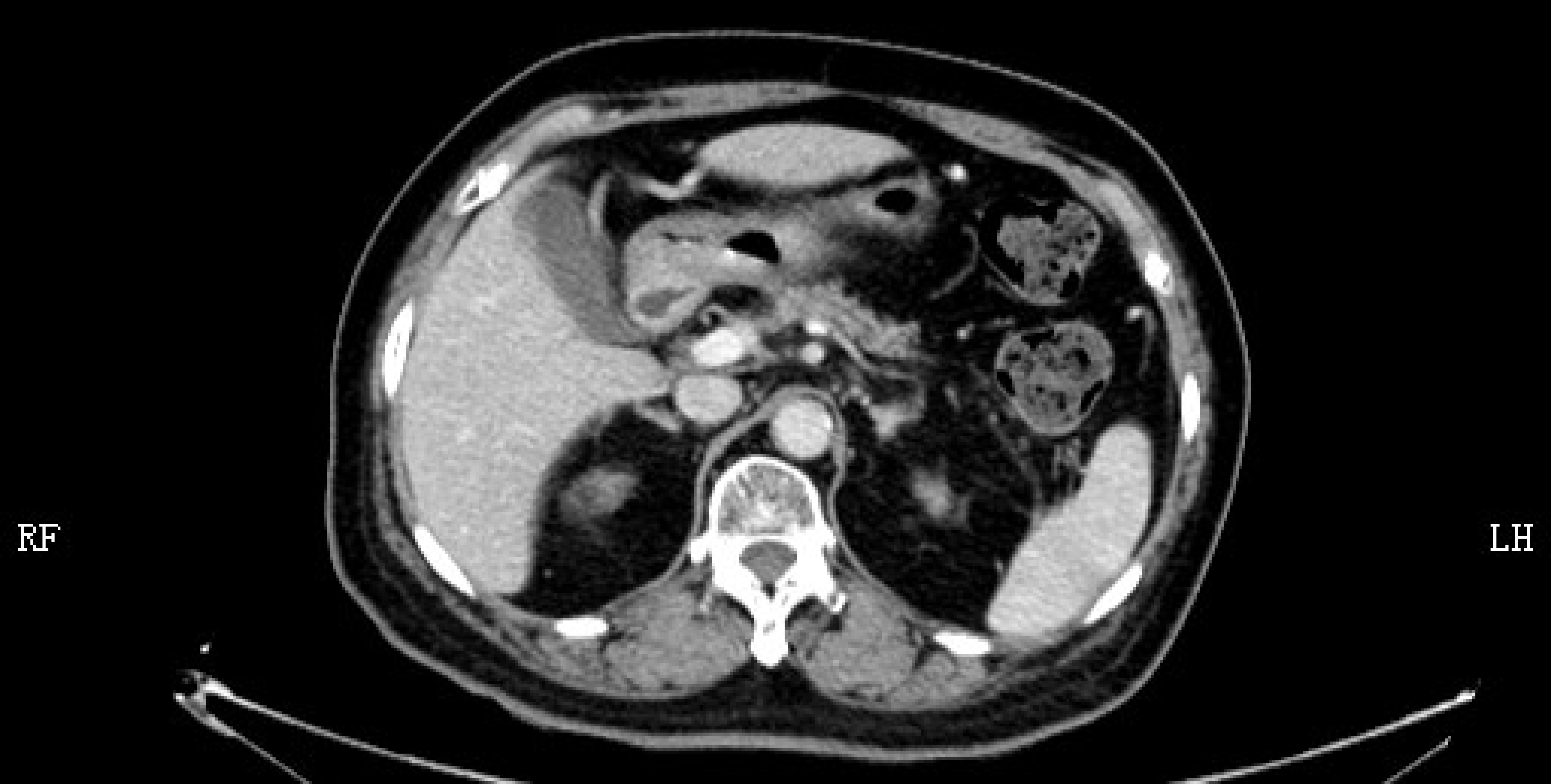

RF
LH

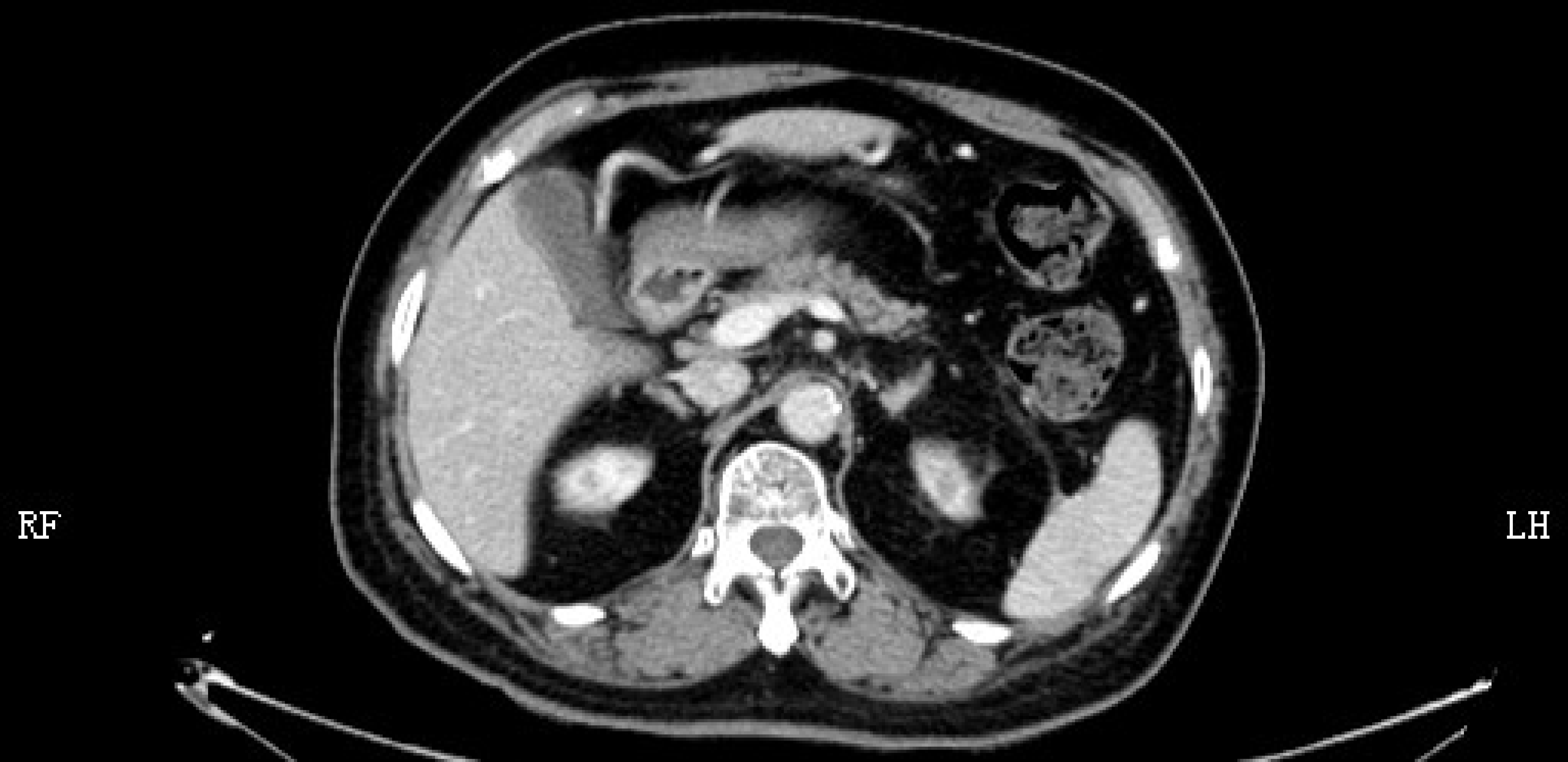
RF
LH

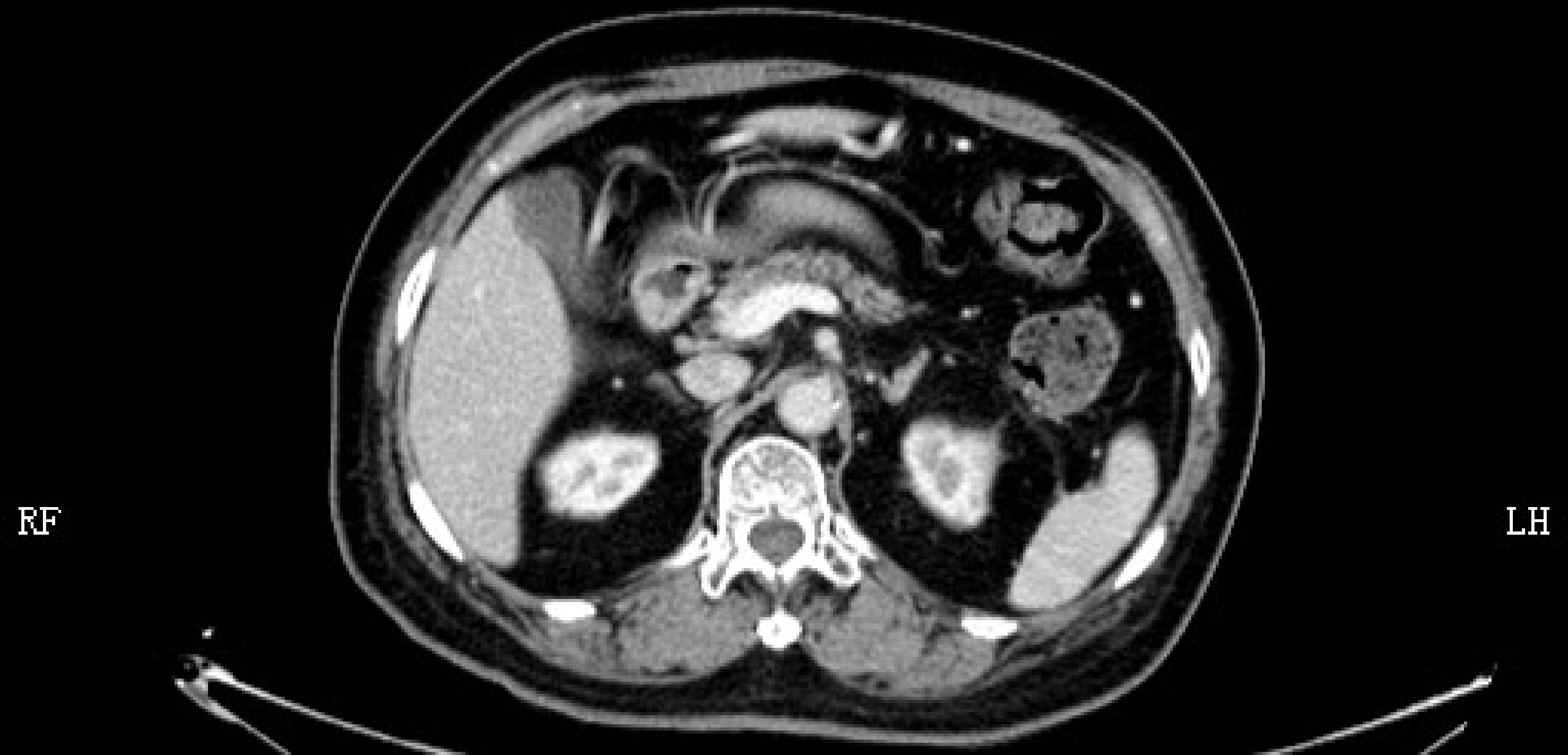
RF
LH

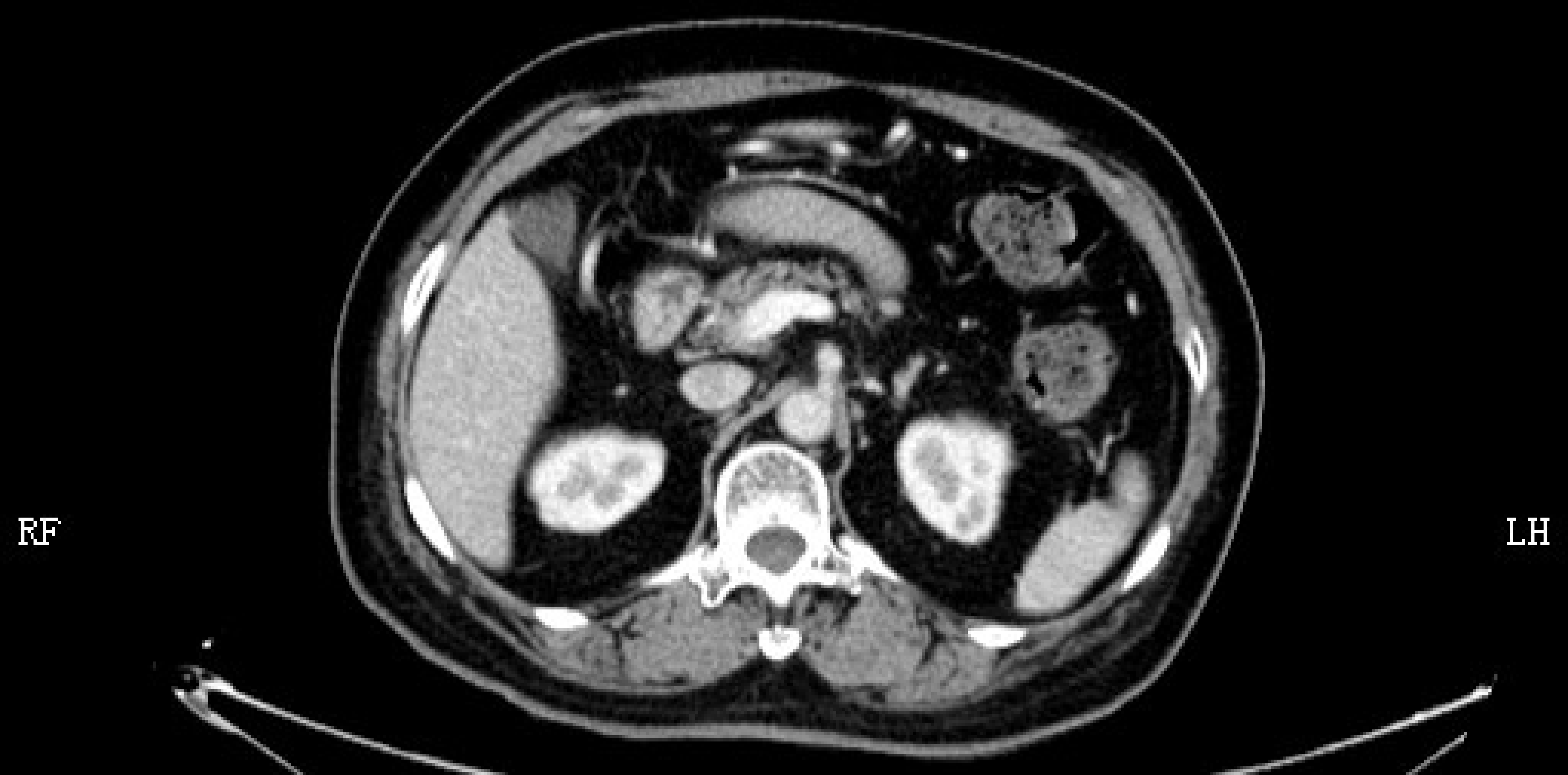

RF
LH

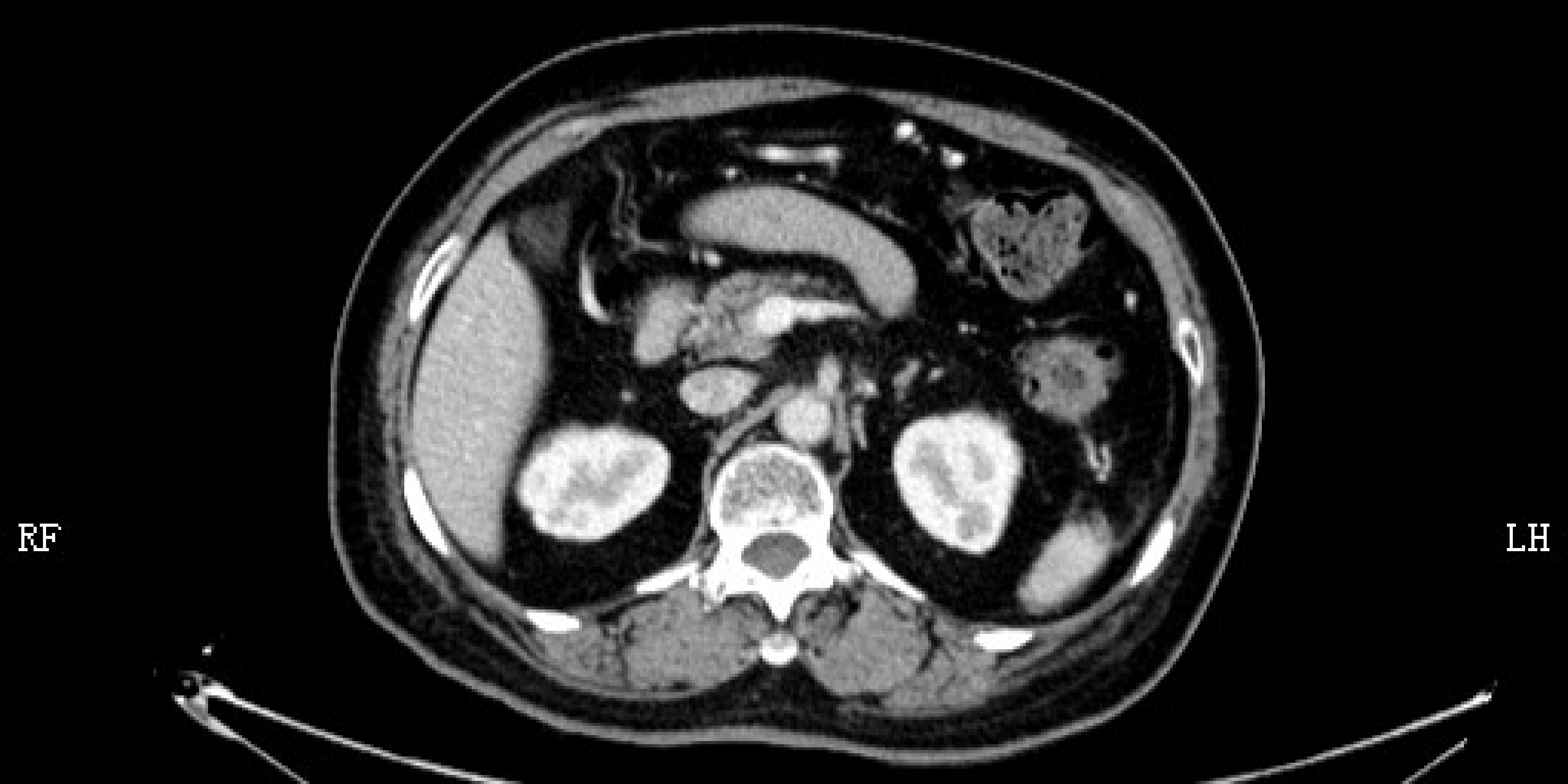
RF
LH

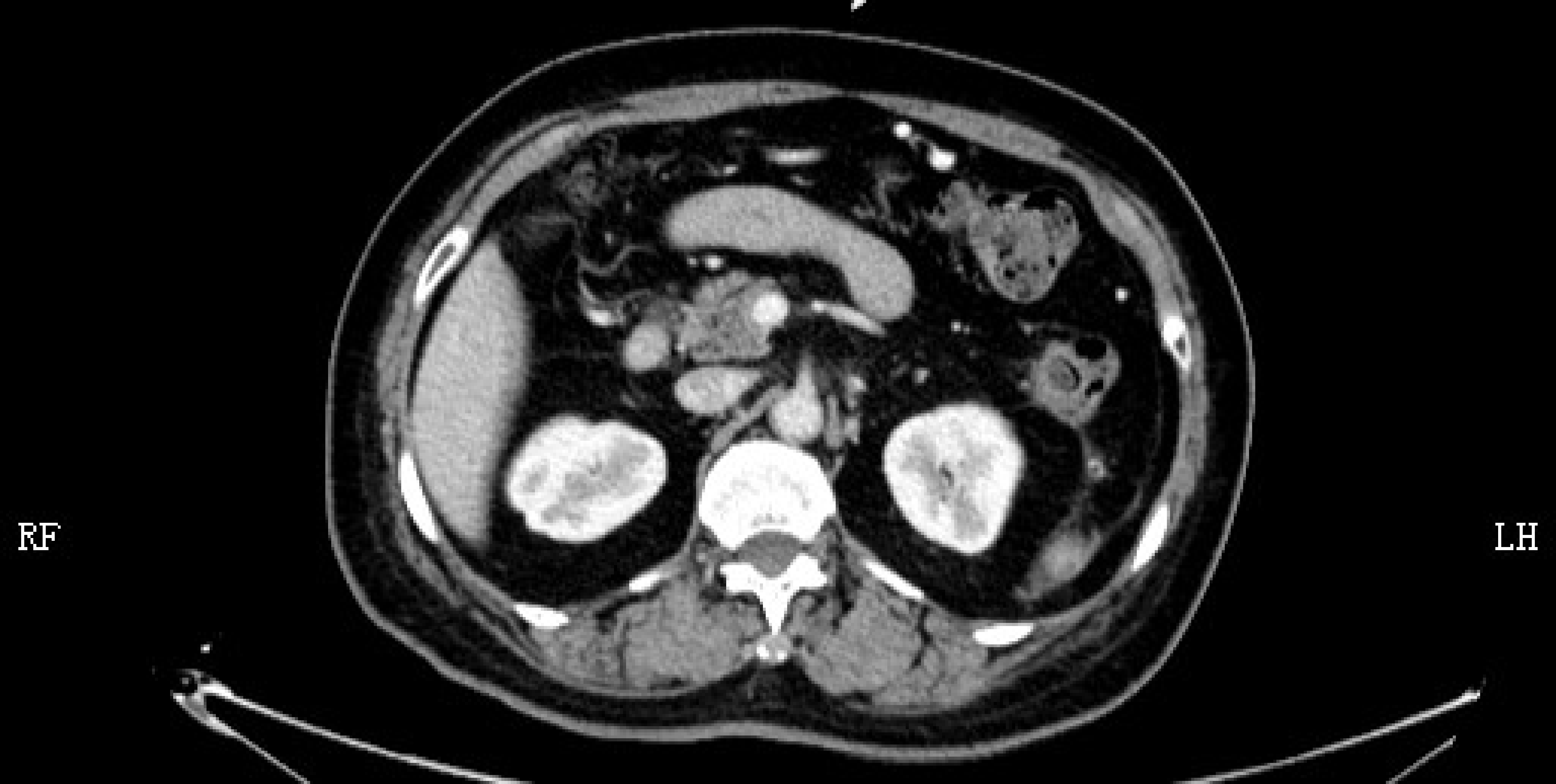
RF
LH

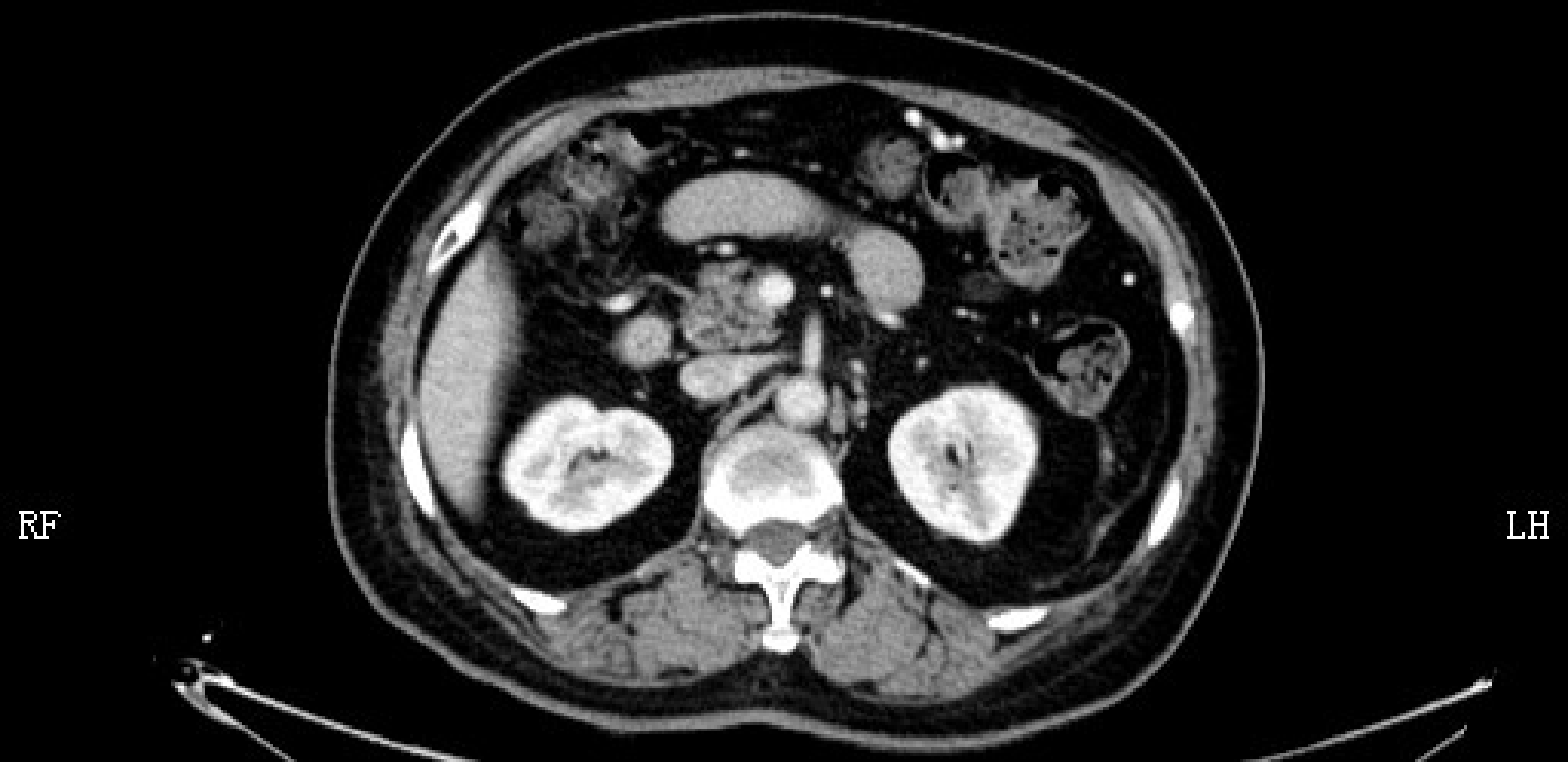
RF
LH

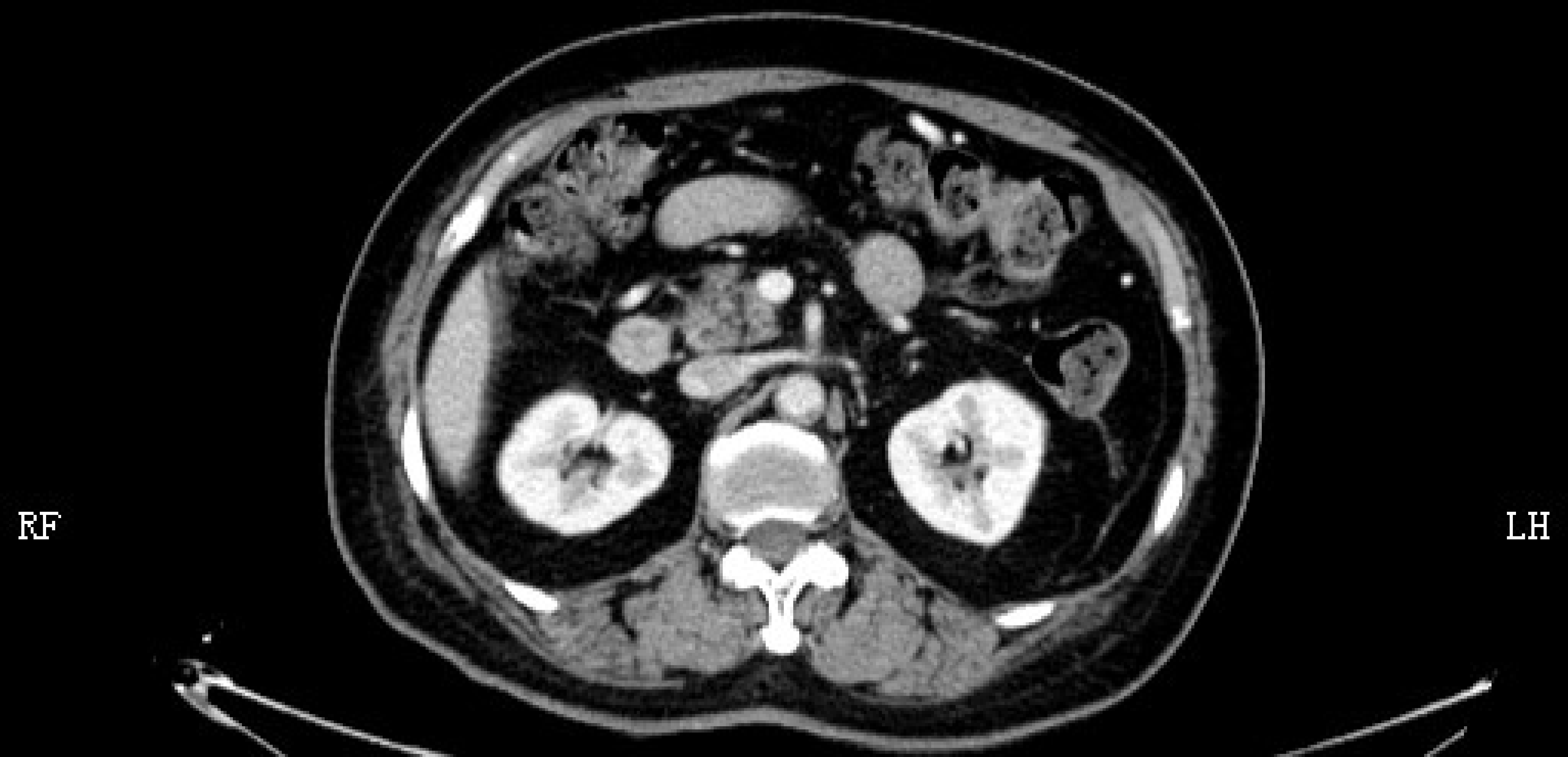
RF
LH

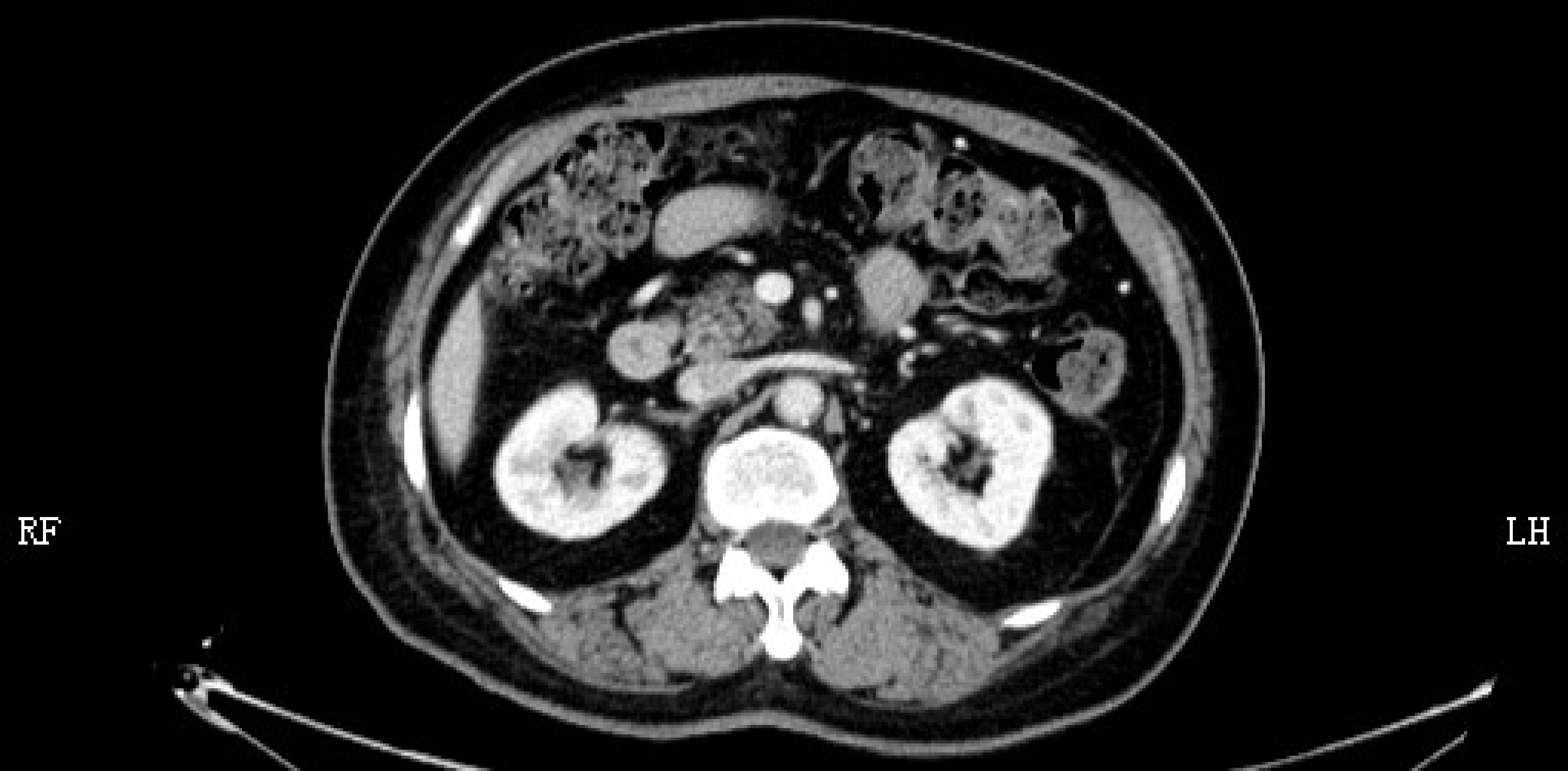
RF
LH

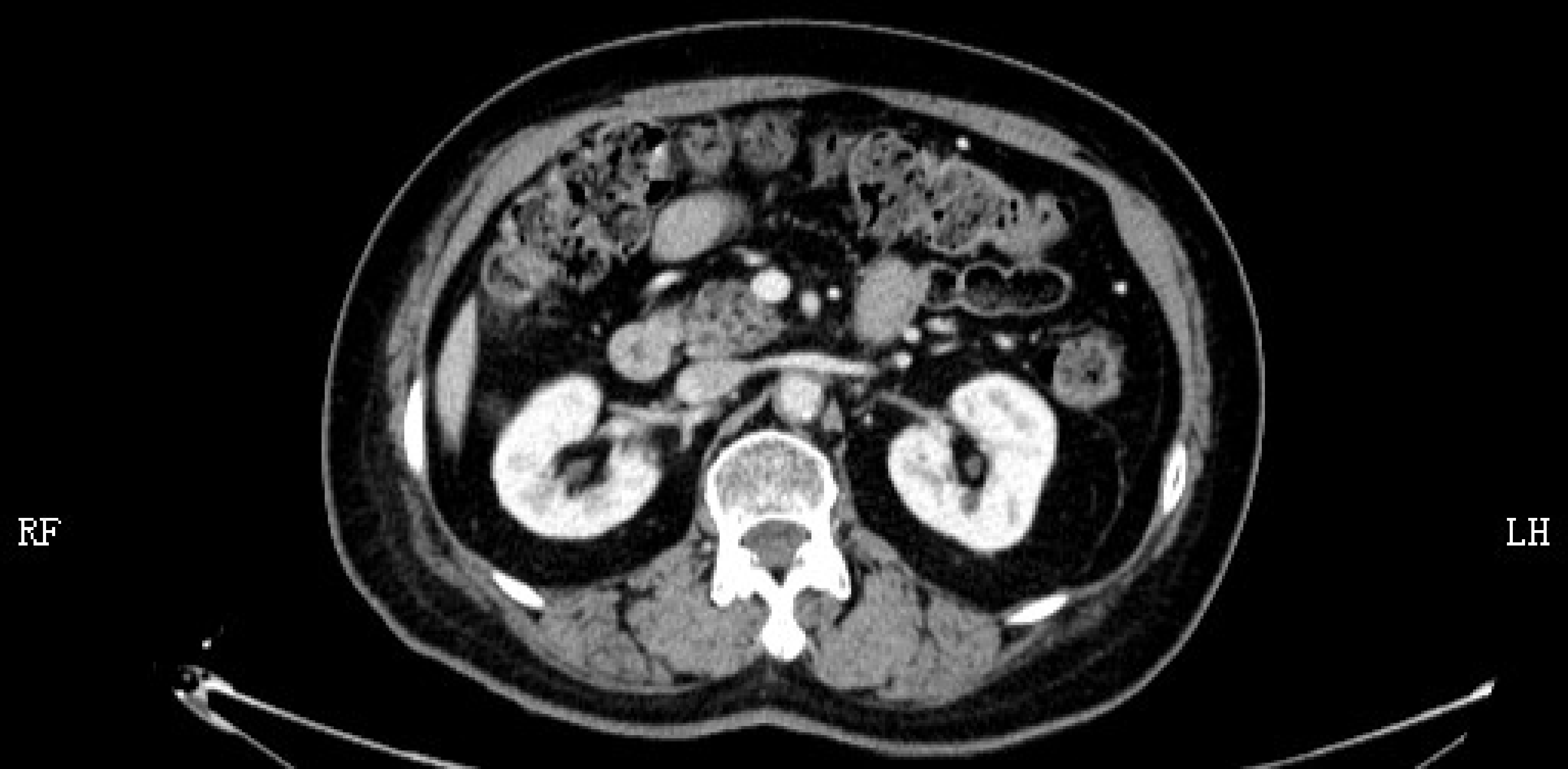
RF
LH
