# Supplementary material for: Complete response to BRICS in Locally advanced pancreatic cancer (pMMR, CPS 30): a case report
Source: Front Immunol. 2026 Jan 21;17:1743752. doi: 10.3389/fimmu.2026.1743752 (PMC12867830; doi:10.3389/fimmu.2026.1743752)
Supplement: Supplementary Figure 5 — Contrast-enhanced CT image of the tumor lesion (August 19, 2025). [file DataSheet5.pdf]

CA199_10.00X

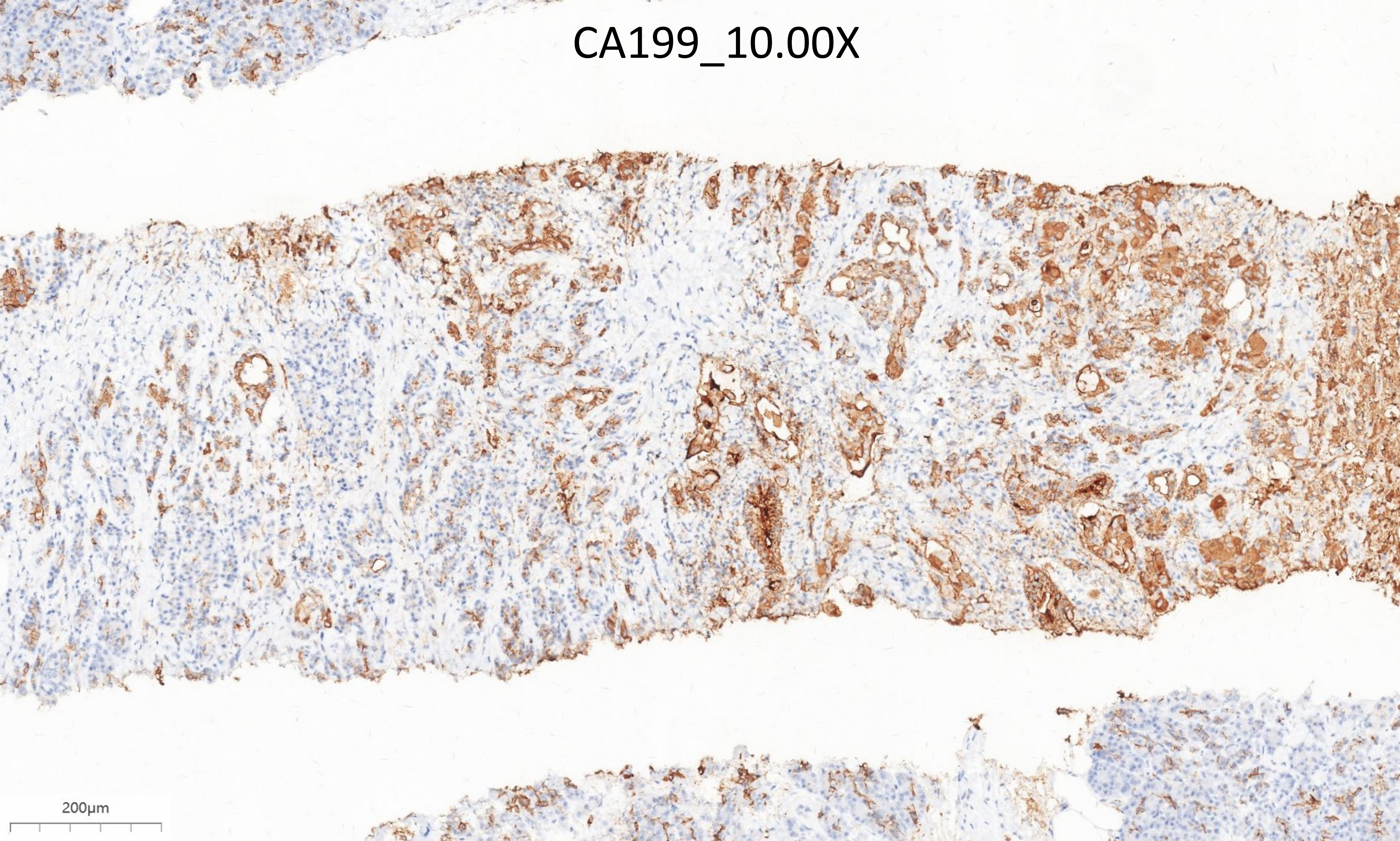

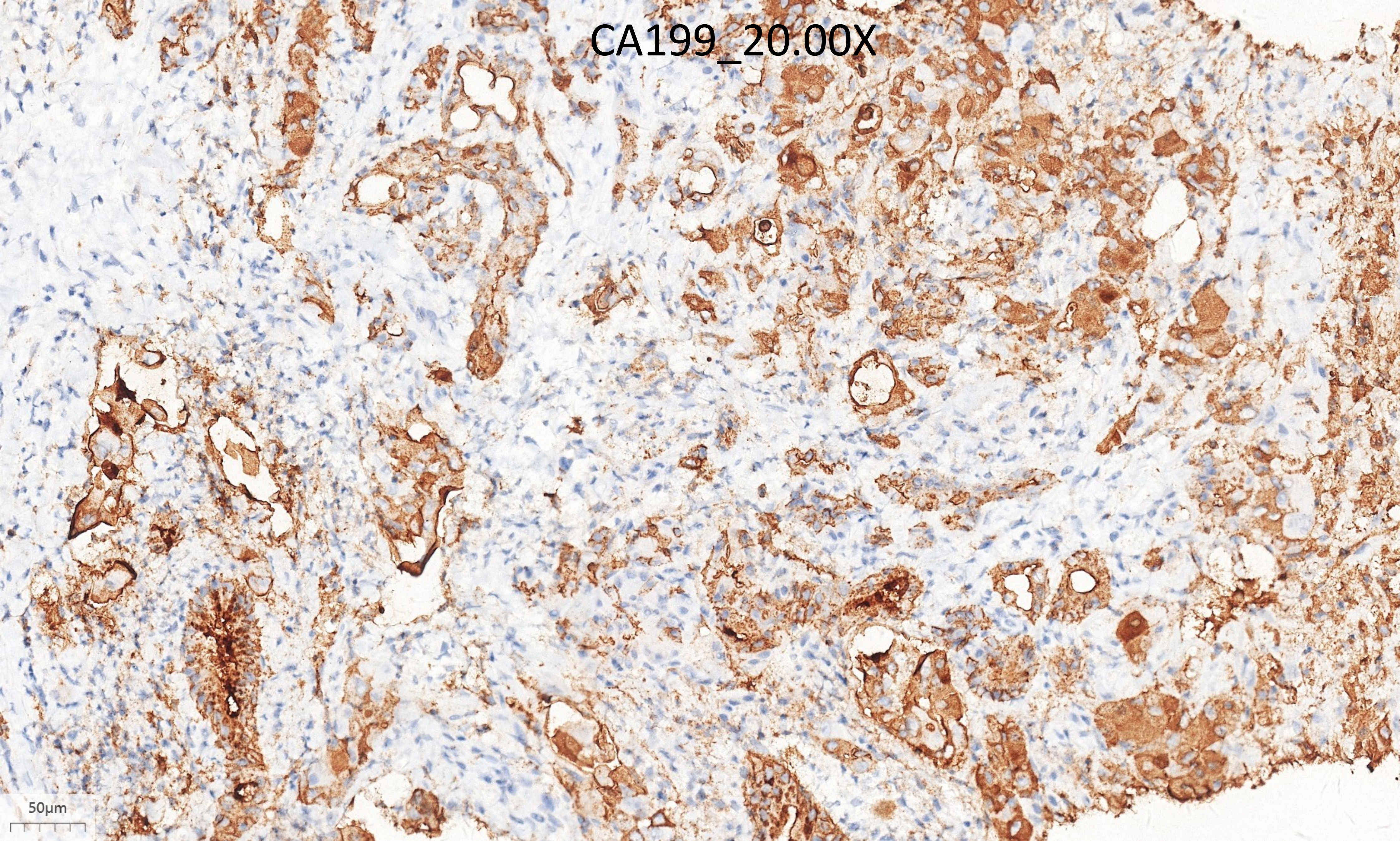
CA199_20.00X
50μm

CD38_10.00X

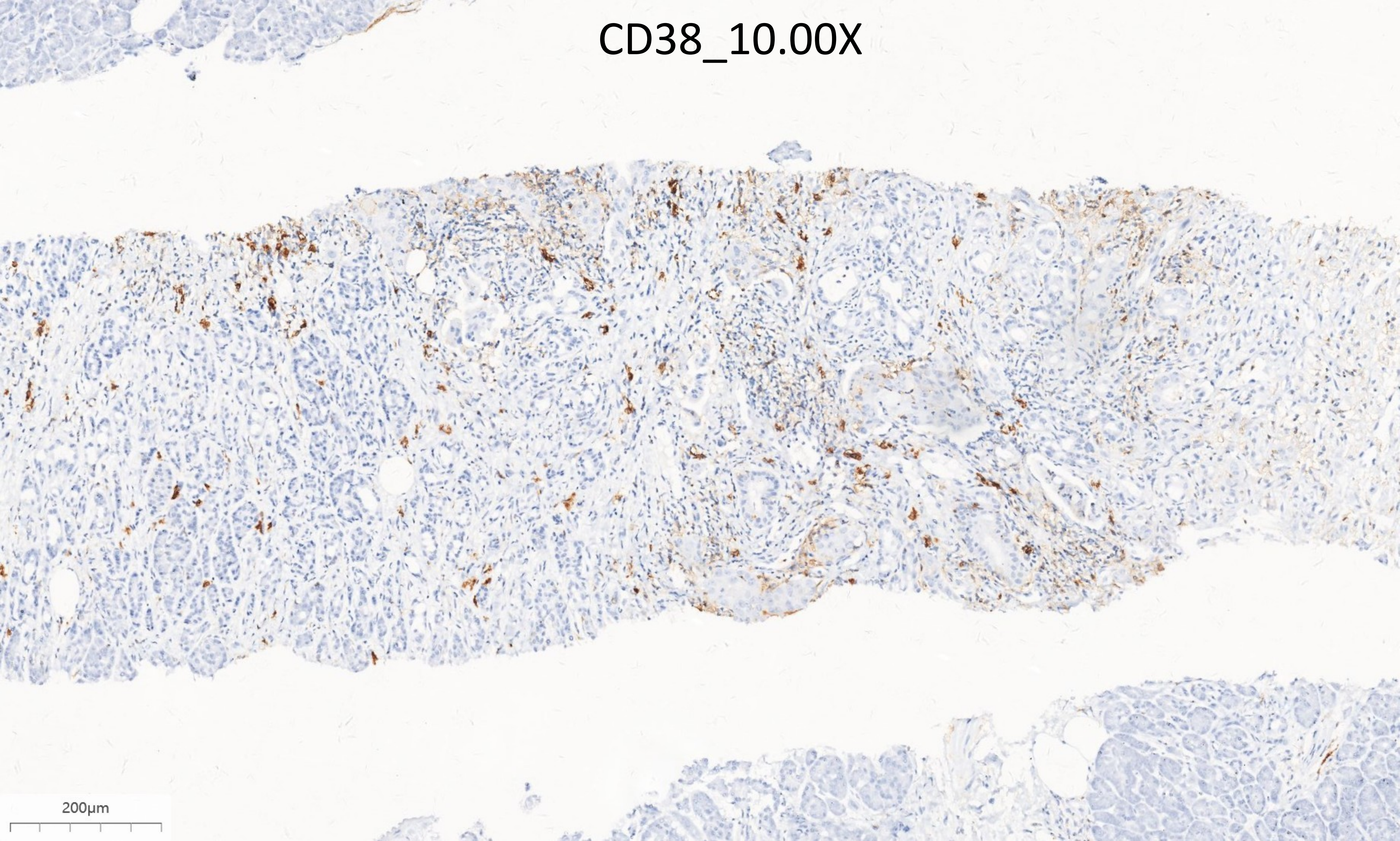

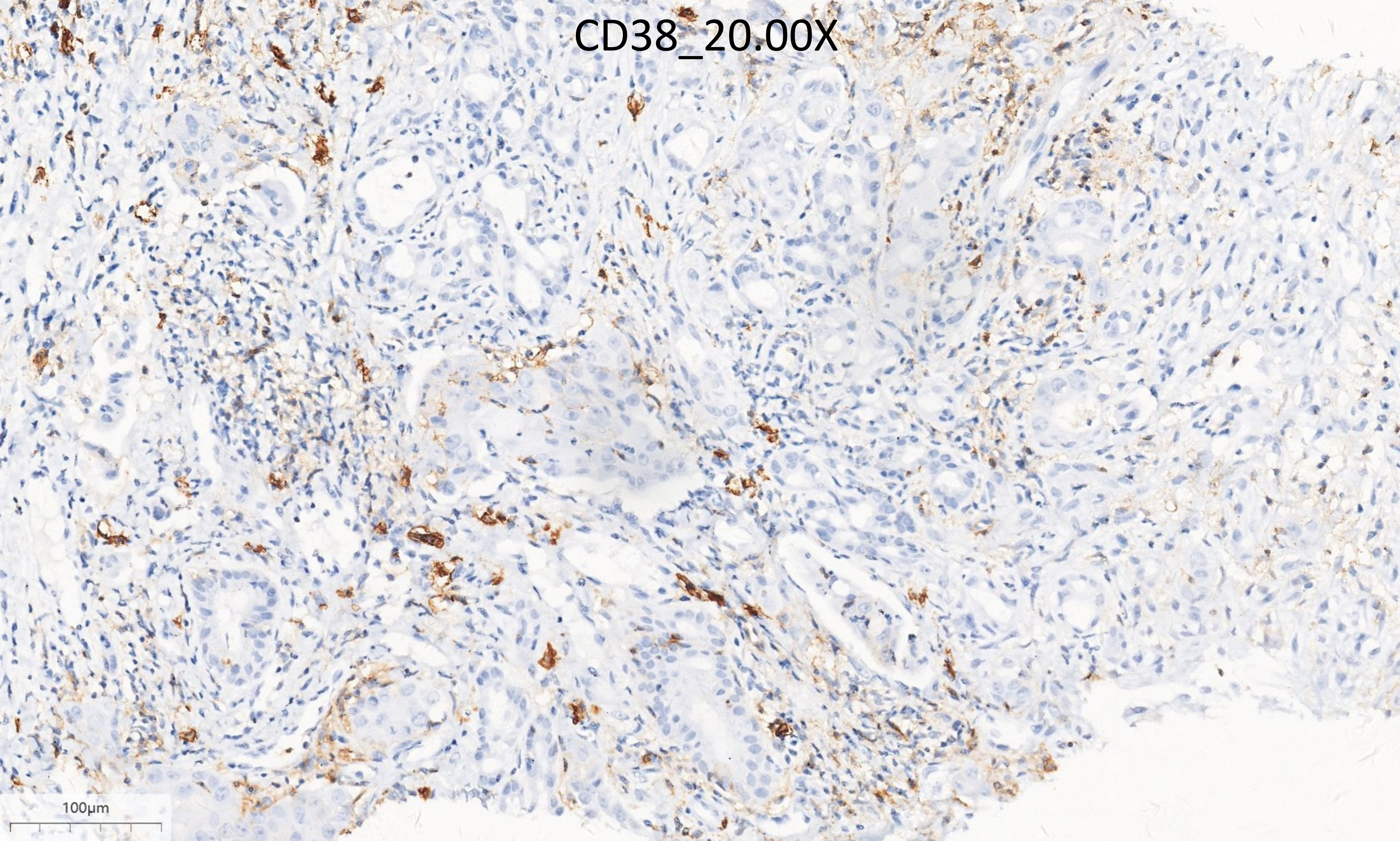
CD38_20.00X
100μm

CEA_10.00X

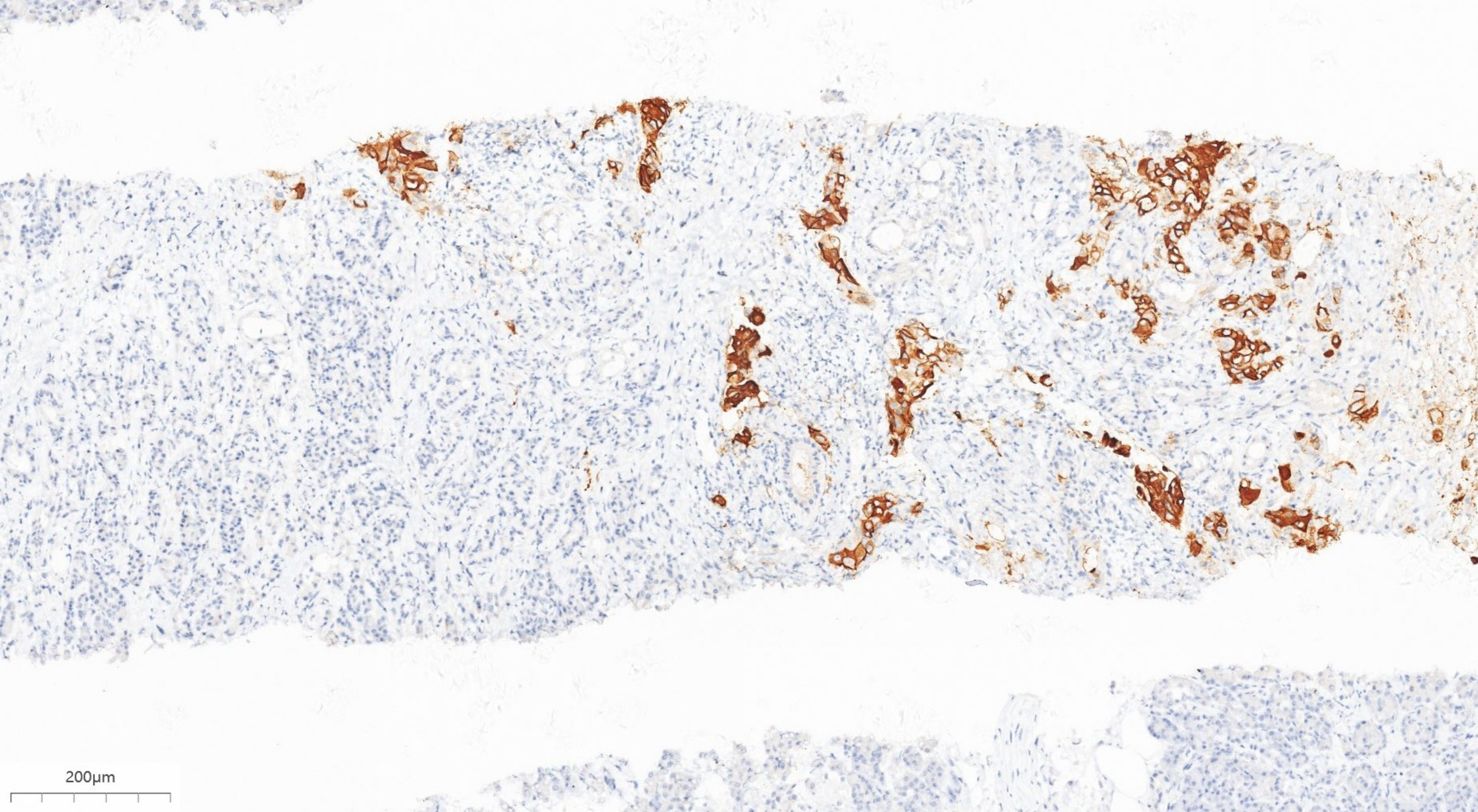

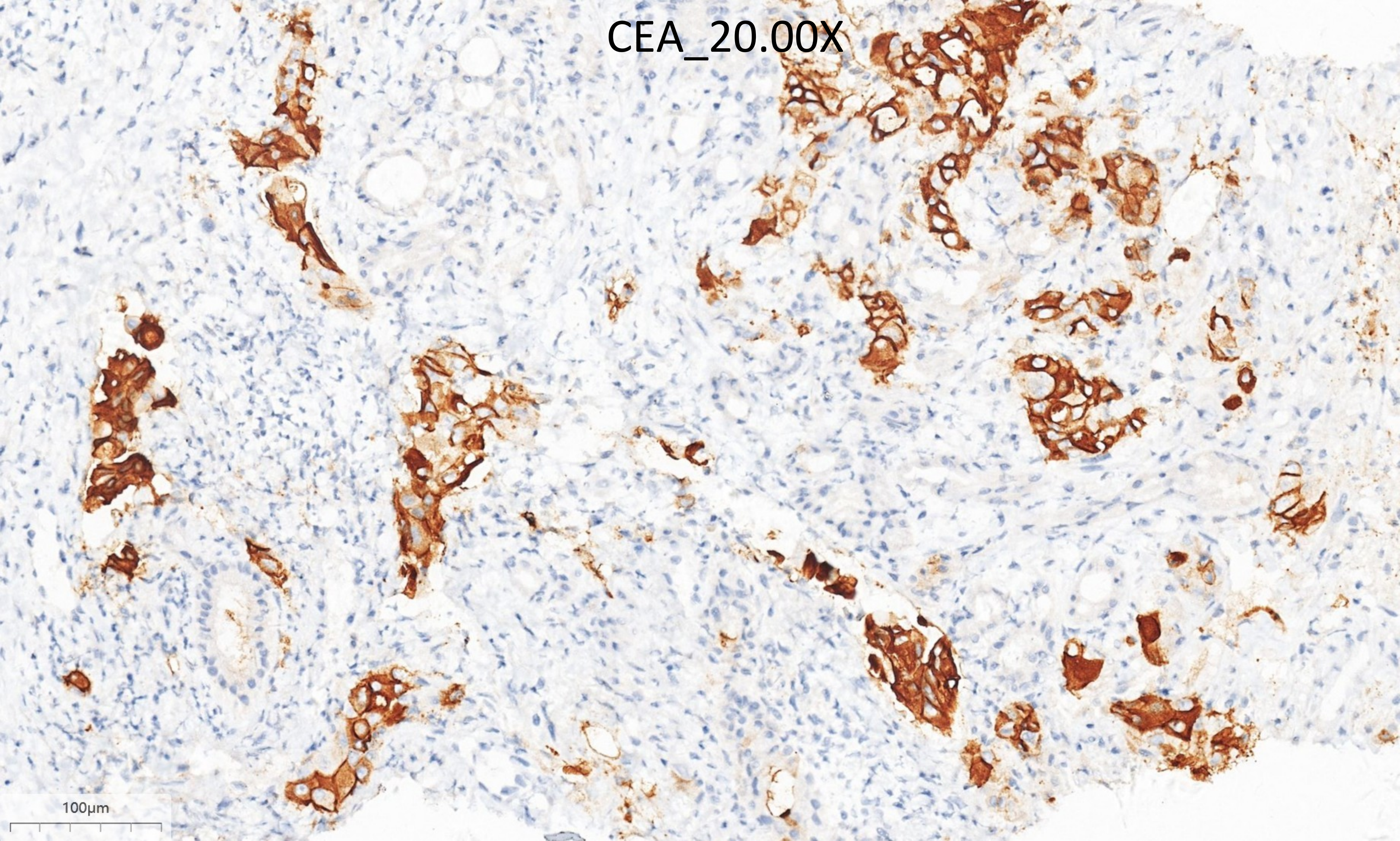
CEA_20.00X
100µm

CerbB-2_10.00X

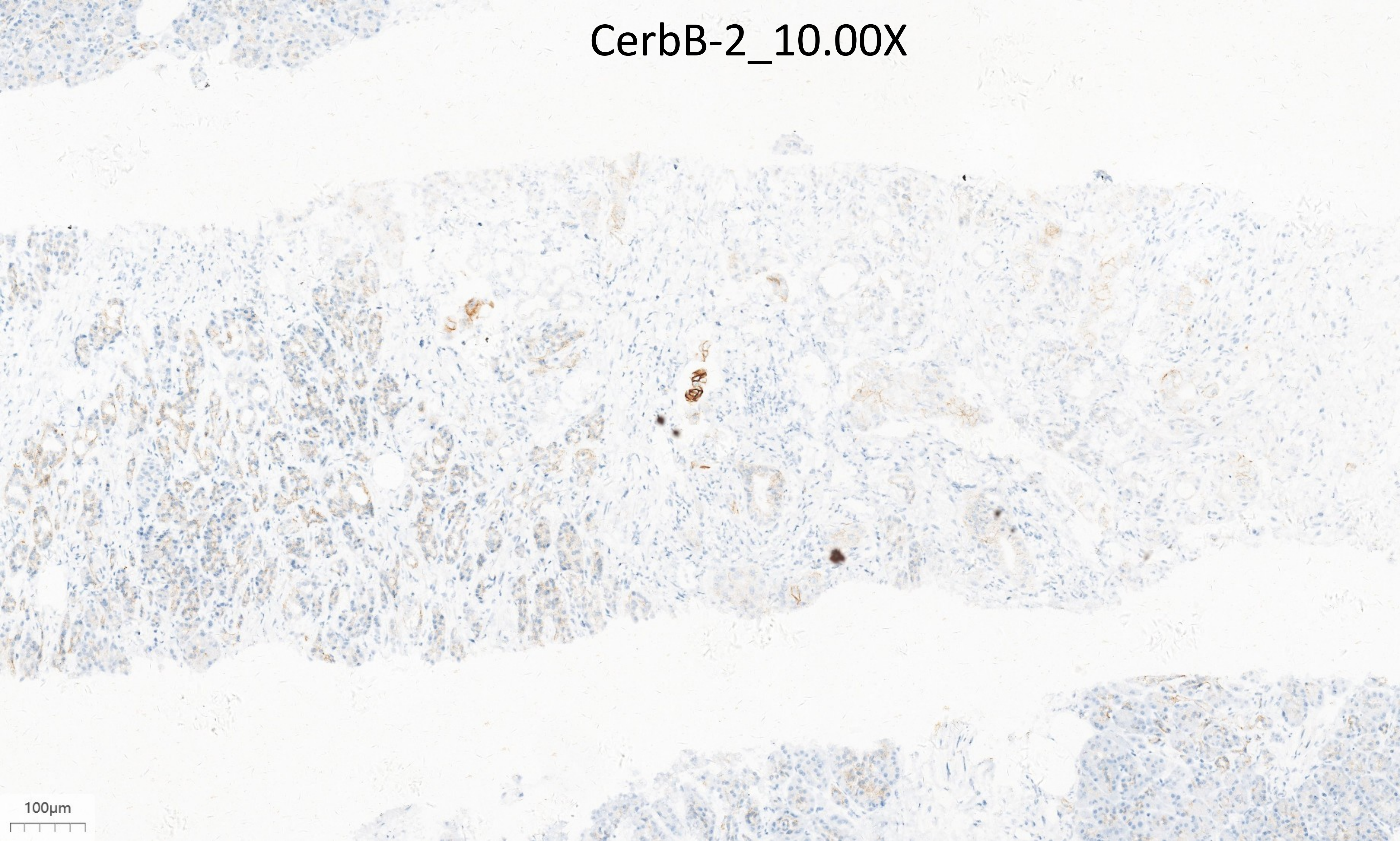

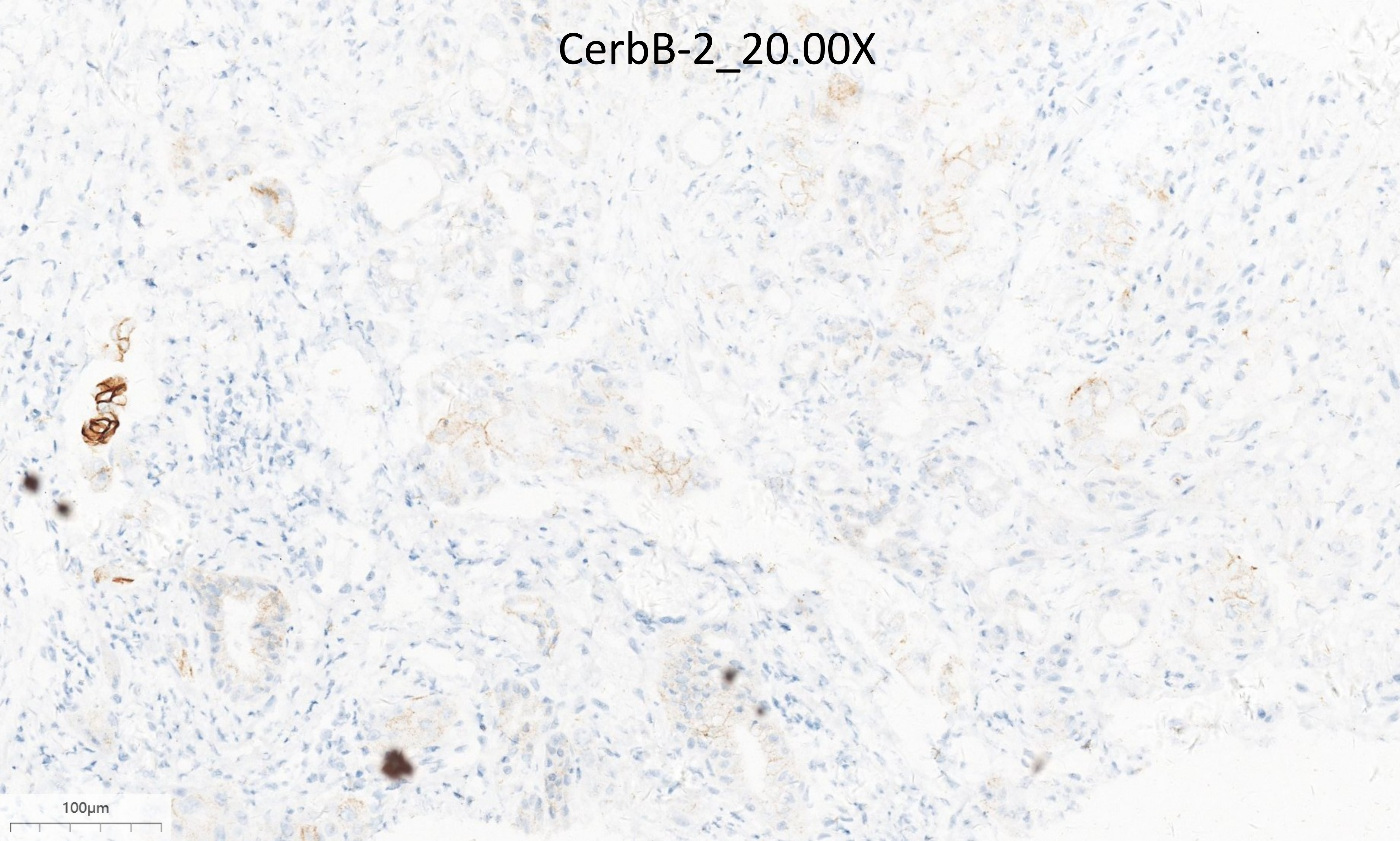
CerbB-2_20.00X
100µm

CK7_10.00X

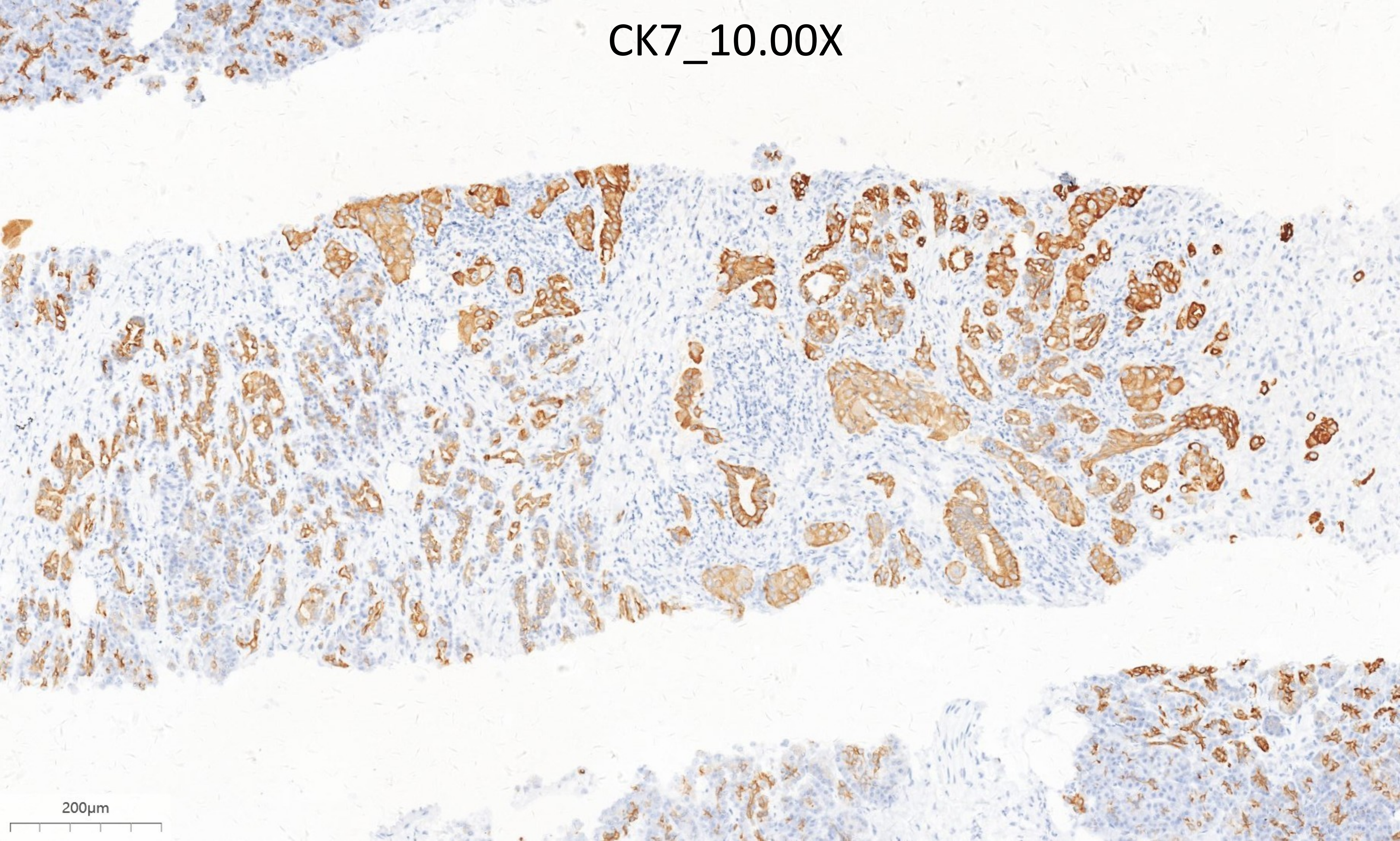

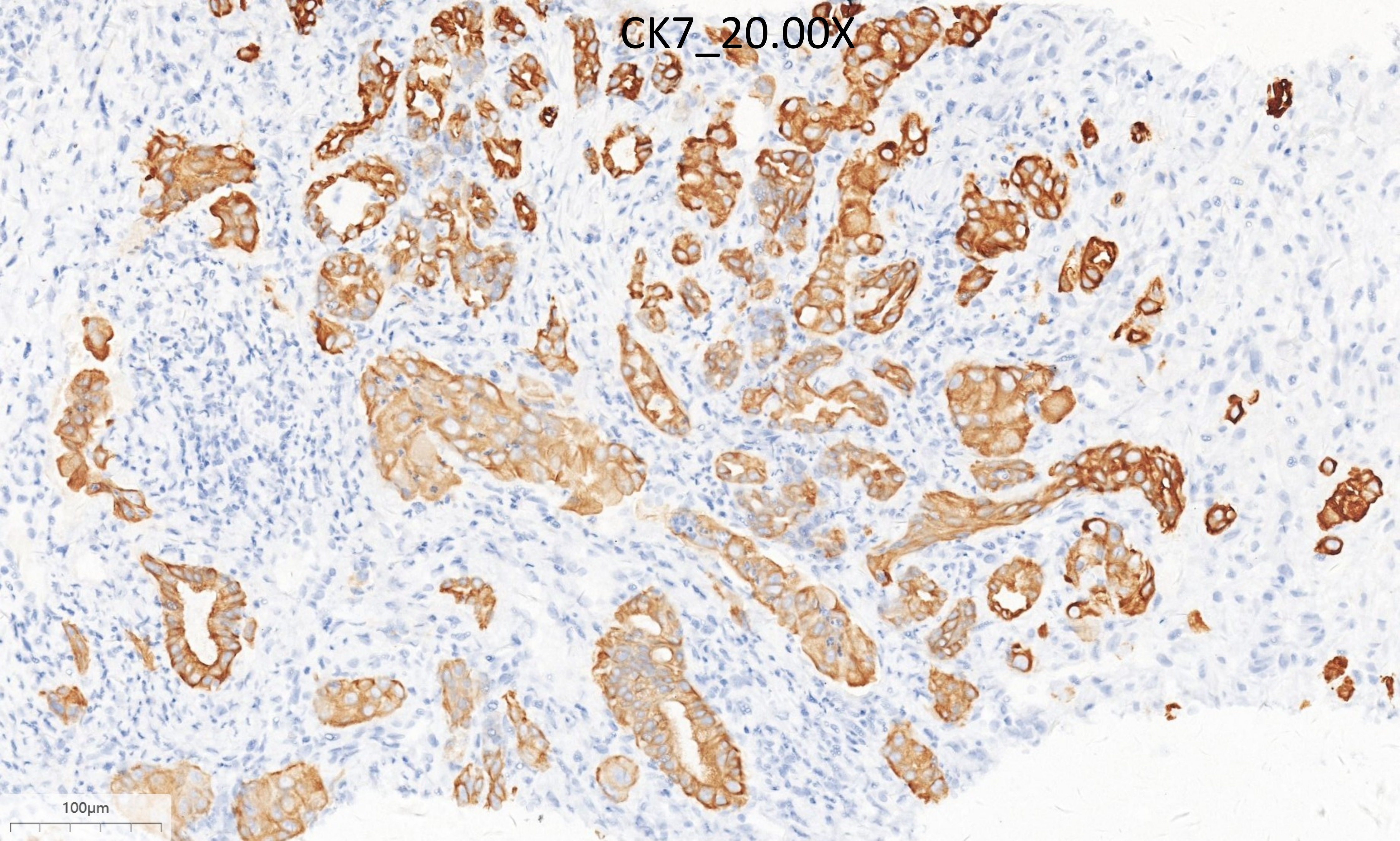
CK7_20.00X
100μm

CK19_10.00X

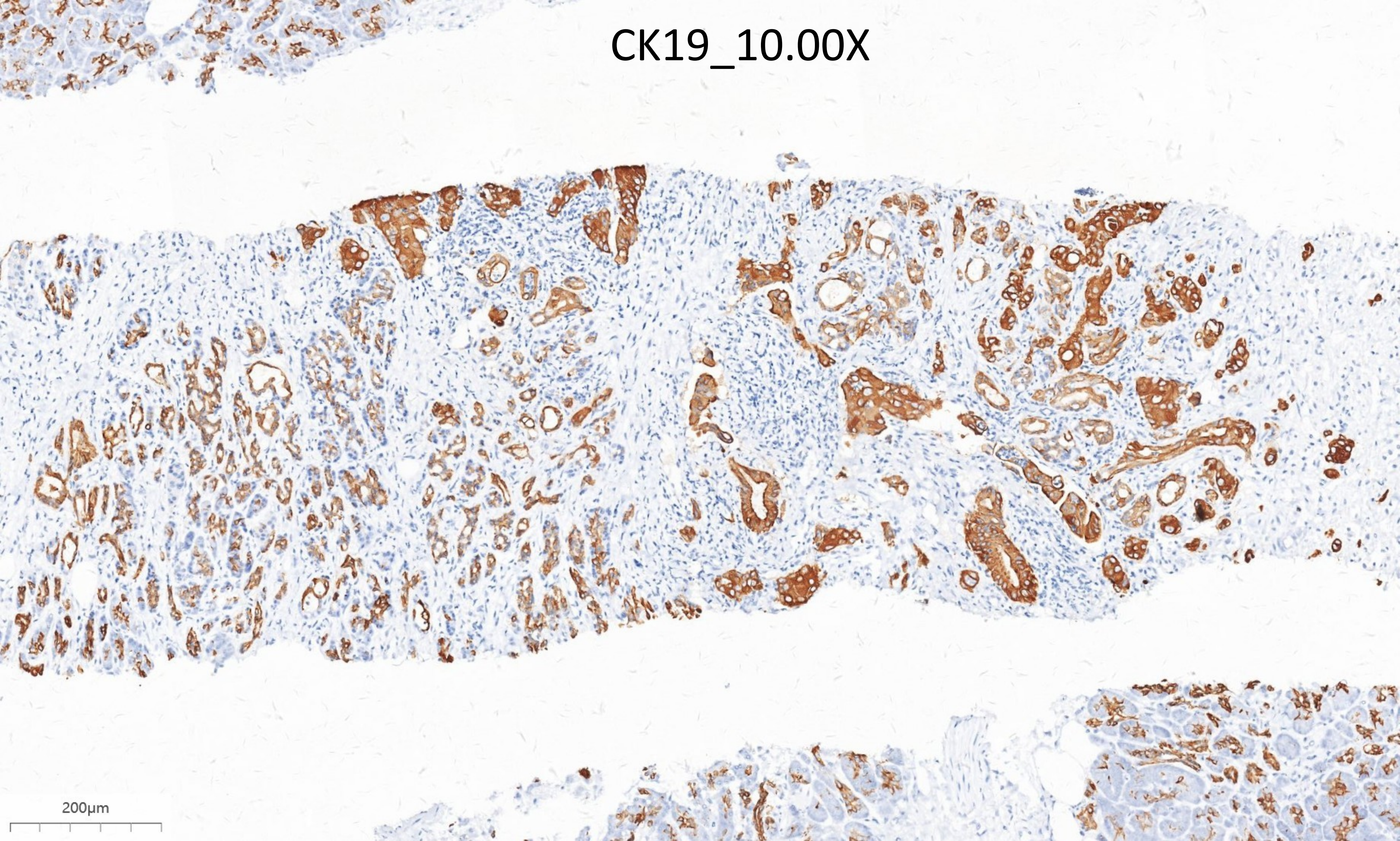

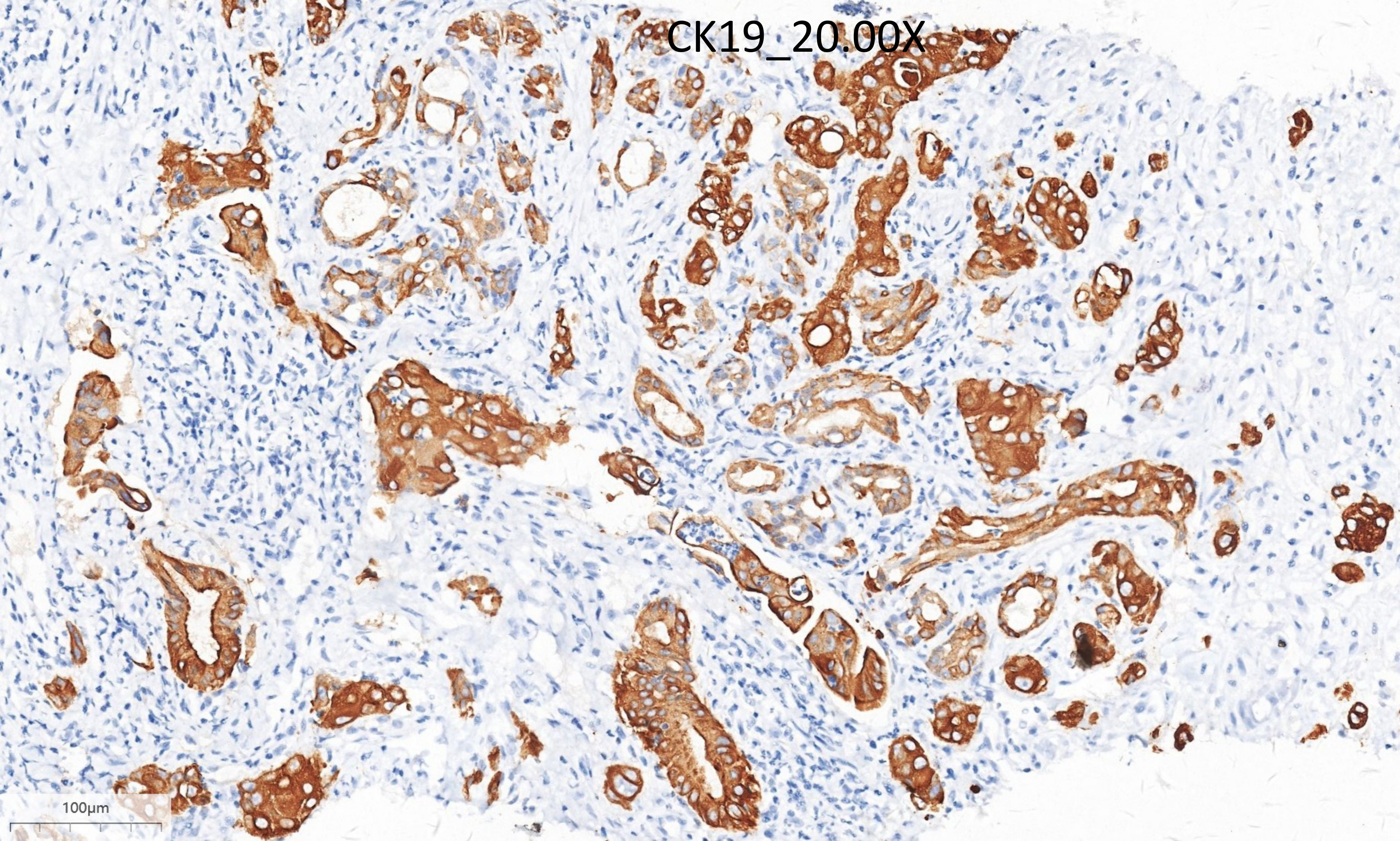
CK19_20.00X
100µm

CK20_10.00X

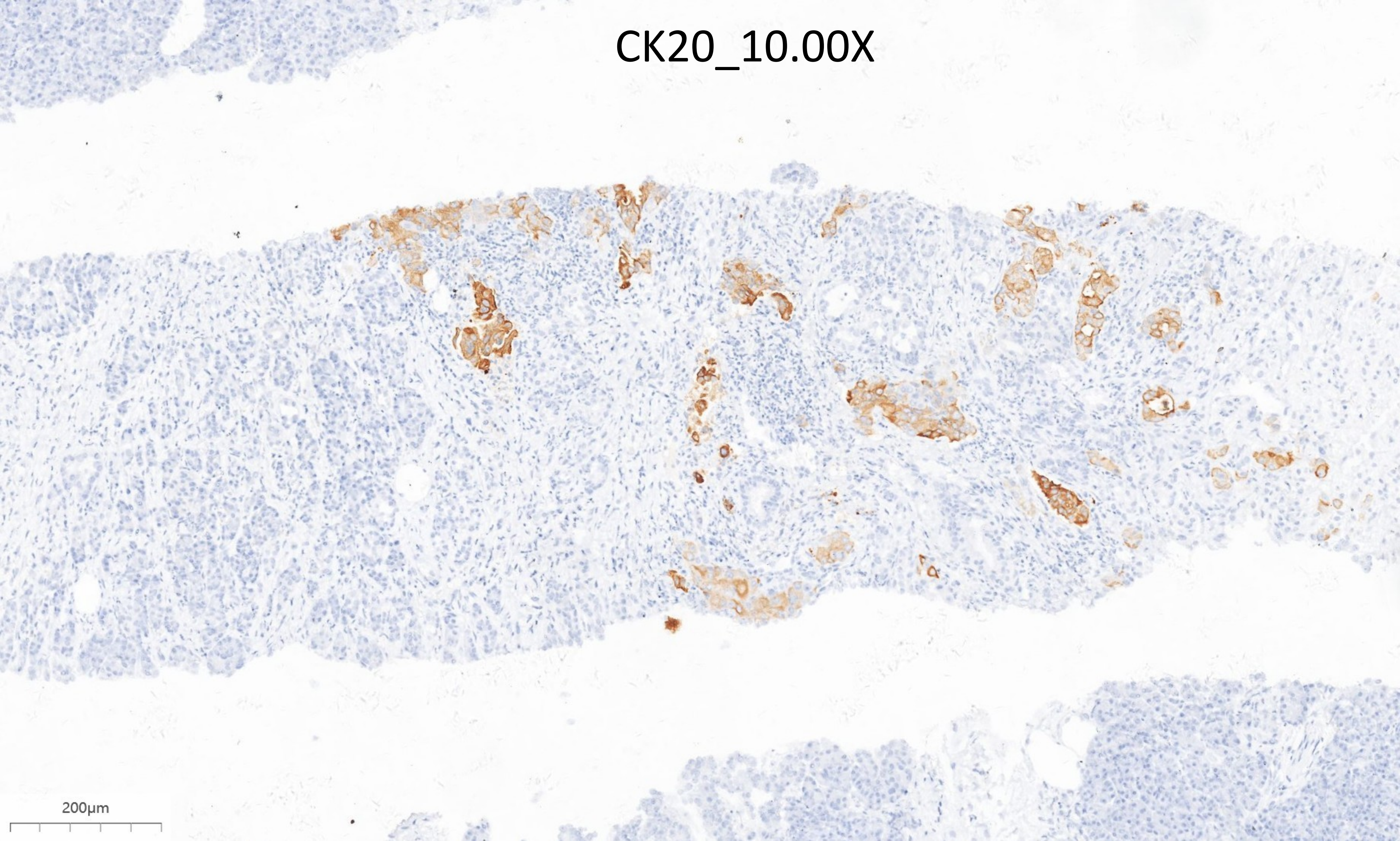

CK20_20.00X

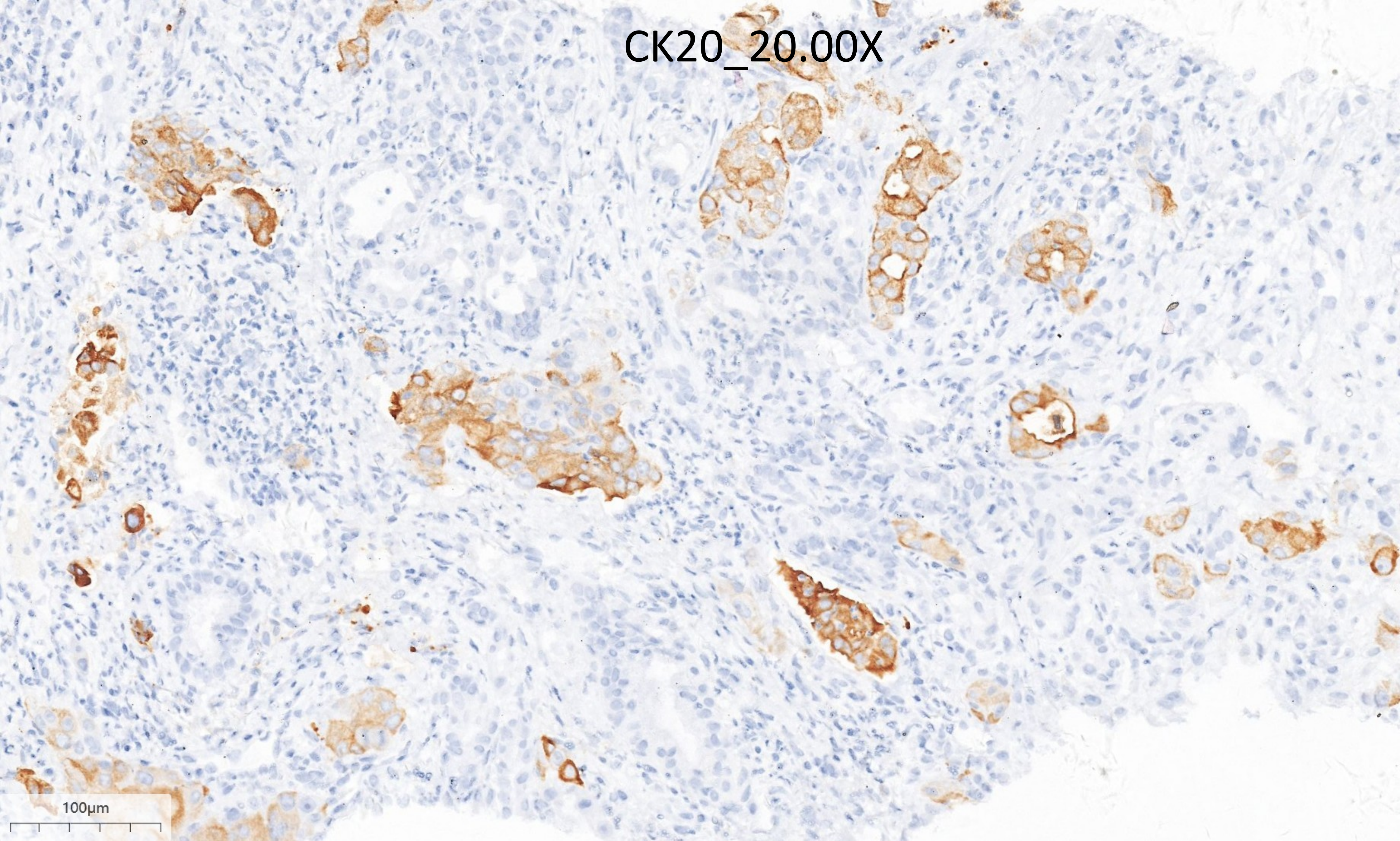

DPC-4_10.00X

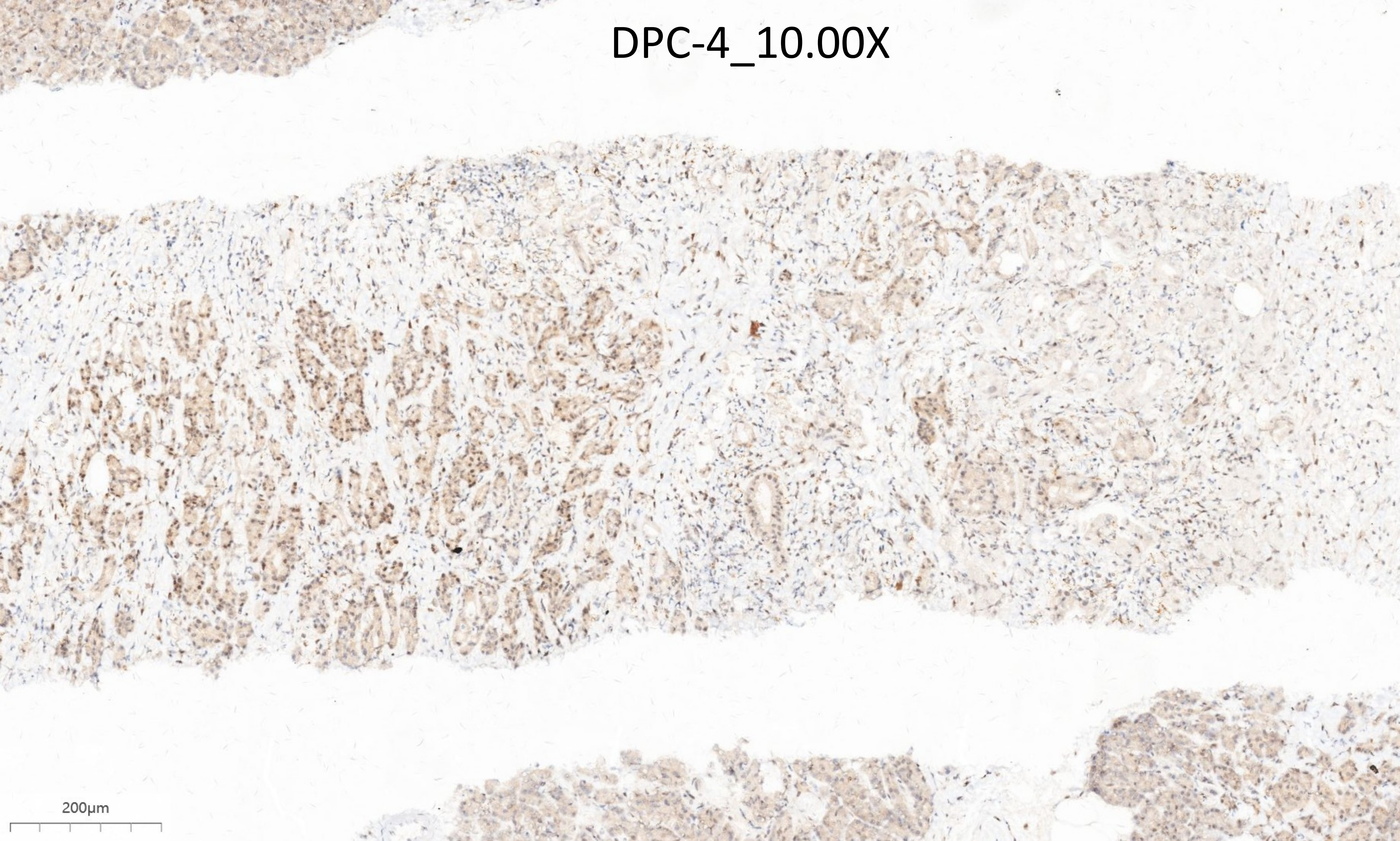

DPC-4_20.00X

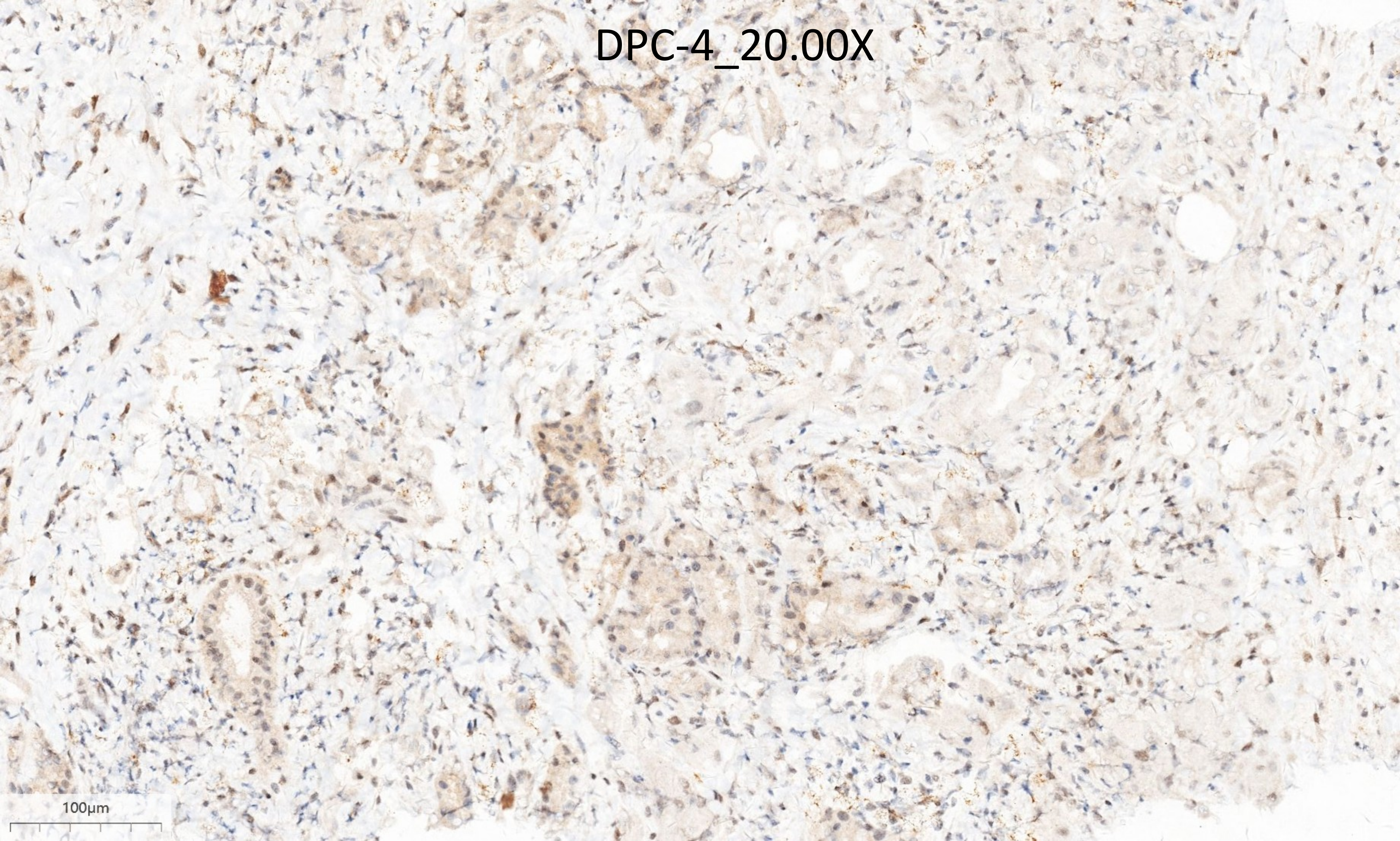

HE1_10.00X

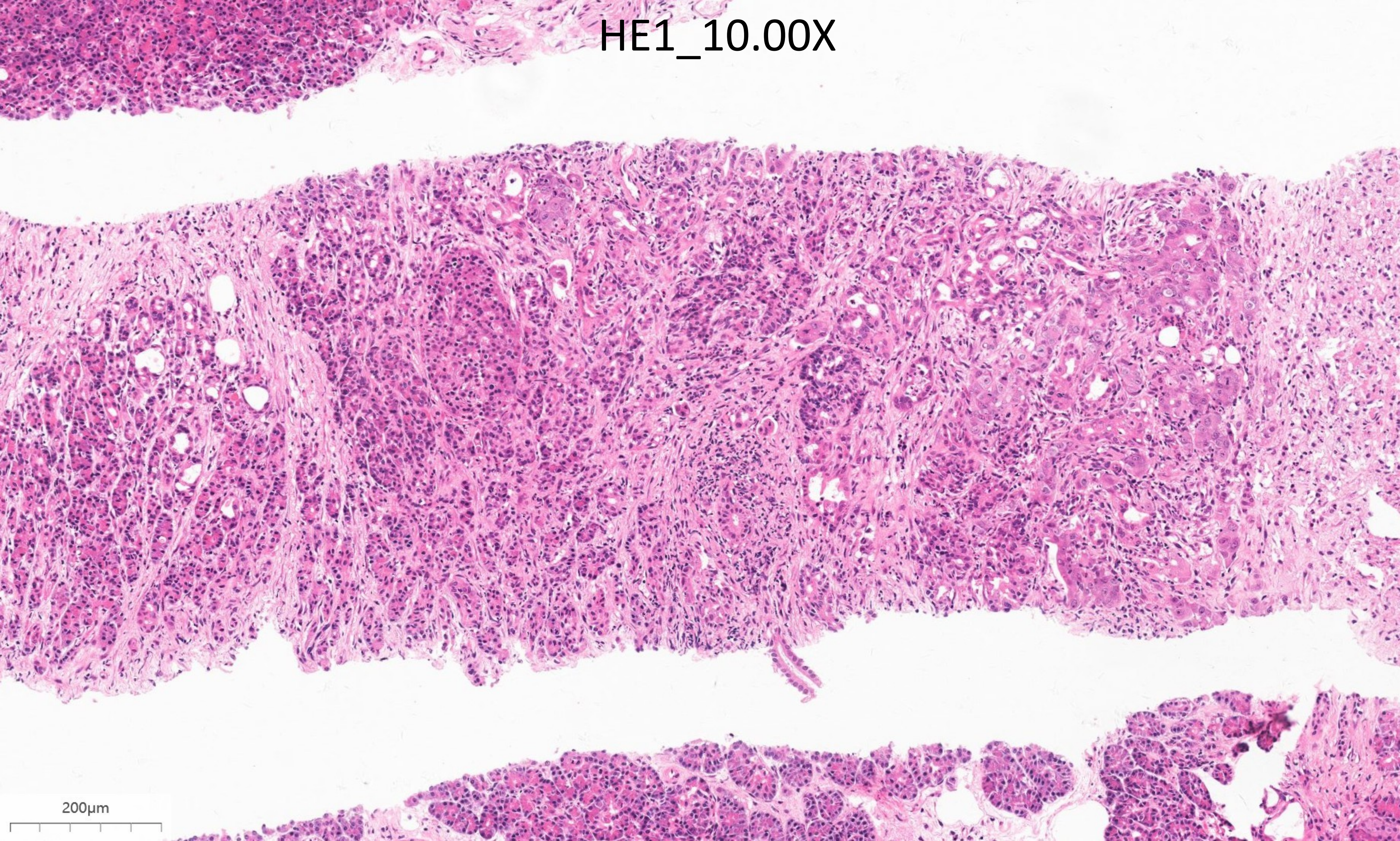

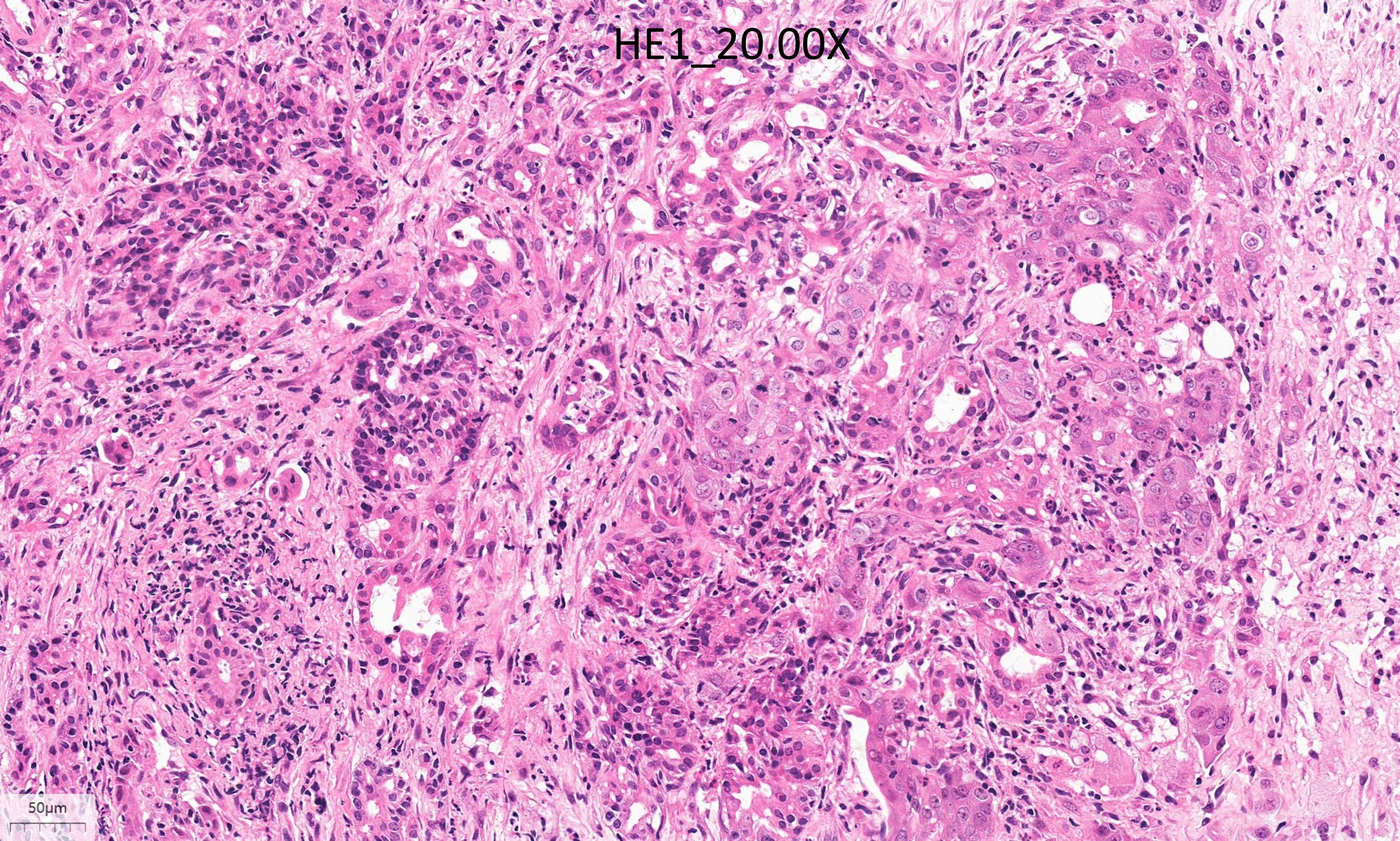
HE1_20.00X
50µm

HNF-1β_10.00X

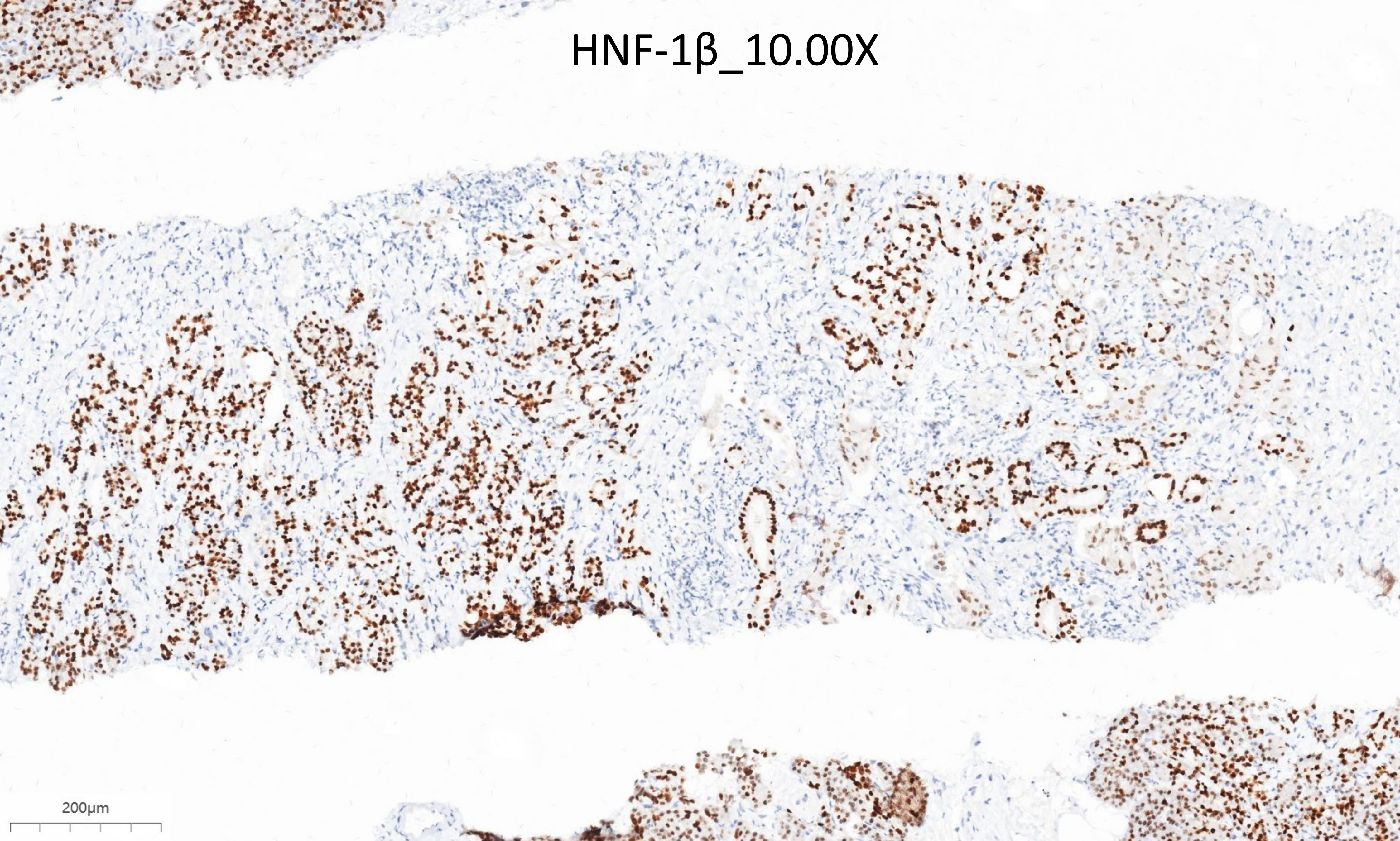

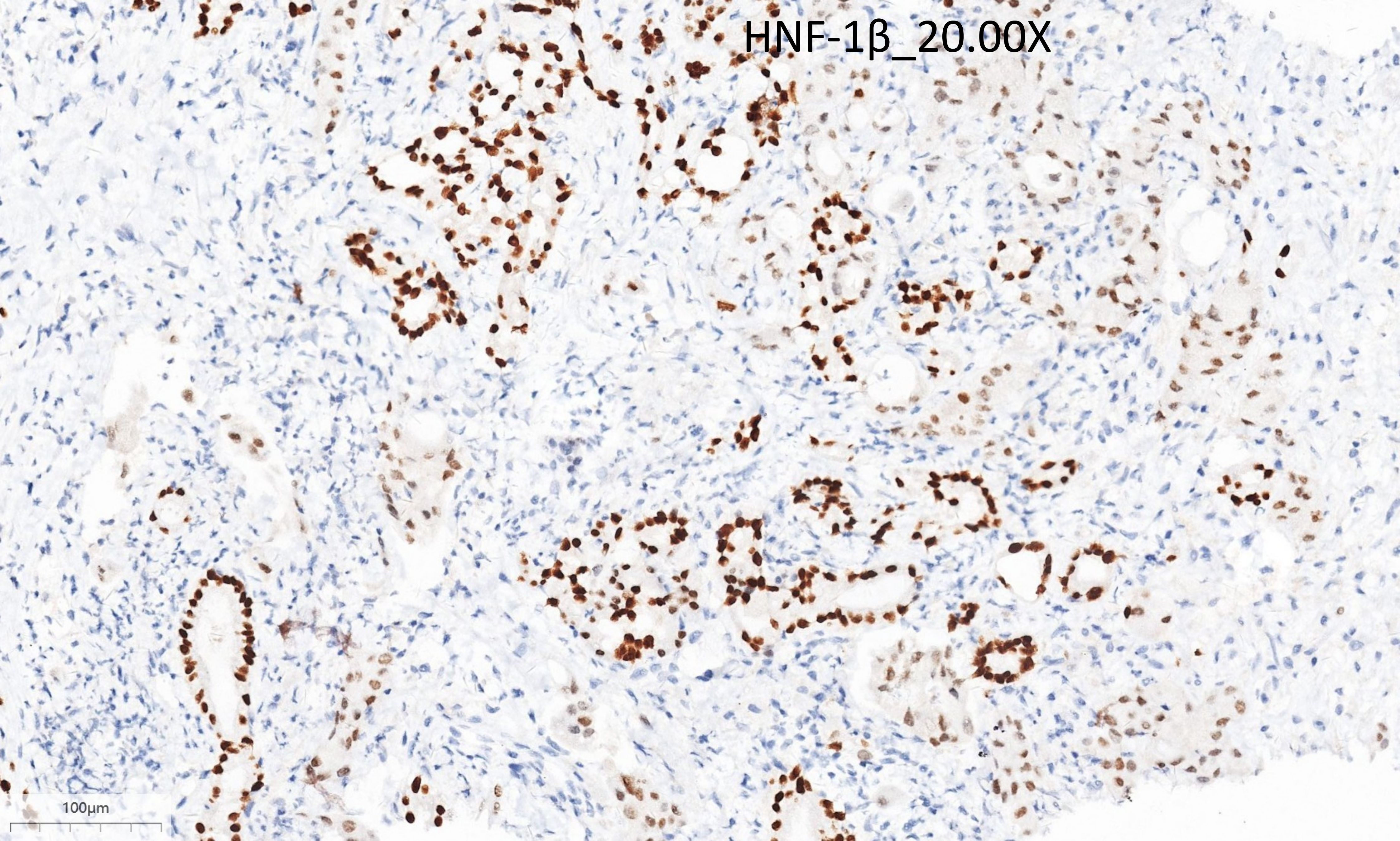
HNF-1β_20.00X
100μm

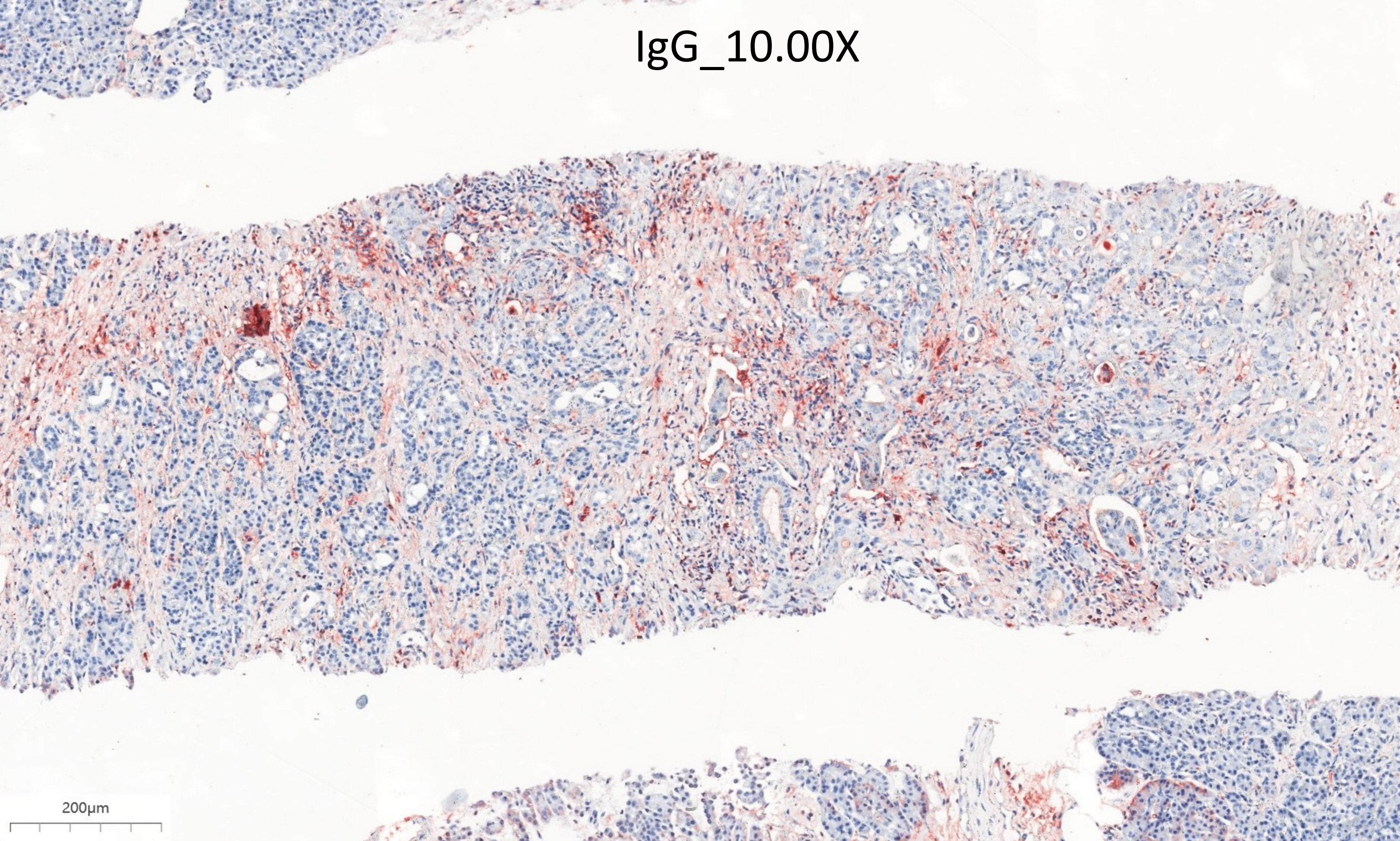
IgG_10.00X
200μm

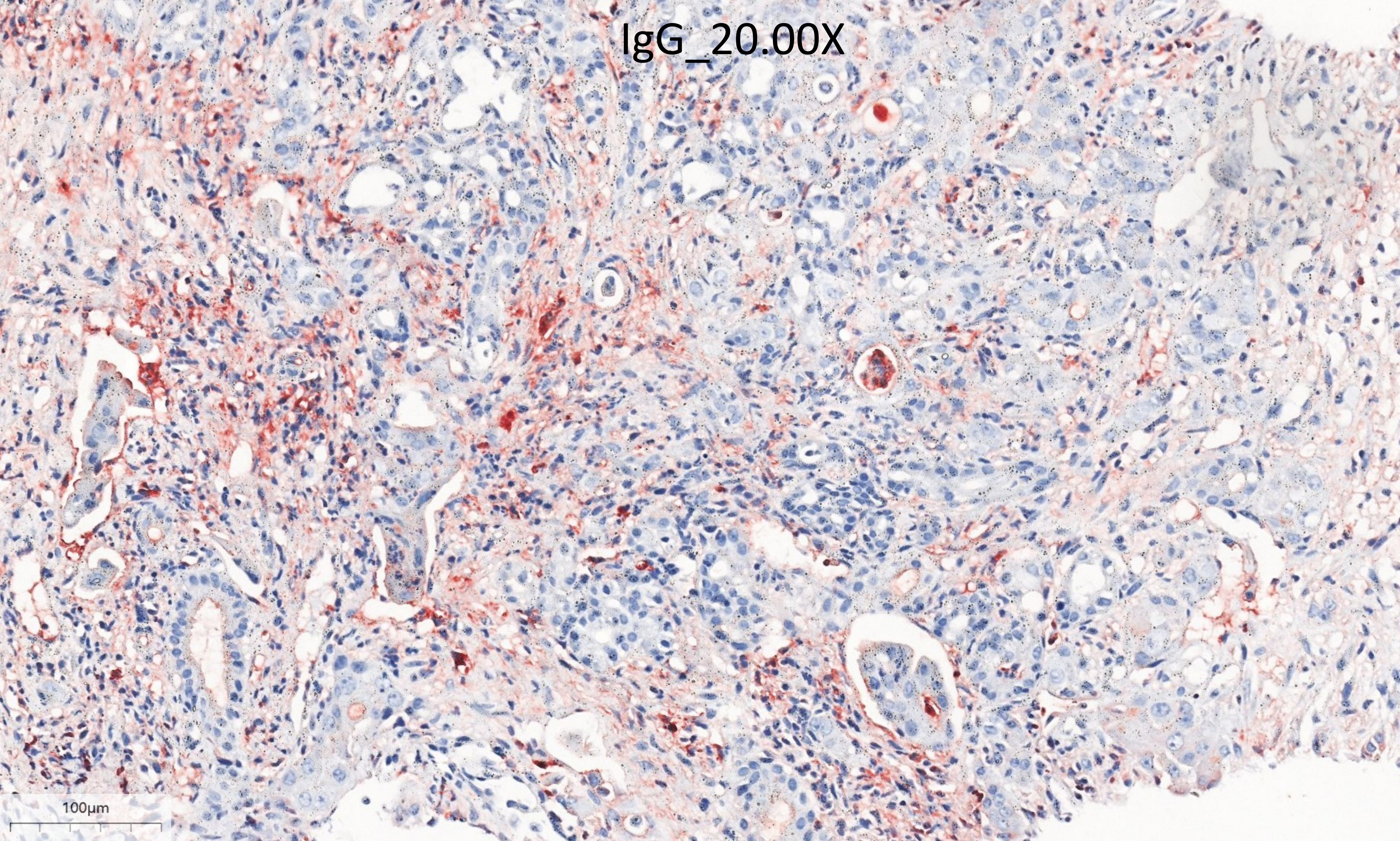
IgG_20.00X
100µm

IgG4_10.00X

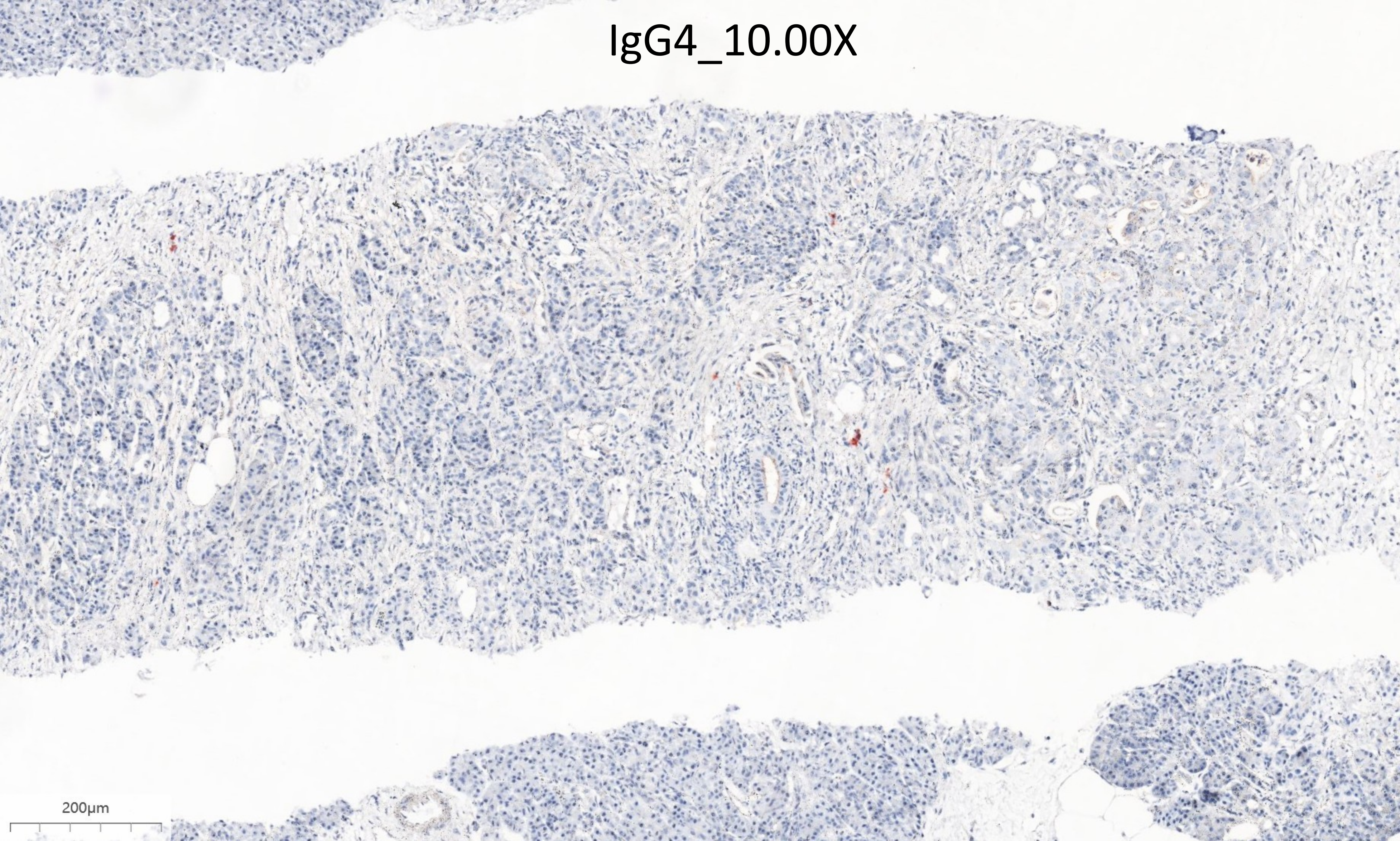

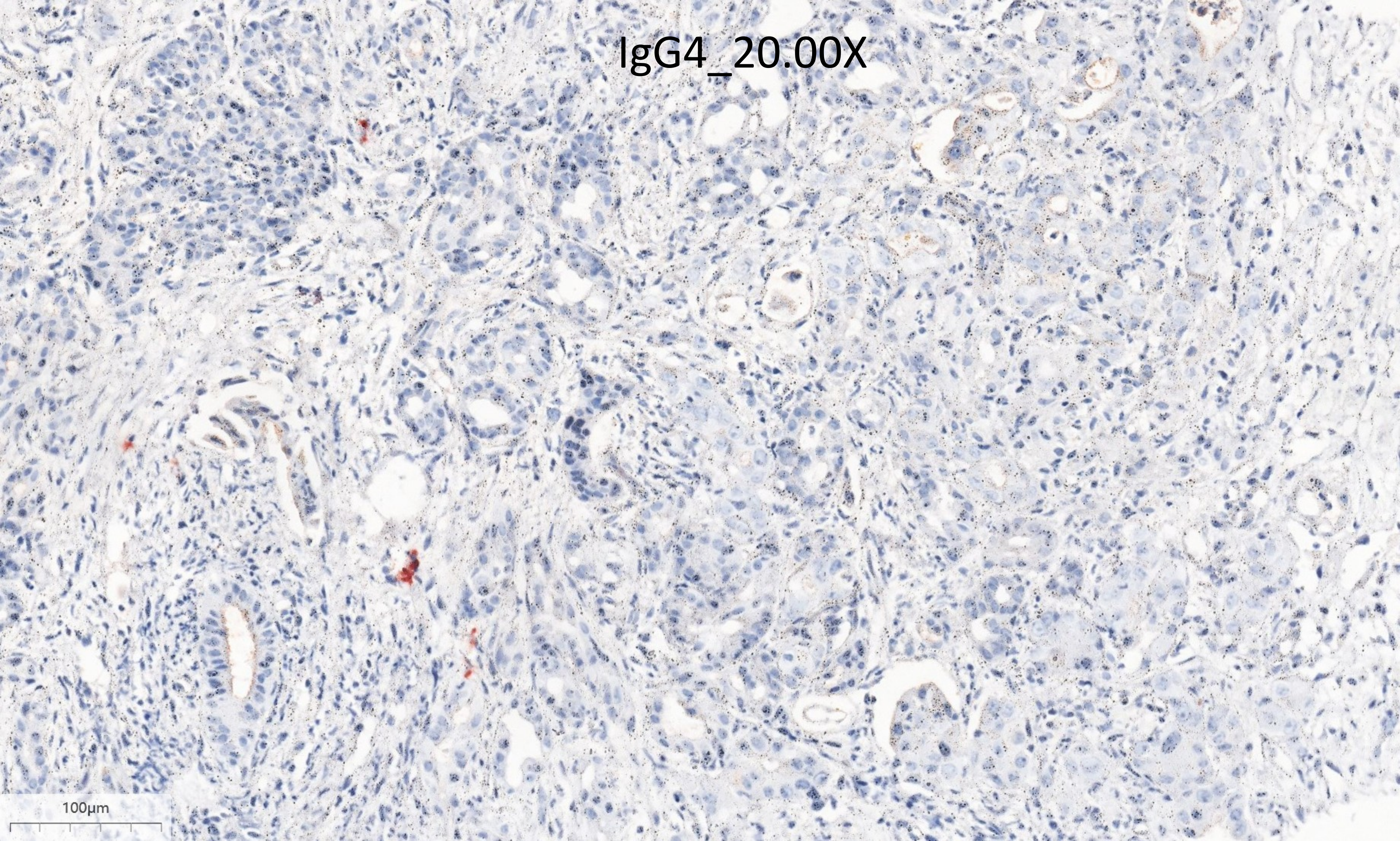
IgG4_20.00X
100µm

INSM1_10.00X

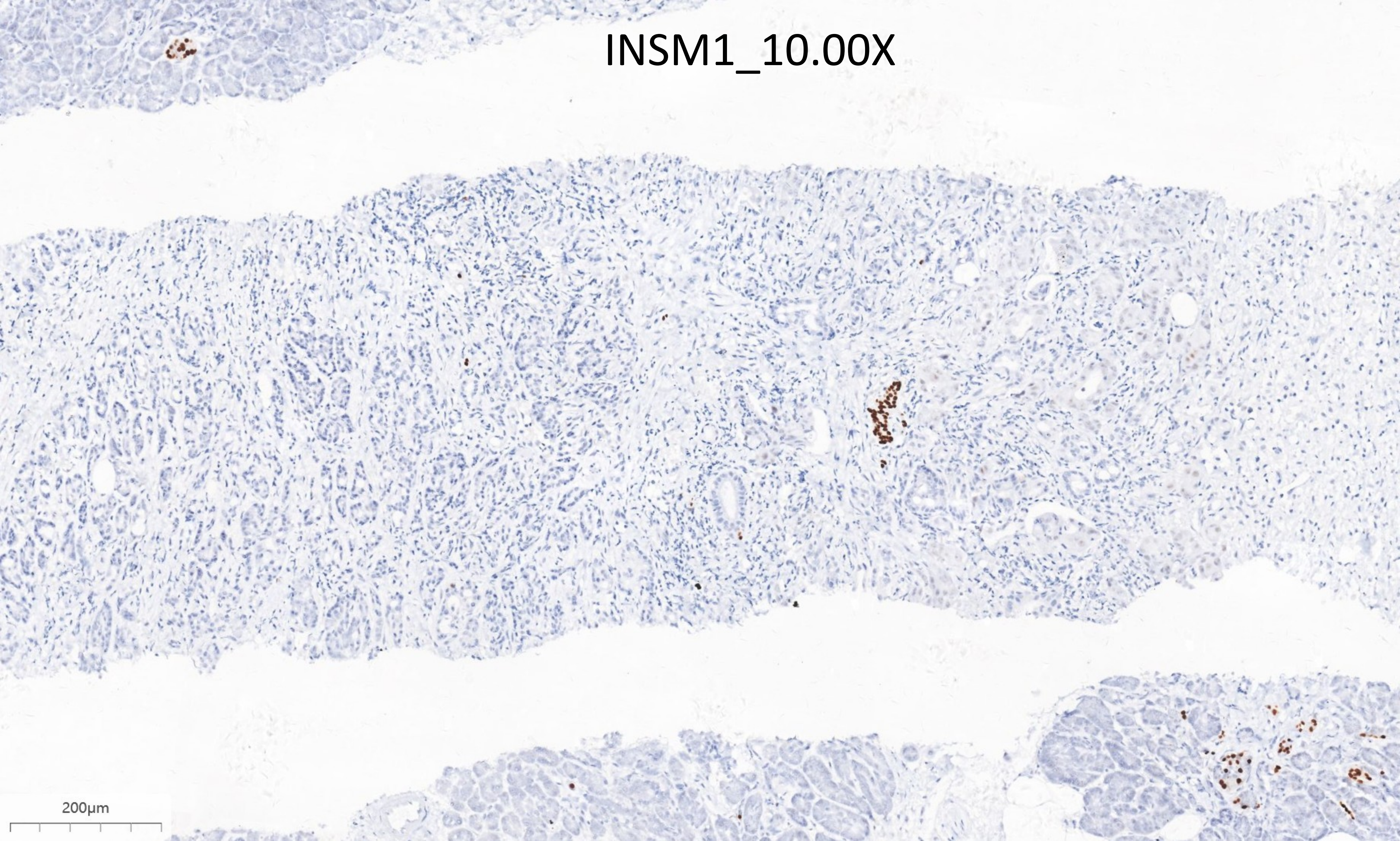

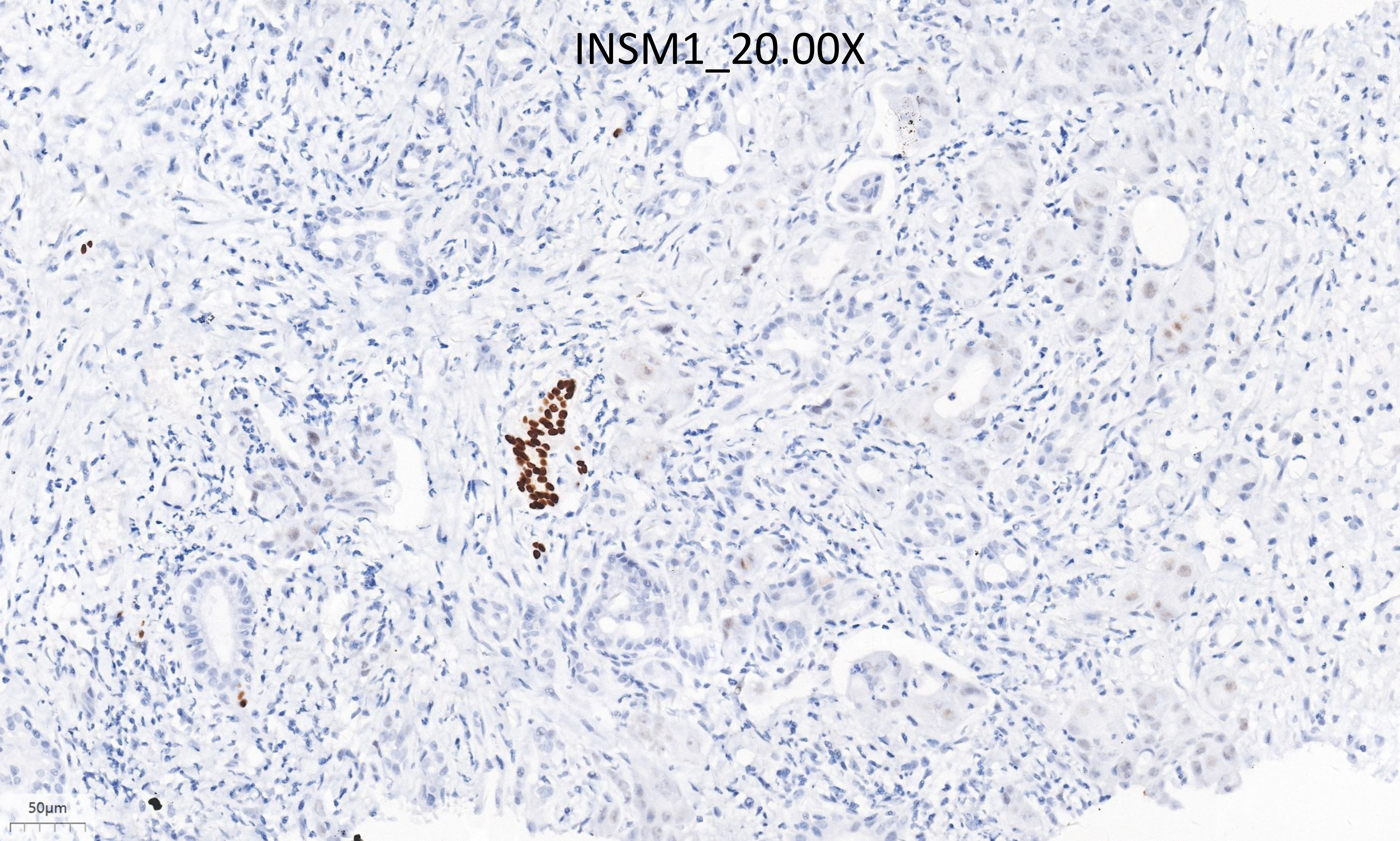
INSM1_20.00X
50μm

Ki67_10.00X

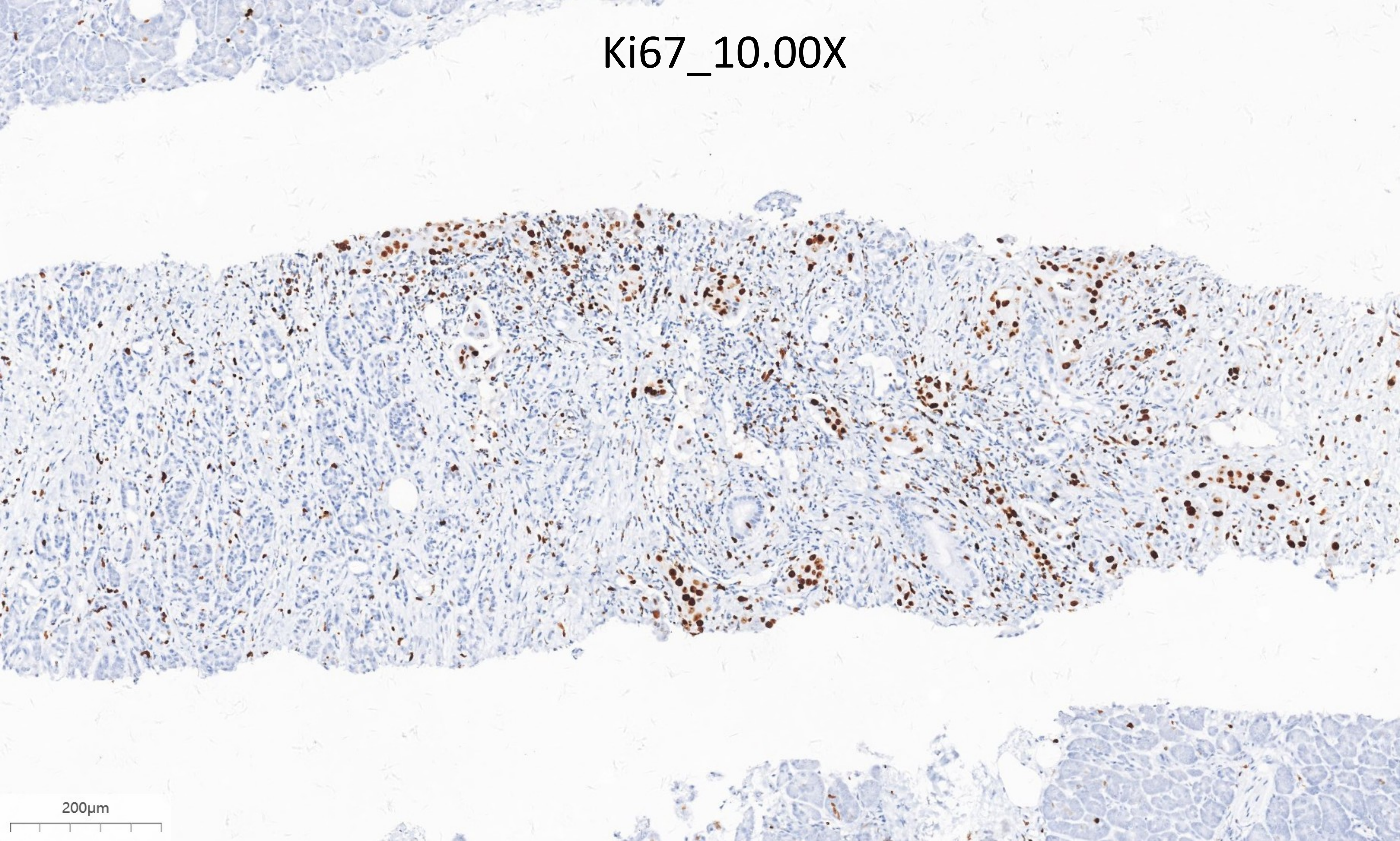

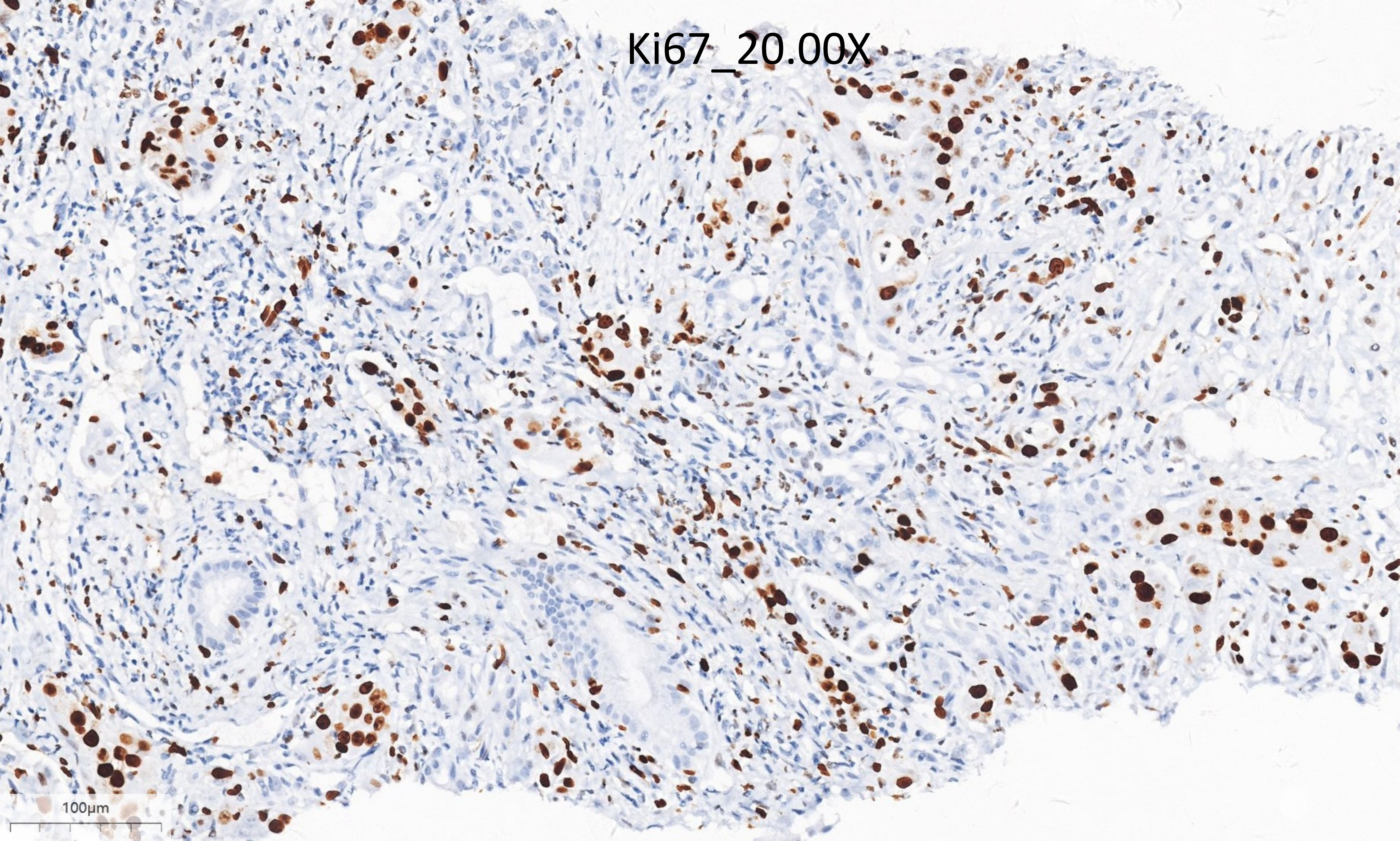
Ki67_20.00X
100μm

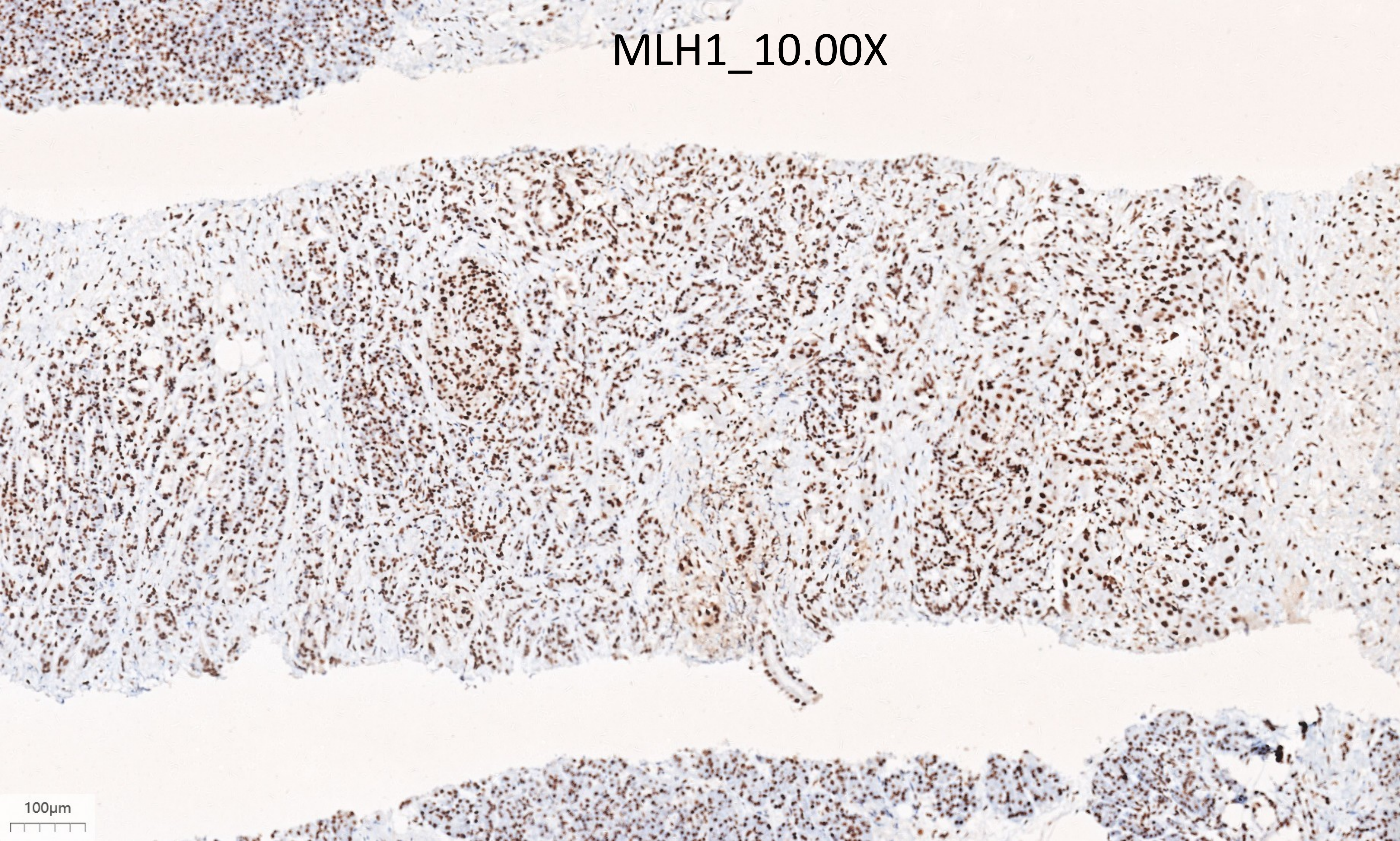
MLH1_10.00X
100µm

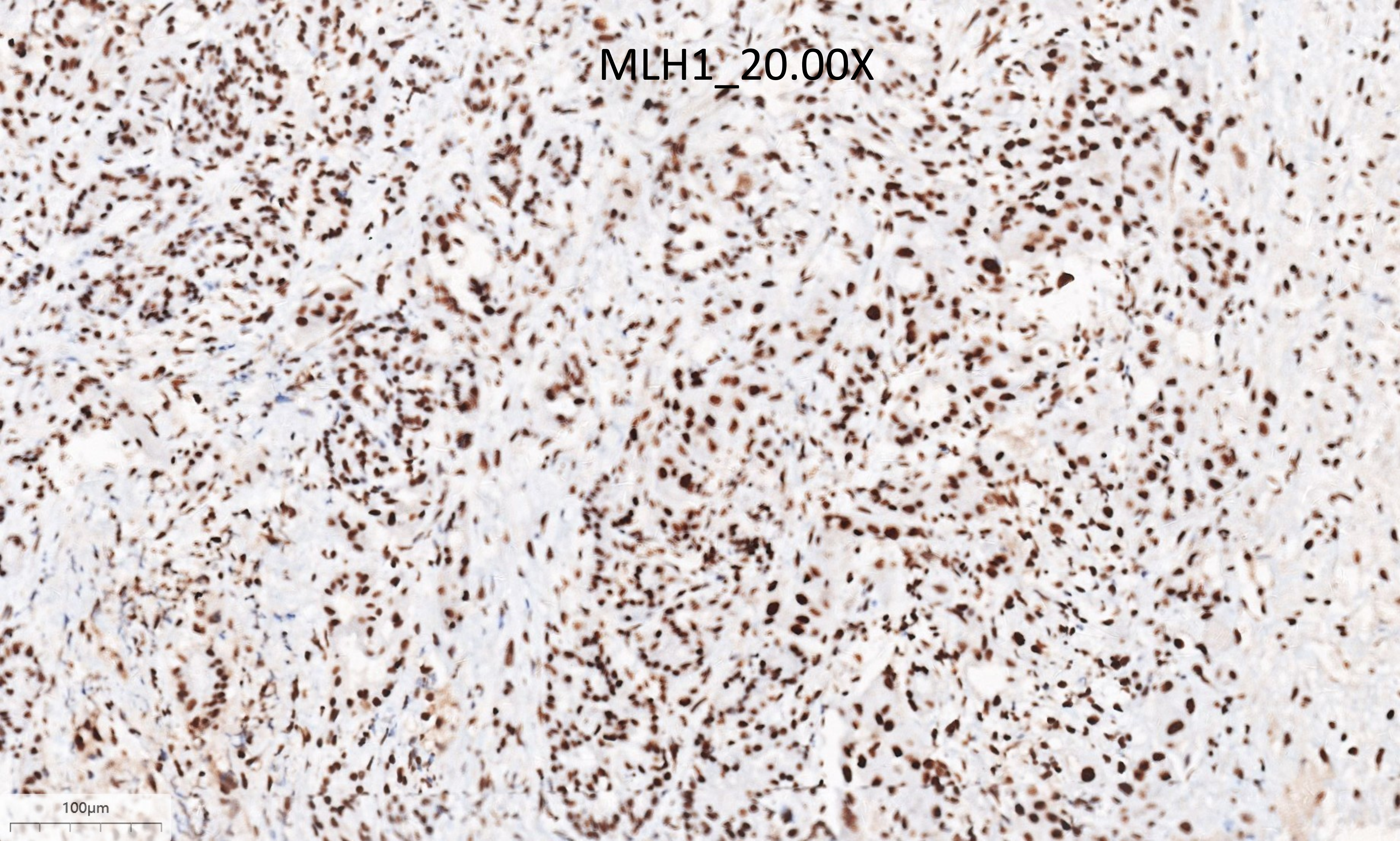
MLH1_20.00X
100μm

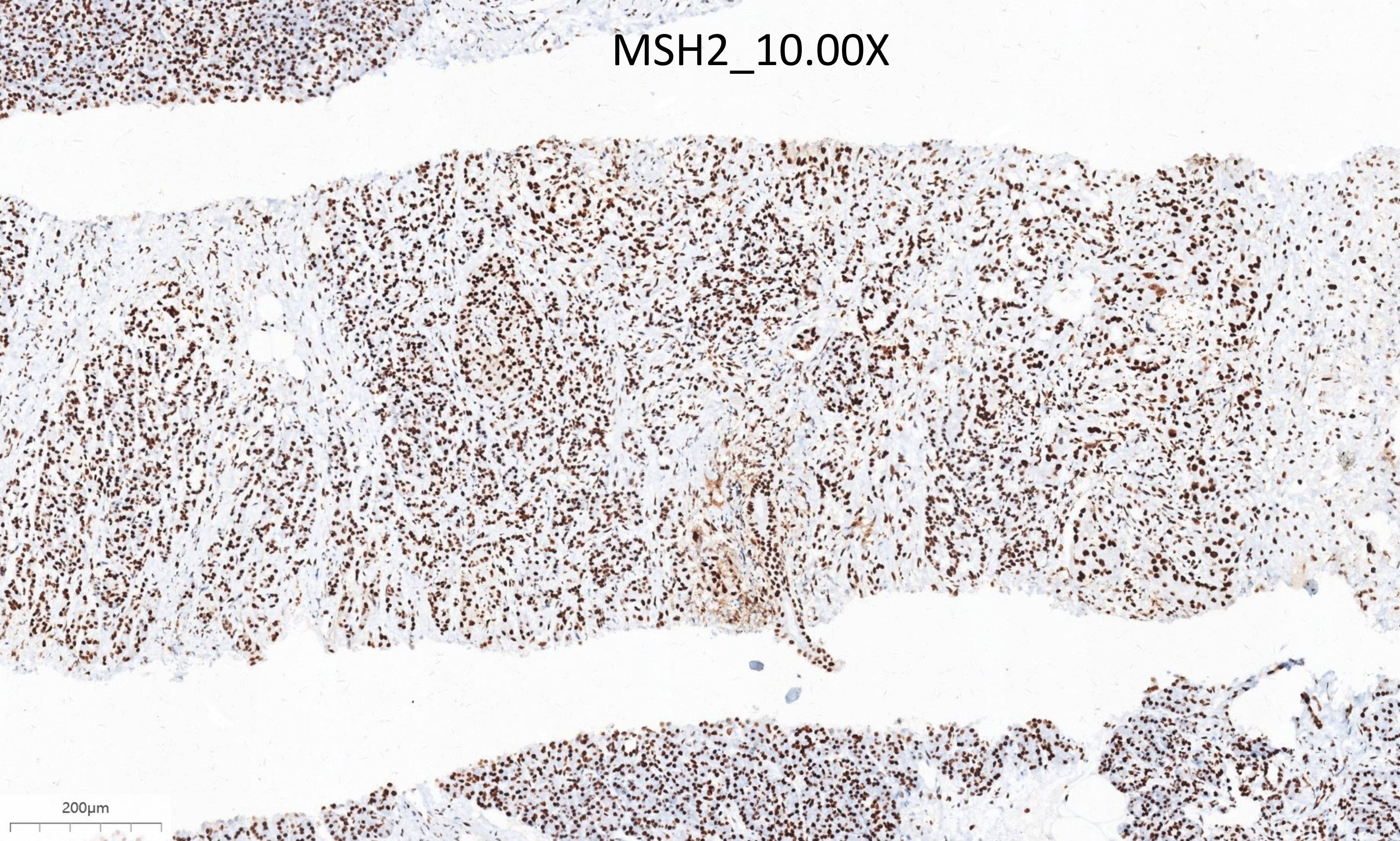
MSH2_10.00X
200µm

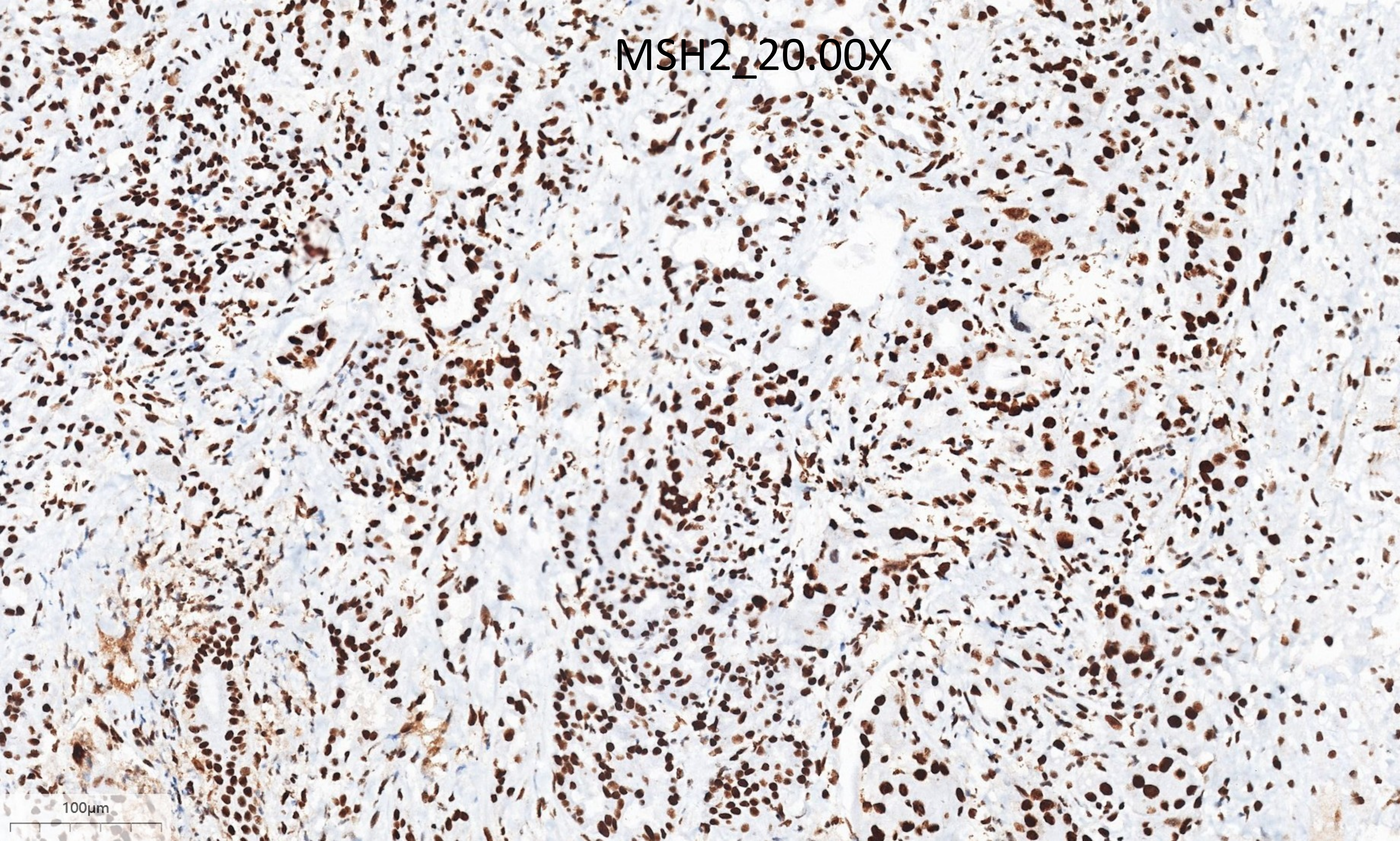
MSH2_20.00X
100µm

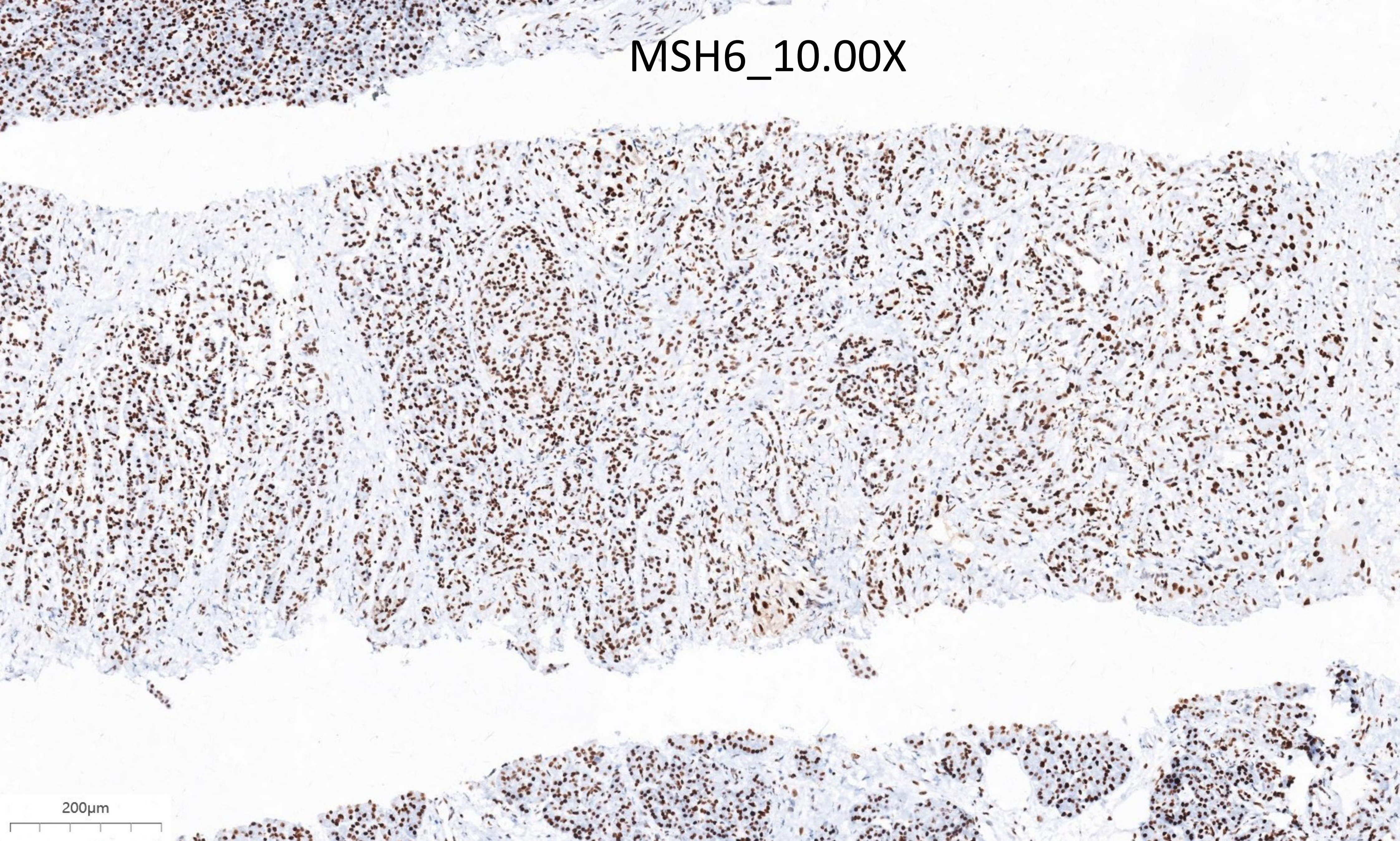
MSH6_10.00X
200µm

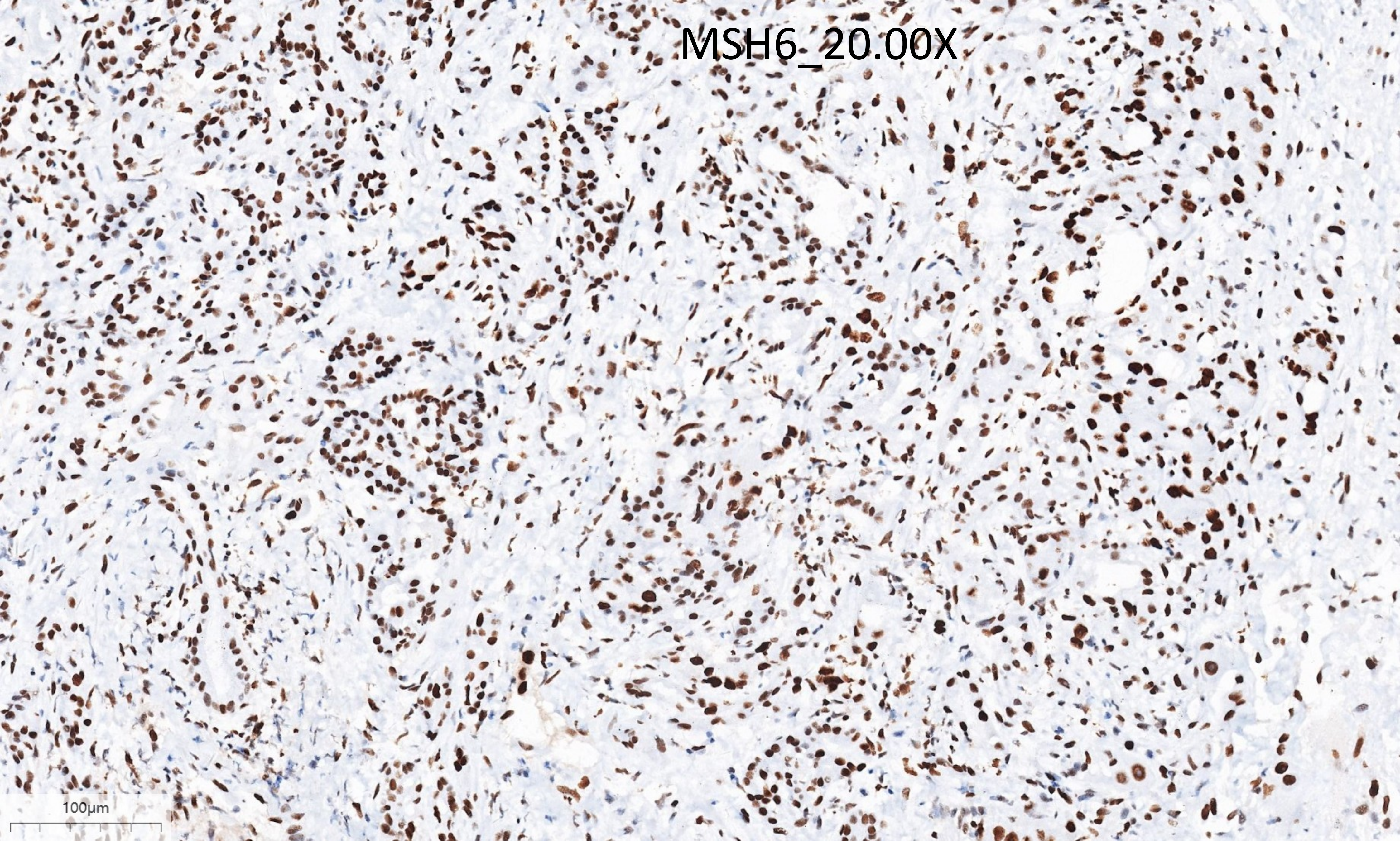
MSH6_20.00X
100μm

Mum-1_10.00X

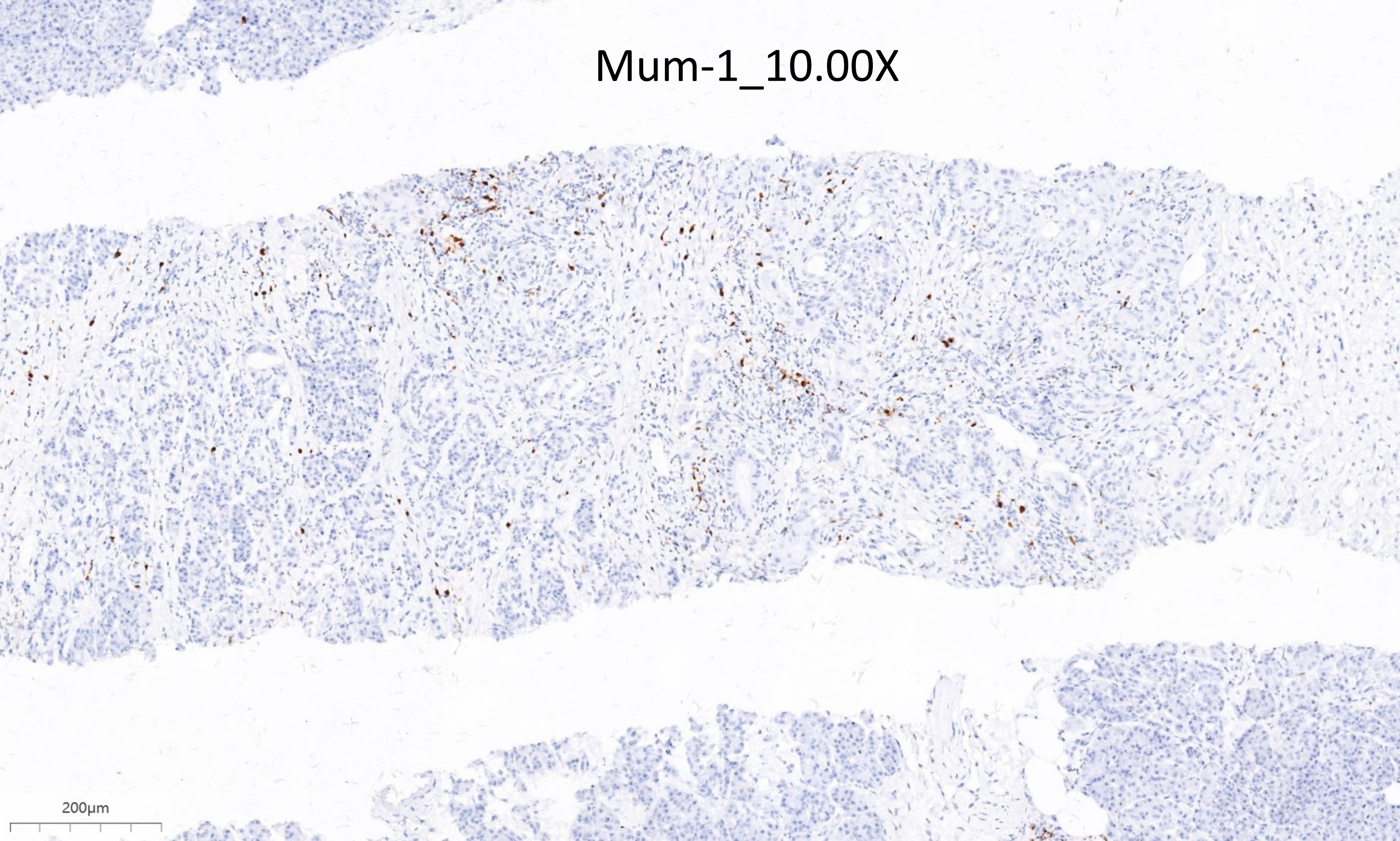

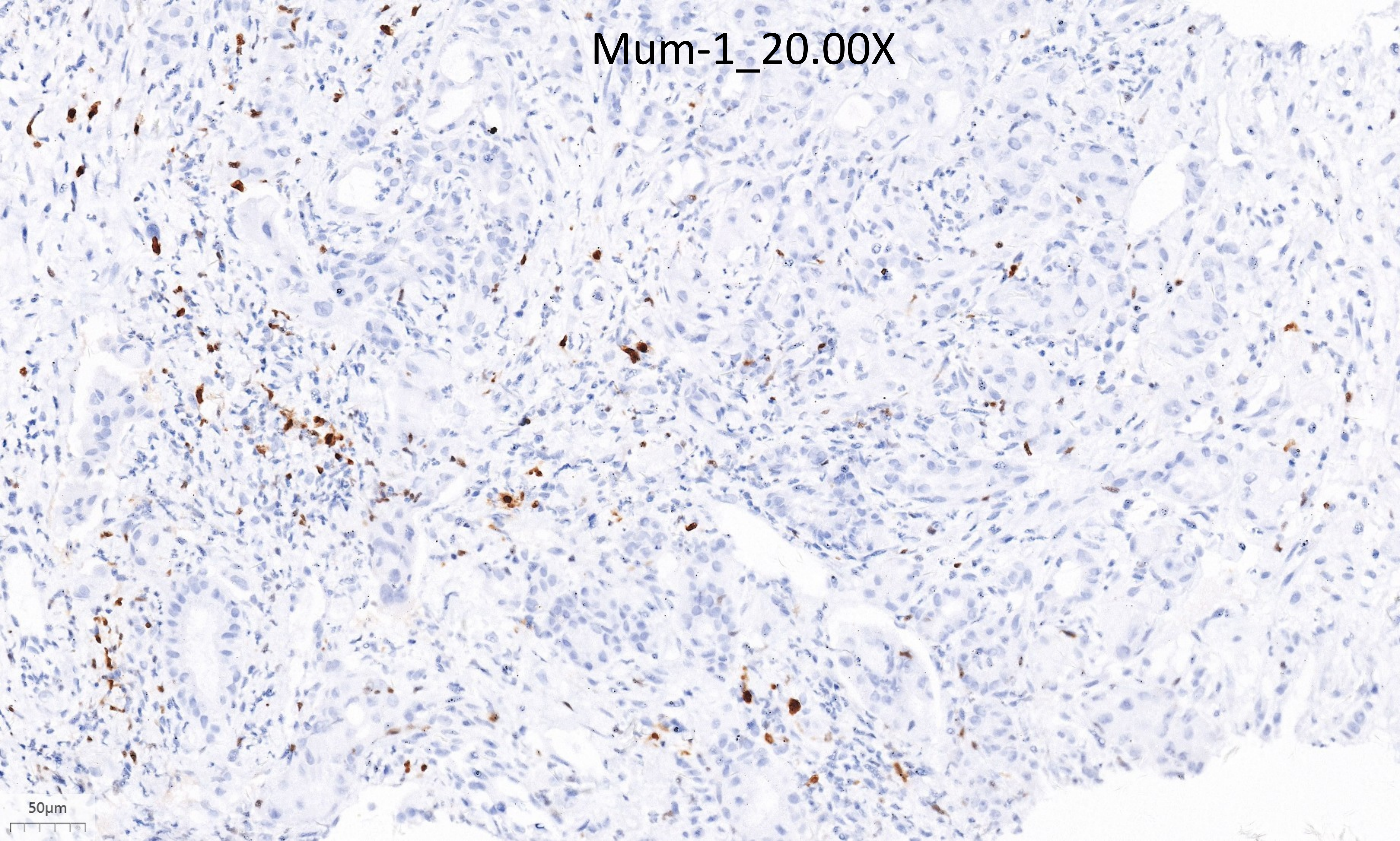
Mum-1_20.00X
50µm

P40_10.00X

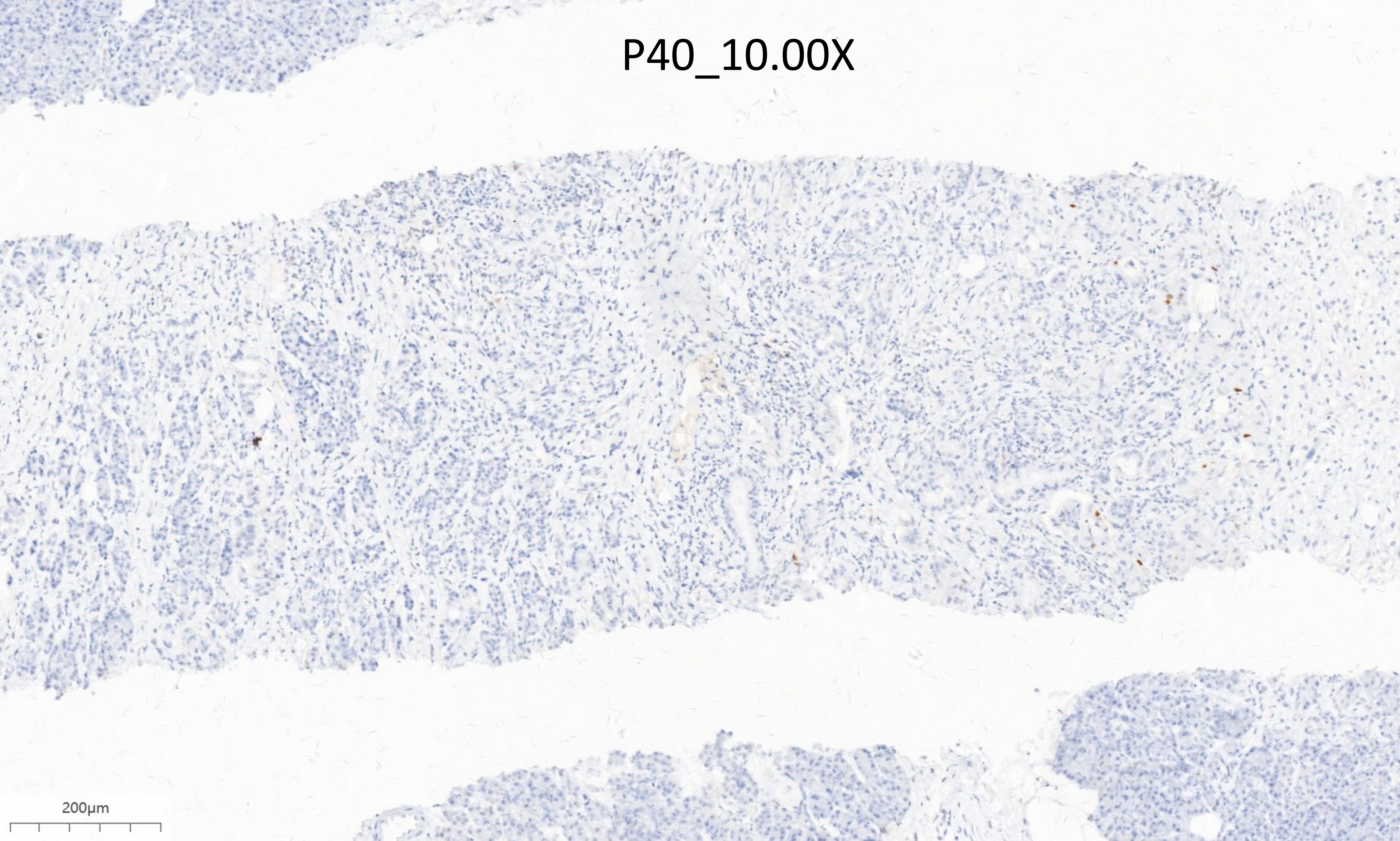

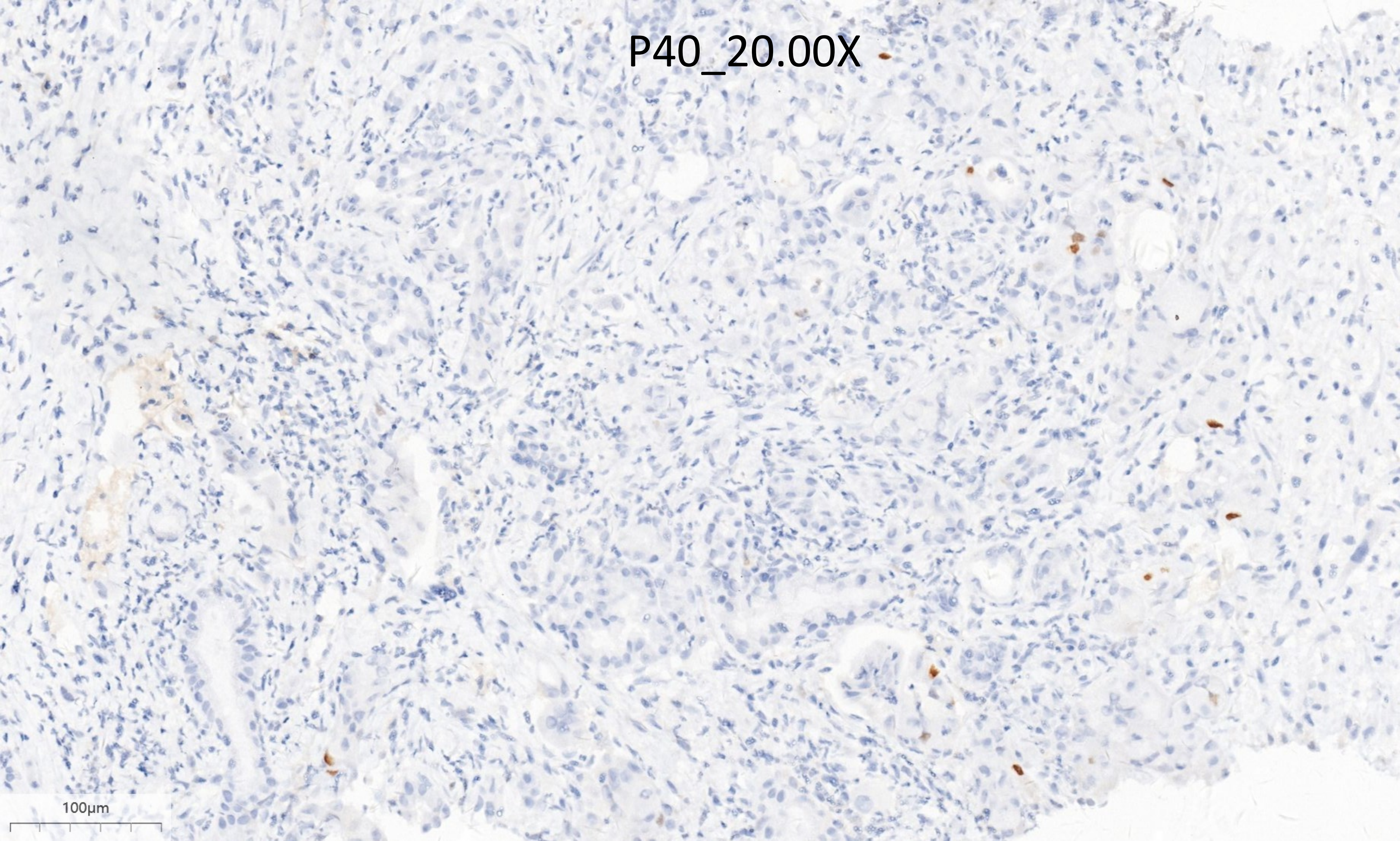

P40_20.00X
100µm

P53_10.00X

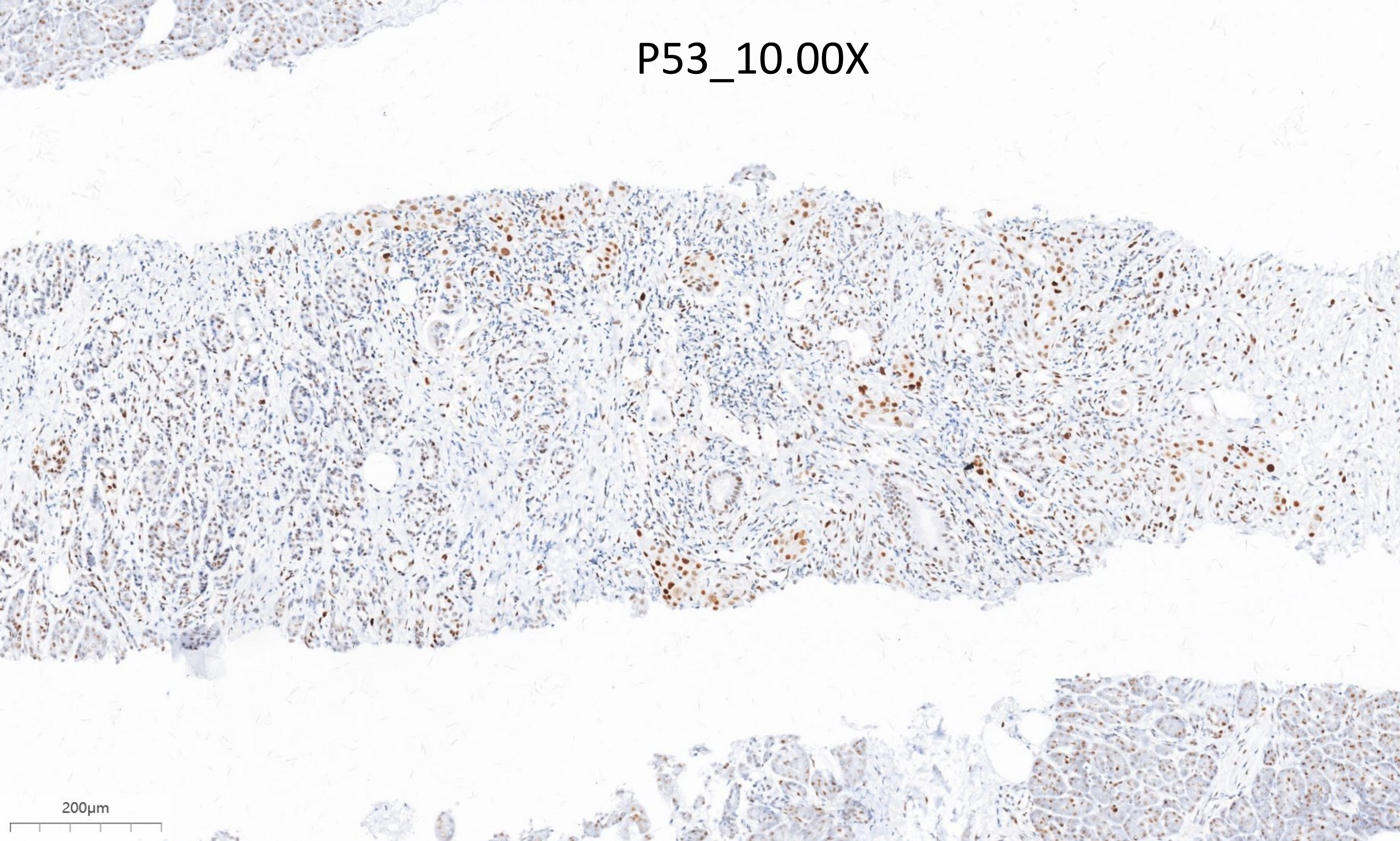

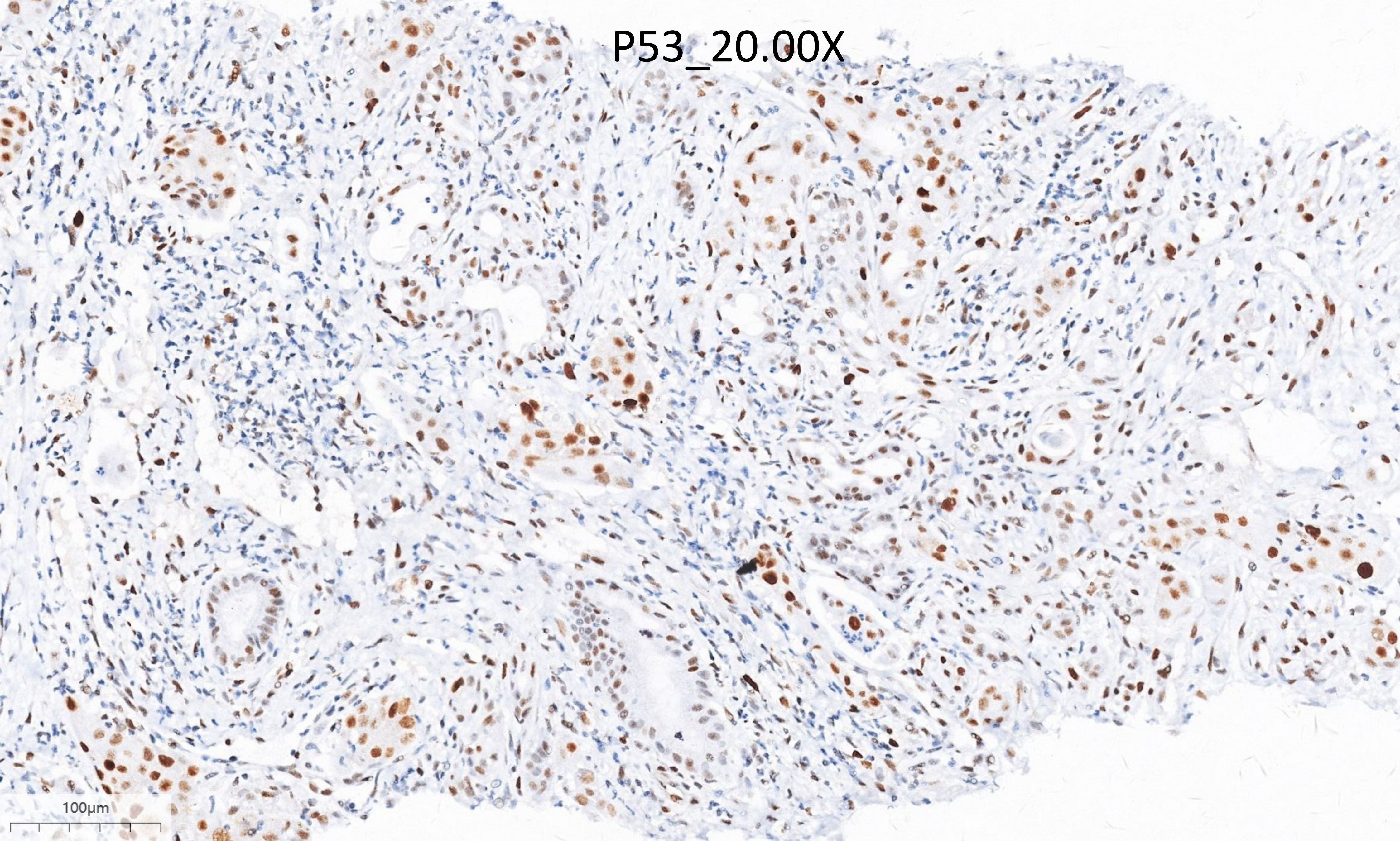
P53_20.00X
100µm

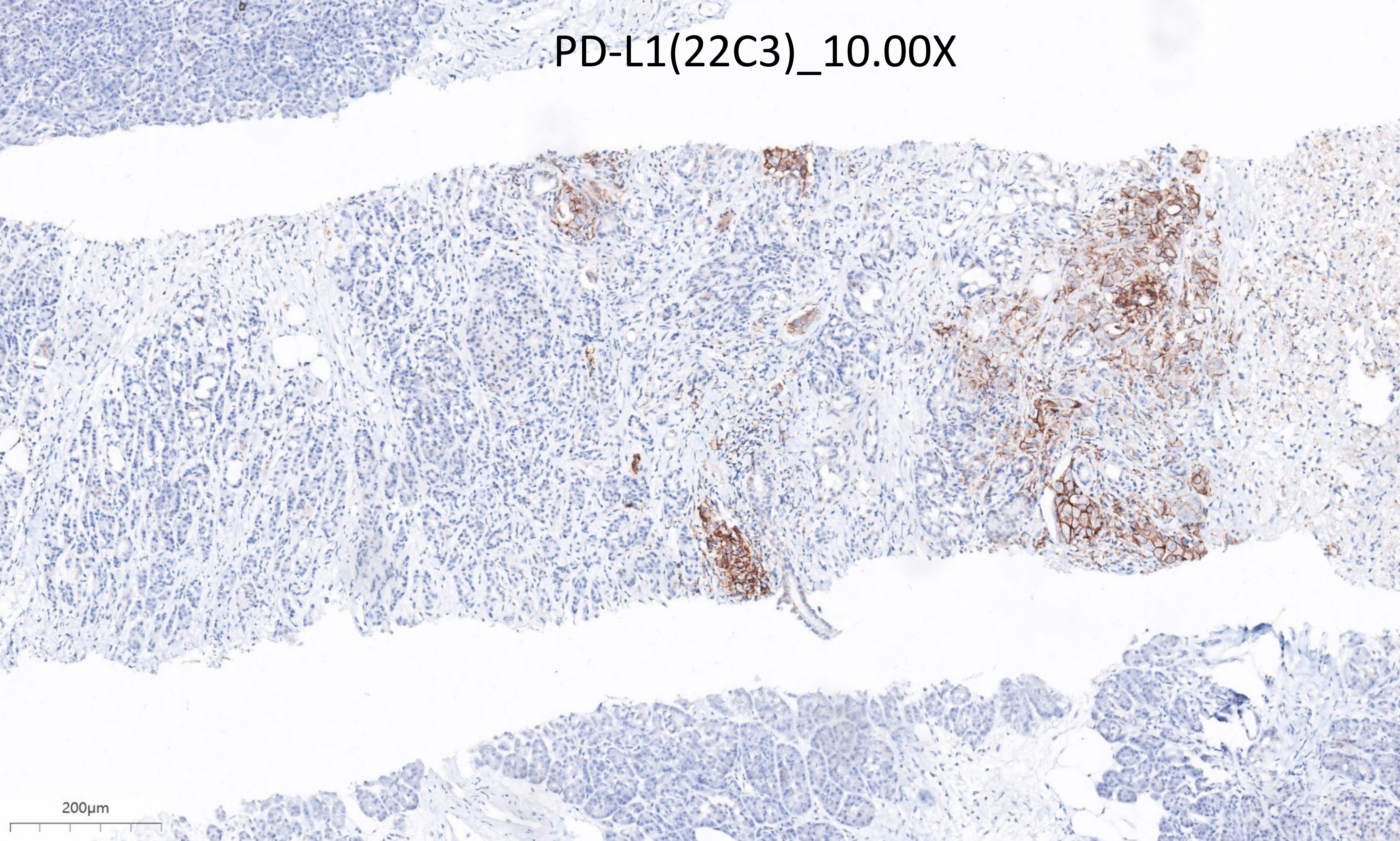
PD-L1(22C3)_10.00X
200μm

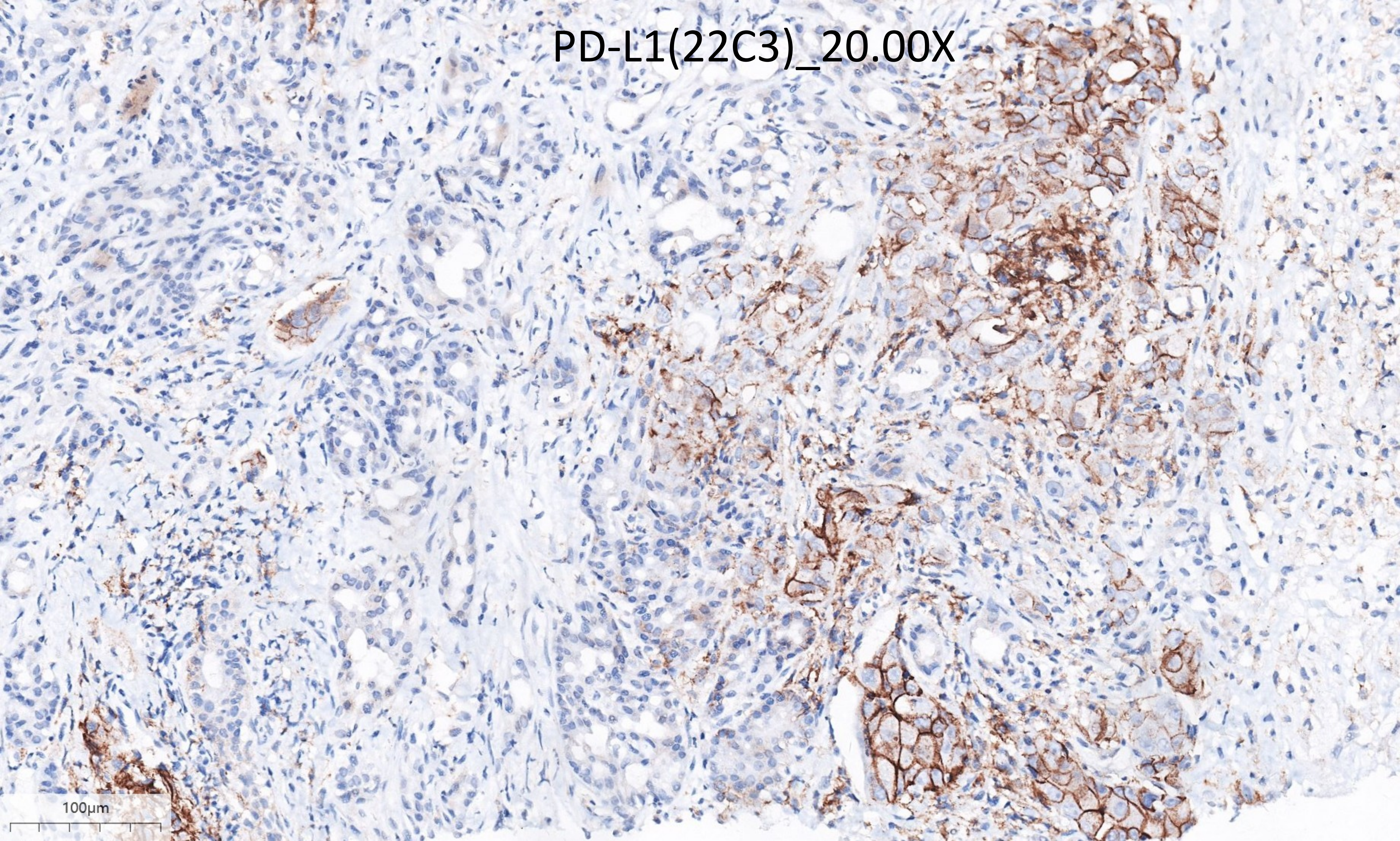
PD-L1(22C3)_20.00X
100µm

PMS2_10.00X

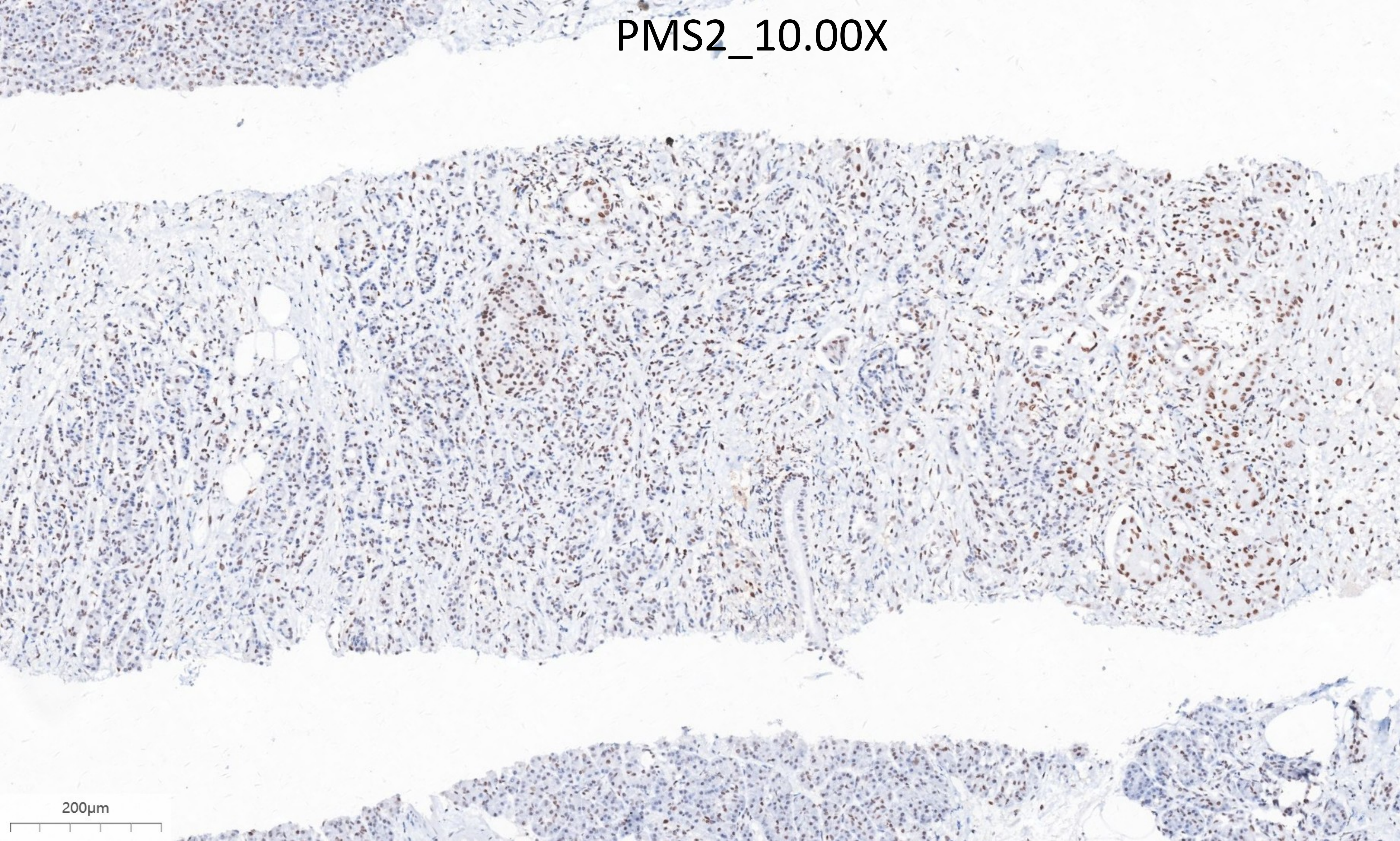

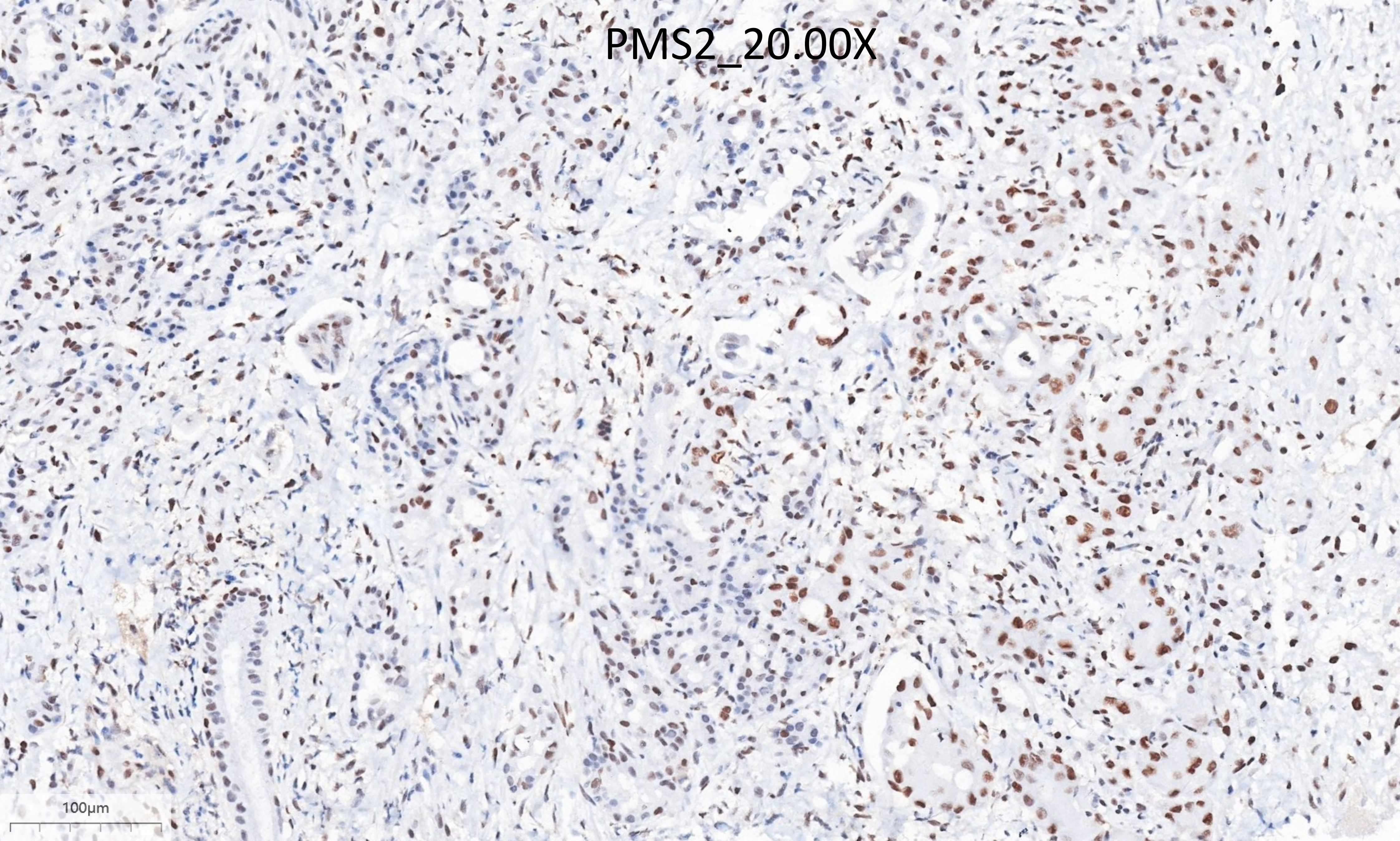
PMS2_20.00X
100µm

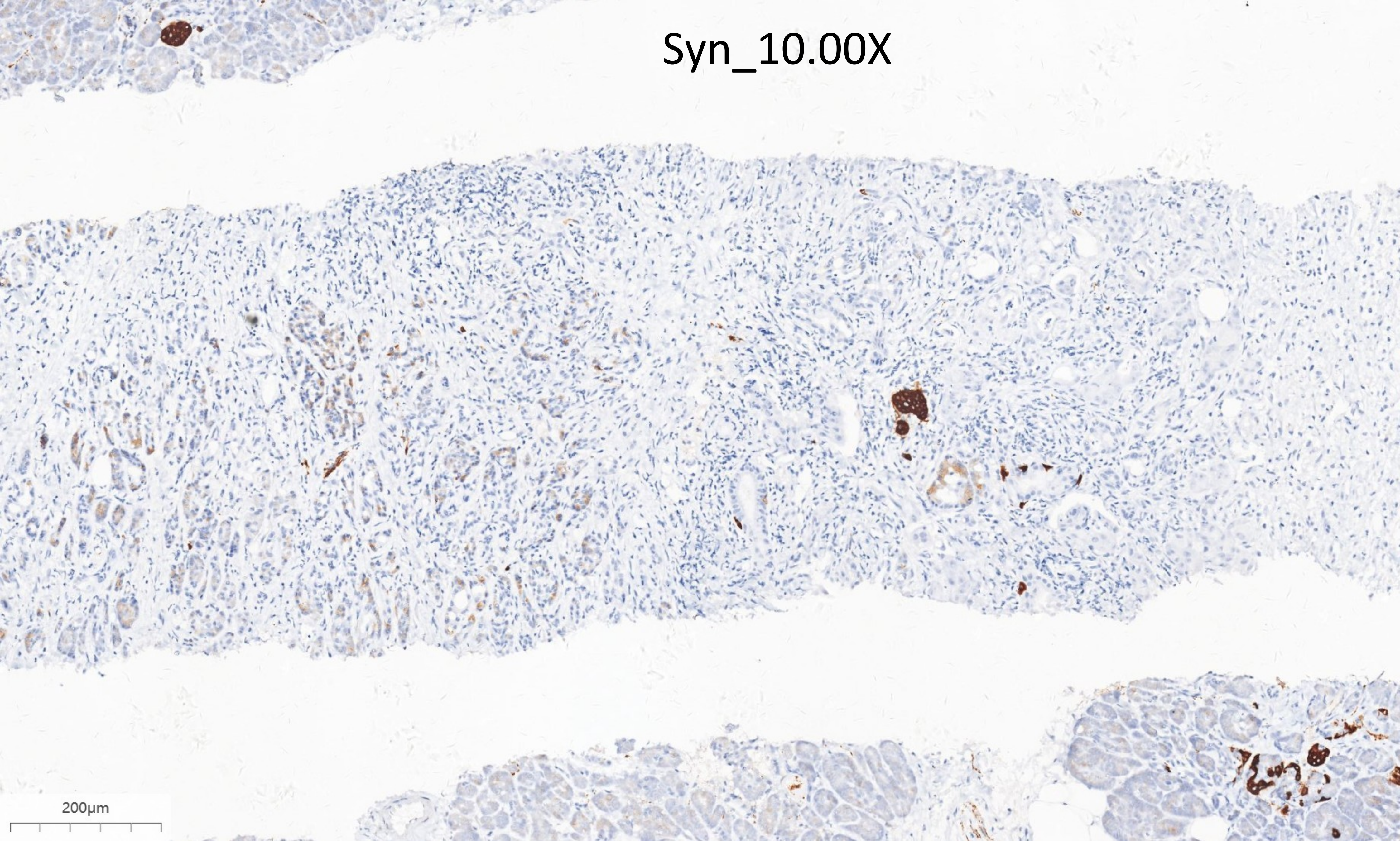
Syn_10.00X
200μm

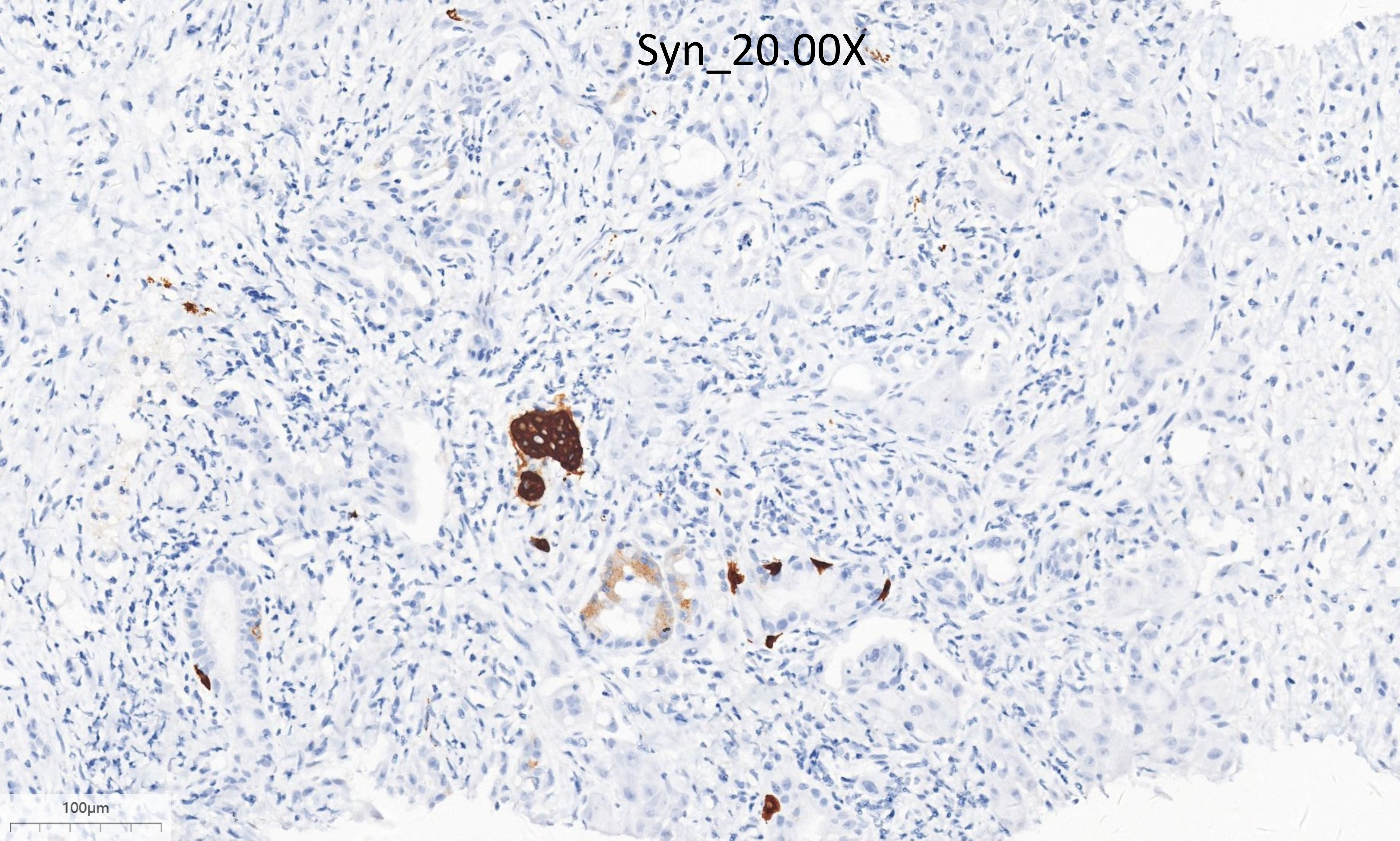
Syn_20.00X
100μm

Villin_10.00X

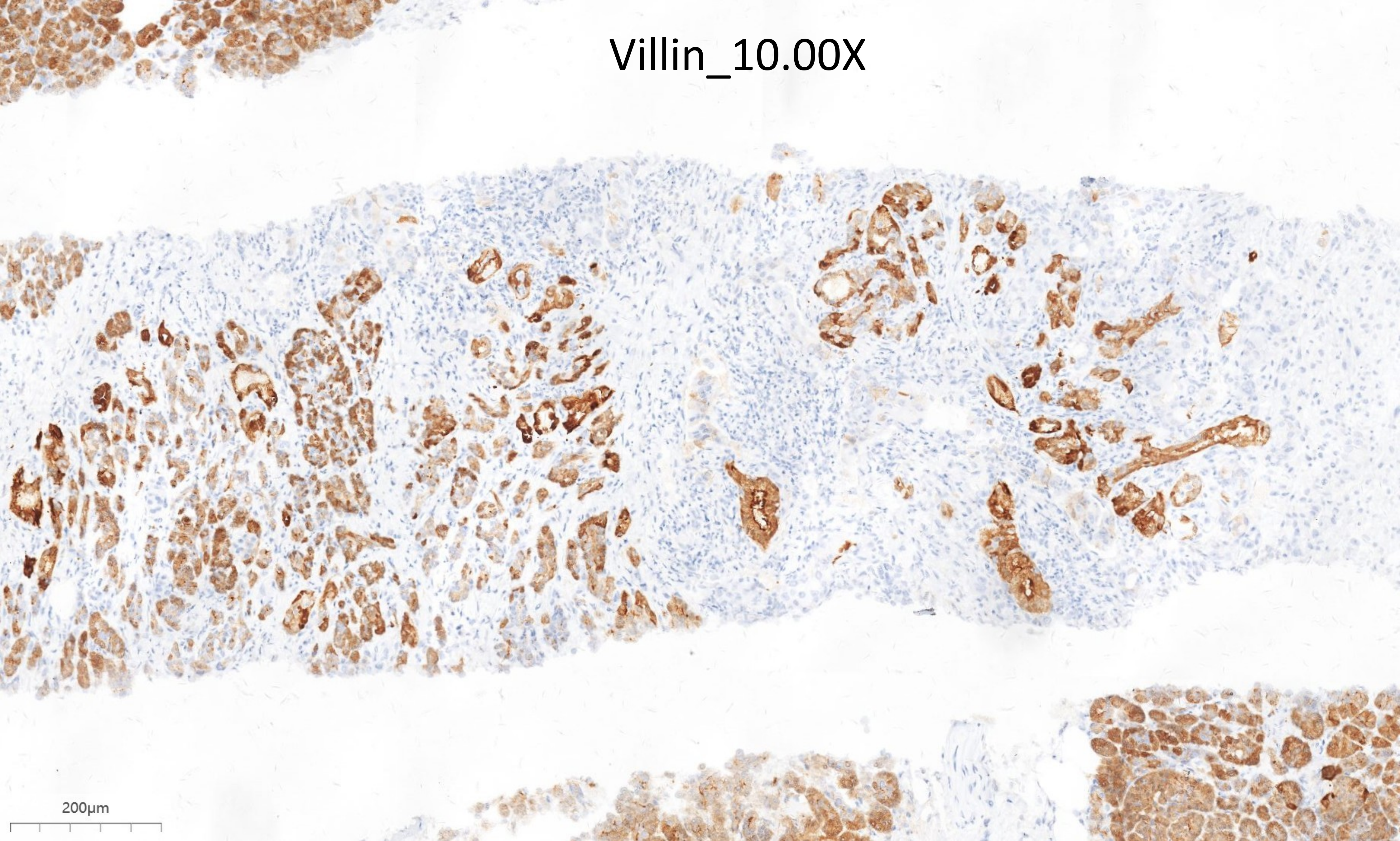

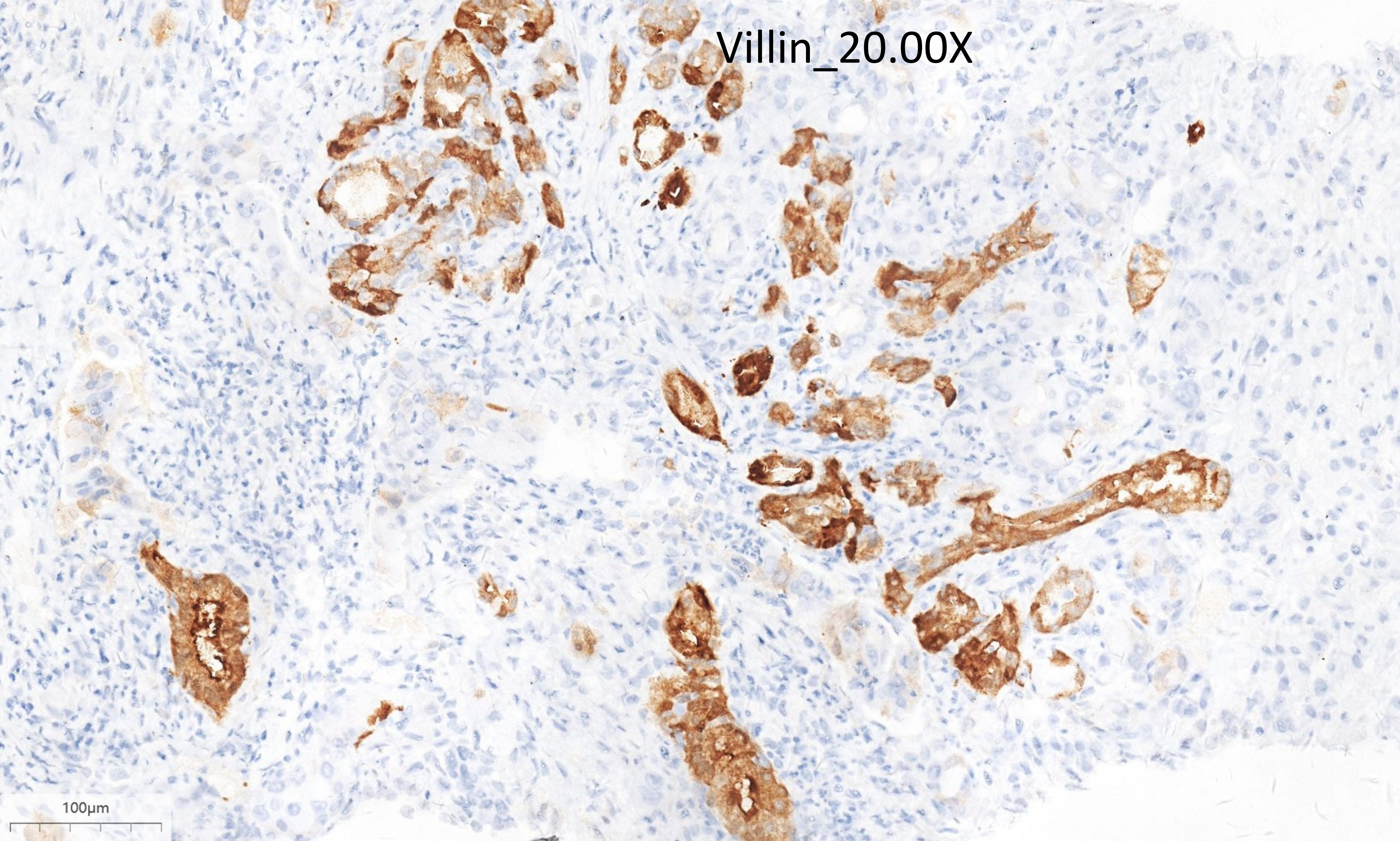
Villin_20.00X
100µm
