# Supplementary material for: Complete response to BRICS in Locally advanced pancreatic cancer (pMMR, CPS 30): a case report
Source: Front Immunol. 2026 Jan 21;17:1743752. doi: 10.3389/fimmu.2026.1743752 (PMC12867830; doi:10.3389/fimmu.2026.1743752)
Supplement: Supplementary Table 1 — Immunohistochemistry Markers. [file Table1.docx]

**Table S1. Immunohistochemistry Markers**

| **Category** | **Parameter** | **Result** |
| --- | --- | --- |
| **PD-L1** | CPS (Dako 22C3) | 30 |
| **Mismatch Repair Status** | MMR Status | pMMR  （MLH1+, MSH2+, MSH6+, PMS2+） |
| **Proliferation & Prognostic Markers** | Ki-67 Index (%) | 80 |
|  | p53 Expression | 60%, wild-type |
|  | CerbB-2 (HER2) | 1+ |
| **Diagnostic IHC Markers** | SMAD4 (DPC4) | ± (equivocal) |
|  | CA19-9 | ++ (moderate) |
|  | CK7 | +++ (strong) |
|  | CK19 | +++ (strong) |
|  | HNF1b | +++ (strong) |
|  | CK20 | ++ (moderate) |
|  | CEA | ++ (moderate) |
|  | P40 | + (weak) |
| **Exclusion Markers** | Synaptophysin (Syn) | Negative |
|  | INSM-1 | Negative |
|  | CD38 | Negative |
|  | IgG | Negative |
|  | IgG4 | Negative |

**Notes:**
PD-L1 CPS (Combined Positive Score) was calculated as the number of PD-L1–positive cells (tumor cells, lymphocytes, and macrophages) divided by the total number of viable tumor cells × 100.
Staining intensity was graded semiquantitatively as follows: –, negative; +, weak; ++, moderate; +++, strong.
